# Supplementary material for: Bioinspired one-pot furan-thiol-amine multicomponent reaction for making heterocycles and its applications
Source: Nat Commun. 2023 Jul 10;14:4086. doi: 10.1038/s41467-023-39708-7 (PMC10333318; doi:10.1038/s41467-023-39708-7)
Supplement: Supplementary file 1 — Supplementary Information [file 41467_2023_39708_MOESM1_ESM.pdf]

Supplementary Information
Bioinspired One-pot Furan-Thiol-Amine Multicomponent Reaction for Making Heterocycles and its Applications

Yuwen Wang[1], Patrick Czabala[1], Monika Raj[1*]

Table of content **Pages**

**I.**	General	2
**II.**	Materials	2
**III.**	Purification	2
**IV.**	Analytical Supplementary Methods	2-3
**V.**	Fmoc Solid-Phase Peptide Synthesis	3
**VI.**	**Fig. 1.** Optimization of the one-pot Furan-Thiol-Amine multicomponent reaction	3-6
**VII.**	**Fig. 2.** The reaction of furan with amine in the absence of thiol in acetone and water	6-8
**VIII.**	**Fig. 3.** The reaction of furan with amine in the absence of thiol in acetonitrile and water	8-11
**IX.**	**Fig. 4.** Substrate scope of Furan-Thiol-Amine reaction	12-29
**X.**	**Fig. 5.** Late-stage modification of 3-thio *N*-pyrrole	29-30
**XI.**	**Fig. 6.** Photophysical properties of 3-thio *N*-pyrrole	31
**XII.**	**Fig. 7.** Procedure for the modification of linear peptide containing lysine	32-33
**XIII.**	**Fig. 8.** Procedure for the modification of linear peptide containing cysteine	34-35
**XIV.**	**Fig. 9.** Procedure for the modification of linear peptides containing furan	36-39
**XV.**	**Fig. 10.** Procedure for the macrocyclization of a linear peptides	40-60
**XVI.**	**Fig. 11.** Procedure for stapling of peptides between two cysteine residues	61-62
**XVII.**	**Fig. 12.** Procedure for stapling of peptides between two lysine residues	63-64
**XVIII.**	**Fig. 13.** Optimization of modification of myoglobin	65-72
**XIX.**	**Fig. 14.** Stability of the modified myoglobin in different pH conditions	72
**XX.**	**Fig. 15.** Bioactivity assay of modified myoglobin	72-73
**XXI.**	**Fig. 16.** Homogeneous labeling and the site of modification on proteins	74-87
**XXII.**	**Fig. 17.** Duo modification of cysteine and lysine residues on aprotinin	88-89
**XXIII.**	**Fig. 18.** Modification of proteins	90-112
**XXIV.**	**Fig. 19.** Selective fluorophore labeling of proteins	112-117
**XXV.**	**Fig. 20.** Cyclization of proteins	118-119
**XXVI.**	**Fig. 21.** Dual modification of proteins	119-121
**XXVII.**	**Fig. 22.** Selective fluorophore labeling of proteins in complex cell lysates	121-125
**XXVIII.**	Supplementary References	125

Supplementary Notes

I. General. All commercial materials (Sigma-Aldrich, Fluka and Novabiochem) were used without further purification. All solvents were reagent or HPLC (Fisher) grade. All reactions were performed under air in glass vials. Yields refer to chromatographically pure compounds; percent conversions were obtained by comparing HPLC peak areas of products and starting materials. TLC, HPLC and MS were used to monitor reaction progress, and product elucidation was done using MS and NMR.

II. Materials. Fmoc-amino acids, Rink amide resin, Hydroxybenzotriazole (HOBT) and N,N′-diisopropylcarbodiimide (DIC) were obtained from CreoSalus (Louisville, Kentucky). Piperidine and trifluoroacetic acid (TFA) were obtained from Alfa Aesar (Ward Hill, Massachusetts). N,N-dimethylformamide (DMF), dichloromethane (DCM), methanol (MeOH) and acetonitrile (ACN) were obtained from VWR (100 Matsonford Road Radnor, Pennsylvania). Furan, sodium bicarbonate, N-Bromosuccinimide (NBS), 1-butylamine, 1-butanethiol, pyridine, thioglycolic acid and all other small molecules used in scope were obtained from Sigma. Commercially available proteins: myoglobin, lysozyme from chicken egg white, cytochrome C, alpha-lactalbumin, carbonic anhydrase, aprotinin, Insulin, lysozyme human, ubiquitin, transferrin, creatine kinase and BSA were obtained from Sigma. Cy5 azide, AZ680 and FITC dye were obtained from Thermo Fisher Scientific. For gel analysis: 30 % acrylamide mix, 1.5 M Tris buffer (pH 8.8), 10 % SDS, 10 % ammonium persulfate and ladders were obtained from Bio-rad.

III. Purification. HPLC: Purification of peptide starting materials was performed using high performance liquid chromatography (HPLC) on an Agilent 1100 series HPLC equipped with a C-18 reverse phase column with a particle size of 5 μm. All separations involved a mobile phase of 0.1 % formic acid in water (solvent A) and 0.1 % formic acid in acetonitrile (solvent B). The HPLC method used a linear gradient of 0-80% solvent B over 30 min at RT with a flow rate of 1 mL min^{-1}. The eluent was monitored by absorbance at 220 nm.

Supplementary Methods

IV. Instrumentation and sample analysis. NMR. ^{1}H and ^{13}C spectra were acquired at 25 °C in DMSO-d_6, $CDCl_3$ using an Agilent DD2 (400 MHz) spectrometer with a 3-mm He triple resonance (HCN) cryoprobe. All ^{1}H NMR chemical shifts (δ) were referenced relative to the residual DMSO-d_6 peak at 2.50 ppm, $CDCl_3$ peak at 7.28 ppm or internal tetramethylsilane (TMS) at 0.00 ppm. ^{13}C NMR chemical shifts were referenced to DMSO-d_6 at 39.52 ppm and $CDCl_3$ at 77.2 ppm. ^{13}C NMR spectra were proton decoupled. NMR spectral data are reported as chemical shift (multiplicity, coupling constants (*J*), integration). Multiplicity is reported as follows: singlet (s), doublet (d), doublet of doublets (dd), doublet of doublet of doublets (ddd), doublet of triplets (td), triplet (t) and multiplet (m). Coupling constants (J) are reported in hertz (Hz).

Analytical HPLC. Analytical HPLC chromatography (HPLC) was performed on an Agilent 1200 series HPLC equipped with a 5 μm pore size C-18 reversed-phase column. All separations involved mobile phase of 0.1 % formic acid in water (solvent A) and 0.1 % formic acid in acetonitrile (solvent B) run in linear gradients with a constant flow rate of 1 mL min^{-1}. The eluent was monitored with a

detection wavelength of 220 nm. **HPLC METHOD A:** Gradient: 0-80 % over 30 min. **HPLC METHOD B:** Gradient: 0-40% over 30 min.

LC/MS. High resolution LC-MS conditions for all purified peptides: Analyses were performed on an ultraperformance LC (UPLC) system (ACQUITY, Waters Corp., USA) coupled with a quadrupole time-of-flight mass spectrometer (Q-ToF Premier, Waters) with electrospray ionization (ESI) in positive mode using Mass lynx software (V4.1) or high-performance LC system (Agilent, 1100 series) coupled with triple quadrupole. Unless otherwise mentioned a sample was injected either onto a C4 or C18 column. C4 column: Phenomenex Aeris™ 3.6 µm WIDEPORE C4 200 Å, LC Column 50 x 2.1 mm with a 400 µL min^{-1} flow rate of mobile phase of solution A (90 % H_2O, 10 % acetonitrile and 0.1 % formic acid) and solution B (95 % acetonitrile, 5 % H_2O and 0.1 % formic acid). Beginning gradient: time- 0 min 10 % B; 5 min 28 % B; 20 min 38 % B; 22 min 90 % B.

HRMS. High resolution MS data were acquired on Thermo Exactive Plus using a heated electrospray source. The solution was infused at a rate of 10-25 µL min^{-1} electrospray using 3.3 kV. The typical settings were Capillary temp 320 °C. S-lens RF level was between 30-80 with an AGC setting of 1 E6. The maximum injection time was set to 50 ms. Spectra were taken at 140,000 resolutions at m/z 200 using Tune software and analyzed with Thermo's Freestyle software.

V. Fmoc Solid-Phase Peptide Synthesis (Fmoc-SPPS).[1] Peptides were synthesized using standard protocols. Peptides were synthesized manually on a 0.25 mmol scale using Rink amide resin. Resin was swollen with DCM for 30 min at RT. Fmoc was deprotected using 20 % piperidine–DMF for 15 min to obtain a deprotected resin. Fmoc protected amino acid (1.25 mmol, 5 equiv.) was coupled using HOBT (1.25 mmol, 5 equiv.) and DIC (1.25 mmol, 5 equiv.) in DMF for 15 min at RT. Fmoc-protected amino acids (0.75 mmol, 3 equiv.) were sequentially coupled on the resin using HOBT (1.25 mmol, 5 equiv.) and DIC (1.25 mmol, 5 equiv.) in DMF for 15 min at RT. Peptides were cleaved from the resin using 4 mL of a cocktail consisting of 95:2.5:2.5 trifluoroacetic acid : water : triisopropylsilane (TIS) for 2 h. The resin was removed by filtration and the resulting solution was concentrated. Peptides were precipitated with cold diethyl ether (3 x 10 mL) to obtain the crude product. Crude peptides were dissolved in ACN:H_2O and purified by HPLC.

Supplementary Figures

VI. Supplementary Fig. 1. Optimization of the one-pot Furan-Thiol-Amine multicomponent reaction to generate *N*-pyrroles

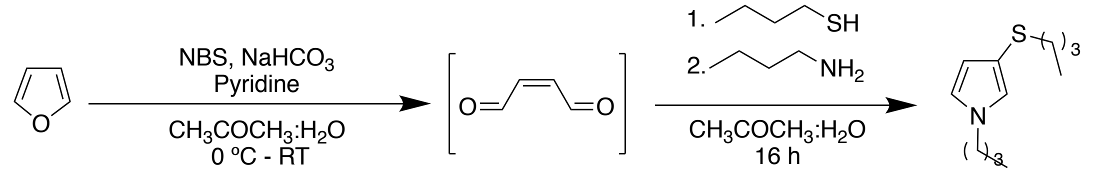

1 2a

Procedure A: Furan **1** (500 µL, 6.88 mmol, 1 equiv.) and sodium bicarbonate (575 mg, 6.88 mmol, 1 equiv.) were added to a solution of 60 mL CH_3COCH_3:H_2O (5:1). The reaction mixture was cooled to 0 °C and left to stir for 15 min. N-Bromosuccinimide NBS (1224 mg, 6.88 mmol, 1 equiv.) was

dissolved in a solution of 60 mL CH_3COCH_3:H_2O (5:1) and added to the reaction mixture dropwise. After the addition of NBS, the reaction mixture was left to stir for 10 min. Pyridine (1110 µL, 13.76 mmol, 2 equiv.) was then added to the reaction mixture, which was allowed to stir for 4 h at 0 °C and then used without further purification. The thiol derivative (4 equiv.) was then added, and the reaction mixture was incubated at 37 °C for 30 min before the addition of the amine derivative (2 equiv.). The reaction mixture was left to stir for 16 h at room temperature or 60 °C. The volatiles were removed under reduced pressure and separated by work-up using ethyl acetate and 1 M aqueous HCl (20:1). The organic layer was extracted, dried with Na_2SO_4, and the crude residue was further purified by column chromatography using a mixture of hexanes and ethyl acetate (9:1) as an eluent to get product **2a** in 36.8 % yield as a dark brown oil. **2a** was characterized by ^{1}H and ^{13}C NMR.

Procedure B: Furan **1** (500 µL, 6.88 mmol, 1 equiv.) and sodium bicarbonate (575 mg, 6.88 mmol, 1 equiv.) were added in a solution of 60 mL CH_3COCH_3:H_2O (5:1). The reaction mixture was cooled to 0 °C and left to stir for 15 min. N-Bromosuccinimide (1224 mg, 6.88 mmol, 1 equiv.) was dissolved in a solution of CH_3COCH_3:H_2O (50 mL and 10 mL, respectively) and added to the reaction mixture dropwise. After the addition of NBS, the reaction mixture was left to stir for 10 min. Pyridine (1110 µL, 13.76 mmol, 2 equiv.) was then added to the reaction mixture, which was allowed to stir for 2 h and then used without further purification. This reaction was also done on a lower scale of furan (100 µL, 1.38 mmol, 1 equiv.) in some cases and all other reaction conditions were lowered accordingly.1-butanethiol (4 equiv.) was then added, and the reaction mixture was incubated at 37 °C for 30 min before the addition of 1-butylamine (2 equiv.). The reaction mixture was left to stir for 16 h at room temperature. The volatiles were removed under reduced pressure and separated by work-up using ethyl acetate and 1 M aqueous HCl (20:1). The organic layer was extracted, dried with Na_2SO_4, and the crude residue was further purified by column chromatography using a mixture of hexanes and ethyl acetate (9:1) as an eluent to get product **2a** in 58.2 % yield as a dark brown oil. **2a** was characterized by ^{1}H and ^{13}C NMR.

Procedure C: Furan **1** (500 µL, 6.88 mmol, 1 equiv.) and sodium bicarbonate (575 mg, 6.88 mmol, 1 equiv.) were added in a solution of 60 mL CH_3COCH_3:H_2O (5:1). The reaction mixture was cooled to 0 °C and left to stir for 15 min. N-Bromosuccinimide (NBS) (1224 mg, 6.88 mmol, 1 equiv.) was dissolved in a solution of CH_3COCH_3:H_2O (50 mL and 10 mL, respectively) and added to the reaction mixture dropwise. After the addition of NBS, the reaction mixture was left to stir for 10 min. Pyridine (1110 µL, 13.76 mmol, 2 equiv.) was then added to the reaction mixture, which was allowed to stir for 4 h and then used without further purification. This reaction was also done on a lower scale of furan (100 µL, 1.38 mmol, 1 equiv.) in some cases and all other reaction conditions were lowered accordingly. Thiol derivative (4 equiv.) was mixed with amine derivative (4 equiv.) and added to the reaction mixture together. The reaction mixture was left to stir for 16 h at room temperature. The volatiles were removed under reduced pressure and separated by work-up using ethyl acetate and 1 M aqueous HCl (20:1). The organic layer was extracted, dried with Na_2SO_4, and the crude residue was further purified by column chromatography using a mixture of hexanes and ethyl acetate (9:1) as an eluent to get product **2a** in 36.4 % yield as a dark brown oil. **2a** was characterized by ^{1}H and ^{13}C NMR.

Procedure D: Furan **1** (500 µL, 6.88 mmol, 1 equiv.) and sodium bicarbonate (575 mg, 6.88 mmol, 1 equiv.) were added in a solution of 60 mL CH_3COCH_3:H_2O (5:1). The reaction mixture was cooled to 0 °C and left to stir for 15 min. N-Bromosuccinimide NBS (1224 mg, 6.88 mmol, 1 equiv.) was dissolved in a solution of 60 mL CH_3COCH_3:H_2O (5:1) and added to the reaction mixture dropwise. After the addition of NBS, the reaction mixture was left to stir for 10 min. Pyridine (1110 µL, 13.76 mmol, 2 equiv.) was then added to the reaction mixture, which was allowed to stir for 4 h and then used without further purification. The thiol derivative (4 equiv.) was then added, and the reaction mixture was incubated at 37 °C for 30 min before the addition of amine derivative (2 equiv.). The reaction mixture was left to stir for 16 h at room temperature or 60 °C. The volatiles were removed under reduced pressure and separated by work-up using ethyl acetate and 1 M aqueous HCl (20:1). The organic layer was extracted, dried with Na_2SO_4, and the crude residue was further purified by column chromatography using a mixture of hexanes and ethyl acetate (9:1) as an eluent to get product **2a** in 63.4 and 71.7 % yields (room temperature and 60 °C, respectively) as a dark brown oil. **2a** was characterized by ^{1}H and ^{13}C NMR.

NMR data for Compound 2a

1-butyl-3-(butylthio)-1*H*-pyrrole **2a**: ^{1}**H NMR** (400 MHz, Chloroform-*d*) δ 6.72 (t, *J* = 2.0 Hz, 1H), 6.61 (t, *J* = 2.5 Hz, 1H), 6.18 (dd, *J* = 2.8, 1.7 Hz, 1H), 3.84 (t, *J* = 7.1 Hz, 2H), 2.66 (dd, *J* = 8.0, 6.7 Hz, 2H), 1.80 – 1.70 (m, 2H), 1.62 – 1.54 (m, 2H), 1.44 (ddd, *J* = 9.9, 7.5, 5.9 Hz, 2H), 1.33 (h, *J* = 7.3 Hz, 2H), 0.94 (dt, *J* = 10.6, 7.4 Hz, 6H). ^{13}**C NMR** (101 MHz, Chloroform-*d*) δ 124.52, 121.19, 113.15, 111.88, 49.58, 37.20, 33.44, 31.81, 21.74, 19.86, 13.75, 13.64.

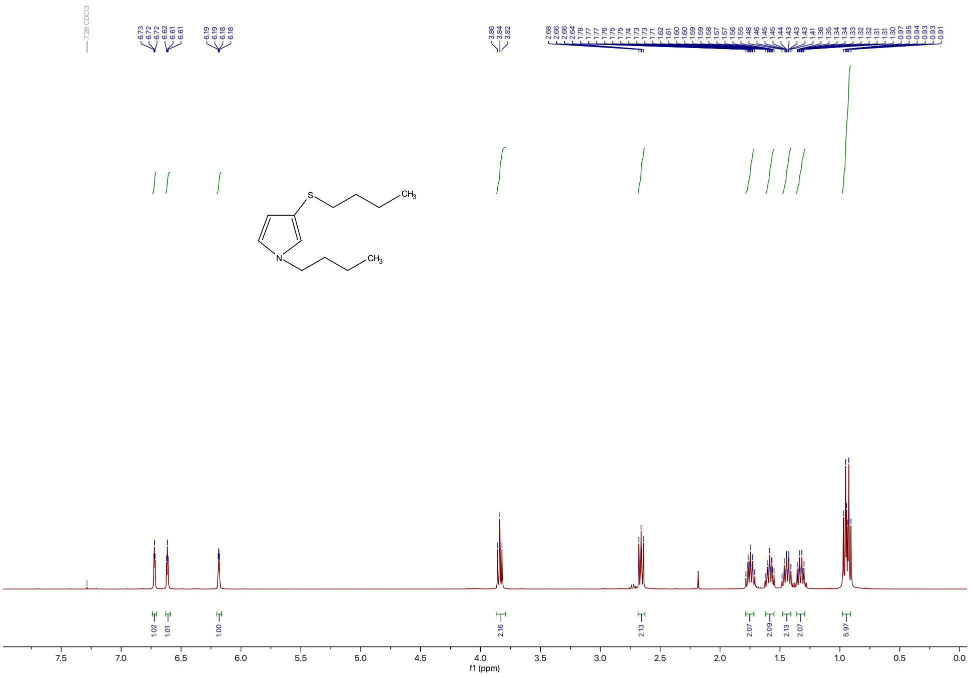

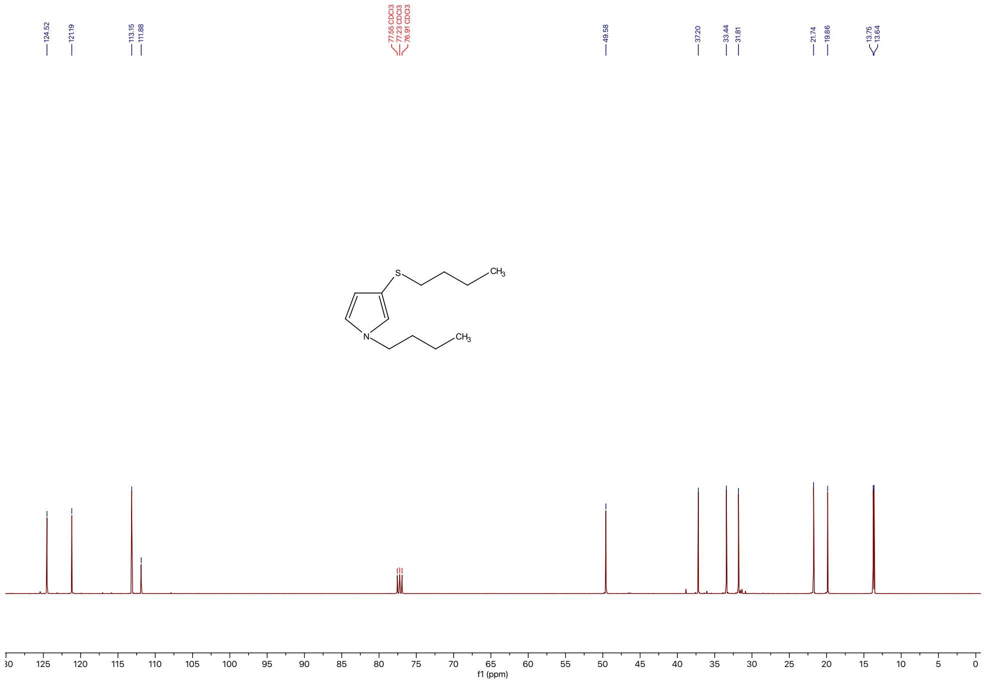

VII. Supplementary Fig. 2. The reaction of furan with amine in the absence of thiol in acetone and water as solvent.

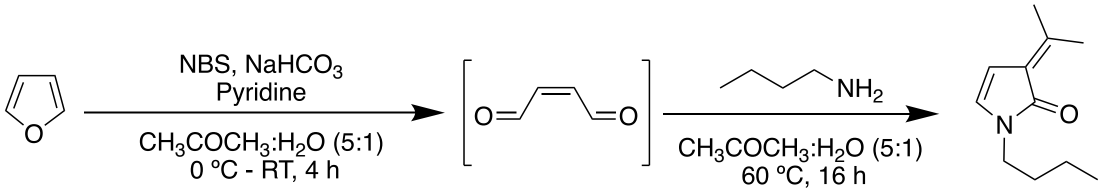

Furan **1** (500 μL, 6.88 mmol, 1 equiv.) and sodium bicarbonate (575 mg, 6.88 mmol, 1 equiv.) were added to in a solution of 60 mL CH_3COCH_3:H_2O (5:1). The reaction mixture was cooled to 0 °C and left to stir for 15 min. N-Bromosuccinimide (1224 mg, 6.88 mmol, 1 equiv.) was dissolved in a solution of 60 mL CH_3COCH_3:H_2O (5:1) and added to the reaction mixture dropwise. After the addition of NBS, the reaction mixture was left to stir for 10 min. Pyridine (1110 μL, 13.76 mmol, 2 equiv.) was then added to the reaction mixture, which was allowed to stir for 4 h and then used without further purification. 1-butylamine (2 equiv.) was then added, and the reaction mixture was left to stir for 16 h at 60 °C. The volatiles were removed under reduced pressure and separated by work-up using ethyl acetate and 1 M aqueous HCl (20:1). The organic layer was extracted, dried with Na_2SO_4, and the crude residue was further purified by column chromatography using a mixture of hexanes and ethyl acetate (7:3) as an eluent and product **3** was obtained in 13.6 % yield as a dark brown oil and characterized by ^{1}H, ^{13}C NMR and HRMS.

NMR data for Compound 3

1-butyl-3-(propan-2-ylidene)-1,3-dihydro-2*H*-pyrrol-2-one **3**: **^{1}H NMR** (400 MHz, Chloroform-*d*) δ 6.40 (d, *J* = 4.8 Hz, 1H), 5.73 (d, *J* = 4.9 Hz, 1H), 3.47 (t, *J* = 7.2 Hz, 2H), 2.40 (s, 3H), 2.03 (s, 3H), 1.58 – 1.54 (m, 2H), 1.35 – 1.28 (m, 2H), 0.90 (t, *J* = 7.4 Hz, 3H). **^{13}C NMR** (101 MHz, Chloroform-*d*) δ 167.11, 151.31, 129.50, 127.79, 100.98, 41.65, 31.28, 23.98, 20.36, 19.98, 13.70.

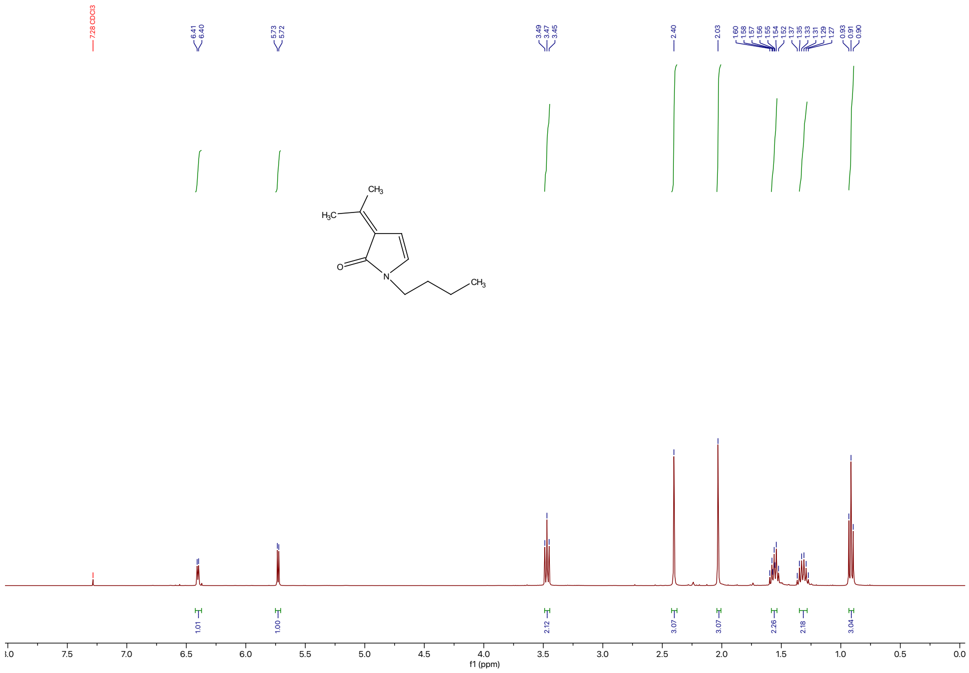

7.26 CDCl3
6.41
6.40
5.73
5.72
3.49
3.47
3.45
2.40
2.03
1.60
1.58
1.57
1.56
1.55
1.54
1.52
1.37
1.35
1.33
1.31
1.29
1.27
0.93
0.91
0.90
CH3
H3C
O
N
CH3
1.01
1.00
2.12
3.07
3.07
2.26
2.18
3.04
f1 (ppm)

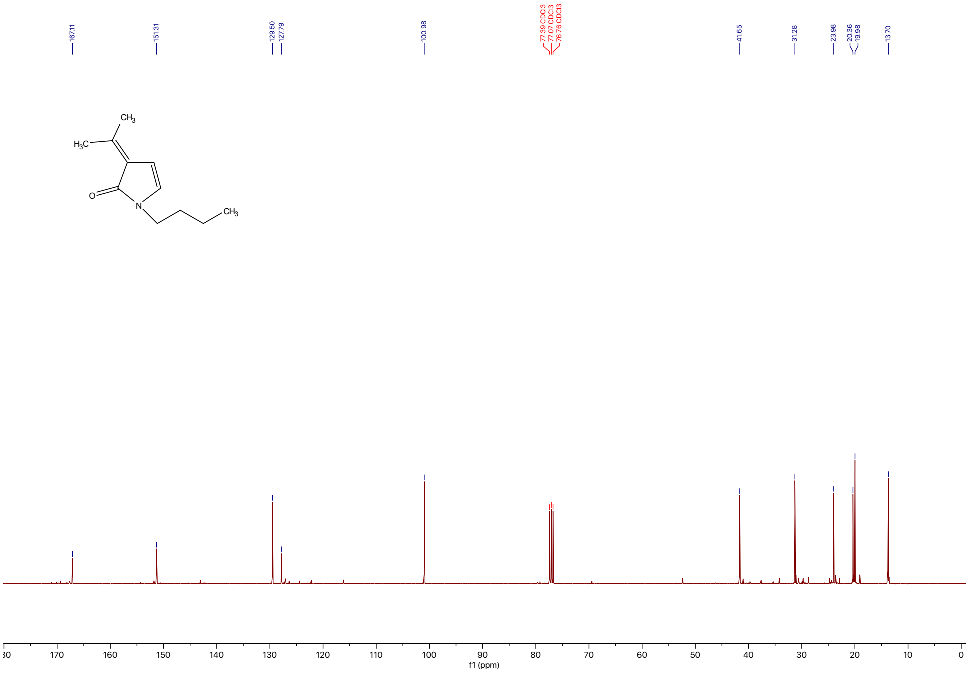

167.11
151.31
129.50
127.79
100.98
77.39 CDCl3
77.07 CDCl3
76.76 CDCl3
41.65
31.28
23.98
20.36
19.98
13.70
CH3
H3C
O
N
CH3
f1 (ppm)

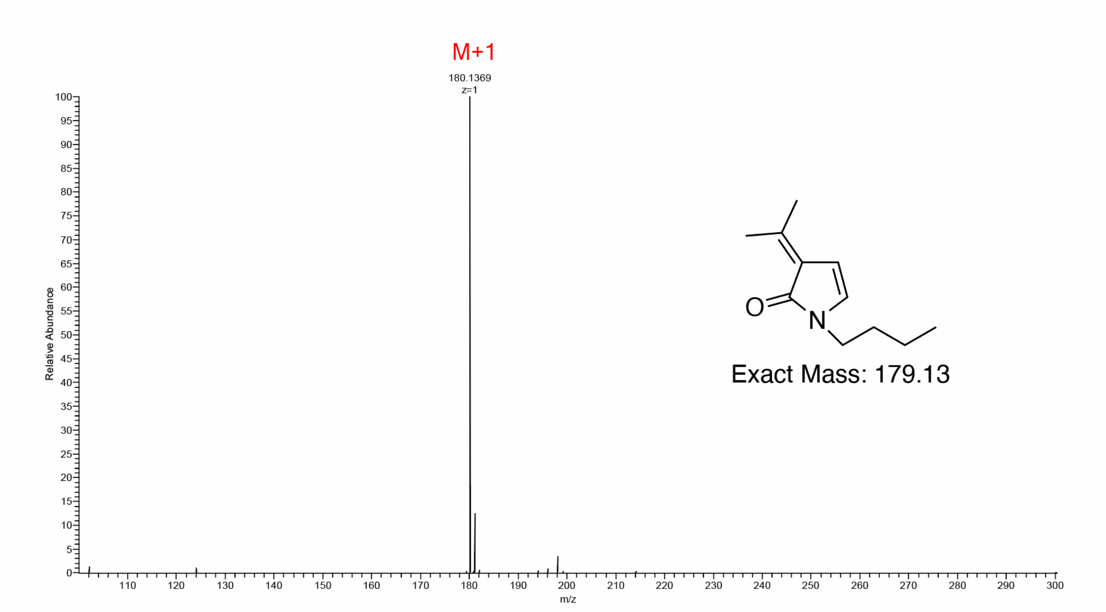

VIII. Supplementary Fig. 3. The reaction of furan with amine in the absence of thiol in acetonitrile and water as solvent.

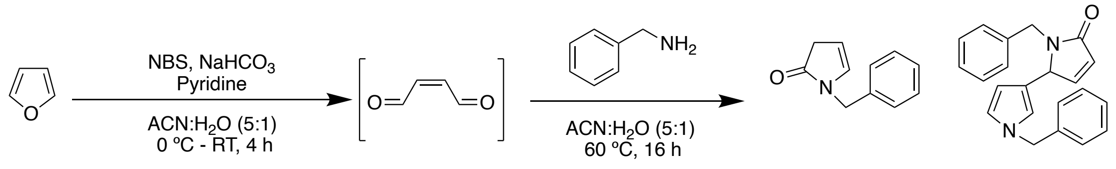

Furan (500 µL, 6.88 mmol, 1 equiv.) and sodium bicarbonate (575 mg, 6.88 mmol, 1 equiv.) were added in a solution of 60 mL CH_3COCH_3:H_2O (5:1). The reaction mixture was cooled to 0 °C and left to stir for 15 min. N-Bromosuccinimide (1224 mg, 6.88 mmol, 1 equiv.) was dissolved in a solution of 60 mL CH_3COCH_3:H_2O (5:1) and added to the reaction mixture dropwise. After the addition of NBS, the reaction mixture was left to stir for 10 min. Pyridine (1110 µL, 13.76 mmol, 2 equiv.) was then added to the reaction mixture, which was allowed to stir for 4 h and then used without further purification. Benzylamine (2 equiv.) was then added, and the reaction mixture was left to stir for 16 h at 60 °C. The volatiles were removed under reduced pressure and separated by work-up using ethyl acetate and 1 M aqueous HCl (20:1). The organic layer was extracted, dried with Na_2SO_4, and the crude residue was further purified by column chromatography using a mixture of hexanes and ethyl acetate to get products **4** and **5** in 6.3 % and 9.8 % yield, respectively. The products **4** and **5** were characterized by ^{1}H and ^{13}C NMR. **5** was further characterized with 2D NMR and HRMS.

Label	Solvent	Derivatives	Eluent (Hexanes: EA)	Characteristics	Yield (%)
4	ACN:H_2O (5:1)	benzylamine	2:8	dark yellow oil	6.3
5	ACN:H_2O (5:1)	benzylamine	5:5	dark yellow oil	9.8

NMR data for Compound 4

1-benzyl-1,3-dihydro-2*H*-pyrrol-2-one **4**: ^{1}**H NMR** (400 MHz, Chloroform-*d*) δ 7.36 – 7.31 (m, 3H), 7.27 (d, *J* = 1.7 Hz, 1H), 7.25 (s, 1H), 7.09 – 7.05 (m, 1H), 6.25 (dt, *J* = 6.0, 1.9 Hz, 1H), 4.66 (s, 2H), 3.90 (t, *J* = 1.9 Hz, 2H). ^{13}**C NMR** (101 MHz, Chloroform-*d*) δ 171.41, 142.84, 137.28, 128.78, 128.01, 127.96, 127.61, 52.29, 45.99.

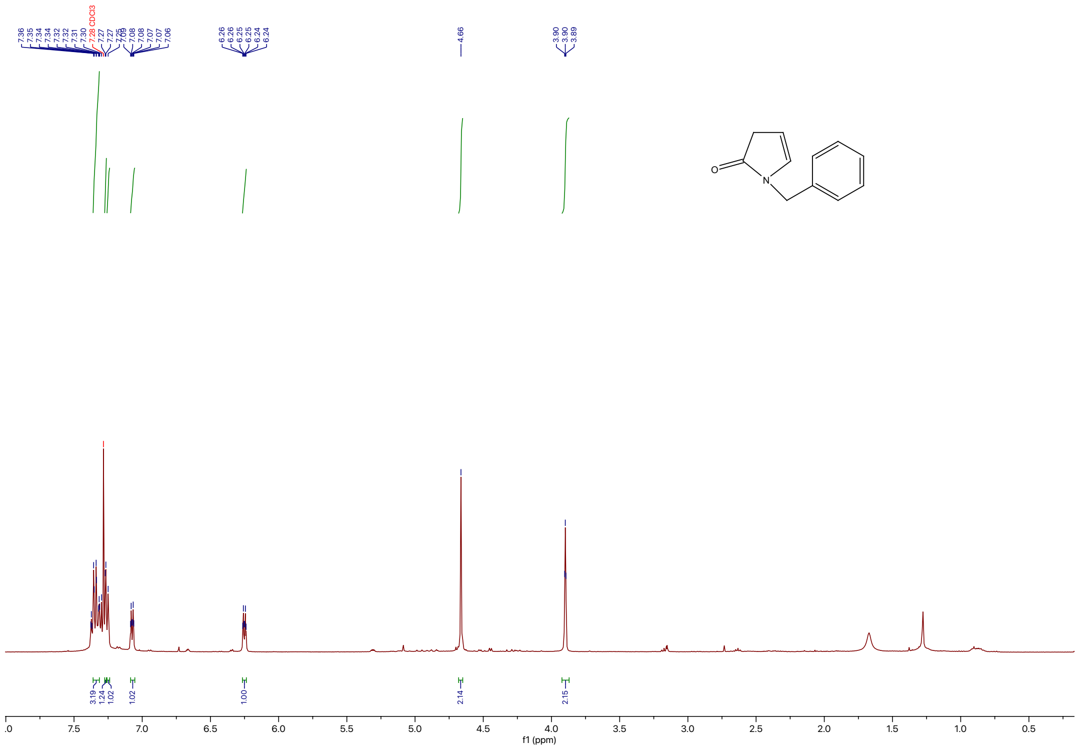

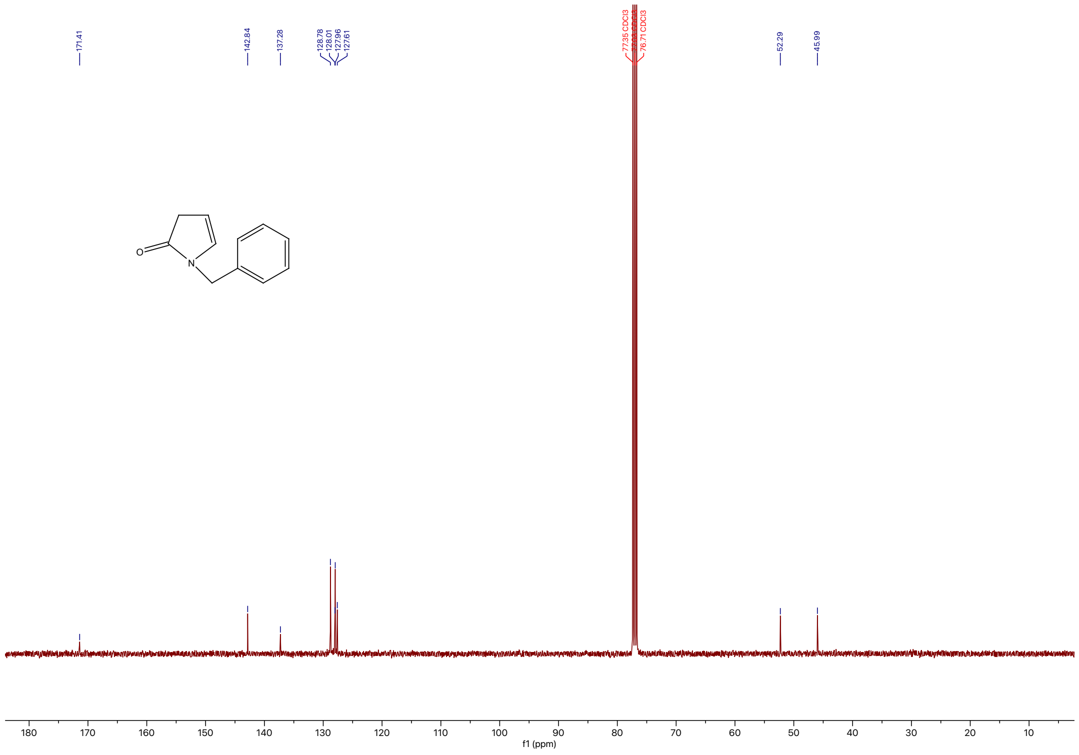

NMR data for Compound 5

1,1'-dibenzyl-1,2-dihydro-1'H,5H-[2,3'-bipyrrol]-5-one **5**: **^{1}H NMR** (400 MHz, Chloroform-*d*) δ 7.41 – 7.37 (m, 2H), 7.36 – 7.34 (m, 1H), 7.30 (d, *J* = 6.4 Hz, 1H), 7.28 – 7.24 (m, 2H), 7.15 (td, *J* = 6.2, 3.2 Hz, 4H), 7.05 (dd, *J* = 5.9, 1.8 Hz, 1H), 6.69 (t, *J* = 2.5 Hz, 1H), 6.53 (t, *J* = 2.0 Hz, 1H), 6.24 (dd, *J* = 5.8, 1.8 Hz, 1H), 5.94 (dd, *J* = 2.8, 1.8 Hz, 1H), 5.13 (d, *J* = 15.0 Hz, 1H), 5.05 (s, 2H), 4.90 (t, *J* = 1.9 Hz, 1H), 3.77 (d, *J* = 15.0 Hz, 1H). **^{13}C NMR** (101 MHz, Chloroform-*d*) δ 170.95, 148.46, 137.97, 137.61, 128.85, 128.55, 128.16, 127.94, 127.22, 127.08, 125.93, 122.35, 120.38, 117.08, 107.56, 59.70, 53.57, 43.14.

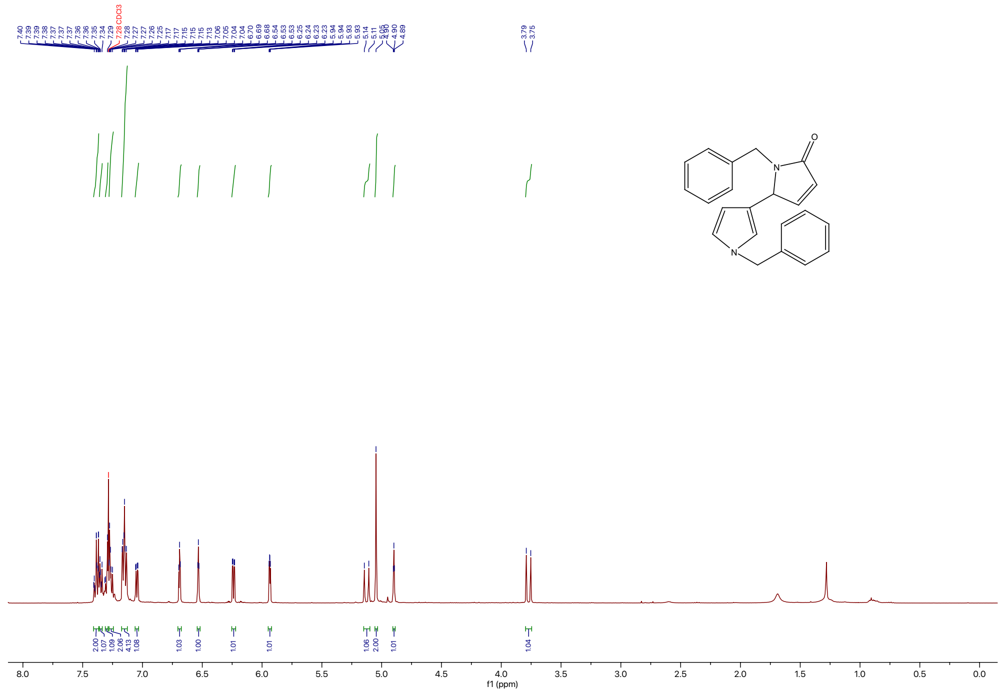

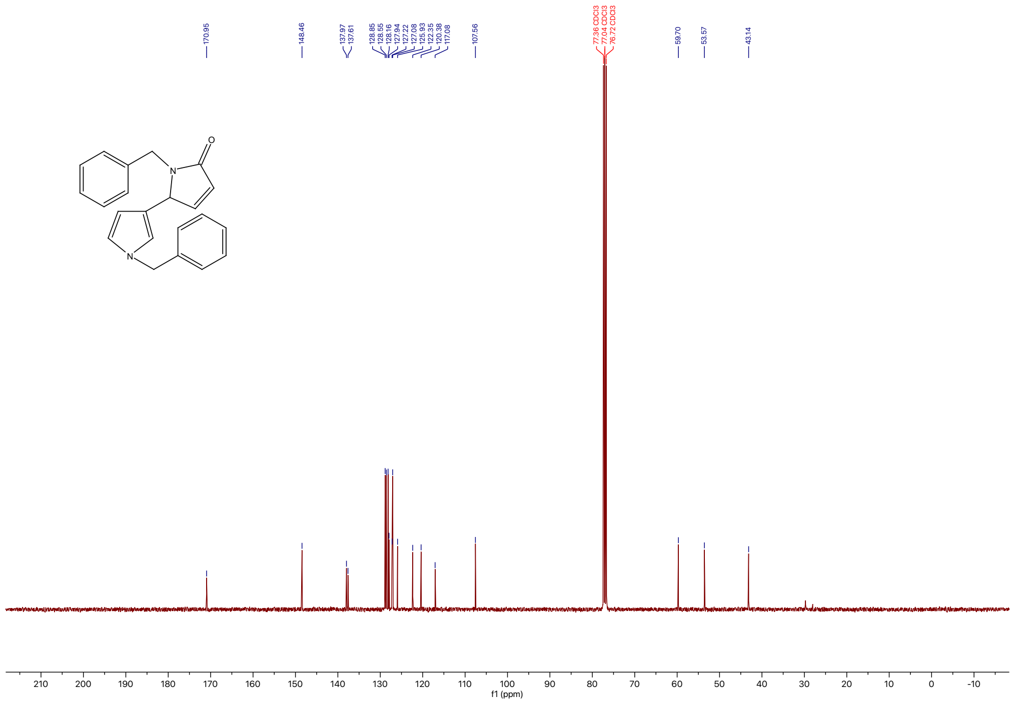

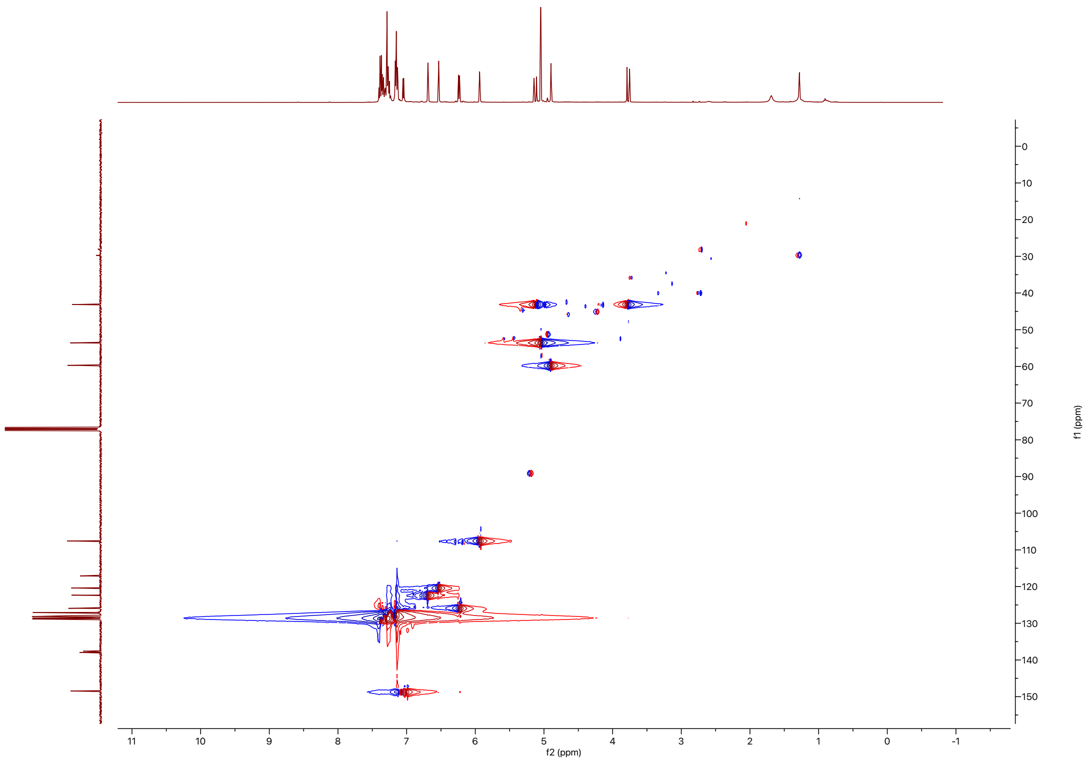

HRMS data for Compound 5

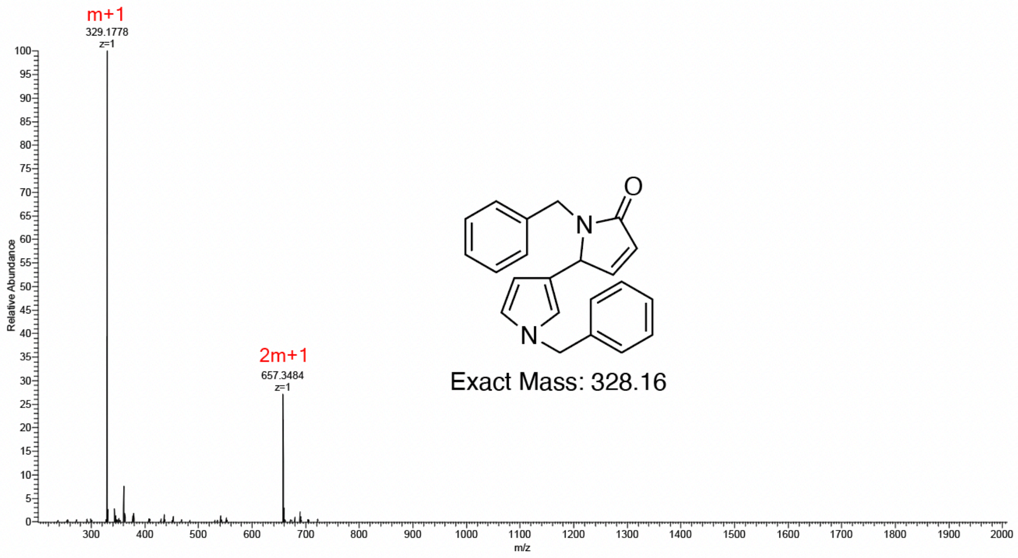

IX. Supplementary Fig. 4. Substrate scope of Furan-Thiol-Amine reaction

Furan or furan derivative (1.38 mmol or 6.88 mmol, 1 equiv.) and sodium bicarbonate (1 equiv.) were added to in a solution of 60 mL or 12 mL CH_3COCH_3:H_2O (5:1). The reaction mixture was cooled to 0 °C and left to stir for 15 min. N-Bromosuccinimide (1 equiv.) was dissolved in a solution of 60 mL or 12 mL CH_3COCH_3:H_2O (5:1) and added to the reaction mixture dropwise. Afterwards, the reaction mixture was left to stir for 10 min, and pyridine (2 equiv.) was added to the reaction mixture. The reaction mixture was stirred for 4 h and used without further purification. To the reaction mixture, thiol derivative (4 equiv.) was added, and the reaction mixture was incubated at 37°C for 30 min before the addition of amine derivative (2 equiv.) In some cases, the thiol and amine were added together, with no observed effect on yield. The reaction mixture was left to stir for 16 h at 60 °C. The volatiles were removed under reduced pressure and purified in work-up using ethyl acetate and 1 M aqueous HCl (20:1). The organic layer was extracted, dried with Na_2SO_4, and crude residue was further purified by column chromatography using a mixture of hexanes and ethyl acetate as the eluent to get products **2b-m**.

Thiol Scope: Reaction of different thiols with furan and butylamine (2b-2e)

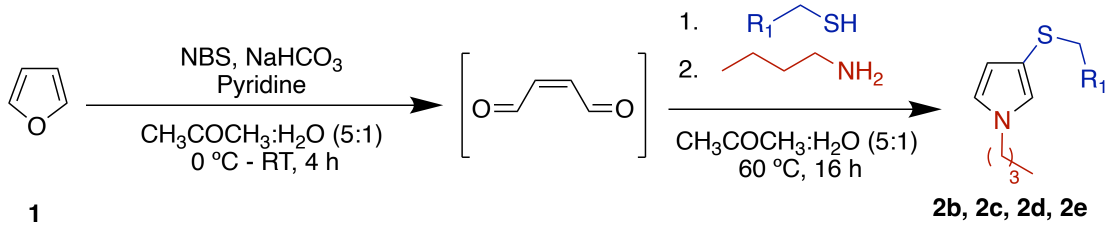

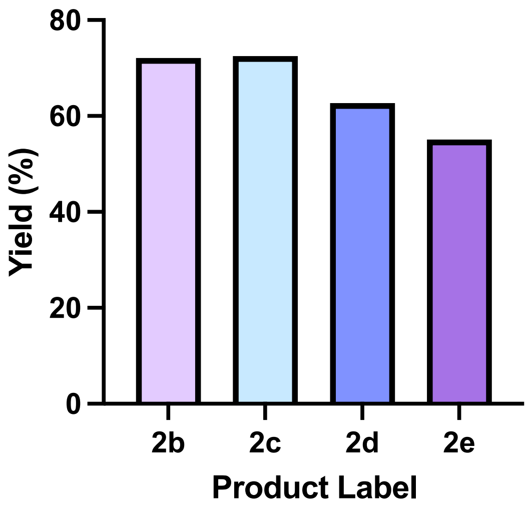

Label	Derivatives	Eluent (Hexanes: EA)	Characteristics	Yield (%)
2b	2-Butanethiol	9:1	brown oil	72.1
2c	Thioglycolic acid	5:5	brown oil	72.5
2d	2-Mercaptoethanol	5:5	brown oil	62.7
2e	N-Acetyl-L-cysteine methyl ester	3:7	Dark yellow oil	55.1

NMR data for Compound 2b

butyl-3-(*sec*-butylthio)-1*H*-pyrrole **2b**: ^{1}**H NMR** (400 MHz, Chloroform-*d*) δ 6.72 (t, *J* = 2.0 Hz, 1H), 6.61 (t, *J* = 2.5 Hz, 1H), 6.17 (dd, *J* = 2.8, 1.7 Hz, 1H), 3.84 (t, *J* = 7.1 Hz, 2H), 2.71 (h, *J* = 6.7 Hz, 1H), 1.80 – 1.69 (m, 2H), 1.66 – 1.55 (m, 1H), 1.45 (dp, *J* = 14.2, 7.2 Hz, 1H), 1.37 – 1.27 (m, 2H), 1.22 (d, *J* = 6.8 Hz, 3H), 1.02 (t, *J* = 7.4 Hz, 3H), 0.94 (t, *J* = 7.4 Hz, 3H). ^{13}**C NMR** (101 MHz, Chloroform-*d*) δ 126.04, 121.00, 114.71, 109.59, 49.57, 46.00, 33.45, 29.30, 20.71, 19.83, 13.64, 11.69.

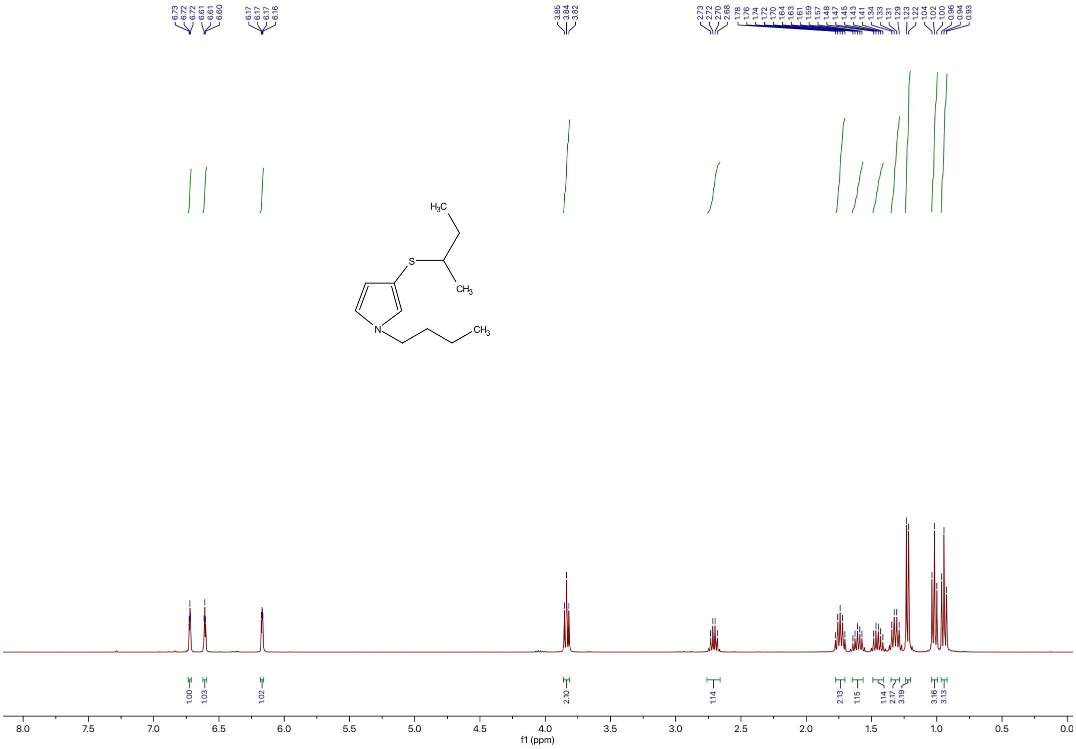

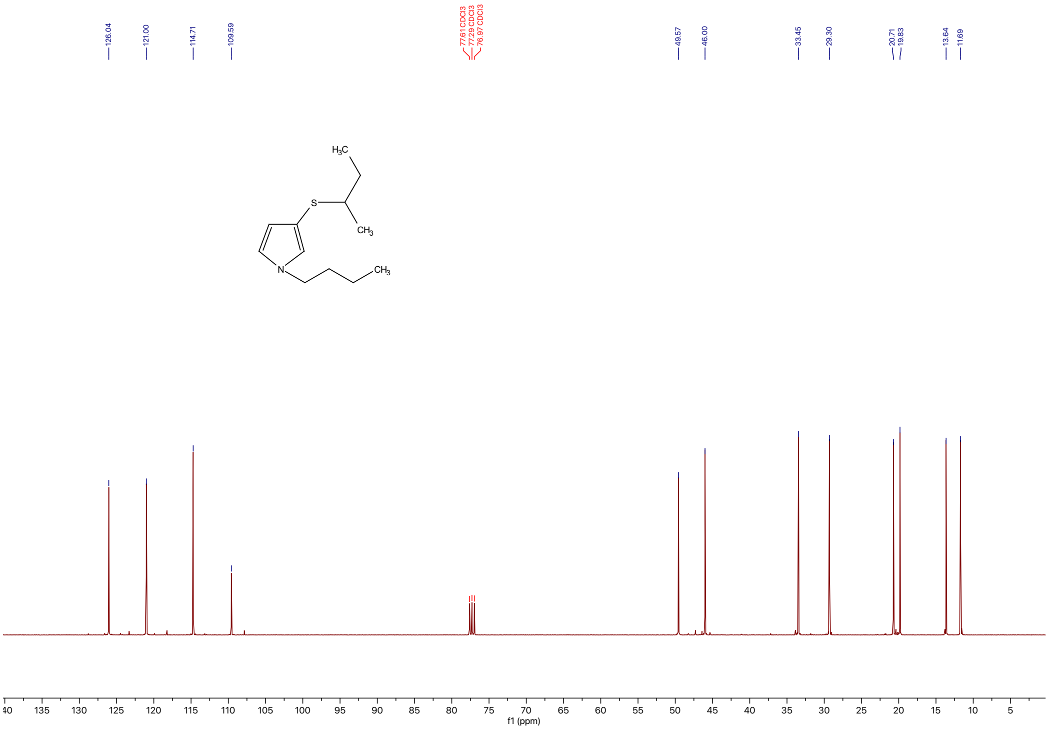

NMR data for Compound 2c

2-((1-butyl-1*H*-pyrrol-3-yl)thio)acetic acid **2c**: **^{1}H NMR** (400 MHz, Chloroform-*d*) δ 6.82 (t, *J* = 2.0 Hz, 1H), 6.61 (d, *J* = 2.5 Hz, 1H), 6.24 (d, *J* = 1.1 Hz, 0H), 3.84 (s, 1H), 3.41 (s, 2H), 1.78 – 1.70 (m, 2H), 1.31 (q, *J* = 7.5 Hz, 2H), 0.94 (t, *J* = 7.4 Hz, 3H). **^{13}C NMR** (101 MHz, Chloroform-*d*) δ 175.40, 125.55, 121.62, 113.27, 109.72, 49.73, 40.18, 33.31, 19.81, 13.60.

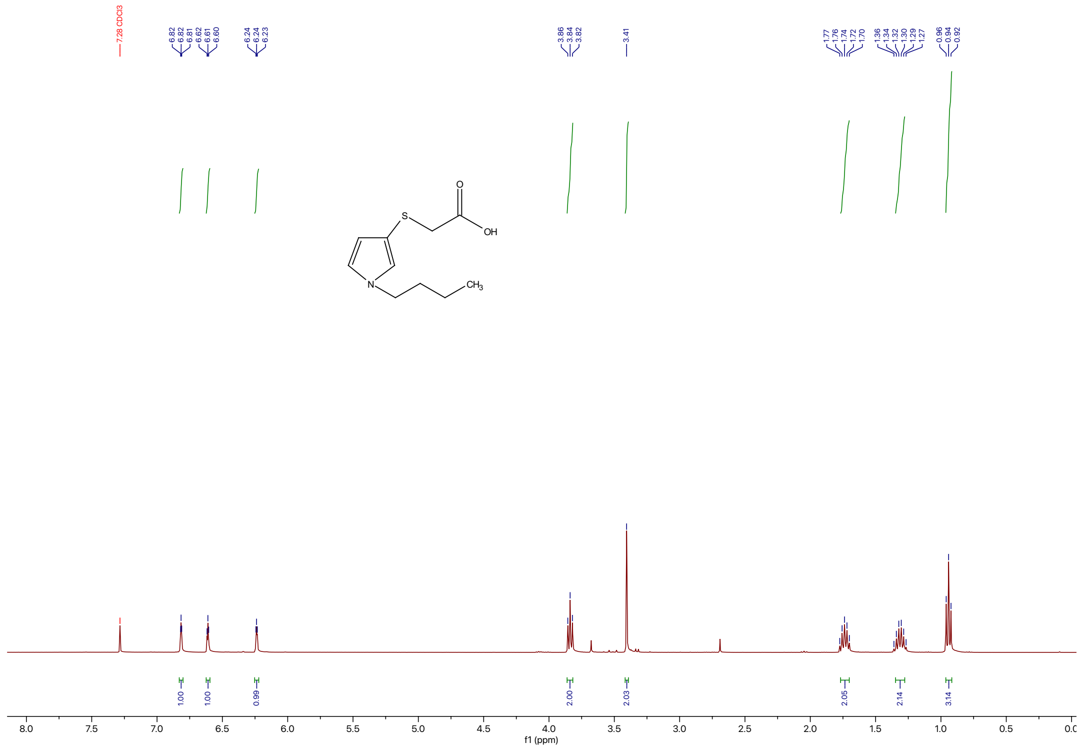

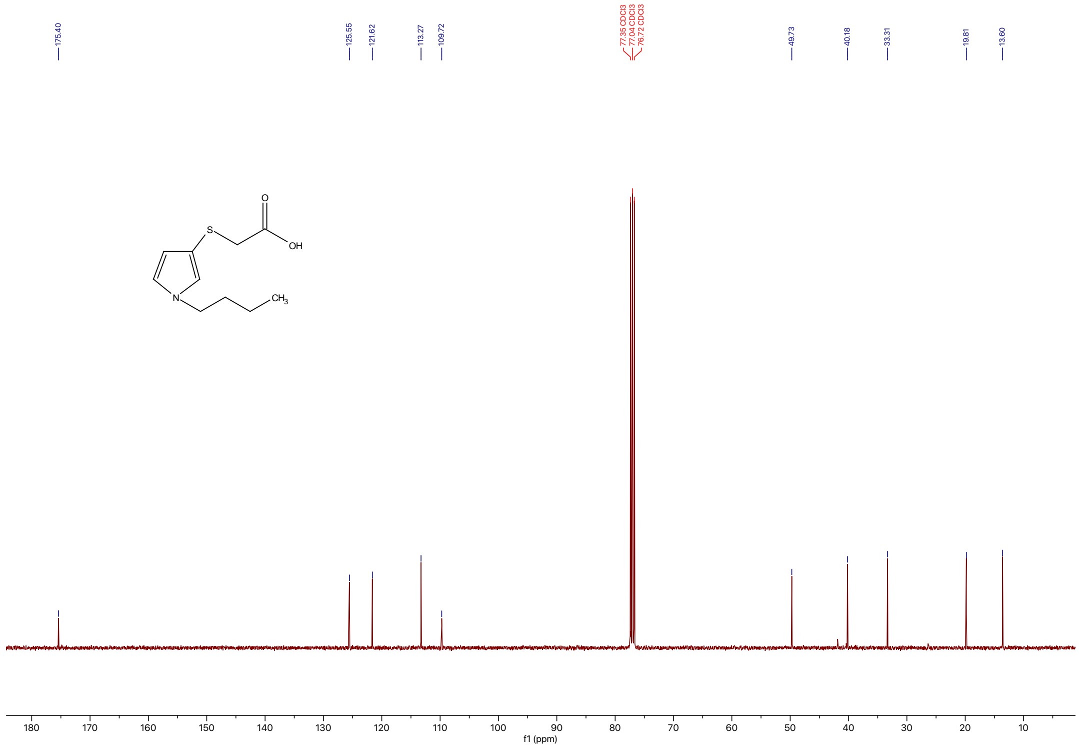

NMR data for Compound 2d

2-((1-butyl-1*H*-pyrrol-3-yl)thio)ethan-1-ol 2d: **^{1}H NMR** (400 MHz, Chloroform-*d*) δ 6.70 (t, *J* = 2.0 Hz, 1H), 6.56 (t, *J* = 2.5 Hz, 1H), 6.12 (dt, *J* = 2.7, 1.6 Hz, 1H), 3.77 (dd, *J* = 7.9, 6.4 Hz, 2H), 3.63 (t, *J* = 6.2 Hz, 2H), 3.02 (s, 1H), 2.72 (s, 1H), 1.74 – 1.63 (m, 2H), 1.29 – 1.20 (m, 1H), 0.89 (dd, *J* = 8.1, 6.6 Hz, 3H). **^{13}C NMR** (101 MHz, Chloroform-*d*) δ 125.21, 121.53, 113.39, 109.70, 60.32, 49.58, 40.02, 33.34, 19.79, 13.61.

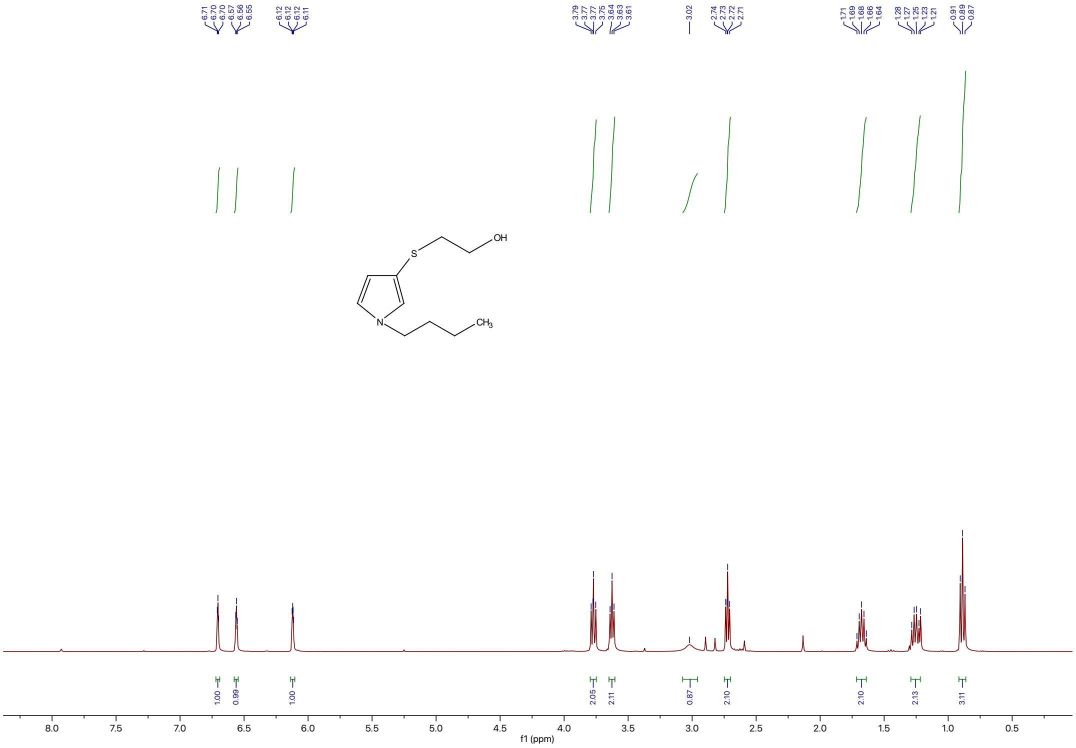

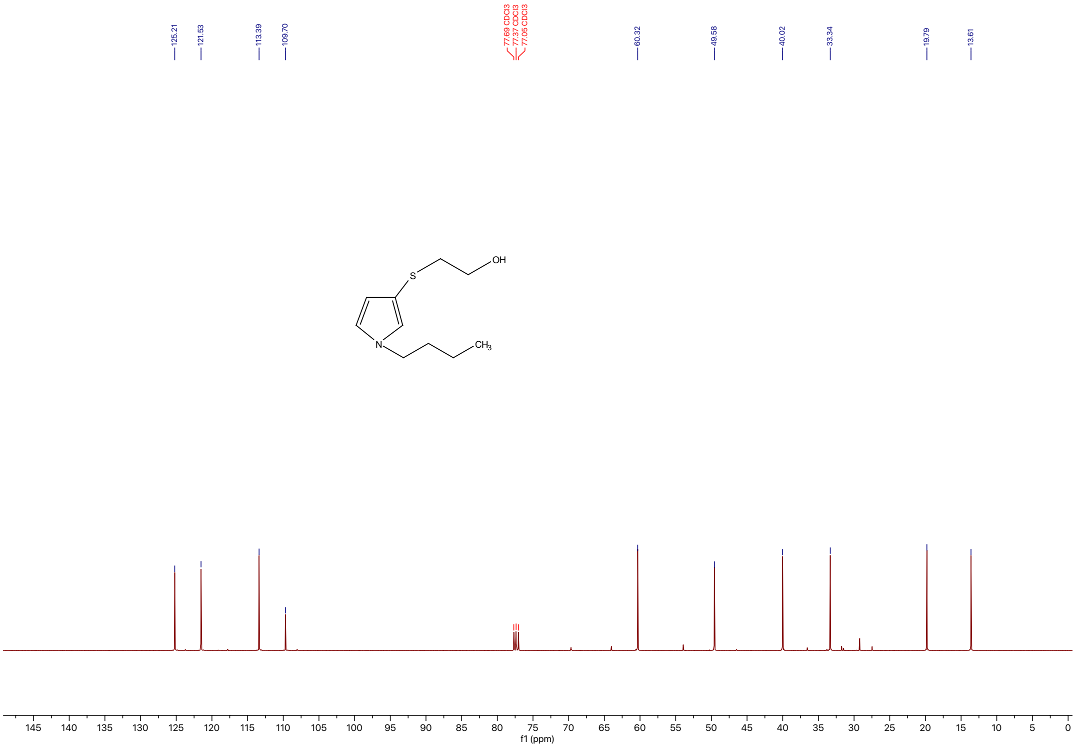

NMR data for Compound 2e

Methyl *N*-acetyl-*S*-(1-butyl-1*H*-pyrrol-3-yl)-*L*-cysteinate **2e**: **^{1}H NMR** (400 MHz, Chloroform-*d*) δ 6.75–6.70 (m, 1H), 6.59 (t, *J* = 2.6 Hz, 1H), 6.38 (d, *J* = 7.9 Hz, 1H), 6.16 (dd, *J* = 2.8, 1.7 Hz, 1H), 4.76 (ddd, *J* = 7.9, 5.7, 4.5 Hz, 1H), 3.80 (t, *J* = 7.2 Hz, 2H), 3.62 (s, 3H), 3.16–2.98 (m, 2H), 1.97 (s, 3H), 1.71 (dd, *J* = 8.7, 6.3 Hz, 2H), 1.34–1.27 (m, 2H), 0.95–0.89 (m, 3H). **^{13}C NMR** (101 MHz, Chloroform-*d*) δ 171.27, 169.85, 125.50, 121.60, 113.40, 109.72, 52.26 (d, *J* = 1.4 Hz), 49.66, 39.09, 33.37, 22.98, 19.82, 13.58.

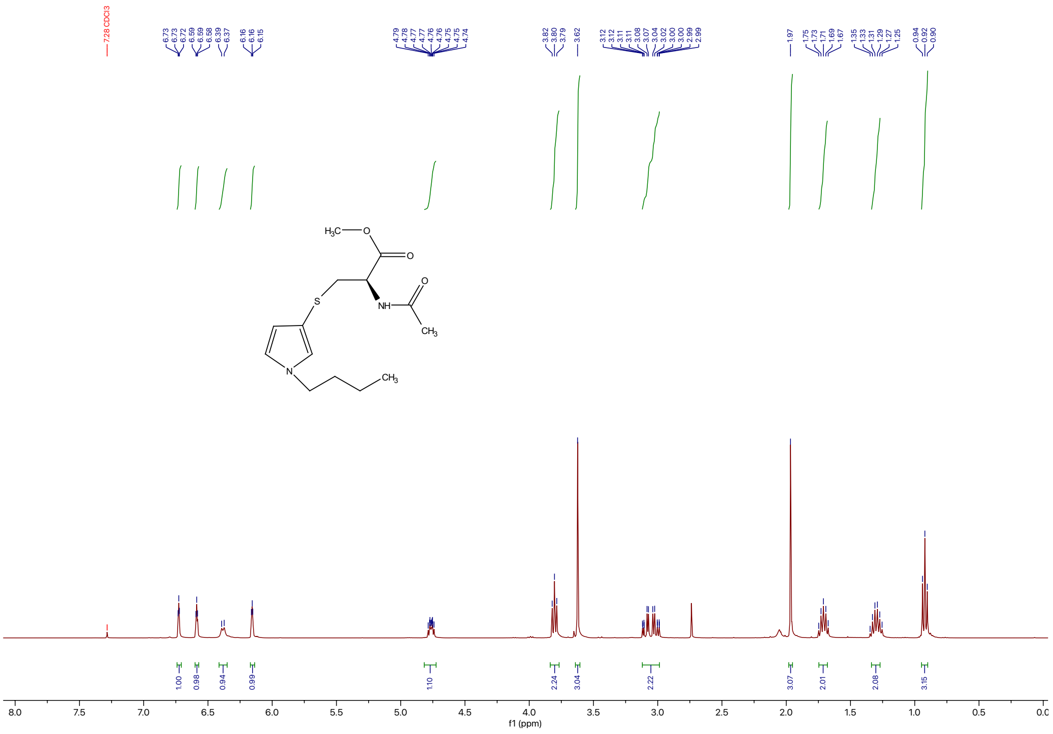
7.28 CDCl3
6.73
6.73
6.72
6.59
6.59
6.58
6.39
6.37
6.16
6.16
6.15
4.79
4.78
4.77
4.77
4.76
4.76
4.75
4.75
4.74
3.82
3.80
3.79
3.62
3.12
3.12
3.11
3.11
3.08
3.07
3.04
3.02
3.00
3.00
2.99
2.99
1.97
1.75
1.73
1.71
1.69
1.67
1.35
1.33
1.31
1.29
1.27
1.25
0.94
0.92
0.90
H3C
O
O
S
NH
O
CH3
N
CH3
1.00
0.98
0.94
0.99
1.10
2.24
3.04
2.22
3.07
2.01
2.08
3.15
f1 (ppm)

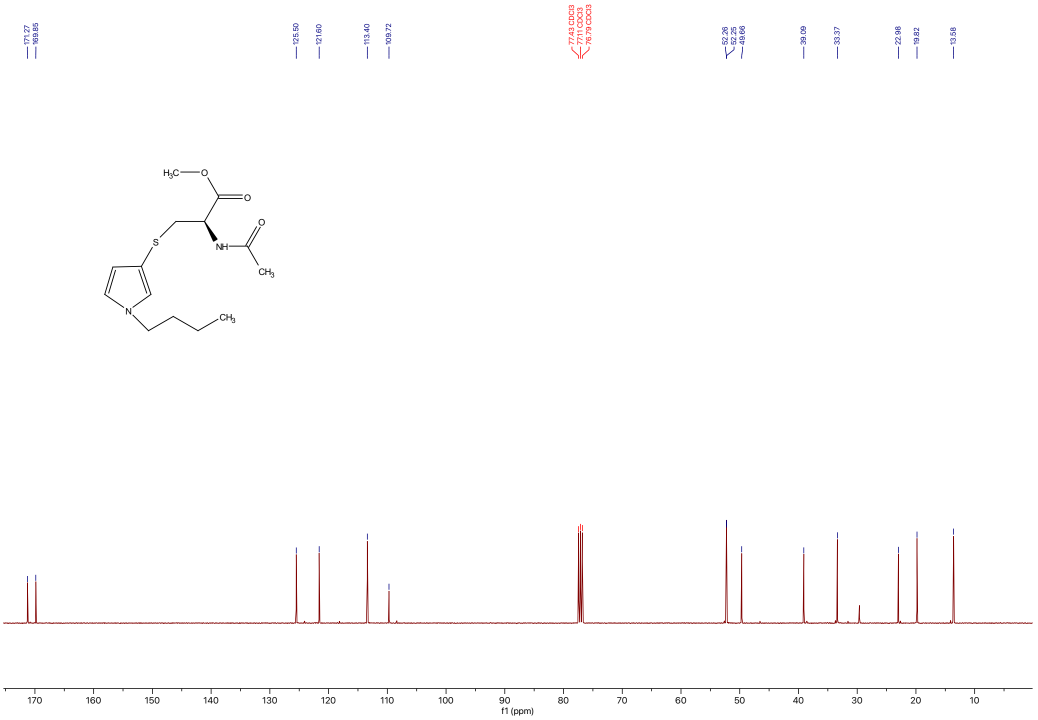
171.27
169.85
125.50
121.60
113.40
109.72
77.43 CDCl3
77.11 CDCl3
76.79 CDCl3
52.26
52.25
49.66
39.09
33.37
22.98
19.82
13.58
H3C
O
O
S
NH
O
CH3
N
CH3
f1 (ppm)

Amine Scope: Reaction of different amines with furan and 1-butanethiol (2f-2i)

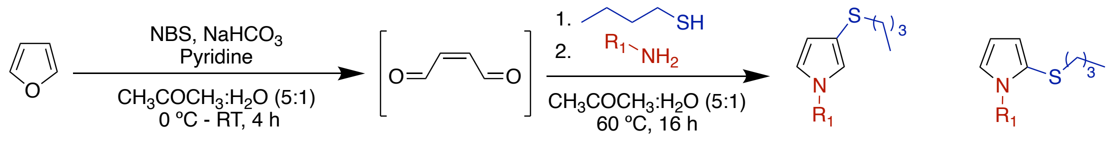

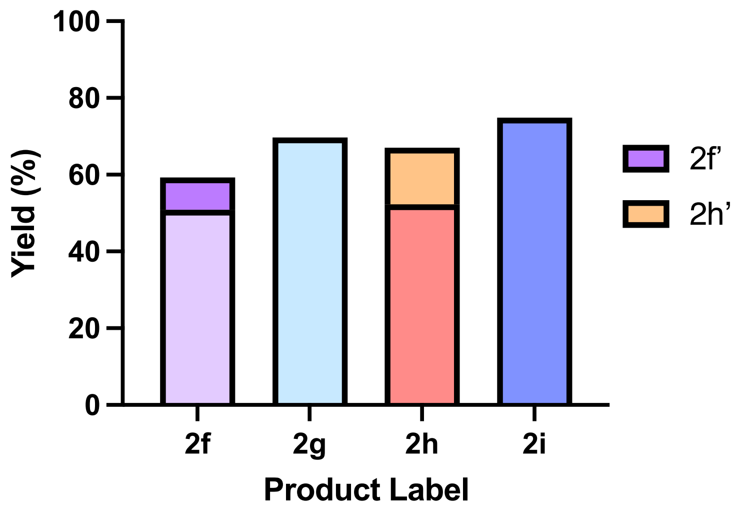

Label	Derivatives	Eluent (Hexanes: EA)	Characteristics	Yield (%)
2f	glycine	1:1	brown oil	53.9
2f'	glycine	1:1	brown oil	5.3
2g	Propargylamine	4:1	dark brown oil	69.7
2h	Ethanolamine	1:1	brown oil	52.3
2h'	Ethanolamine	3:2	dark green oil	14.7
2i	L-Phenylalanine methyl ester	3:7	light yellow oil	74.9

NMR data for Compound 2f and 2f'

2-(3-(butylthio)-1*H*-pyrrol-1-yl)acetic acid **2f**: ^{1}H NMR (400 MHz, Chloroform-*d*) δ 9.56 (s, 1H), 6.72 (t, *J* = 2.0 Hz, 1H), 6.63 (t, *J* = 2.6 Hz, 1H), 6.24 (dd, *J* = 2.8, 1.6 Hz, 1H), 4.62 (s, 2H), 2.71 – 2.64 (m, 2H), 1.58 (p, *J* = 7.2 Hz, 2H), 1.46 – 1.41 (m, 2H), 0.92 (t, *J* = 7.3 Hz, 3H). **^{13}C NMR** (101 MHz, Chloroform-*d*) δ 173.87, 125.43, 122.63, 114.12, 113.65, 50.51, 36.83, 31.72, 21.74, 13.74.

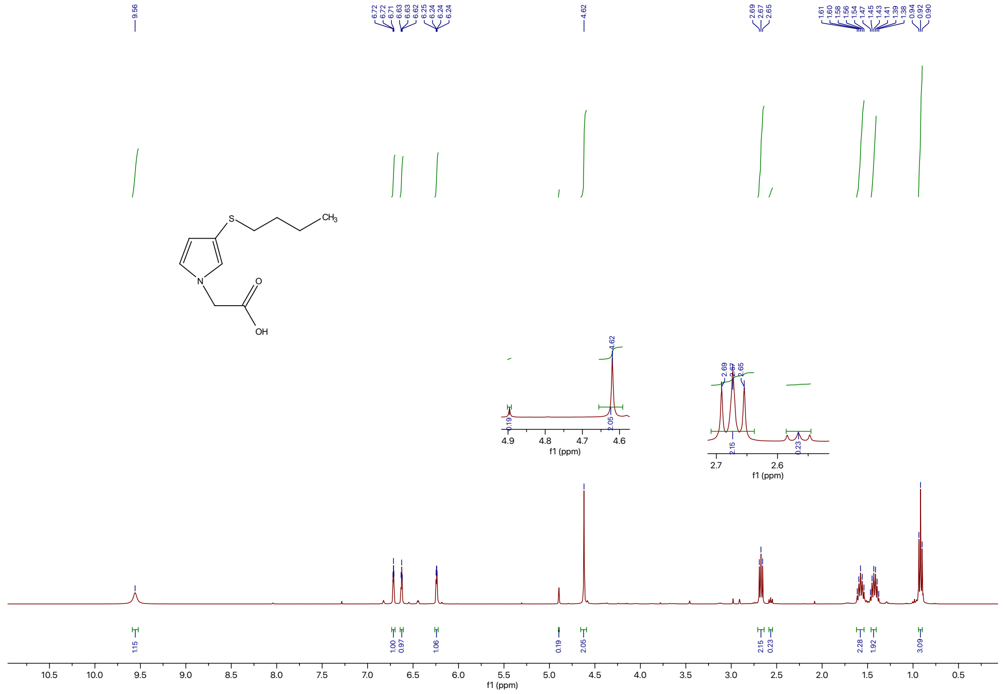

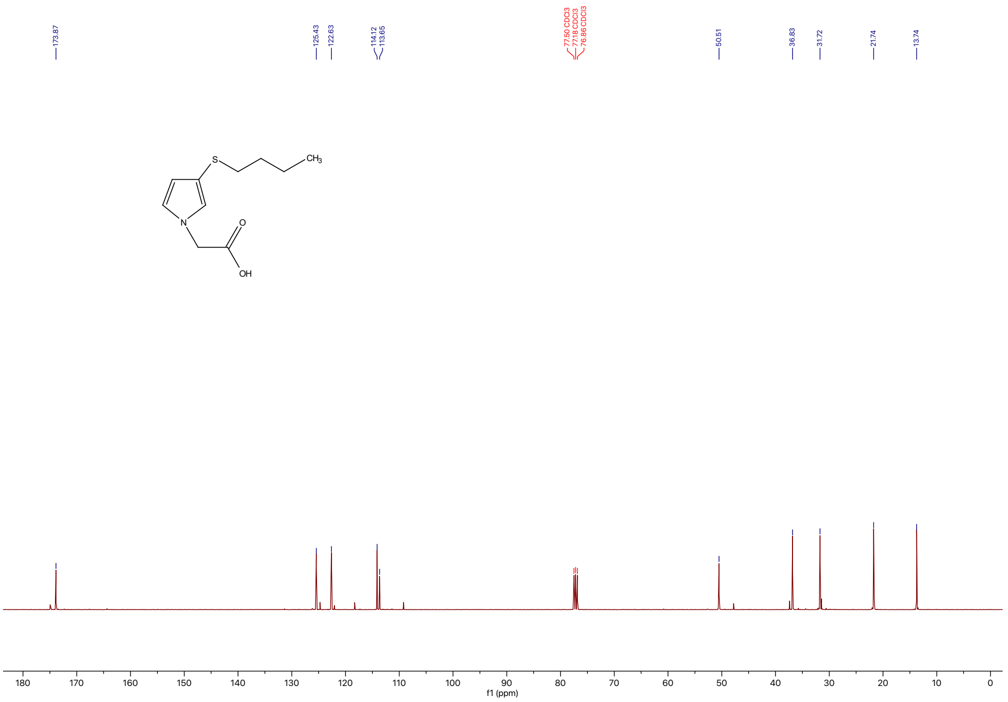

NMR data for Compound 2g

3-(butylthio)-1-(prop-2-yn-1-yl)-1*H*-pyrrole **2g**: **^{1}H NMR** (400 MHz, Chloroform-*d*) δ 6.83 (t, *J* = 2.0 Hz, 1H), 6.74 (t, *J* = 2.6 Hz, 1H), 6.23 (dd, *J* = 2.8, 1.7 Hz, 1H), 4.64 (d, *J* = 2.7 Hz, 2H), 2.69 – 2.65 (m, 2H), 2.47 (t, *J* = 2.6 Hz, 1H), 1.61 – 1.55 (m, 2H), 1.43 (ddd, *J* = 9.2, 7.3, 5.7 Hz, 2H), 0.91 (t, *J* = 7.3 Hz, 3H). **^{13}C NMR** (101 MHz, Chloroform-*d*) δ 124.10, 121.10, 114.01, 113.38, 77.62, 74.14, 38.82, 36.99, 31.79, 21.75, 13.75.

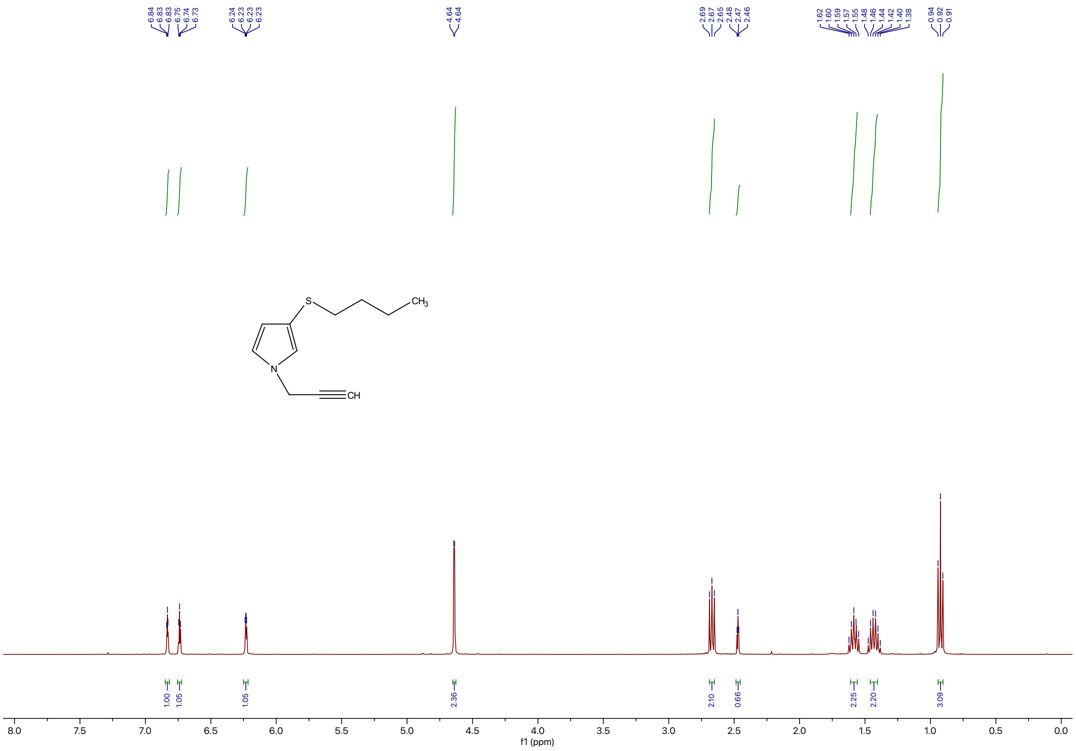

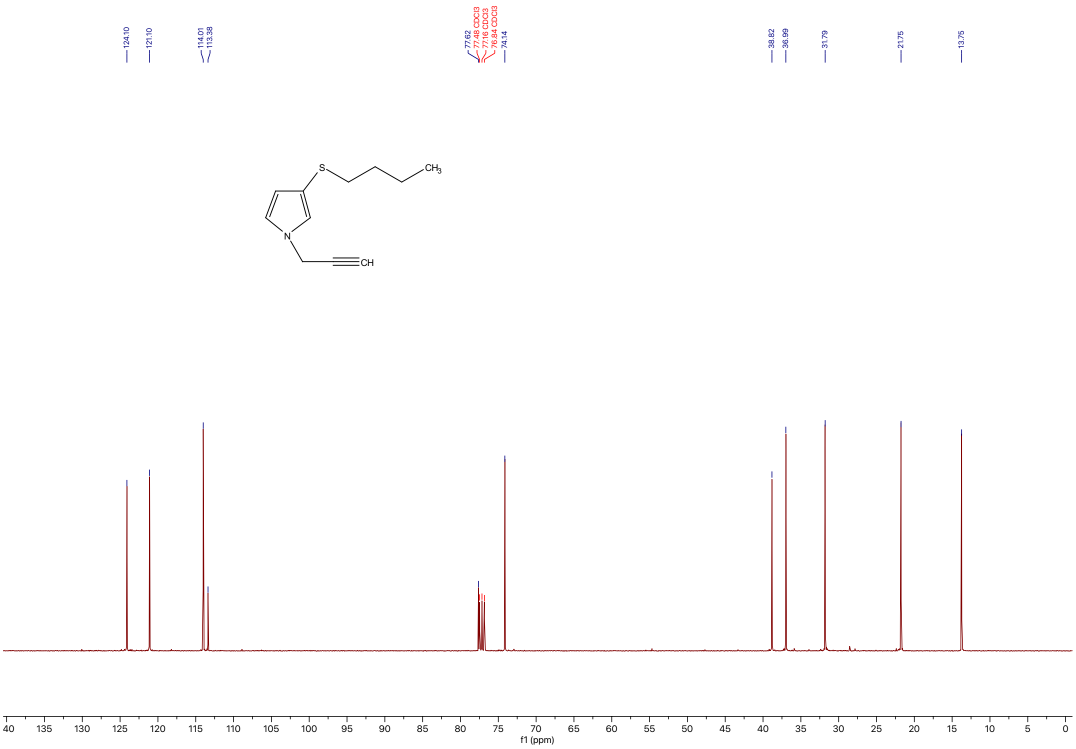

NMR data for Compound 2h

2-(3-(butylthio)-1*H*-pyrrol-1-yl)ethan-1-ol **2h**: ^{1}**H NMR** (400 MHz, Chloroform-*d*) δ 6.75 (d, *J* = 2.1 Hz, 1H), 6.66 (t, *J* = 2.5 Hz, 1H), 6.19 (dd, *J* = 2.8, 1.6 Hz, 1H), 3.95 (t, *J* = 5.2 Hz, 2H), 3.81 (t, *J* = 5.2 Hz, 2H), 2.69 – 2.60 (m, 2H), 1.59 – 1.52 (m, 2H), 1.41 (q, *J* = 7.4 Hz, 2H), 0.91 (t, *J* = 7.4 Hz, 3H). ^{13}**C NMR** (101 MHz, Chloroform-*d*) δ 124.60, 121.75, 113.55, 112.76, 62.63, 52.18, 37.03, 31.79, 21.74, 13.73.

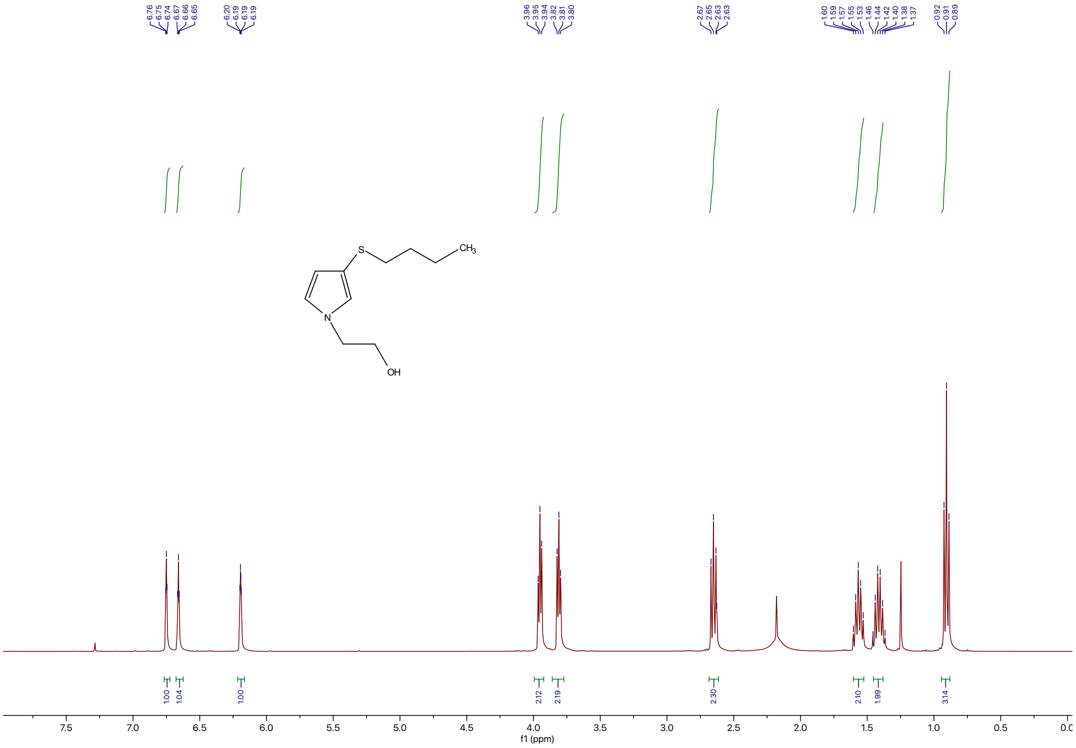

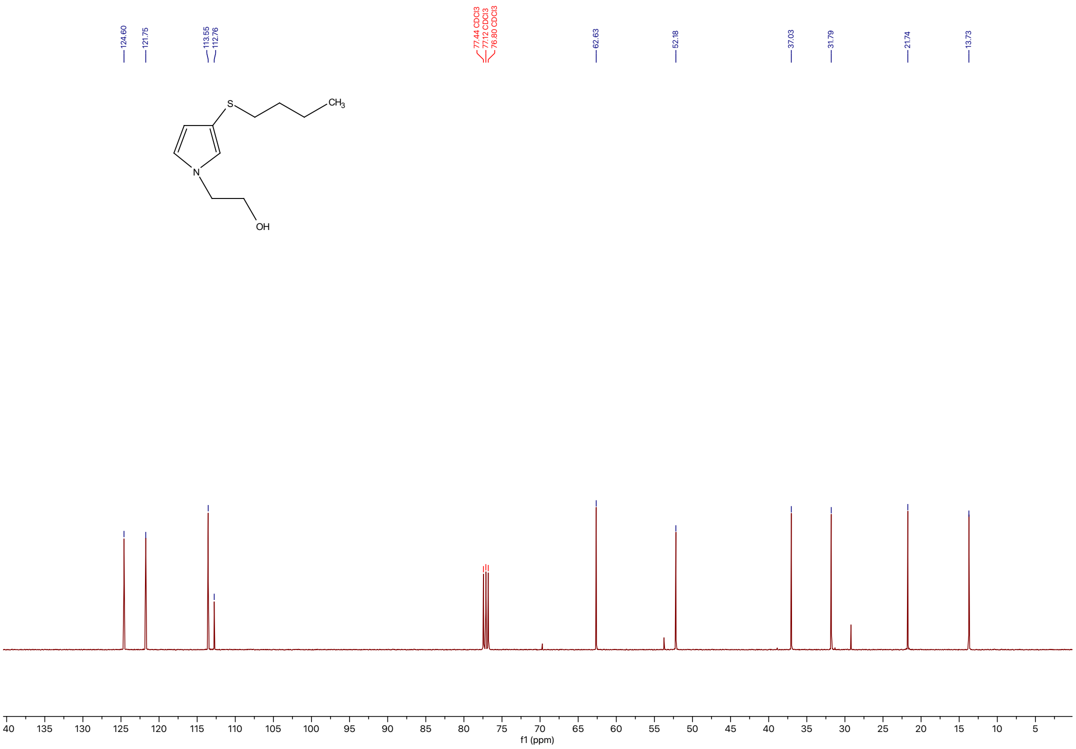

NMR data for Compound 2h'

2-(2-(butylthio)-1*H*-pyrrol-1-yl)ethan-1-ol **2h'**: **^{1}H NMR** (400 MHz, Chloroform-*d*) δ 6.90 (dd, *J* = 2.9, 1.8 Hz, 1H), 6.41 (dd, *J* = 3.6, 1.8 Hz, 1H), 6.19 (dd, *J* = 3.6, 2.8 Hz, 1H), 4.23 (t, *J* = 5.4 Hz, 2H), 3.89 (t, *J* = 5.4 Hz, 2H), 2.61 (dd, *J* = 7.9, 6.8 Hz, 2H), 1.57 – 1.52 (m, 2H), 1.44 – 1.39 (m, 2H), 0.92 (t, *J* = 7.3 Hz, 3H). **^{13}C NMR** (101 MHz, Chloroform-*d*) δ 124.06, 121.26, 117.89, 108.42, 63.02, 49.06, 37.62, 31.47, 21.69, 13.68.

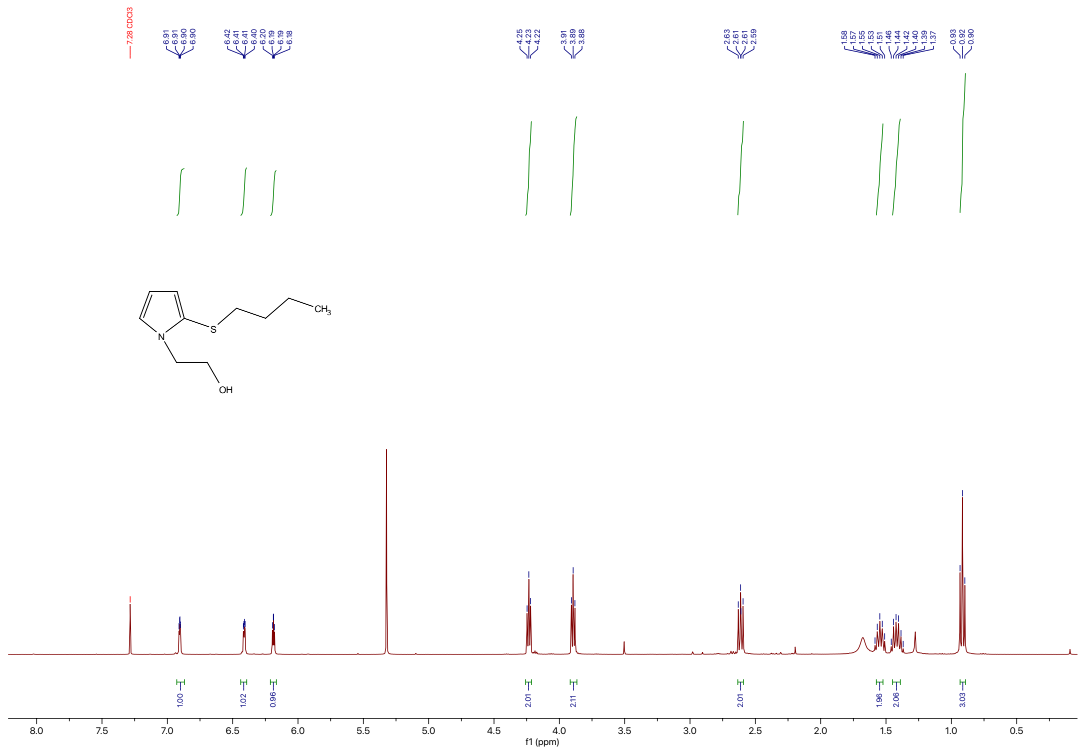

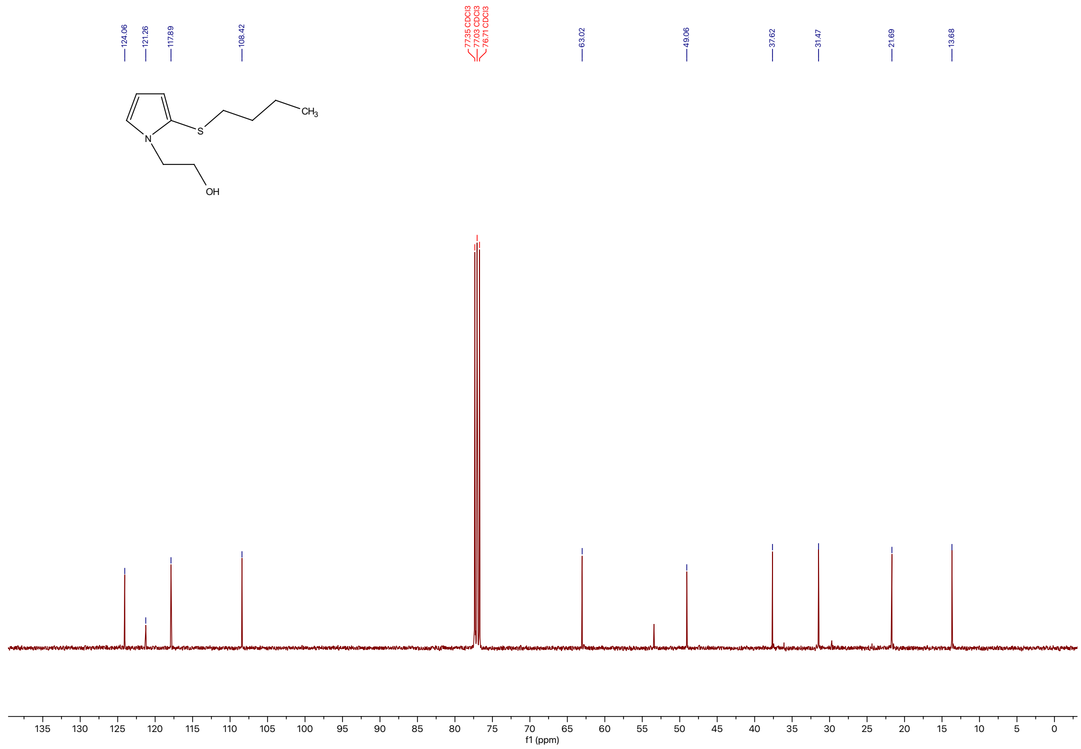

Proposed mechanism for the formation of compound 2h’

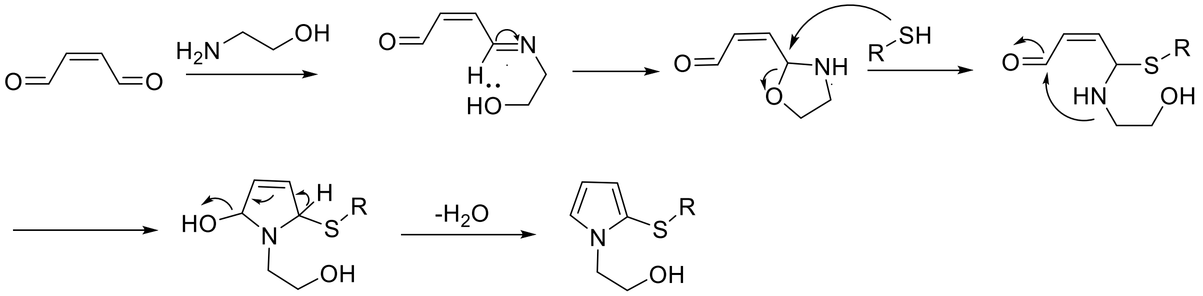

NMR data for Compound 2i

Methyl (*S*)-2-(3-(butylthio)-1*H*-pyrrol-1-yl)-3-phenylpropanoate **2i**: ^{1}**H NMR** (400 MHz, Chloroform-*d*) δ 7.24 (tdd, *J* = 4.8, 3.6, 1.5 Hz, 3H), 7.05 – 7.00 (m, 2H), 6.77 (t, *J* = 2.0 Hz, 1H), 6.68 (t, *J* = 2.6 Hz, 1H), 6.19 (dd, *J* = 2.9, 1.7 Hz, 1H), 4.70 (dd, *J* = 9.3, 6.0 Hz, 1H), 3.74 (s, 3H), 3.41 (dd, *J* = 13.9, 6.0 Hz, 1H), 3.25 (dd, *J* = 13.8, 9.3 Hz, 1H), 2.63 (dd, *J* = 7.9, 6.7 Hz, 2H), 1.57 – 1.50 (m, 2H), 1.45 – 1.38 (m, 2H), 0.93 (t, *J* = 7.3 Hz, 3H). ^{13}**C NMR** (101 MHz, Chloroform-*d*) δ 170.26, 136.07, 128.82, 128.60, 127.14, 123.97, 120.89, 113.80, 113.10, 63.80, 52.66, 39.52, 36.84, 31.68, 21.72, 13.76.

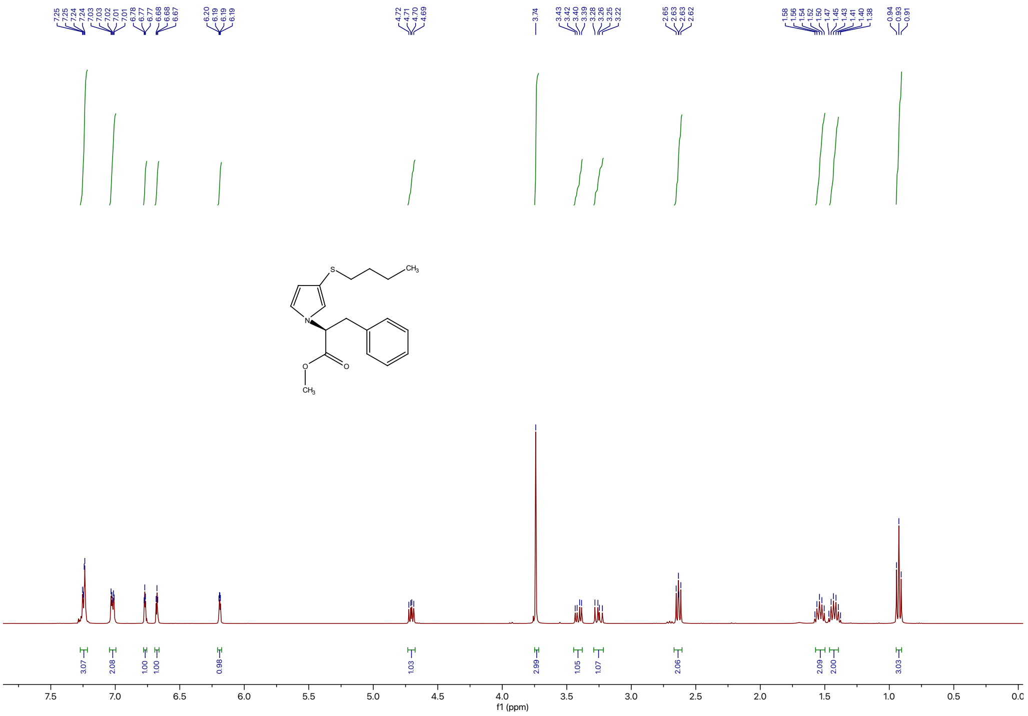

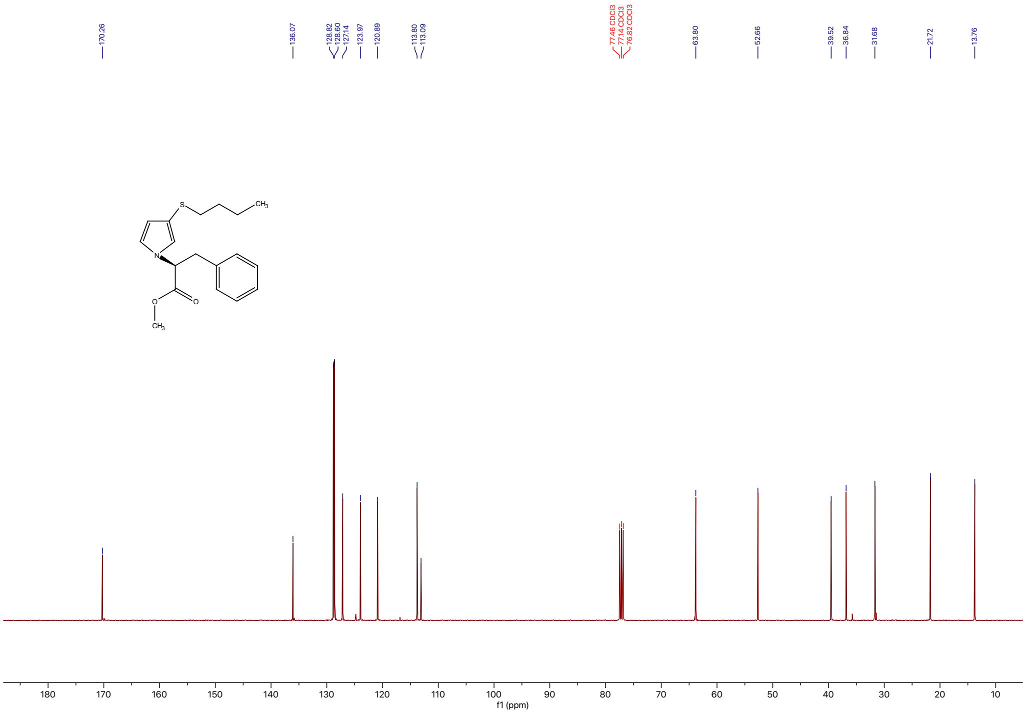

Substrate Scope: Reaction with varying furan, thiol, amines (2j-2m)

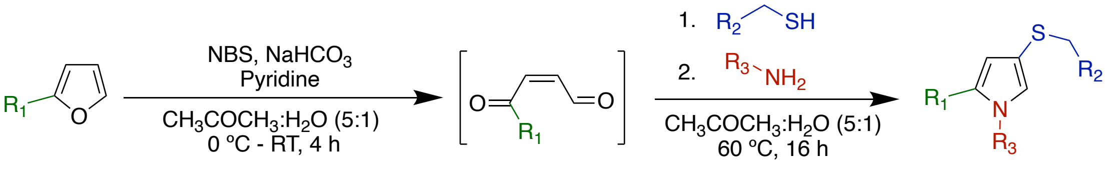

2j,2k,2l,2m

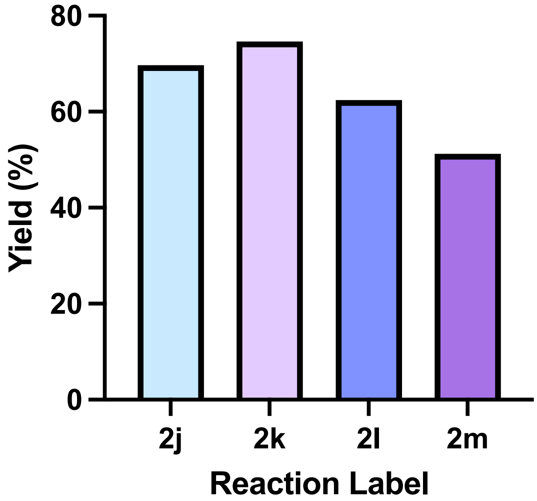

Label	R_1	R_2	R_3	Eluent (Hexanes: EA)	Characteristics	Yield (%)
2j	H	1-butanethiol	aniline	9:1	dark yellow oil	69.7
2k	H	N-Acetyl-L-cysteine methyl ester	N-alpha-Acetyl-L-lysine	1:4	bright yellow oil	74.6
2l	CH_3	1-butanethiol	1-butylamine	9:1	dark brown oil	62.4
2m	H	1-butanethiol	m-xylenediamine	4:1	brown oil	51.2

NMR data for Compound 2j

3-(butylthio)-1-phenyl-1*H*-pyrrole **2j**: **^{1}H NMR** (400 MHz, Chloroform-*d*) δ 7.47–7.43 (m, 2H), 7.41–7.37 (m, 2H), 7.31–7.27 (t, 1H), 7.17–7.15 (t, 1H), 7.09–7.06 (m, 1H), 6.40 (dd, J = 2.9, 1.7 Hz, 1H), 2.77–2.72 (t, 2H), 1.67–1.60 (m, 2H), 1.50–1.42 (m, 2H), 0.94 (t, J = 7.3 Hz, 3H). **^{13}C NMR** (101 MHz, Chloroform-*d*) δ 140.24, 129.64, 125.92, 122.54, 120.32, 119.96, 115.42, 115.10, 36.73, 31.82, 21.79, 13.74.

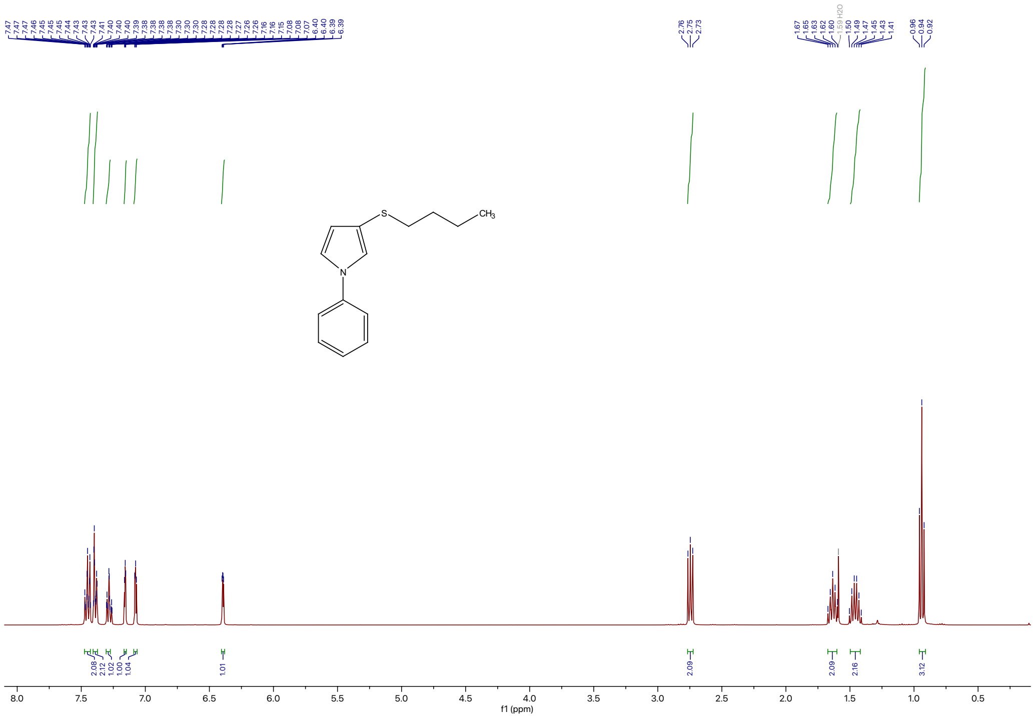

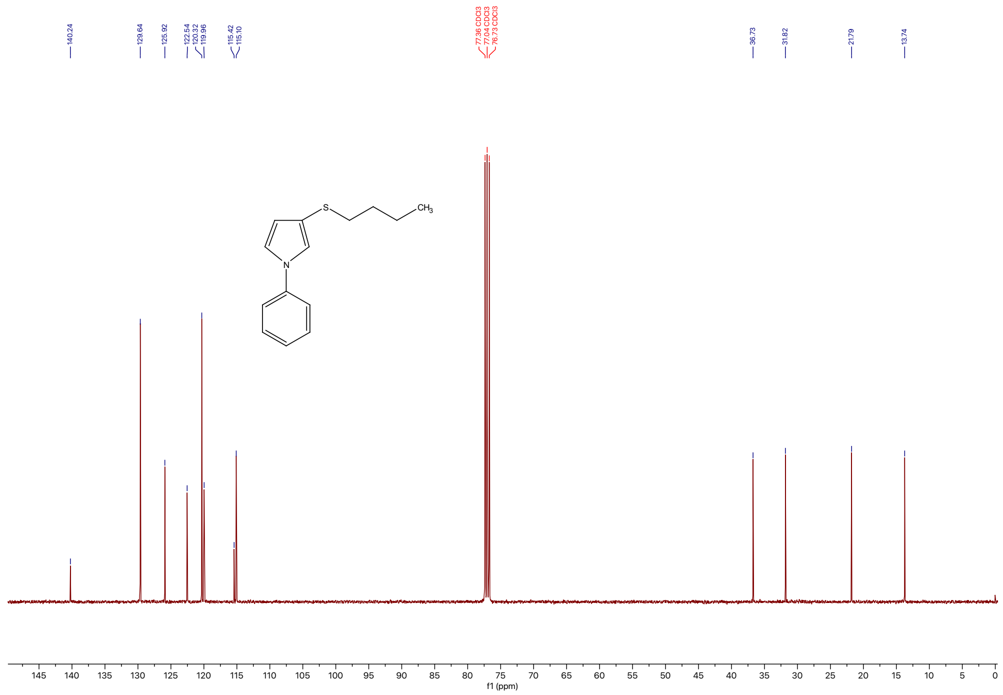

NMR data for Compound 2k

(*R*)-2-acetamido-6-(3-(((*R*)-2-acetamido-3-methoxy-3-oxopropyl)thio)-1*H*-pyrrol-1-yl)hexanoic acid **2k**: ^{1}**H NMR** (400 MHz, Chloroform-*d*) δ 6.74 (t, *J* = 2.0 Hz, 1H), 6.63 (d, *J* = 7.4 Hz, 1H), 6.59 (t, *J* = 2.5 Hz, 1H), 6.56 (d, *J* = 7.8 Hz, 1H), 6.18 (dd, *J* = 2.8, 1.7 Hz, 1H), 4.68 – 4.63 (m, 1H), 4.58 (q, *J* = 6.0 Hz, 1H), 3.84 (ddd, *J* = 26.7, 13.9, 7.3 Hz, 2H), 3.72 (s, 3H), 3.10 (dd, *J* = 14.0, 5.2 Hz, 1H), 2.91 (dd, *J* = 14.0, 6.9 Hz, 1H), 2.05 (s, 3H), 2.03 (s, 3H), 1.89 – 1.72 (m, 4H), 1.38 – 1.24 (m, 2H). ^{13}**C NMR** (101 MHz, Chloroform-*d*) δ 173.89, 171.50, 171.34, 170.63, 125.95, 121.64, 113.72, 109.30, 65.87, 52.62, 52.19, 49.41, 38.88, 31.44, 30.63, 23.03, 22.89, 21.78.

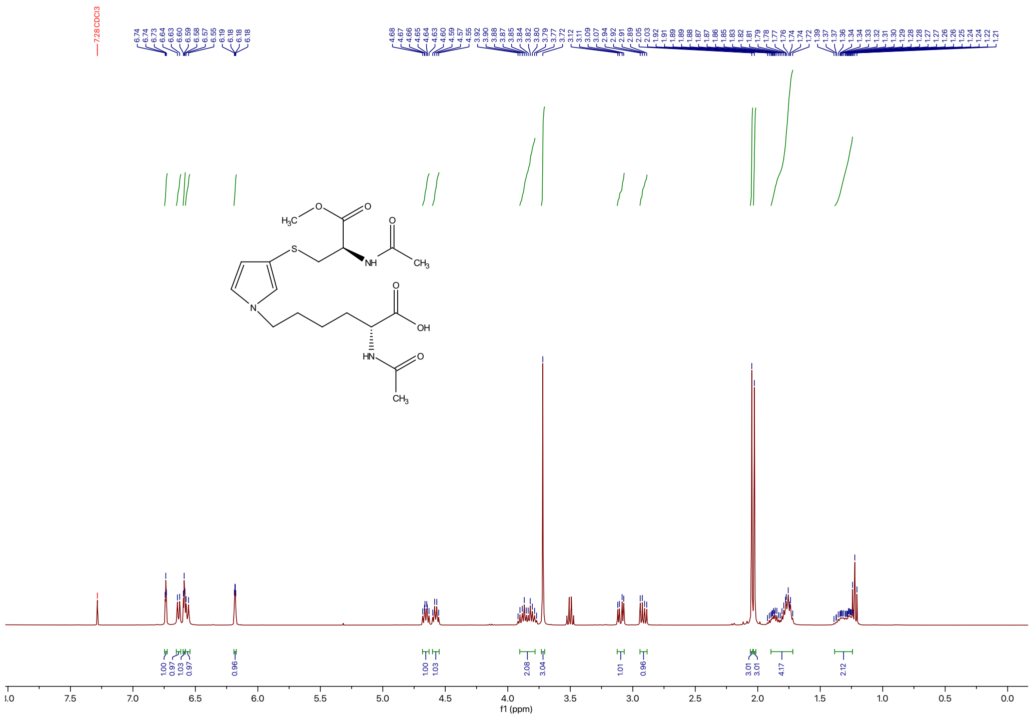

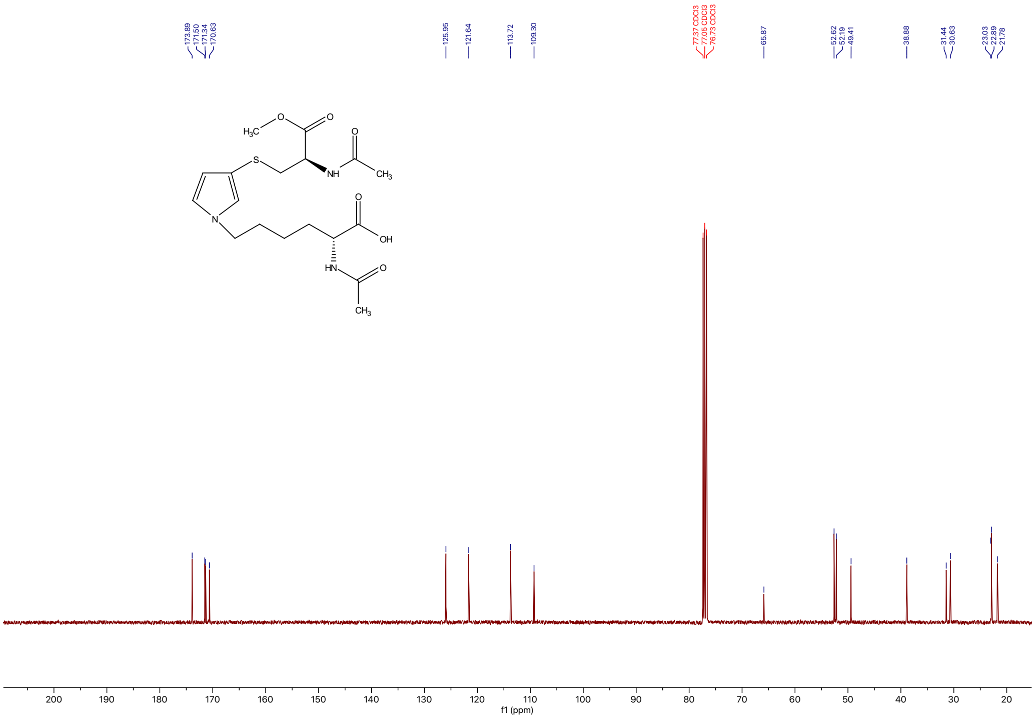

NMR data for Compound 2l

1-butyl-4-(butylthio)-2-methyl-1*H*-pyrrole **2l**: ^{1}**H NMR** (400 MHz, Chloroform-*d*) δ 6.57 (d, *J* = 2.9 Hz, 1H), 6.18 (d, *J* = 2.8 Hz, 1H), 3.81 (t, *J* = 7.3 Hz, 2H), 2.62 – 2.57 (m, 2H), 2.31 (s, 3H), 1.73 – 1.68 (m, 2H), 1.56 – 1.50 (m, 2H), 1.46 – 1.42 (m, 2H), 1.36 (q, *J* = 7.4, 6.2 Hz, 2H), 0.98 – 0.91 (m, 6H). ^{13}**C NMR** (101 MHz, Chloroform-*d*) δ 132.06, 119.29, 112.67, 108.77, 47.34, 37.22, 33.22, 31.77, 21.79, 19.94, 13.77 (d, *J* = 3.3 Hz), 10.06.

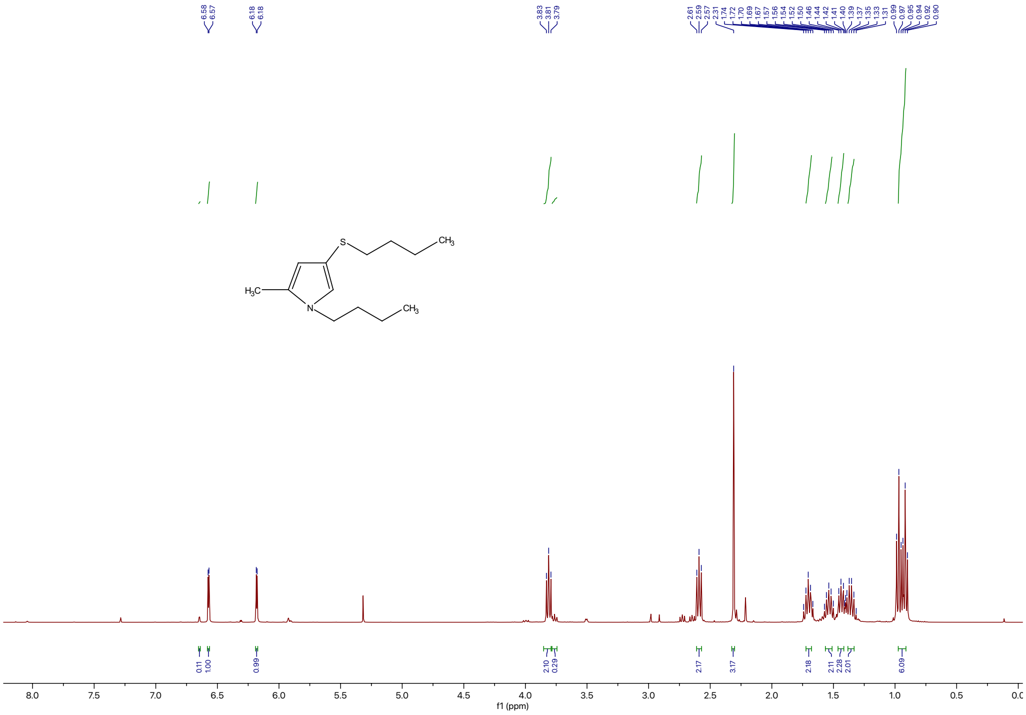

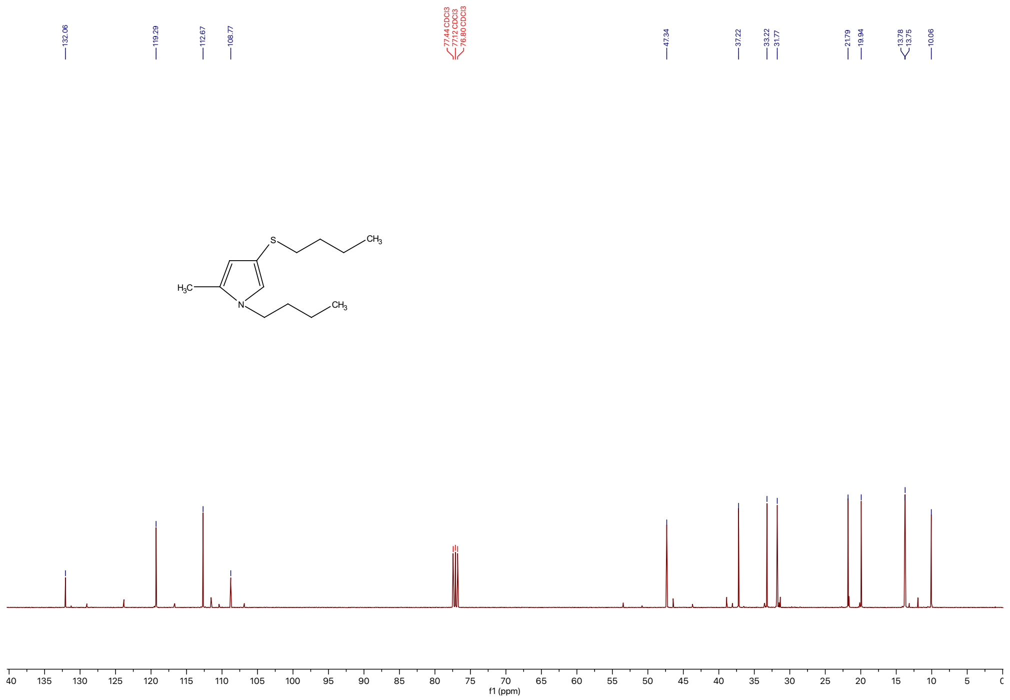

NMR data for Compound 2m

1,3-bis((3-(butylthio)-1*H*-pyrrol-1-yl)methyl)benzene **2m**: **^{1}H NMR** (400 MHz, Chloroform-*d*) δ 7.33 – 7.28 (m, 1H), 7.03 (dd, *J* = 7.7, 1.8 Hz, 2H), 6.91 (d, *J* = 2.1 Hz, 1H), 6.73 (t, *J* = 2.0 Hz, 2H), 6.64 (t, *J* = 2.5 Hz, 2H), 6.24 (t, *J* = 2.3 Hz, 2H), 5.00 (s, 4H), 2.70 – 2.65 (m, 4H), 1.61 – 1.56 (m, 4H), 1.47 – 1.40 (m, 4H), 0.92 (t, *J* = 7.3 Hz, 6H). **^{13}C NMR** (101 MHz, Chloroform-*d*) δ 138.29, 129.35, 126.53, 125.69, 124.85, 121.84, 113.80, 113.02, 53.36, 37.01, 31.77, 21.73, 13.76.

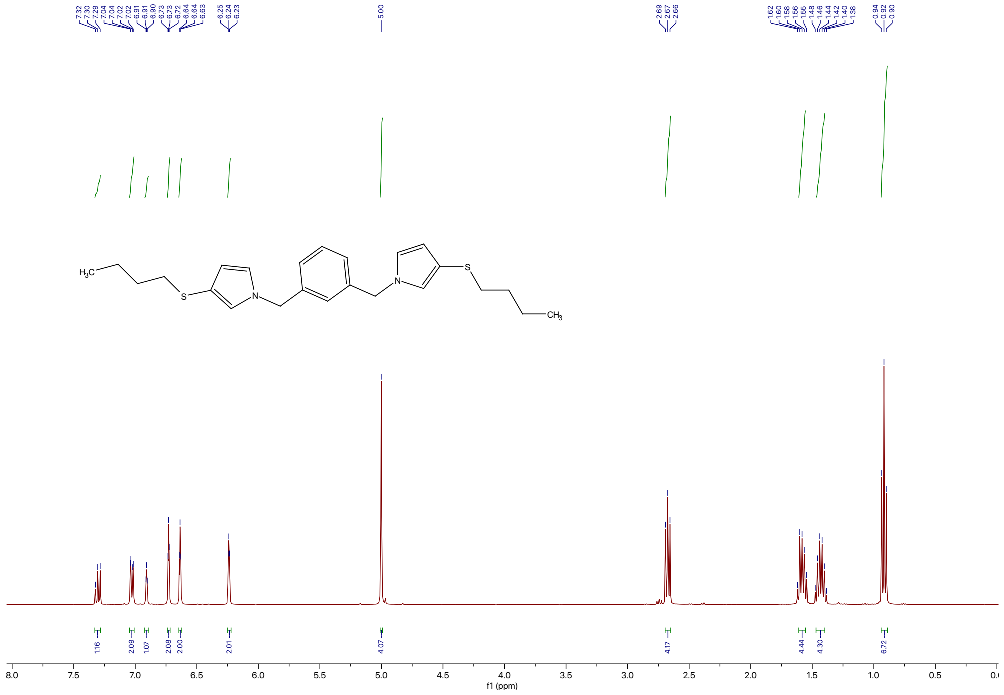

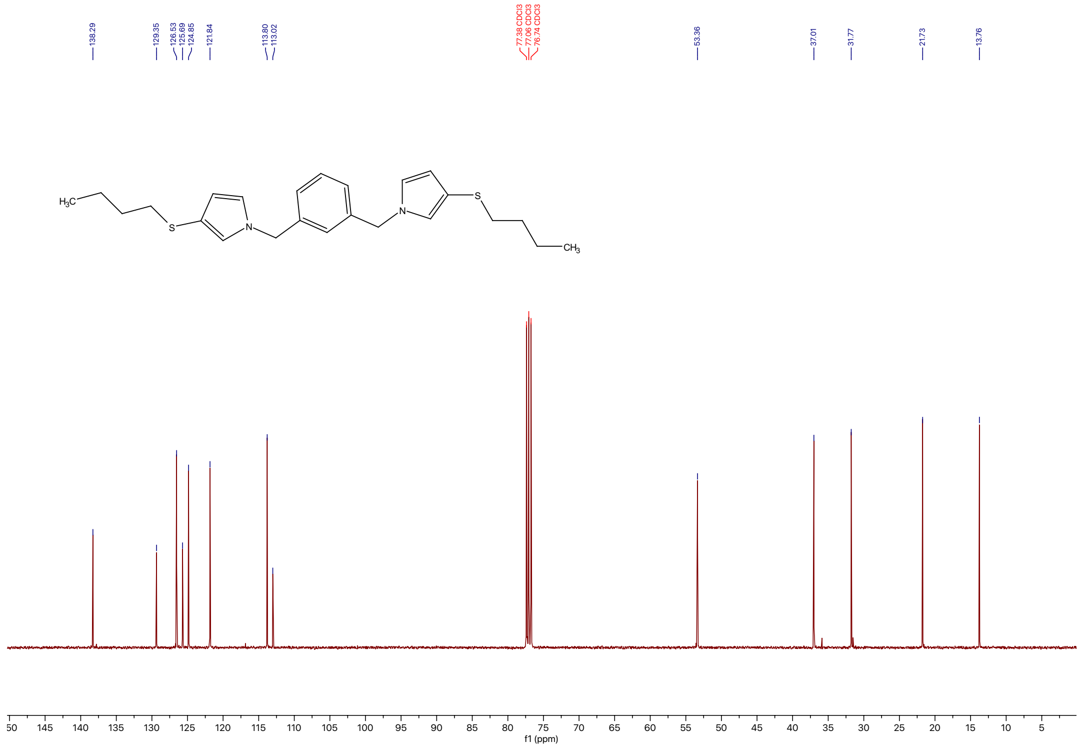

X. Supplementary Fig. 5. Late-stage modification of 3-thio *N*-pyrrole

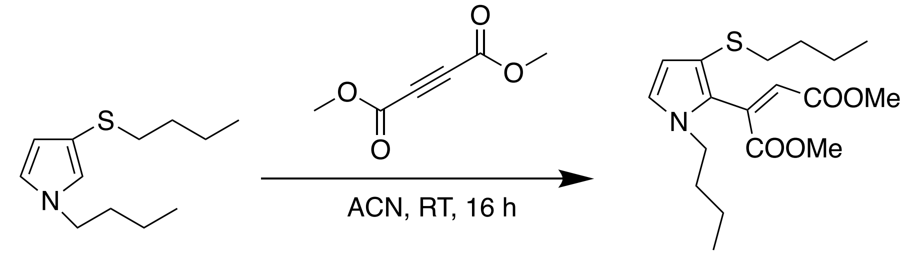

2a **5a**

1-butyl-3-(butylthio)-1*H*-pyrrole (0.47 mmol, 1 equiv.) was dissolved in 10 mL of acetonitrile and added to a reaction mixture of dimethyl acetylenedicarboxylate (1.5 equiv.) in acetonitrile (10 mL) and the reaction mixture was stirred for 16 h at RT. TLC was used to monitor the reaction progression. The volatiles were removed under reduced pressure and the crude product was purified by column chromatography using a mixture of hexanes and ethyl acetate (7:3) as the eluent to get product **5a**. **5a** was obtained in 49.7 % yield as a bright yellow oil. **5a** was characterized by ^{1}H and ^{13}C NMR.

NMR data for Compound 5a

Dimethyl 2-(1-butyl-3-(butylthio)-1*H*-pyrrol-2-yl)maleate **5a**: ^{1}**H NMR** (400 MHz, Chloroform-*d*) δ 6.75 (d, *J* = 2.9 Hz, 1H), 6.27 (d, *J* = 2.8 Hz, 1H), 6.12 (s, 1H), 3.86 (s, 3H), 3.83 (d, *J* = 7.5 Hz, 2H), 3.80 (s, 3H), 2.71 – 2.66 (m, 2H), 1.67 (t, *J* = 7.5 Hz, 2H), 1.56 – 1.49 (m, 2H), 1.42 – 1.37 (m, 2H), 1.29 – 1.22 (m, 2H), 0.93 – 0.86 (m, 6H). ^{13}**C NMR** (101 MHz, Chloroform-*d*) δ 167.29, 165.43, 137.03, 128.41, 124.86, 123.30, 118.29, 113.42, 52.67, 52.00, 48.58, 36.58, 33.22, 31.39, 21.79, 19.79, 13.66, 13.61.

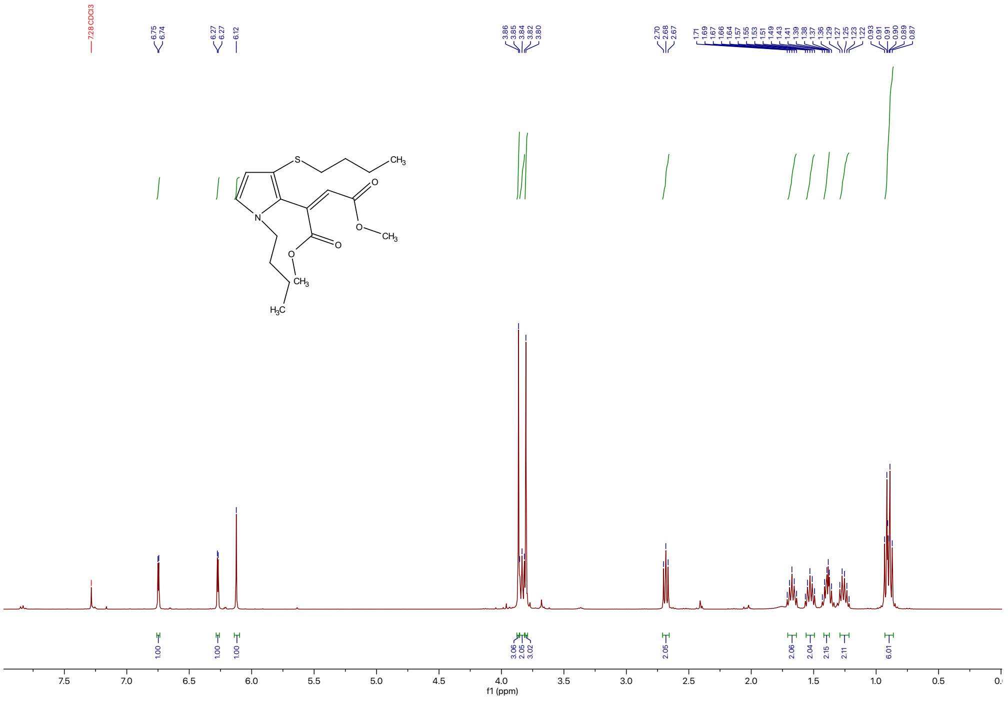

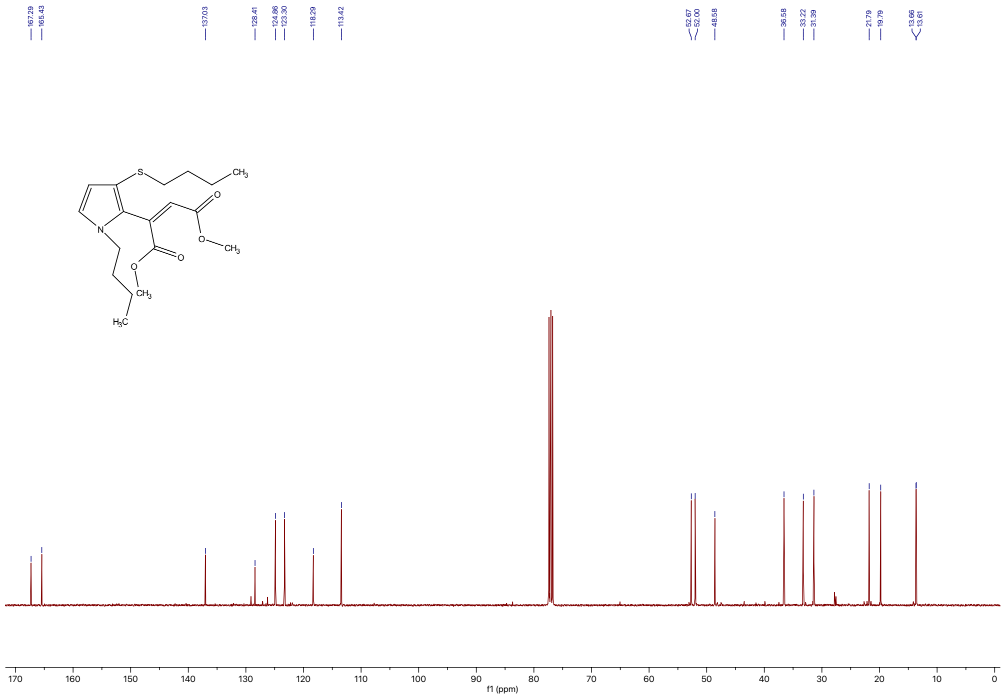

XI. Supplementary Fig. 6. Photophysical properties of 3-thio *N*-pyrrole.

5 mM solutions of 3-thio *N*-pyrrole, furan, 1-butanethiol, 1-butylamine were made in cuvette separately using DMSO as the solvent. A scan was run from 270 nm to 800 nm to determine the UV absorbance of 3-thio *N*-pyrrole using Agilent Cary UV-Vis Compact.

Compound	Amount	Total volume	Concentration
furan	0.7 μL	2 mL	5 mM
1-butanethiol	0.9 μL	2 mL	5 mM
1-butylamine	1.0 μL	2 mL	5 mM
3-thio *N*-pyrrole	2.1 mg	2 mL	5 mM

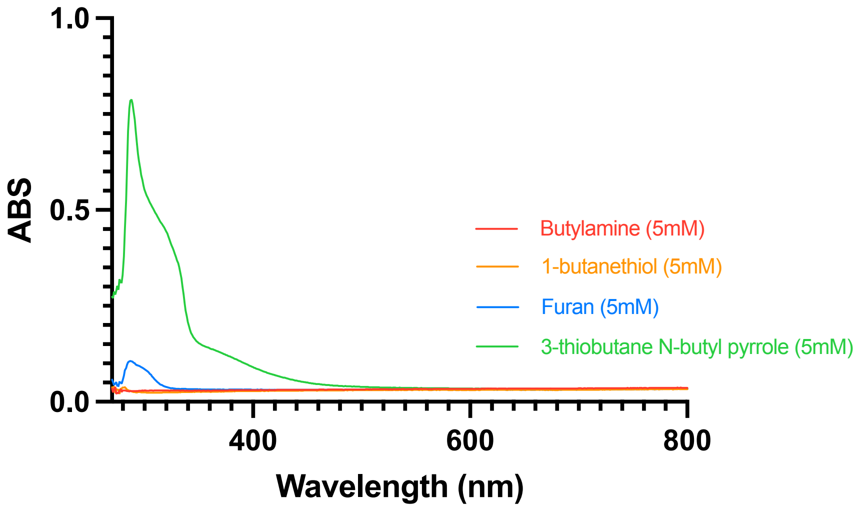

XII. Supplementary Fig. 7. Procedure for the modification of linear peptide **1n** containing lysine to generate 3-thio *N*-pyrrole product **2n**

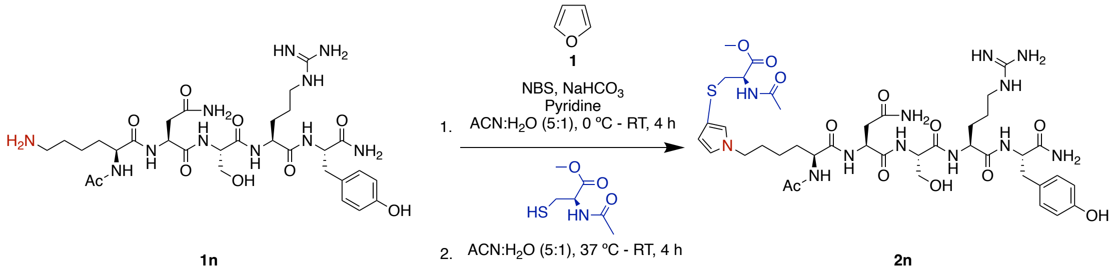

Furan **1** (100 µL, 1.38 mmol, 1 equiv.) and sodium bicarbonate (115 mg, 1.38 mmol, 1 equiv.) were added to a solution of 12 mL ACN:H_2O (5:1). The reaction mixture was cooled to 0 °C and left to stir for 15 min. N-Bromosuccinimide NBS (244 mg, 1.38 mmol, 1 equiv.) was dissolved in a solution of 12 mL ACN:H_2O (5:1) and added to the reaction mixture dropwise. Afterward, the reaction mixture was left to stir for 10 min, and pyridine (222 µL, 2.76 mmol, 2 equiv.) was added to the reaction mixture. The reaction mixture was stirred for 4 h and used without further purification. From the pot, 1.5 equiv. (184 µL) of the reaction solution was taken and incubated with N-acetyl-cysteine methyl ester (7.4 mg, 6 equiv.) at 37 °C for 30 min in 5 mL of acetonitrile and 1 mL of water. 5 mg of Ac-KNSRY (**1n**, 1 equiv.) was dissolved in 5 mL of acetonitrile and 1 mL of water and was then added to the reaction mixture. The concentration of peptide in the reaction mixture was 589 nM. The reaction mixture was left to stir for 4 h at room temperature. Samples were injected directly into LC-MS to analyze the peptide modification. The reaction mixture was analyzed by HPLC using method A to determine the percent conversion to modified product **2n** (87.9 %).

Ac-KNSRY linear peptide 1n. LCMS *m/z* 708.3749 (calcd. $[M{+}H^+]$ = 708.37), *m/z* 354.6907 (calcd. $[(M{+}2H^+)/2]$ = 354.685), Purity: > 95 % (HPLC analysis at 220 nm). Retention time in HPLC: 3.502 min.

Ac-KNSRY modified peptide 2n. LCMS *m/z* 933.4193 (calcd. $[M{+}H^+]$ = 933.42), *m/z* 955.4009 (calcd. $[M{+}Na^+]$ = 955.42), *m/z* 467.2131 (calcd. $[(M{+}2H^+)/2]$ = 467.21), Purity: > 95 % (HPLC analysis at 220 nm). Retention time in HPLC: 9.511 min.

HPLC trace of 1n

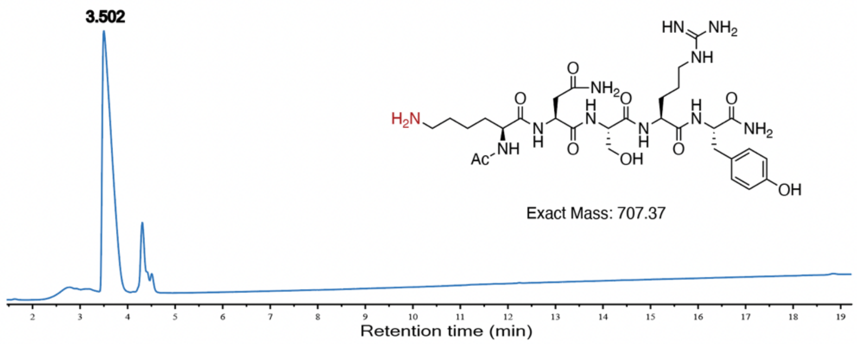

HPLC trace of reaction mixture to generate 2n

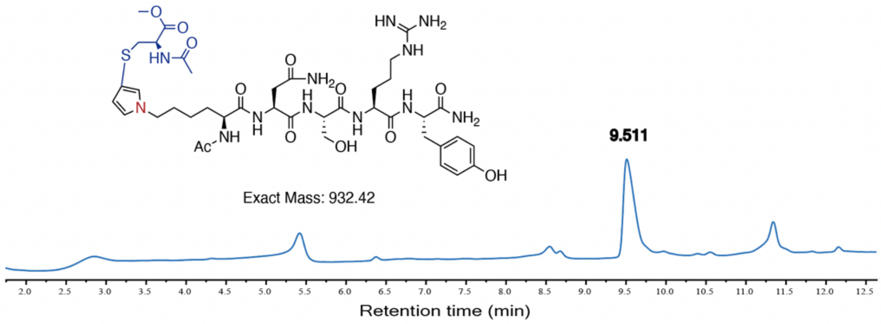

MS-trace 1n

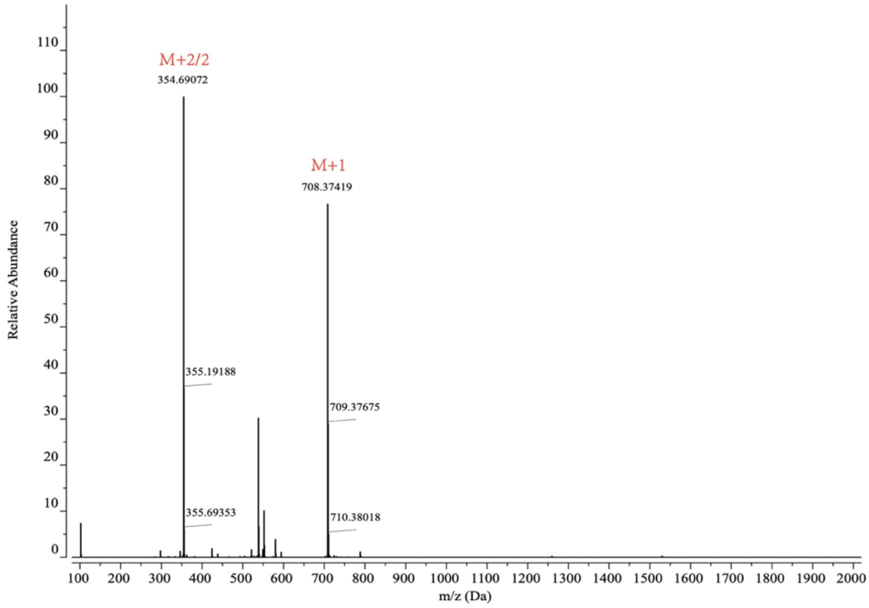

MS-trace 2n

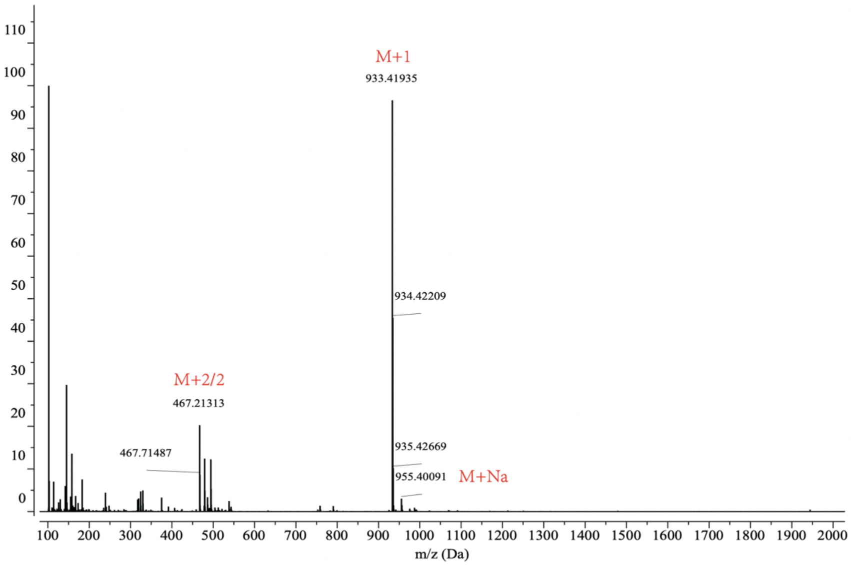

XIII. Supplementary Fig. 8. Procedure for the modification of linear peptide 1o containing cysteine to generate 3-thio *N*-pyrrole product 2o

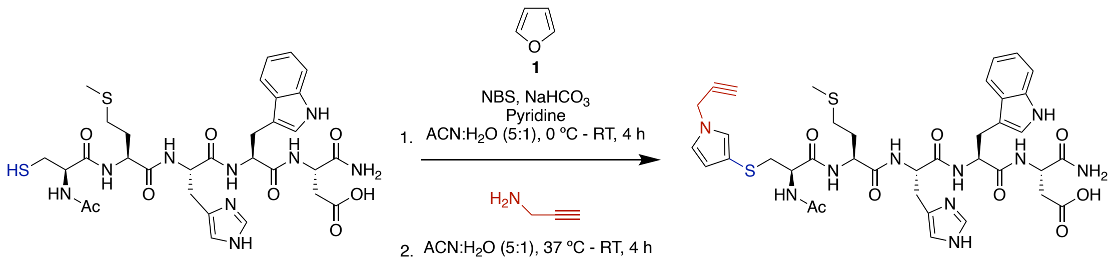

Furan **1** (100 µL, 1.38 mmol) and sodium bicarbonate (115 mg, 1.38 mmol, 1 equiv.) were added to a solution of 12 mL ACN:H_2O (5:1). The reaction mixture was cooled to 0 °C and left to stir for 15 min. N-Bromosuccinimide (244 mg, 1.38 mmol, 1 equiv.) was dissolved in a solution of 12 mL ACN:H_2O (5:1) and added to the reaction mixture dropwise. Afterward, the reaction mixture was left to stir for 10 min, and pyridine (222 µL, 2.76 mmol, 2 equiv.) was added to the reaction mixture. The reaction mixture was stirred for 4 h and used without further purification. 1.5 equiv. (68 µL) of the reaction mixture was taken from a pot and incubated with 1.9 mg of Ac-CMHWD (**1o**, 1 equiv.) dissolved in 5 mL of acetonitrile and 1 mL of water at 37 °C for 30 min. The concentration of peptide in the reaction mixture was 1.13 µM. Propargylamine (1.2 equiv.) was then added to the reaction mixture and left to stir for 4 h at room temperature. The reaction mixture was analyzed by HPLC using method A to determine the percent conversion to modified product **2o** (78.7 %).

Ac-CMHWD linear peptide 1o. LCMS *m/z* 732.2545 (calcd. [M+H^+] = 732.25), *m/z* 1463.5021 (calcd. [2M+H^+] = 1463.5), Purity: > 95 % (HPLC analysis at 220 nm). Retention time in HPLC: 9.896 min.

Ac-CMHWD modified peptide 2o. LCMS *m/z* 835.2959 (calcd. [M+H^+] = 835.29), Purity: > 95 % (HPLC analysis at 220 nm). Retention time in HPLC: 12.660 min.

HPLC trace of 1o

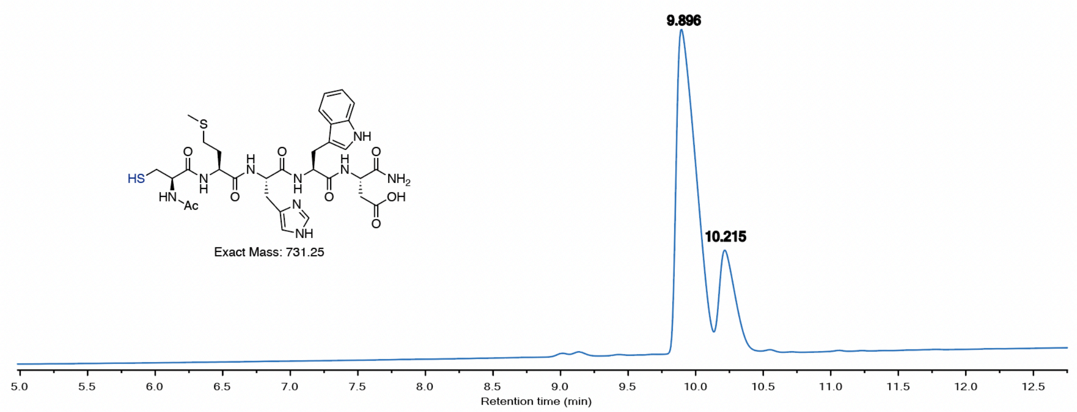

HPLC trace of 2o

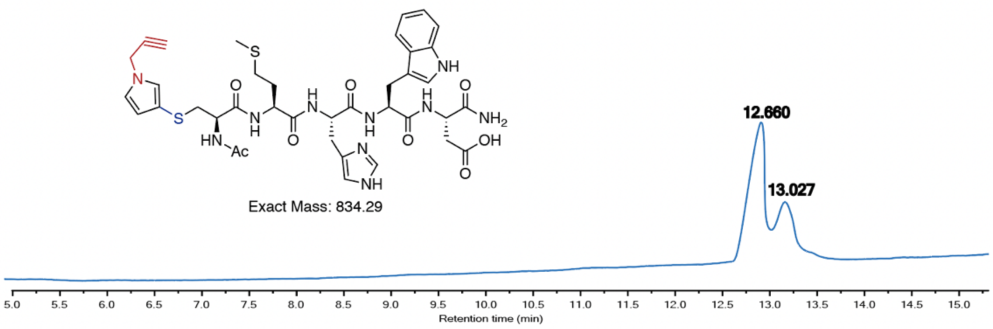

MS-trace of 1o

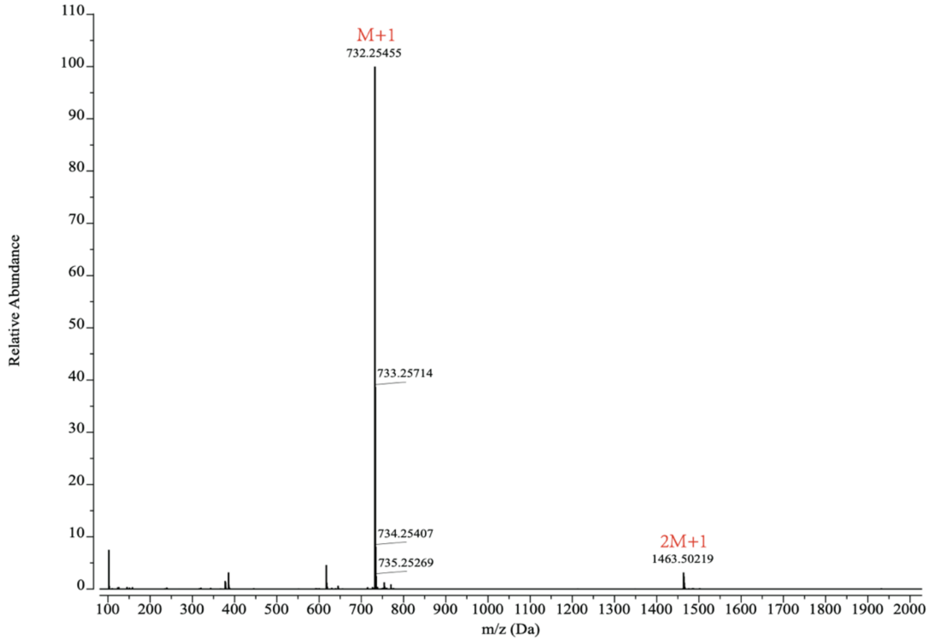

MS-trace of 2o

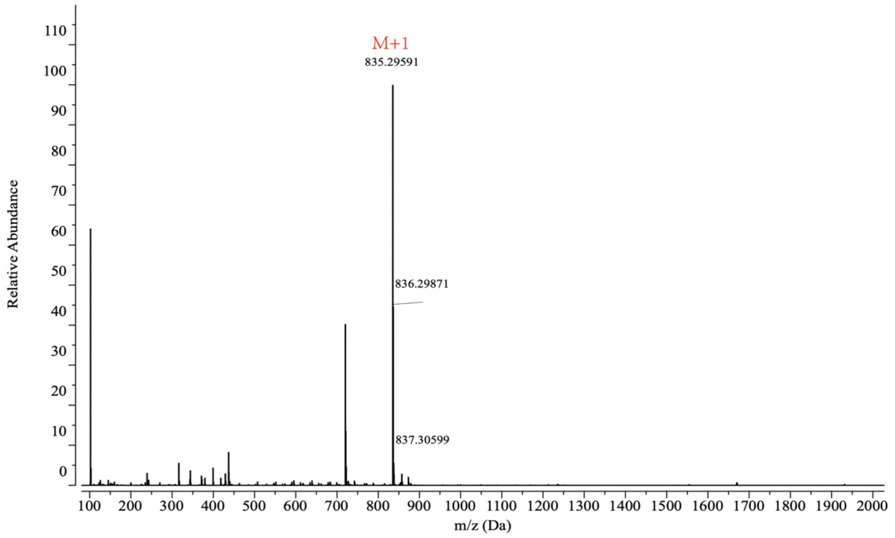

XIV. Supplementary Fig. 9. Procedure for the modification of linear peptide **1p** and **1q** containing furan to generate 3-thio *N*-pyrrole products **2p** and **2q**

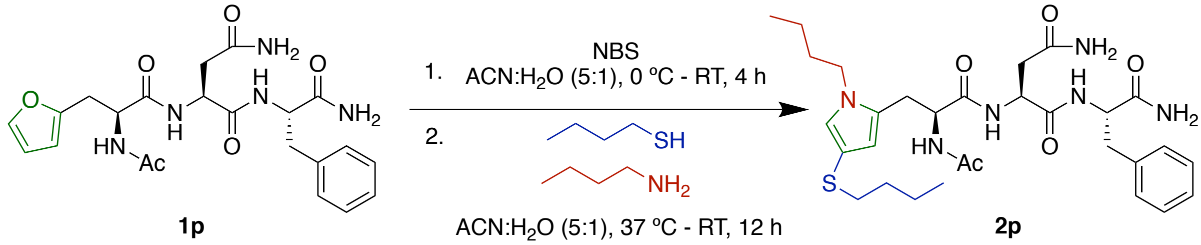

1.5 mg of peptide **1p** (3.3 µmol, 1 equiv.) was dissolved in 2 mL of ACN:H_2O (5:1). NBS (1.8 mg, 9.3 µmol, 3 equiv.) was added in one portion at 0 °C and left to stir at RT for 4h. 1-butanethiol (2.1 µL, 19.7 µmol, 6 equiv.) and 1-butylamine (1.6 µL, 16.4 mmol, 5 equiv.) were then added from prepared stock solutions in ACN to the reaction vial. The solution was stirred at RT for 12 h. Following the completion of the reaction, the solvent was removed using a centrifugal vacuum concentrator system. The reaction mixture was re-dissolved in 350 µL of ACN:H_2O (5:1) and was analyzed by HPLC using method A to determine the percent conversion to modified product **2p** (87 %).

Ac-A$_{\mathbf{fur}}$NF linear peptide 1p. LCMS *m/z* 458.1998 (calcd. [M+H$^+$] = 458.2034), *m/z* 480.1815 (calcd. [M+Na$^+$] = 480.1859), *m/z* 496.1552 (calcd. [M+K$^+$] = 496.5853), *m/z* 915.3927 (calcd. [2M+H$^+$] = 915.3995), *m/z* 937.3742 (calcd. [2M+Na$^+$] = 937.3820), *m/z* 953.3478 (calcd. [2M+K$^+$] = 953.3560), Purity: > 99 % (HPLC analysis at 220 nm). Retention time in HPLC: 11.354 min.

Ac-A$_{\mathbf{fur}}$NF modified peptide 2p. LCMS *m/z* 601.3122 (calcd. [M+H$^+$] = 601.3167), *m/z* 623.2938 (calcd. [M+Na$^+$] = 623.3992), *m/z* 639.2676 (calcd. [M+K$^+$] = 639.2731), *m/z* 1223.5984 (calcd. [2M+Na$^+$] = 1223.6085), Purity: > 99 % (HPLC analysis at 220 nm). Retention time in HPLC: 20.712 min.

HPLC trace of 1p

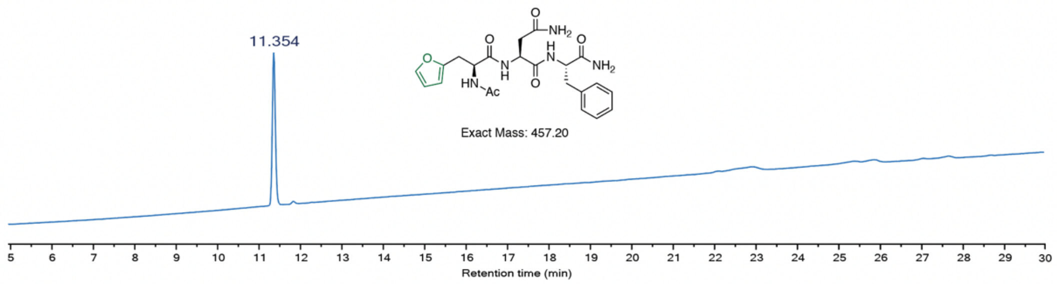

HPLC trace of 2p

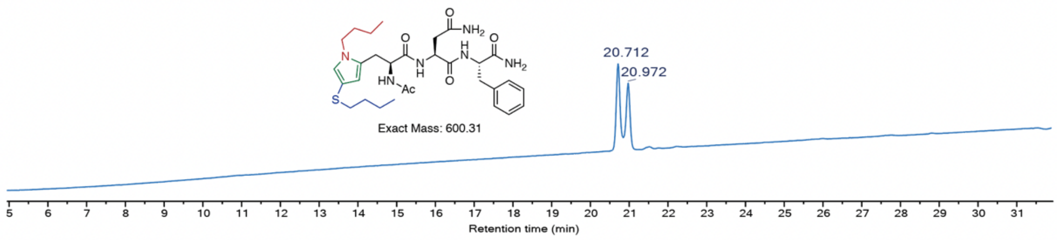

MS-trace of 1p

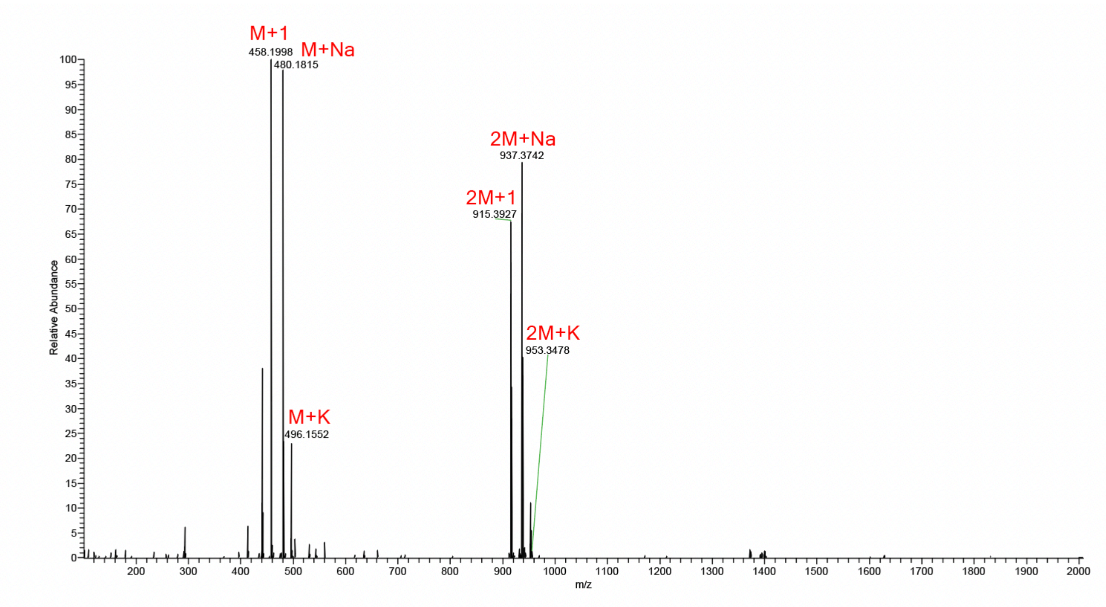

MS-trace of 2p

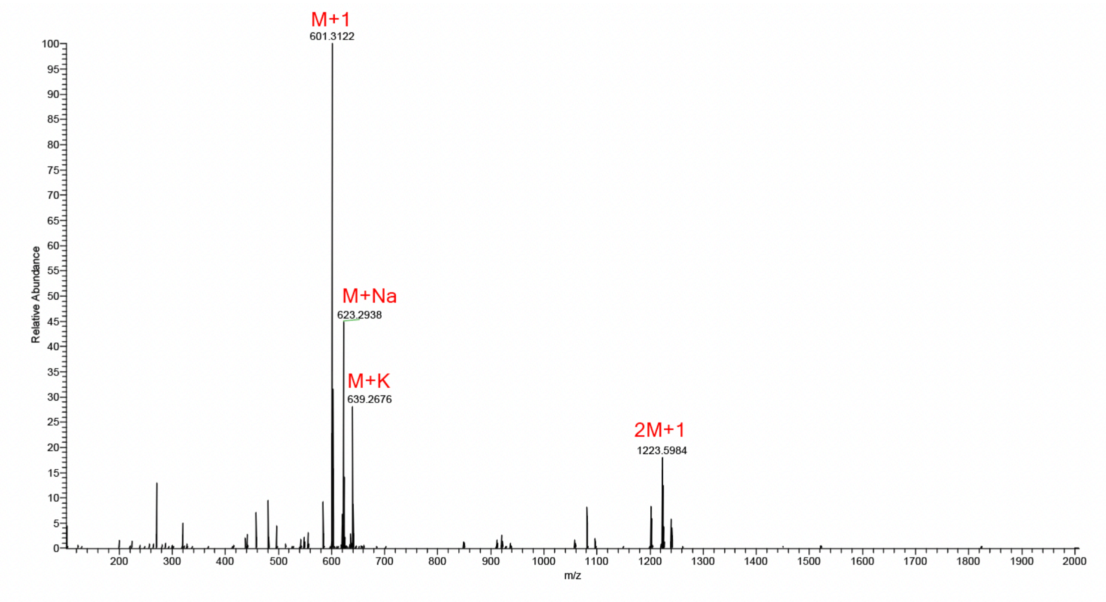

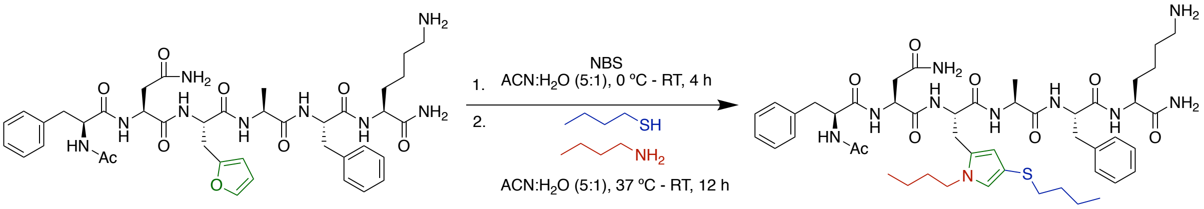

1q **2q**

1 mg of peptide **1q** (1.2 μmol, 1 equiv.) was dissolved in 1.5 mL of ACN:H_2O (5:1). NBS (0.66 mg, 3.7 μmol, 3 equiv.) was added in one portion at RT and the reaction mixture was stirred for 4 h. Next, 1-butanethiol (1.3 μL, 12.4 μmol, 10 equiv.) and 1-butylamine (1.2 μL, 12.4 μmol, 10 equiv.) were added from freshly prepared stock solutions in ACN to the reaction mixture. The solution was stirred at RT for 12 h. Following completion of the reaction, the solvent was removed using a centrifugal vacuum concentrator system. The product was re-dissolved in 350 μL of ACN:H_2O (5:1). The reaction mixture was analyzed by HPLC using method A to determine the percent conversion to the modified product **2q** (53 %).

Ac-FNA$_{\mathbf{fur}}$AFK linear peptide 1q. LCMS *m/z* 804.5552 (calcd. [M+H$^+$] = 804.4044), *m/z* 1607.8068 (calcd. [2M+H$^+$] = 1607.8088), Purity: > 95 % (HPLC analysis at 220 nm). Retention time in HPLC: 11.941 min.

Ac-FNA$_{\mathbf{fur}}$AFK modified peptide 2q. LCMS *m/z* 474.5653 (calcd. [(M+2H$^+$)/2] = 474.2589), *m/z* 947.6648 (calcd. [M+H$^+$] = 947.5177), *m/z* 969.5157 (calcd. [M+Na$^+$] = 969.5177), Purity: > 90 % (HPLC analysis at 220 nm). Retention time in HPLC: 17.503 min.

HPLC trace of 1q

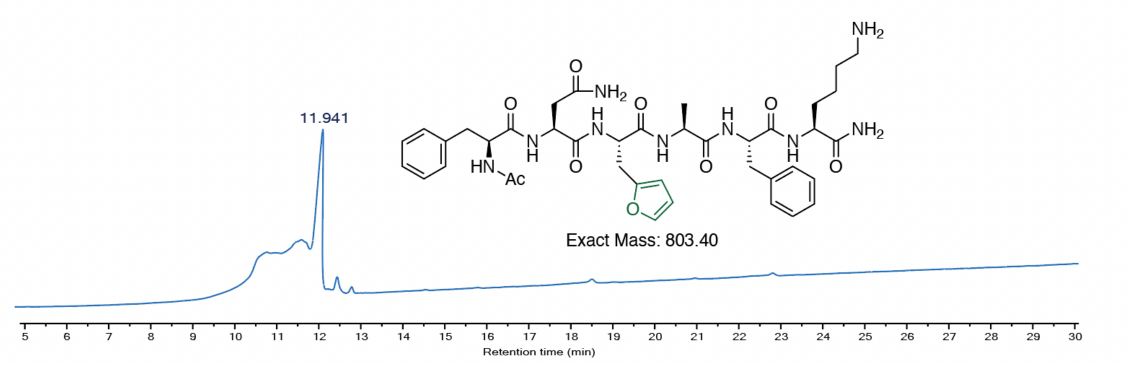

HPLC trace of 2q

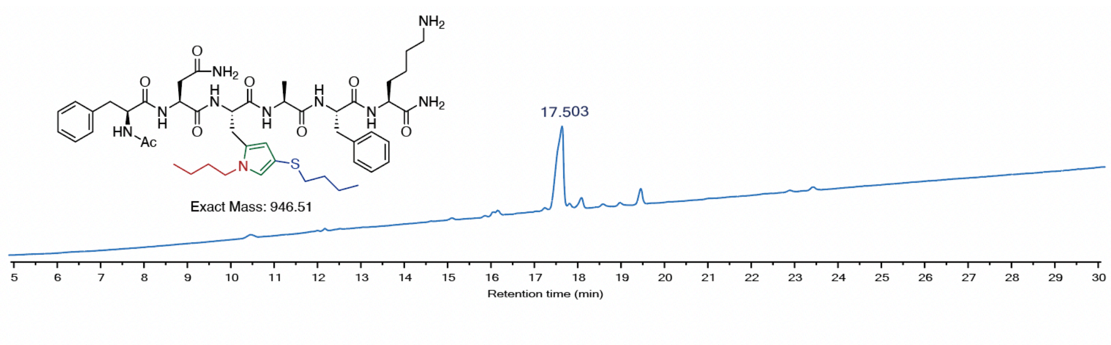

MS-trace of 1q

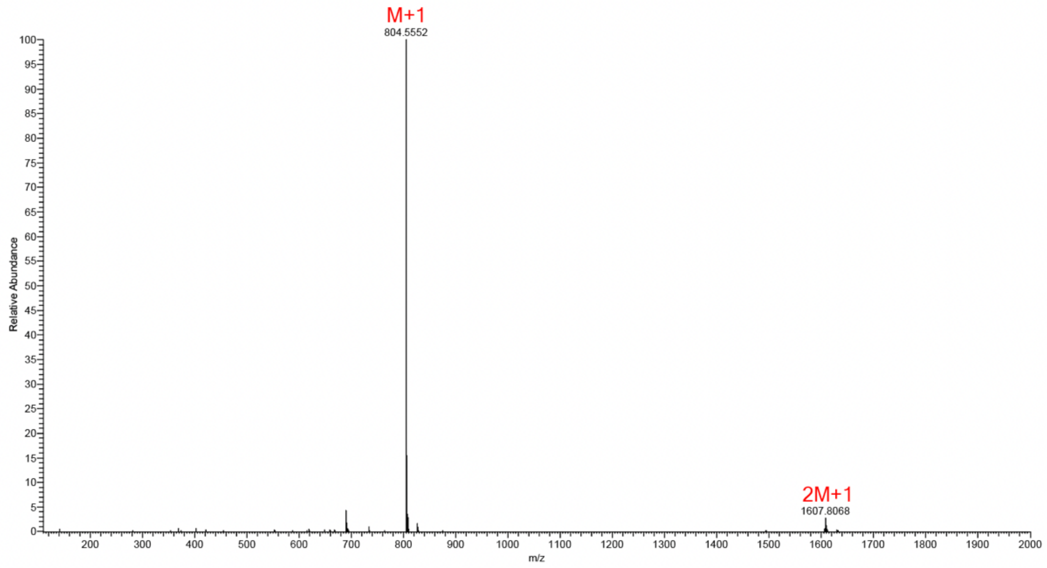

MS-trace of 2q

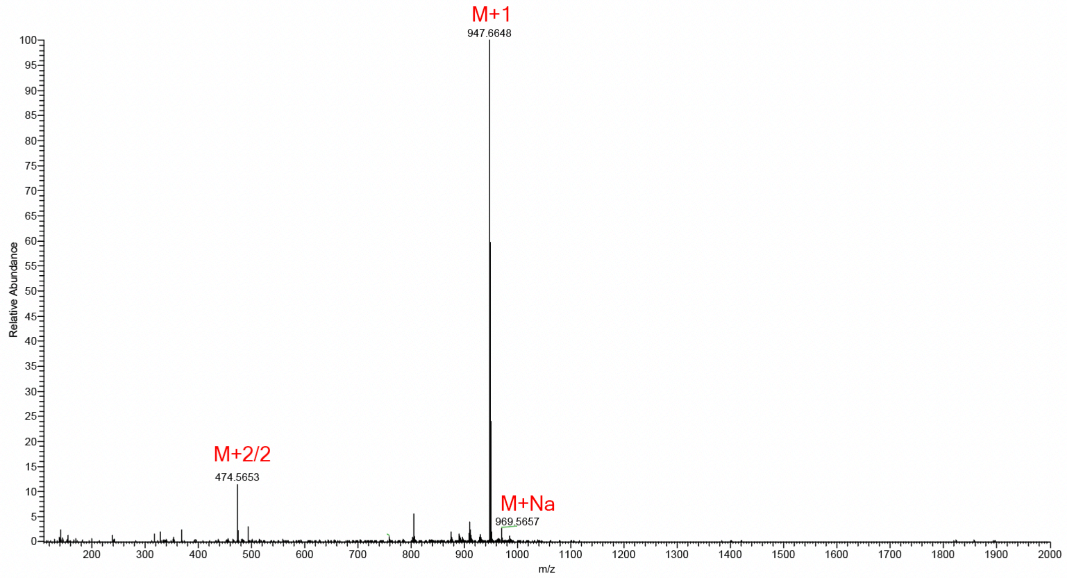

XV. Supplementary Fig. 10. Procedure for the macrocyclization of linear peptides **1r-1t** to generate macrocycles **2r-2t**.

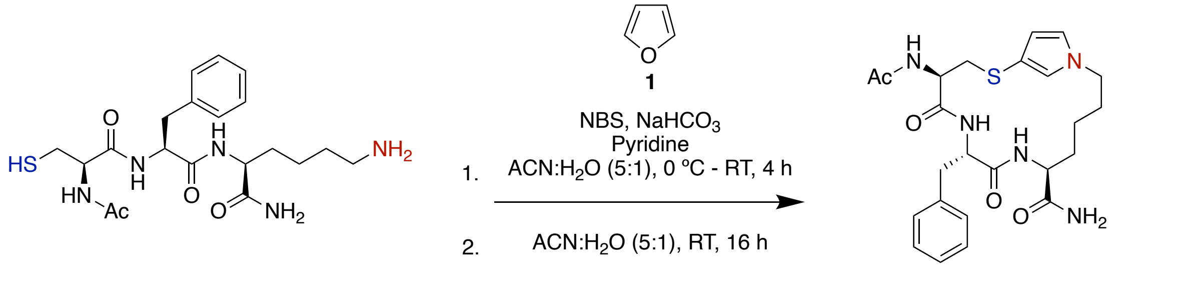

1r **2r**

A solution of furan **1** (100 µL, 1.4 mmol, 1 equiv.) and sodium bicarbonate (118 mg, 1.4 mmol, 1 equiv.) in 12 mL of ACN:H_2O (5:1) was incubated at 0 °C for 15 min. A 12 mL solution of NBS (244 mg, 1.4 mmol, 1 equiv.) in ACN:H_2O (5:1) was added to the mixture dropwise over 5 min at 0 °C and the reaction was stirred for 10 min. Pyridine (222 µL, 2.8 mmol, 2 equiv.) was then added directly and the reaction mixture was allowed to stir at RT for 4 h and used without further purification. 1.2 equiv. (47 µL) of the reaction mixture was taken from a pot and incubated with 1 mg of peptide **1r** (2.3 µmol, 1 equiv.) in a 6 mL solution of ACN:H_2O (5:1) and the reaction was allowed to stir at RT for 16 h. Following completion of the reaction, the solvent was removed using a centrifugal vacuum concentrator system. The product was re-dissolved in 350 µL of ACN:H_2O (5:1) and analyzed by HPLC using method A to determine the percent conversion to product **2r** (95 %).

Ac-CFK linear peptide 1r. LCMS *m/z* 438.2144 (calcd. [M+H$^+$] = 438.2175), Purity: > 95 % (HPLC analysis at 220 nm). Retention time in HPLC: 6.454 min.
Ac-CFK cyclic peptide 2r. LCMS *m/z* 486.4123 (calcd. [M+H$^+$] = 486.2175), *m/z* 508.1960 (calcd. [M+Na$^+$] = 508.1994), *m/z* 971.4217 (calcd. [2M+H$^+$] = 971.4272), *m/z* 993.4034 (calcd. [2M+Na$^+$] = 993.4091), Purity: > 95 % (HPLC analysis at 220 nm). Retention time in HPLC: 12.608 min.

HPLC trace of 1r

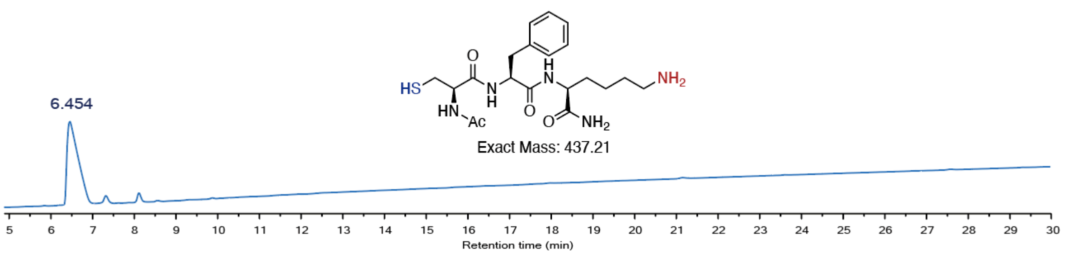

HPLC trace of 2r

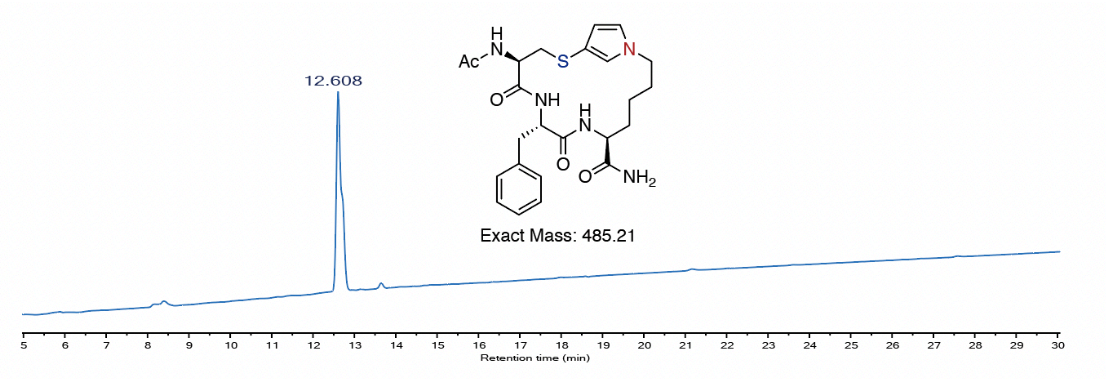

MS-trace of 1r

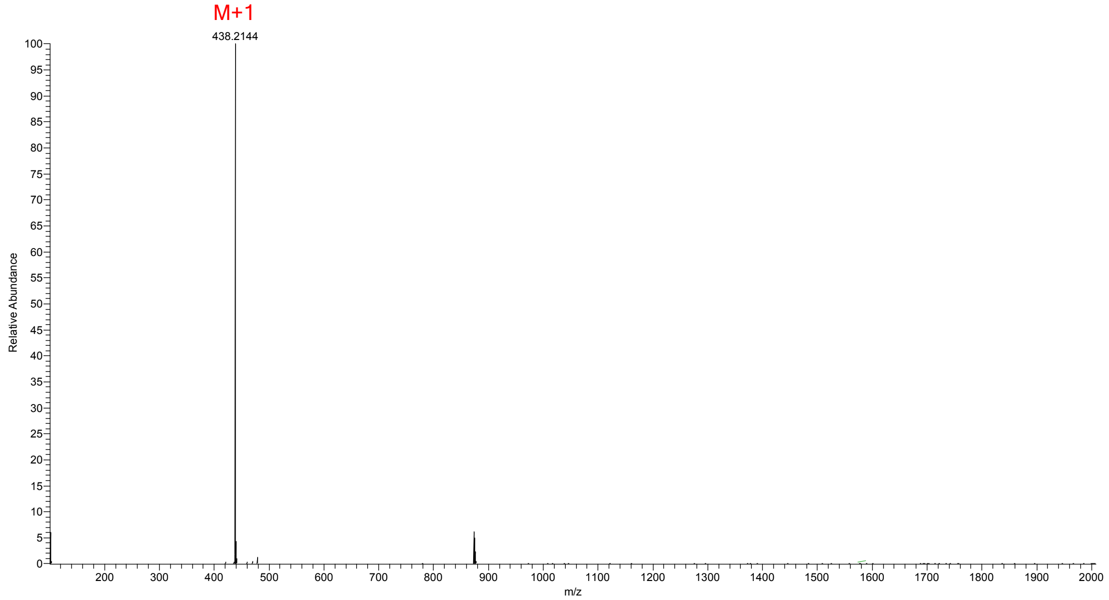

MS-trace of 2r

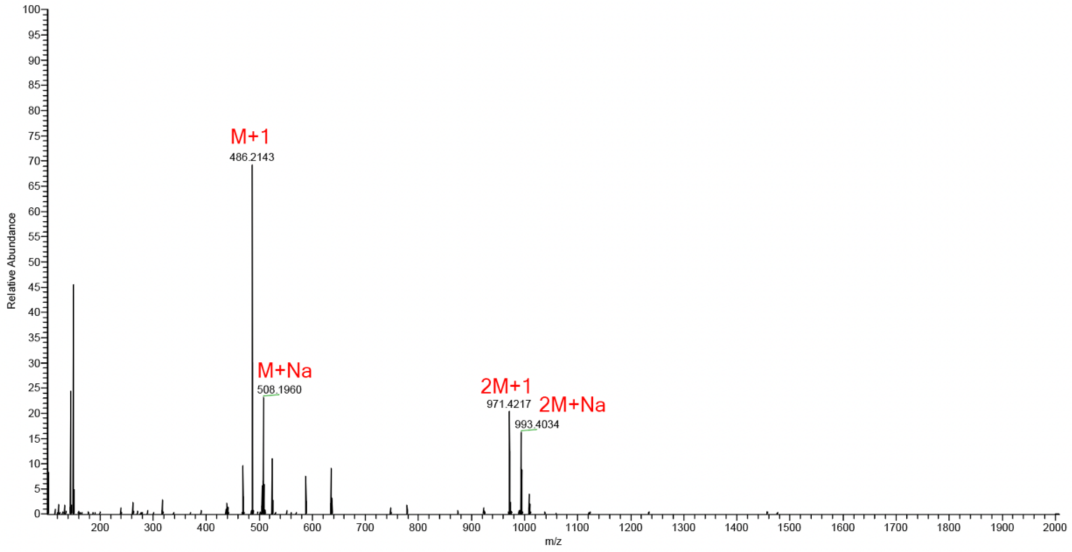

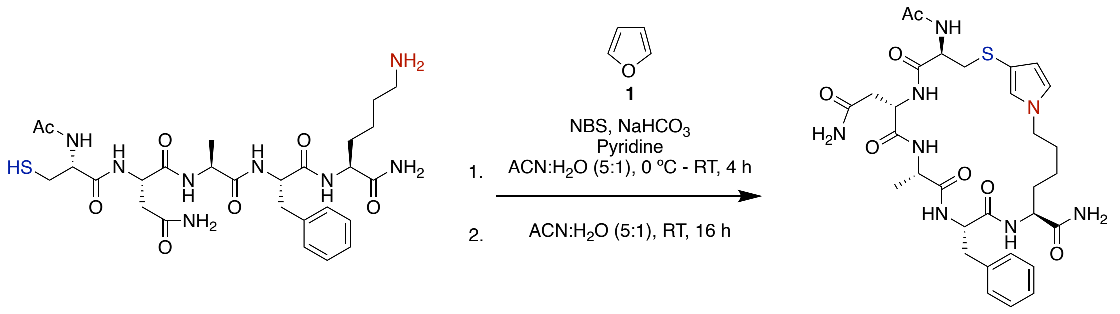

1s **2s**

A solution of furan **1** (100 µL, 1.4 mmol, 1 equiv.) and sodium bicarbonate (118 mg, 1.4 mmol, 1 equiv.) in 12 mL of ACN:H_2O (5:1) was incubated at 0 °C for 15 min. A 12 mL solution of NBS (244 mg, 1.4 mmol, 1 equiv.) in ACN:H_2O (5:1) was added to the mixture dropwise over 5 min at 0 °C and the reaction was stirred for 10 min. Pyridine (222 µL, 2.8 mmol, 2 equiv.) was then added directly and the reaction mixture was allowed to stir at 0 °C for 4 h. This afforded the oxidized intermediate *cis*-2-butene-1,4-dial (BDA), which was not isolated. This reaction served as a stock solution of the reactive intermediate.

From a freshly prepared stock solution of prepared *cis*-2-butene-1,4-dial (BDA), a 33.1 µL aliquot (1.9 µmol, 1.2 equiv.) was added to a 1.9 mL solution of ACN:H_2O (5:1) containing 1 mg of peptide **1s** (1.6 µmol, 1 equiv.). The reaction was allowed to stir at RT for 16 h. Following completion of the reaction, solvent was removed using a centrifugal vacuum concentrator system. The product was re-dissolved in 350 µL of ACN:H_2O (5:1) and analyzed by HPLC using method A to determine the percent conversion to the product **2s** (> 99 %).

Ac-CNAFK linear peptide 1s. LCMS *m/z* 623.2724 (calcd. [M+H^+] = 623.2975), *m/z* 1245.5378 (calc. [2M+H^+] = 1245.5873), Purity: > 99 % (HPLC analysis at 220 nm). Retention time in HPLC: 7.108 min.

Ac-CNAFK cyclic peptide 2s. LCMS *m/z* 671.2966 (calcd. [M+H^+] = 671.2975), *m/z* 693.2785 (calcd. [M+Na^+] = 693.2795), *m/z* 709.2523 (calcd. [M+K^+] = 709.2534), Purity: > 99 % (HPLC analysis at 220 nm). Retention time in HPLC: 13.874 min.

HPLC trace of 1s

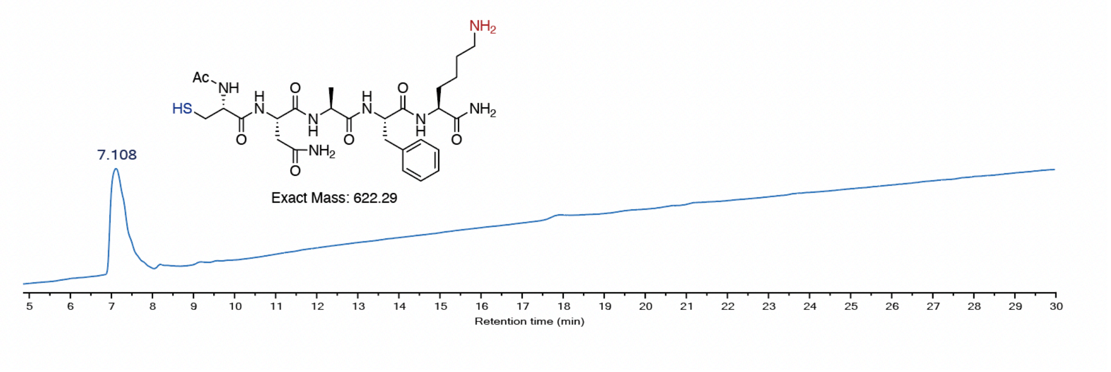

HPLC trace of 2s

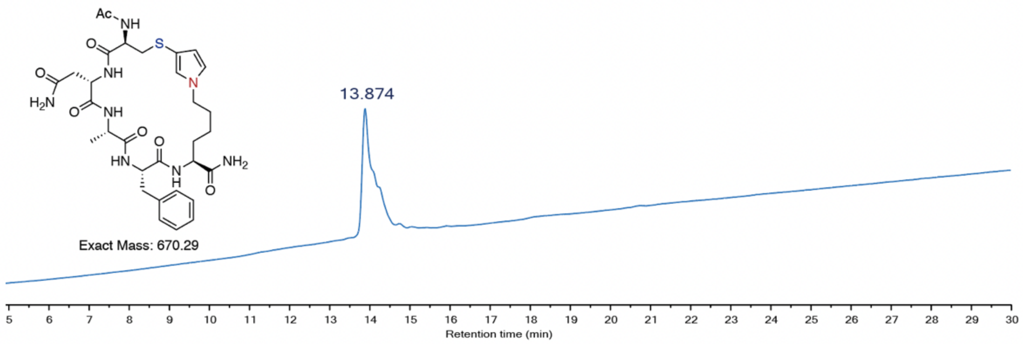

MS-trace of 1s

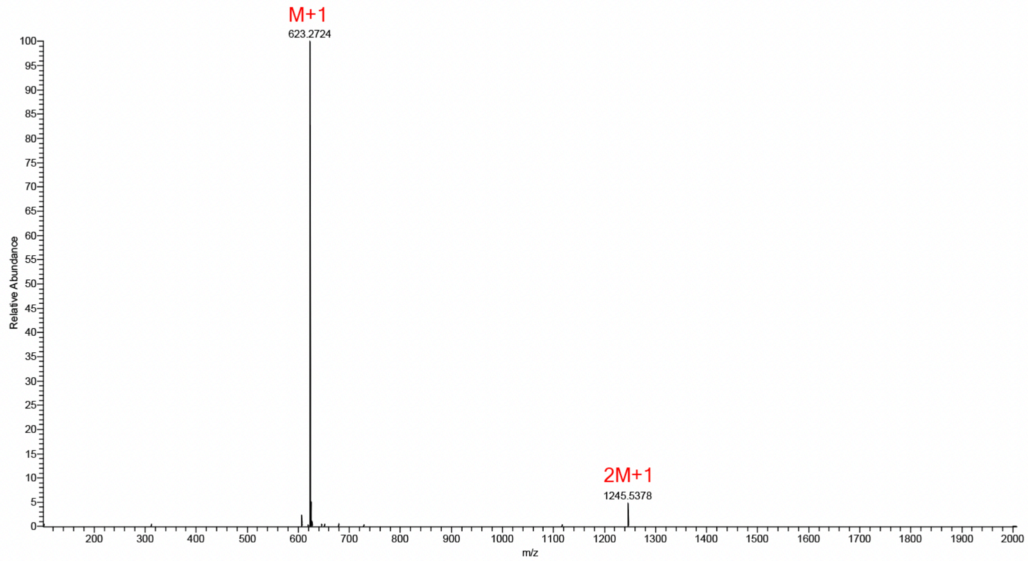

MS-trace of 2s

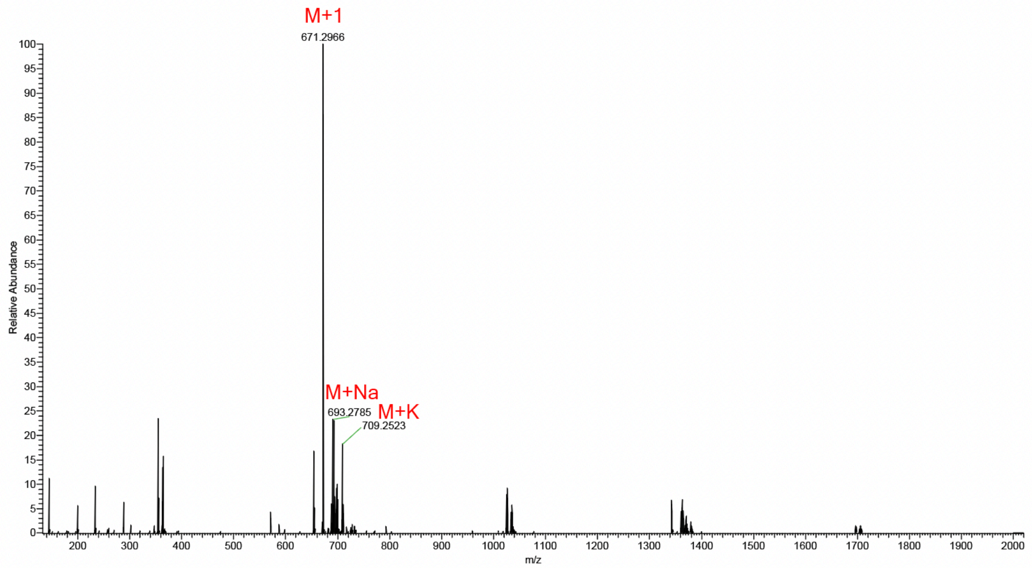

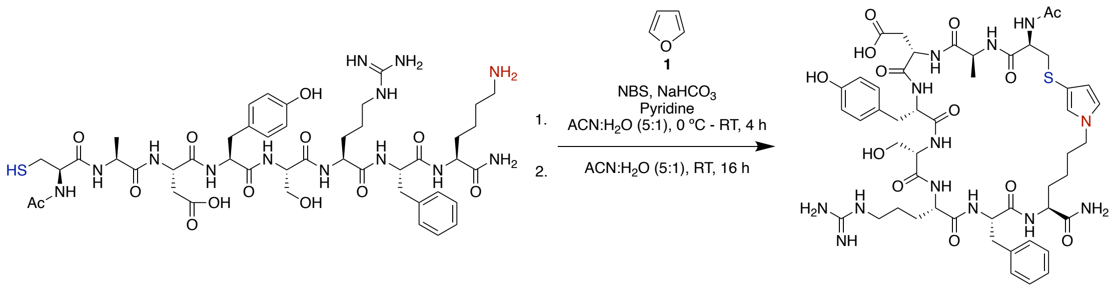

1t **2t**

A solution of furan **1** (100 µL, 1.4 mmol, 1 equiv.) and sodium bicarbonate (118 mg, 1.4 mmol, 1 equiv.) in 12 mL of ACN:H_2O (5:1) was incubated at 0 °C for 15 min. A 12 mL solution of NBS (244 mg, 1.4 mmol, 1 equiv.) in ACN:H_2O (5:1) was added to the mixture dropwise over 5 min at 0 °C and the reaction was stirred for 10 min. Pyridine (222 µL, 2.8 mmol, 2 equiv.) was then added directly and the reaction mixture was allowed to stir at 0 °C for 4 h. This afforded the oxidized intermediate *cis*-2-butene-1,4-dial (BDA), which was not isolated. This reaction served as a stock solution of the reactive intermediate.

From a freshly prepared stock solution of prepared *cis*-2-butene-1,4-dial (BDA), a 20 µL aliquot (1.2 µmol, 1.2 equiv.) was added to a 1.2 mL solution of ACN:H_2O (5:1) containing 1 mg of peptide **1t** (1.0 µmol, 1 equiv.). The reaction was allowed to stir at RT for 16 h. Following completion of the reaction, the solvent was removed using a centrifugal vacuum concentrator system. The product was re-dissolved in 350 µL of ACN:H_2O (5:1) and analyzed by HPLC using method A to determine the percent conversion to the product **2t** (78 %).

Ac-CADYSRFK linear peptide 1t. LCMS *m/z* 516.0042 (calcd. [$(M+2H^+)/2$] = 515.7430), *m/z* 1030.5527 (calcd. [$M+H^+$] = 1030.4775), Purity: > 95 % (HPLC analysis at 220 nm). Retention time in HPLC: 7.975 min.

Ac-CADYSRFK cyclic peptide 2t. LCMS *m/z* 1078.6207 (calcd. [$M+H^+$] = 1078.4780), Purity > 95 % (HPLC analysis at 220 nm). Retention time in HPLC: 11.976 min.

HPLC trace of 1t

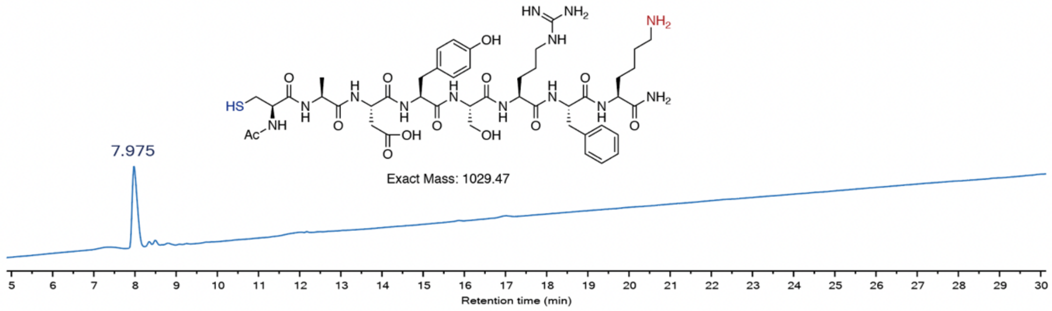

HPLC trace of 2t

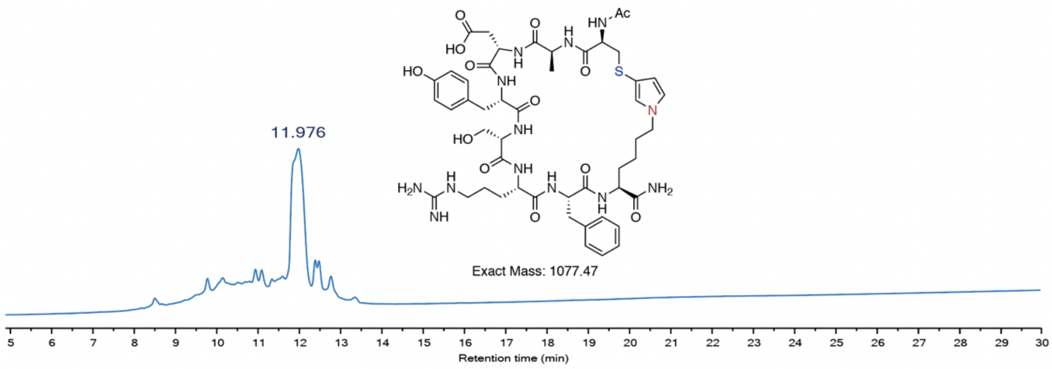

MS-trace of 1t

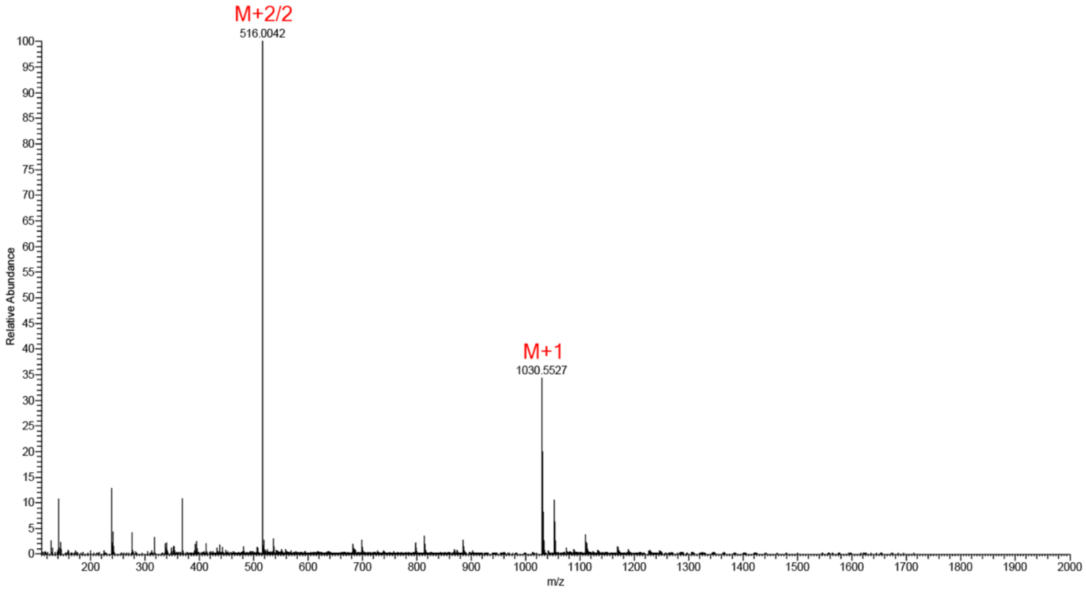

MS-trace of 2t

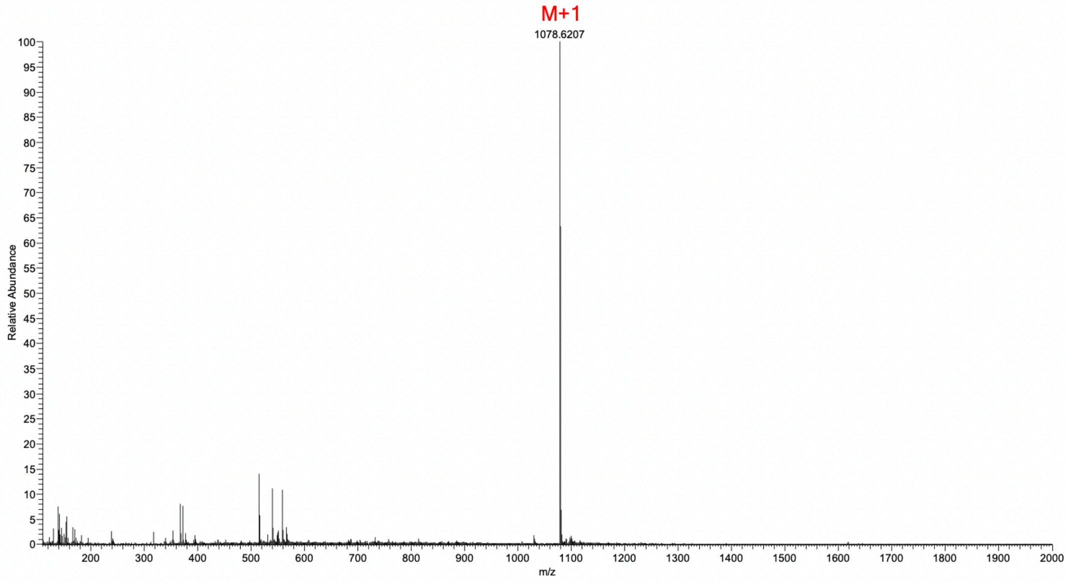

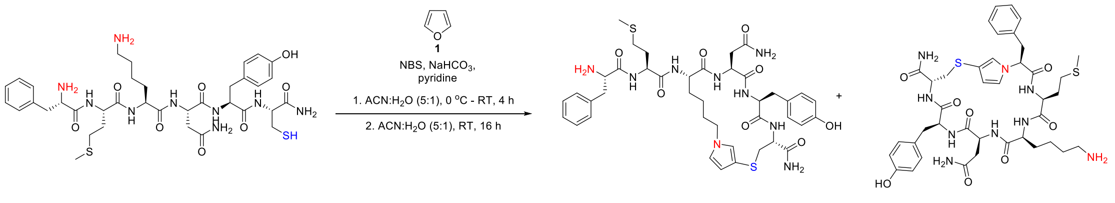

A solution of furan **1** (100 µL, 1.4 mmol, 1 equiv.) and sodium bicarbonate (118 mg, 1.4 mmol, 1 equiv.) in 12 mL of ACN:H_2O (5:1) was incubated at 0 °C for 15 min. A 12 mL solution of NBS (244 mg, 1.4 mmol, 1 equiv.) in ACN:H_2O (5:1) was added to the mixture dropwise over 5 min at 0 °C and the reaction was stirred for 10 min. Pyridine (222 µL, 2.8 mmol, 2 equiv.) was then added directly and the reaction mixture was allowed to stir at 0 °C for 4 h. This afforded the oxidized intermediate *cis*-2-butene-1,4-dial (BDA), which was not isolated. This reaction served as a stock solution of the reactive intermediate.

From a freshly prepared stock solution of prepared *cis*-2-butene-1,4-dial (BDA), a 114 µL aliquot (7.5 µmol, 1.2 equiv.) was added to a 6.0 mL solution of ACN:H_2O (5:1) containing 5 mg of peptide **1u** (6.2 µmol, 1 equiv.). The reaction was allowed to stir at RT for 16 h. Following completion of the reaction, the solvent was removed using a centrifugal vacuum concentrator system. The product was re-dissolved in 600 µL of ACN:H_2O (5:1) and analyzed by HPLC using method B to determine the percent conversion to the major products **2u** (31 %) and **2u'** (40 %), representing 71 % total conversion to cyclic pyrrole peptides. The starting material was observed to have been consumed. The peaks of these two major products were collected and lyophilized.

FMKNYC Linear Peptide 1u. LCMS *m/z* 804.3488 (calcd. $[M{+}H^+]$ = 804.3531), *m/z* 402.6894 (calcd. $[(M{+}2H^+)/2]$ = 402.6802). Purity: > 95 % (HPLC analysis at 220 nm). Retention time in HPLC: 9.726 min.

FMKNYC cyclic peptide 2u. LCMS *m/z* 852.3658 (calcd. $[M{+}H^+]$ = 852.3531), *m/z* 426.6864 (calcd. $[(M{+}2H^+)/2]$ = 426.6765). Retention time in HPLC: 16.395 min.

FMKNYC cyclic peptide 2u'. LCMS *m/z* 852.3685 (calcd. $[M{+}H^+]$ = 852.3531), *m/z* 874.3476 (calcd. $[M{+}Na]$ = 874.3531). Retention time in HPLC: 19.214 min.

HPLC trace of 1u

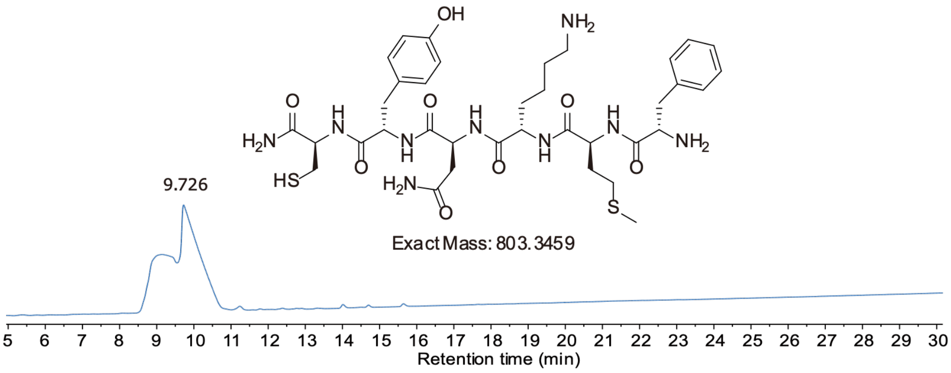

Prep-HPLC trace of reaction mixture to generate 2u and 2u'

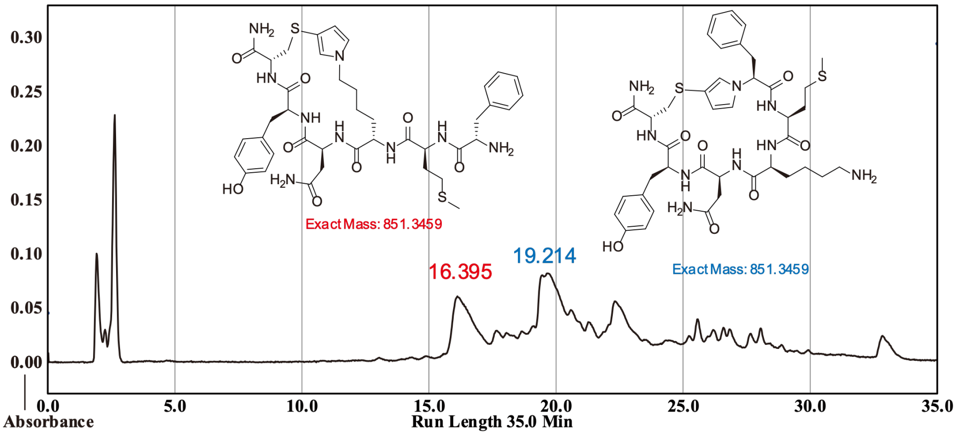

MS-trace of 1u

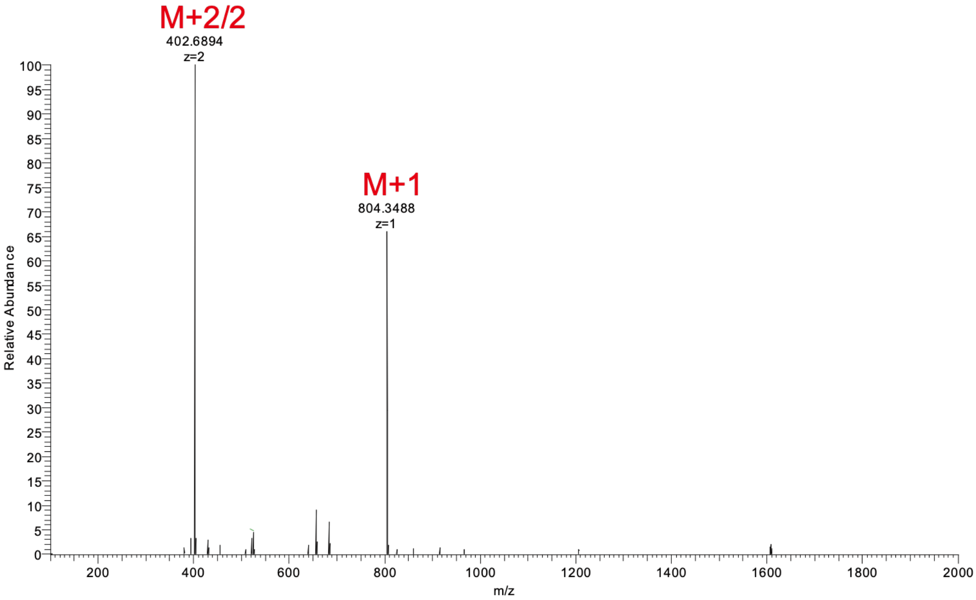

MS-trace of 2u

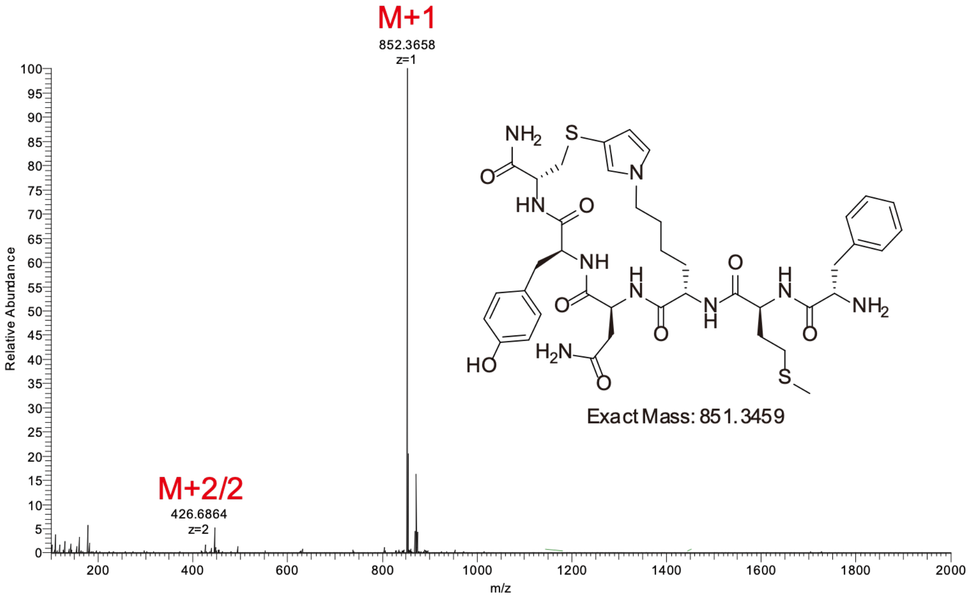

MS-trace of 2u'

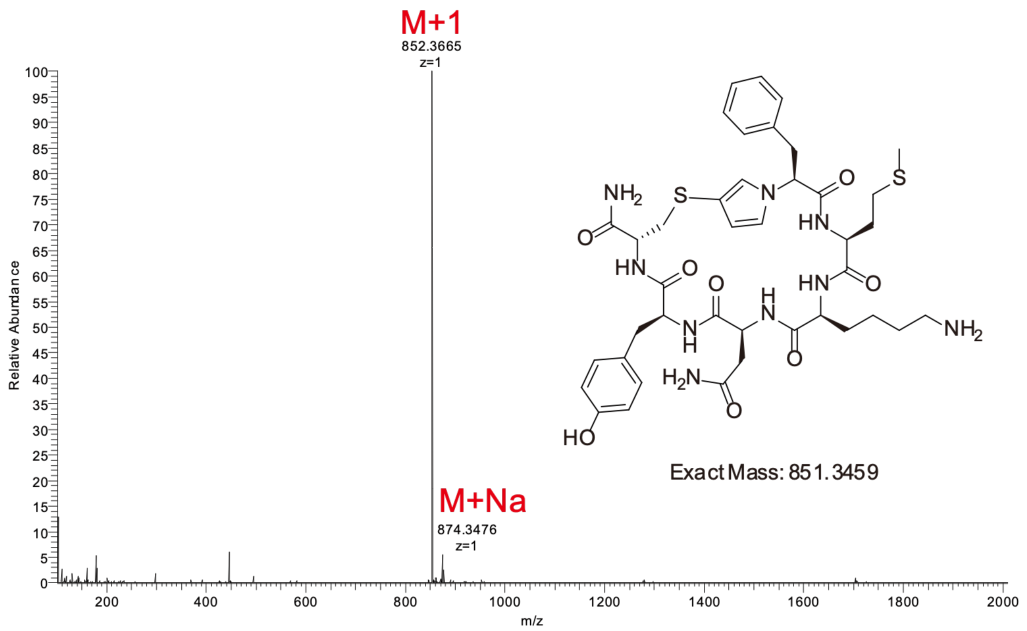

CNBr cleavage to determine site of modifications

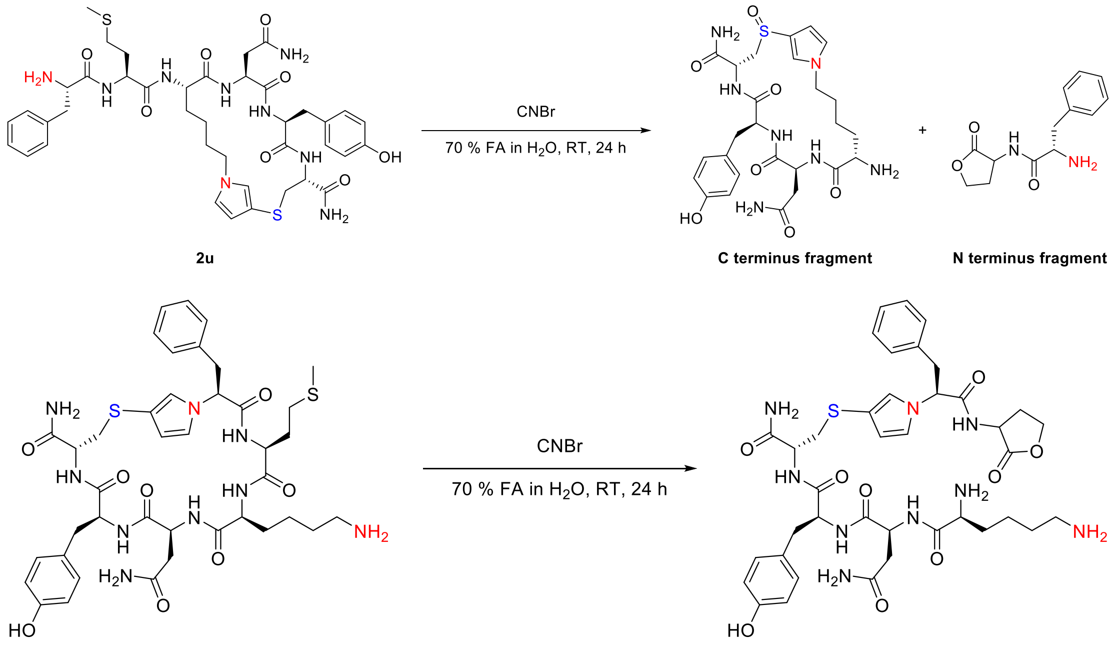

To determine the position of cyclization, cyanogen bromide (CNBr) was used to cleave the cyclic peptide at the methionine residue. To ~0.5 mg of products **2u** and **2u'** was added ~10 mg of CNBr in 500 µL of 70 % aqueous formic acid in 2 mL glass vials which were capped and allowed to stir in the dark for 24 h, before careful evaporation of the acid and HRMS analysis. The results clearly showed the two major products to be cyclization of the cysteine with either of the two amine residues.

N-terminal cleavage product of 2u. LCMS *m/z* 249.1268 (calcd. [M+H$^+$] = 249.1234).
C-terminal cleavage product of 2u. LCMS *m/z* 590.2283 (calcd. [M+H$^+$] = 590.2319).
Cleavage product of 2u'. LCMS *m/z* 822.3598 (calcd. [M+H$^+$] = 822.3603), *m/z* 411.6951 (calcd. [(M+2H$^+$)/2] = 411.6802).

MS trace of C & N terminus fragments of 2u after cleavage

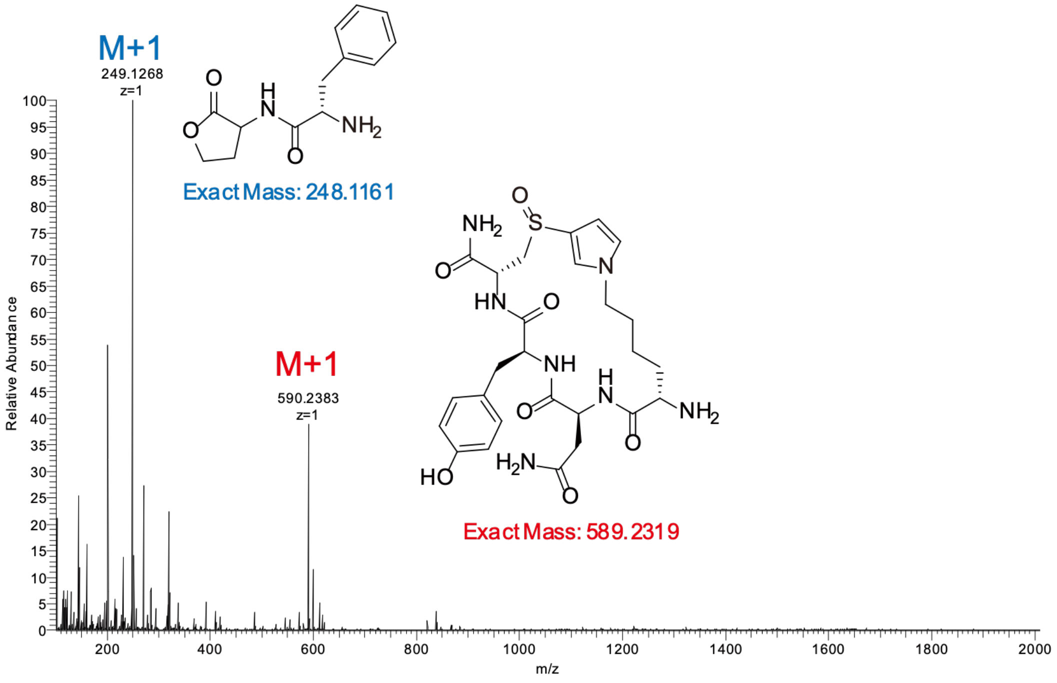

MS trace of 2u' after cleavage

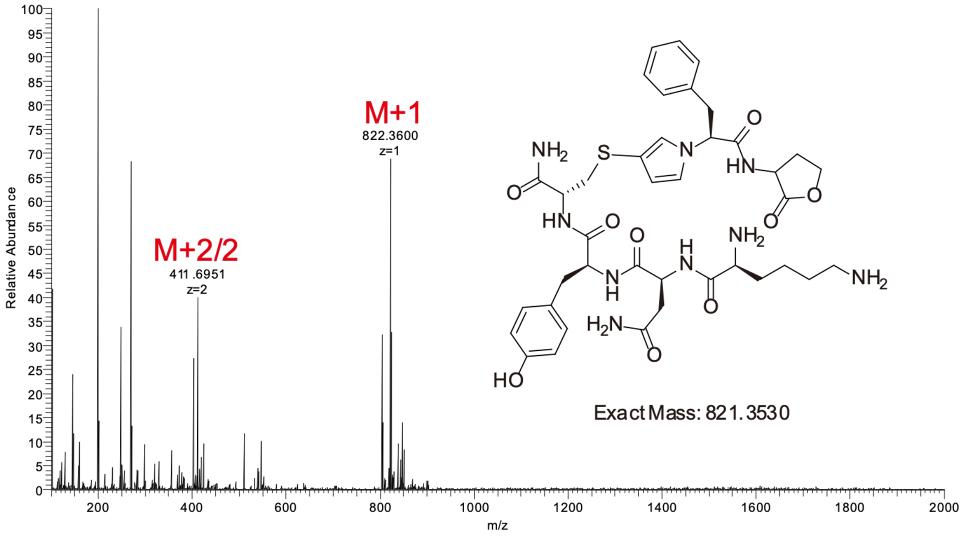

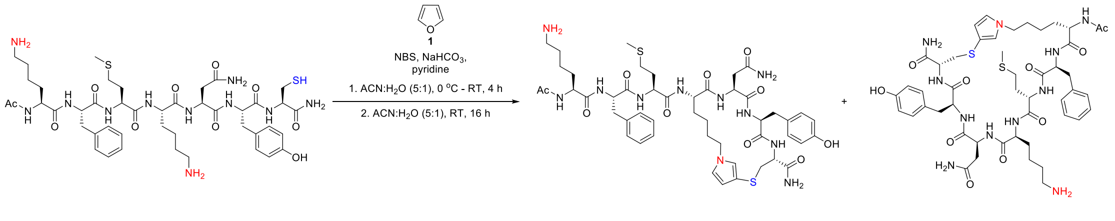

1v **2v** **2v'**

A solution of furan **1** (100 µL, 1.4 mmol, 1 equiv.) and sodium bicarbonate (118 mg, 1.4 mmol, 1 equiv.) in 12 mL of ACN:H_2O (5:1) was incubated at 0 ^oC for 15 min. A 12 mL solution of NBS (244 mg, 1.4 mmol, 1 equiv.) in ACN:H_2O (5:1) was added to the mixture dropwise over 5 min at 0 ^oC and the reaction was stirred for 10 min. Pyridine (222 µL, 2.8 mmol, 2 equiv.) was then added directly and the reaction mixture was allowed to stir at 0 ^oC for 4 h. This afforded the oxidized intermediate *cis*-2-butene-1,4-dial (BDA), which was not isolated. This reaction served as a stock solution of the reactive intermediate.
From a freshly prepared stock solution of prepared *cis*-2-butene-1,4-dial (BDA), a 94 µL aliquot (6.2 µmol, 1.2 equiv.) was added to a 6.0 mL solution of ACN:H_2O (5:1) containing 5 mg of peptide **1v** (5.1 µmol, 1 equiv.). The reaction was allowed to stir at RT for 16 h. Following completion of the reaction, the solvent was removed using a centrifugal vacuum concentrator system. The product was re-dissolved in 600 µL of ACN:H_2O (5:1) and analyzed by HPLC using method B to determine the percent conversion to the major products **2v** (38 %) and **2v'** (35 %), representing 73 % total conversion to cyclic pyrrole peptides. The starting material was observed to have been consumed. The peaks of these two major products were collected and lyophilized.

Ac-KFMKNYC linear peptide 1v. LCMS *m/z* 974.4534 (calcd. $[M\text{+}H^{+}]$ = 974.4587), *m/z* 487.7348 (calcd. $[(M\text{+}2H^{+})/2]$ = 487.7330). Purity: > 95 % (HPLC absorbance at 220 nm). Retention time in HPLC: 13.3 min.
Ac-KFMKNYC cyclic peptide 2v. LCMS *m/z* 1022.4757 (calcd. $[M\text{+}H^{+}]$ = 1022.4587). Retention time in HPLC: 16.936 min.
Ac-KFMKNYC cyclic peptide 2v'. LCMS *m/z* 1022.4759 (calcd. $[M\text{+}H^{+}]$ = 1022.4587). Retention time in HPLC: 18.052 min.

HPLC trace of 1v

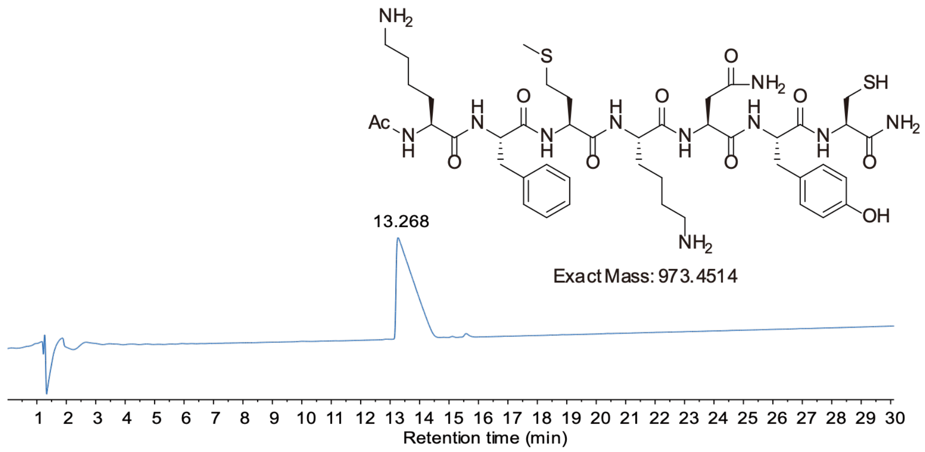

HPLC trace of reaction mixture to generate 2v and 2v'

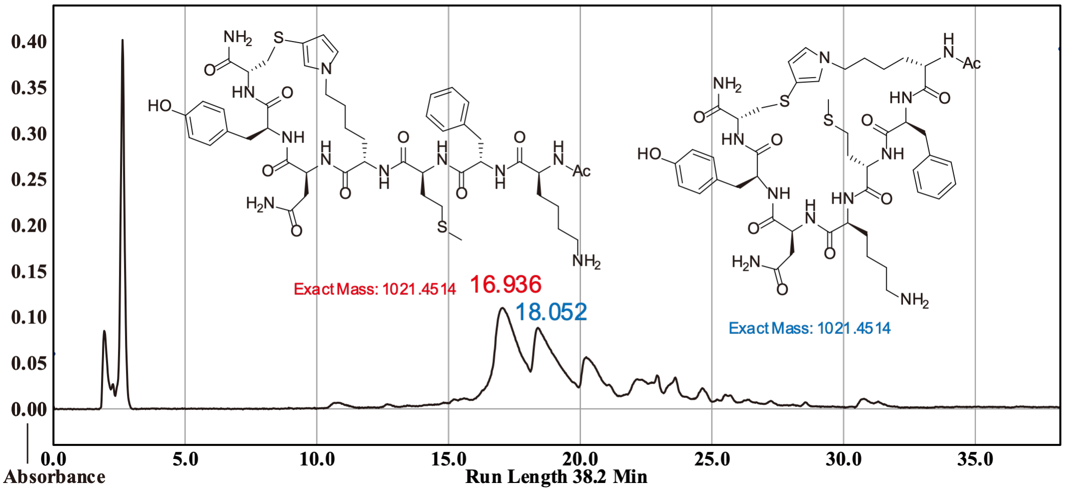

MS trace of 1v

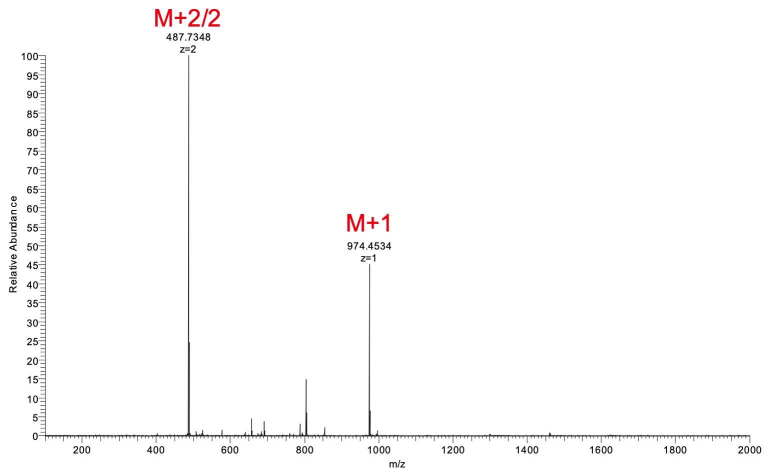

MS trace of 2v

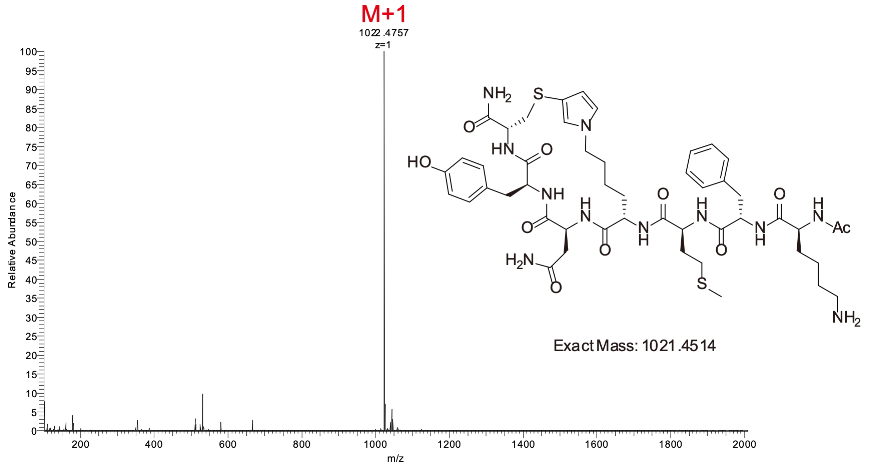

MS trace of 2v'

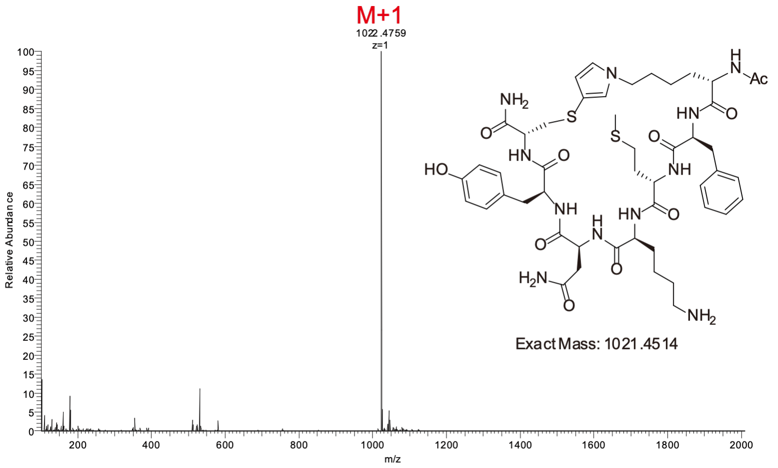

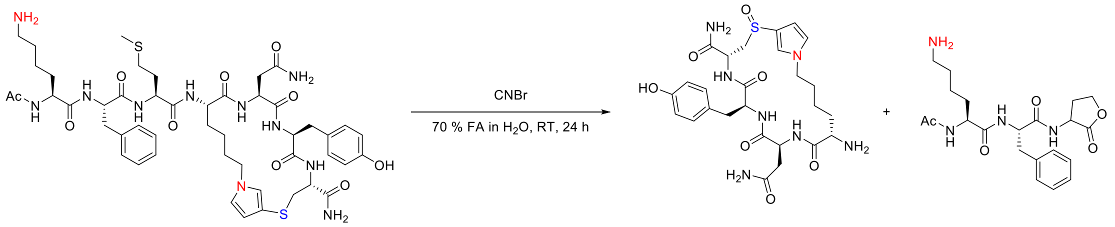

2v **C terminus fragment** **N terminus fragment**

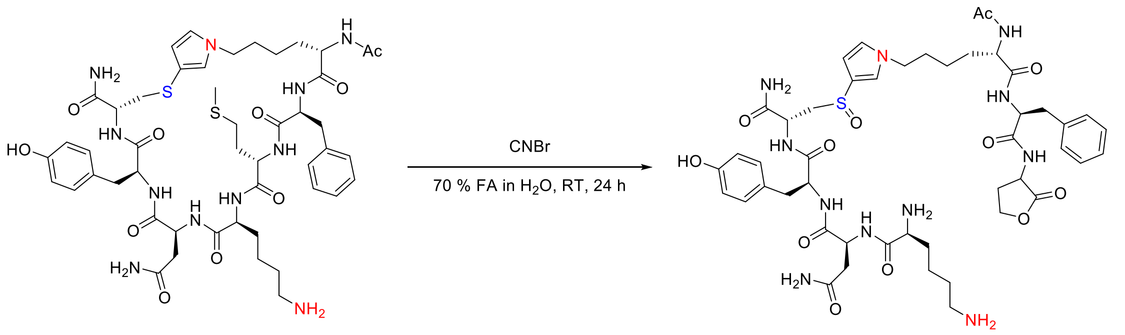

2v' **Cleavage product of 2v'**

To determine the position of cyclization, cyanogen bromide (CNBr) was used to cleave the cyclic peptide at the methionine residue. To ~0.5 mg of products **2v** and **2v’** was added ~10 mg of Cyanogen bromide (CNBr) in 500 μL of 70 % aqueous formic acid in 2 mL glass vials which were capped and allowed to stir in the dark for 24 h, before careful evaporation of the acid and HRMS analysis

N-terminal cleavage product of 2v. LCMS *m/z* 419.2387 (calcd. [M+H^+] = 419.2289).
C-terminal cleavage product of 2v’. LCMS *m/z* 590.2361 (calcd. [M+H^+] = 590.2319).
Cleavage product of 2v’. LCMS *m/z* 1008.4580 (calcd. [M+H^+] = 1008.4535), *m/z* 504.7375 (calcd. [(M+2H^+)/2] = 504.7268).

MS trace of C & N terminus fragments of 2v after cleavage

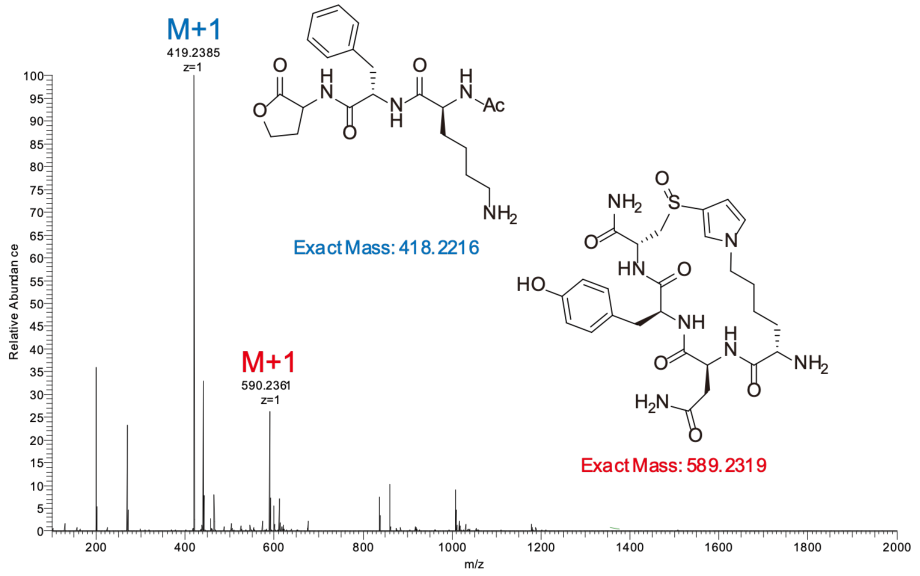

MS trace of 2v’ after cleavage

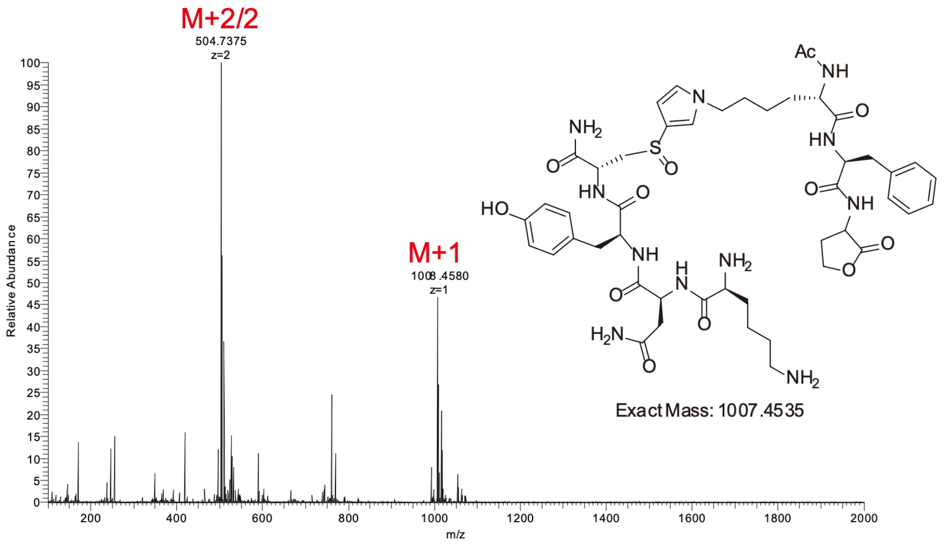

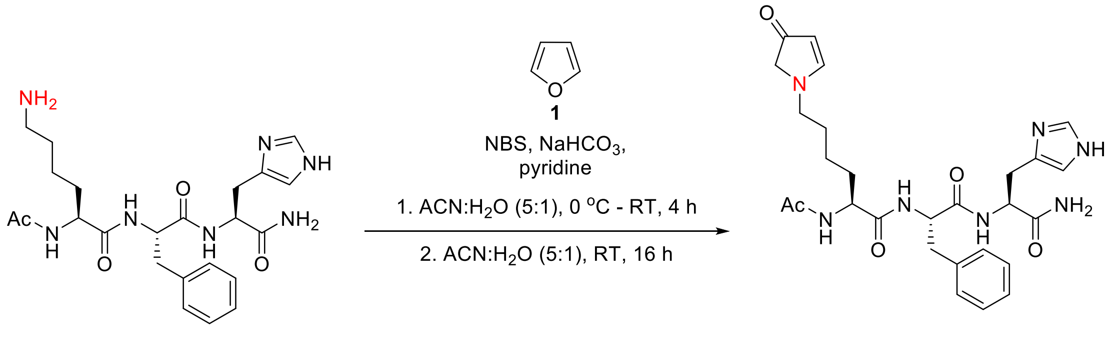

1y **2y**

A solution of furan 1 (100 µL, 1.4 mmol, 1 equiv.) and sodium bicarbonate (118 mg, 1.4 mmol, 1 equiv.) in 12 mL of ACN:H_2O (5:1) was incubated at 0 °C for 15 min. A 12 mL solution of NBS (244 mg, 1.4 mmol, 1 equiv.) in ACN:H_2O (5:1) was added to the mixture dropwise over 5 min at 0 °C and the reaction was stirred for 10 min. Pyridine (222 µL, 2.8 mmol, 2 equiv.) was then added directly and the reaction mixture was allowed to stir at 0 °C for 4 h. This afforded the oxidized intermediate cis-2-butene-1,4-dial (BDA), which was not isolated. This reaction served as a stock solution of the reactive intermediate.

From a freshly prepared stock solution of prepared cis-2-butene-1,4-dial (BDA), a 38.8 µL aliquot (2.6 µmol, 1.2 equiv.) was added to a 1.0 mL solution of ACN:H_2O (5:1) containing 1 mg of peptide **1y** (2.1 µmol, 1 equiv.). The reaction was allowed to stir at RT for 16 h. Following completion of the reaction, the solvent was removed using a centrifugal vacuum concentrator system. The product was re-dissolved in 350 µL of ACN:H_2O (5:1) and analyzed by HPLC using method B to determine the percent conversion to the products **2y**, representing a total conversion of 49 % to the pyrrol-2-one product and its hydrate.

Ac-KFH linear peptide 1y. LCMS *m/z* 472.2684 (calcd. [M+H$^+$] = 472.2667), *m/z* 263.6384 (calcd. [(M+2H$^+$)/2] = 236.637), *m/z* 943.5213 (calcd. [2M+H$^+$] = 943.5261). Purity: > 99 % (HPLC analysis at 220 nm). Retention time in HPLC: 3.711 min.

Ac-KFH linear peptide 1y. LCMS *m/z* 472.2733 (calcd. [M+H$^+$] = 472.2667, *m/z* 236.6403 (calcd. [(M+2H$^+$)/2] = 236.6370). Retention time in HPLC: 3.807 min.

Ac-KFH pyrrolin-2-one hydrate peptide 2y. LCMS *m/z* 538.2354 (calcd. [M+H$^+$] = 538.2772), m/*z* 556.2961 (calcd. [M+H_2O+H$^+$] = 556.2878). Retention time in HPLC: 9.193 min.

Ac-KFH pyrrolin-2-one peptide 2y. LCMS *m/z* 538.2850 (calcd. [M+H$^+$] = 538.2772), *m/z* 560.2668 (calcd. [M+Na$^+$] = 560.2952). Retention time in HPLC: 11.286 min.

HPLC trace of 1y

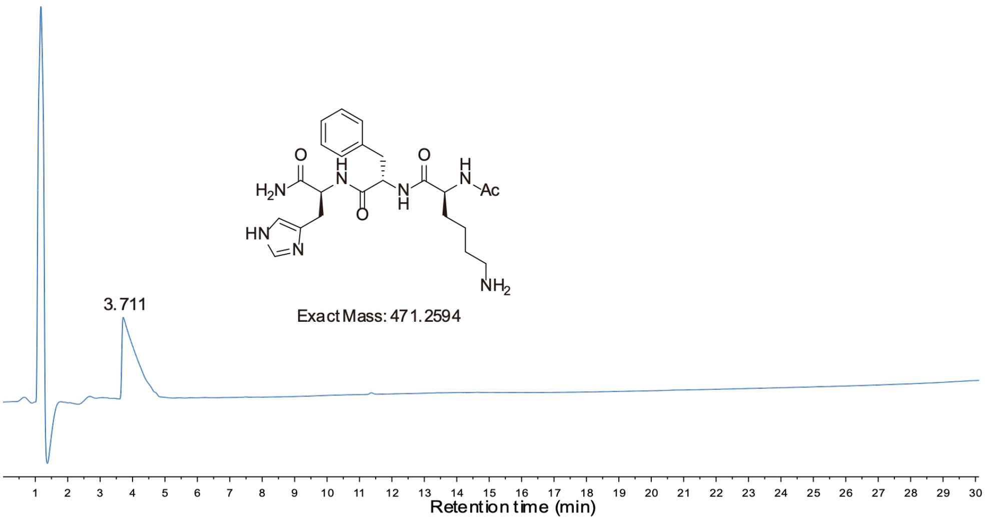

HPLC trace of reaction mixture to generate 2y

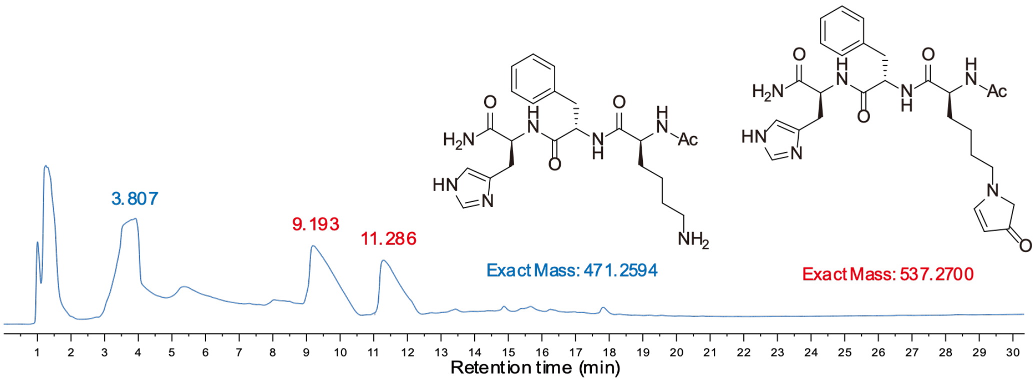

MS-trace 1y

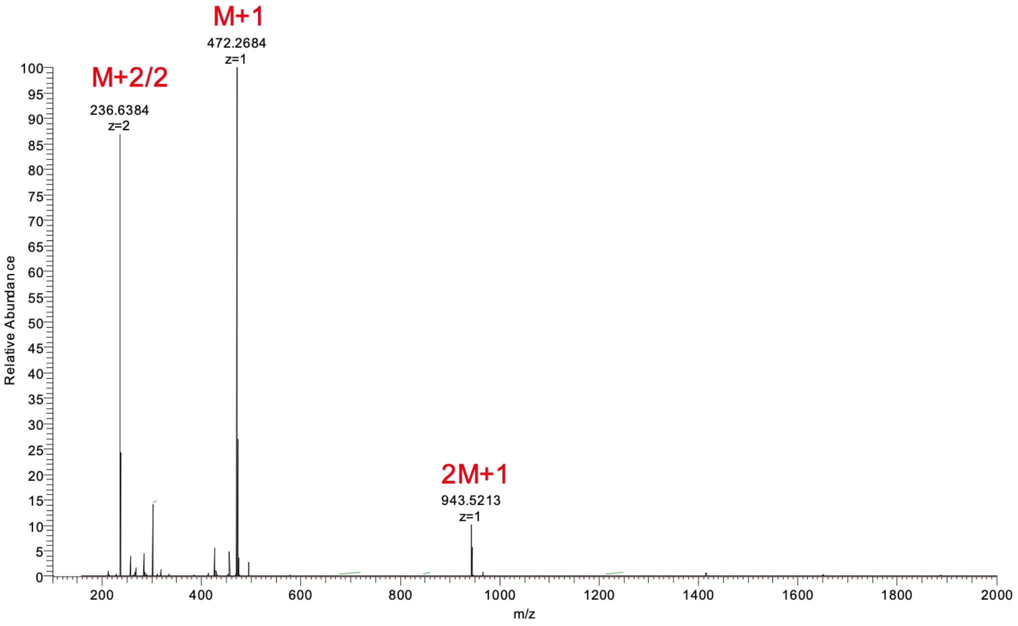

MS-trace of reaction mixture peak at 3.807 min

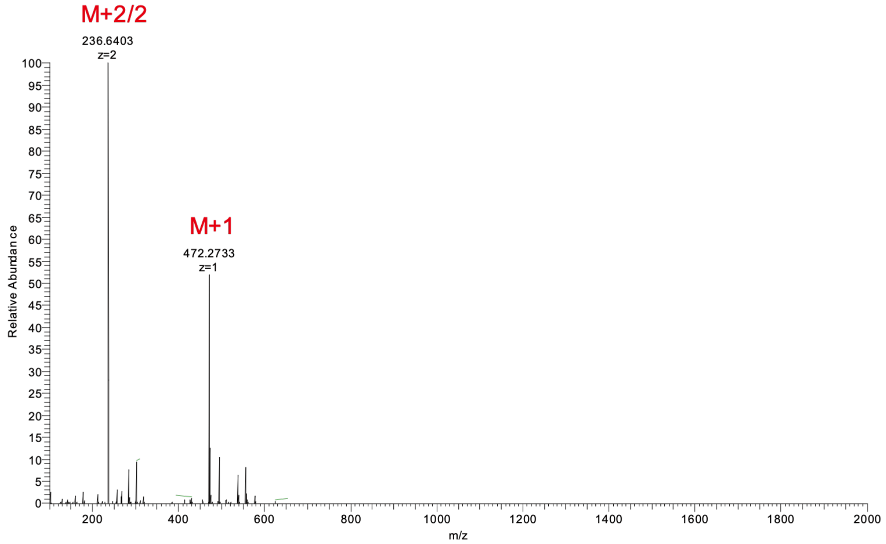

MS-trace of reaction mixture peak at 9.193 min

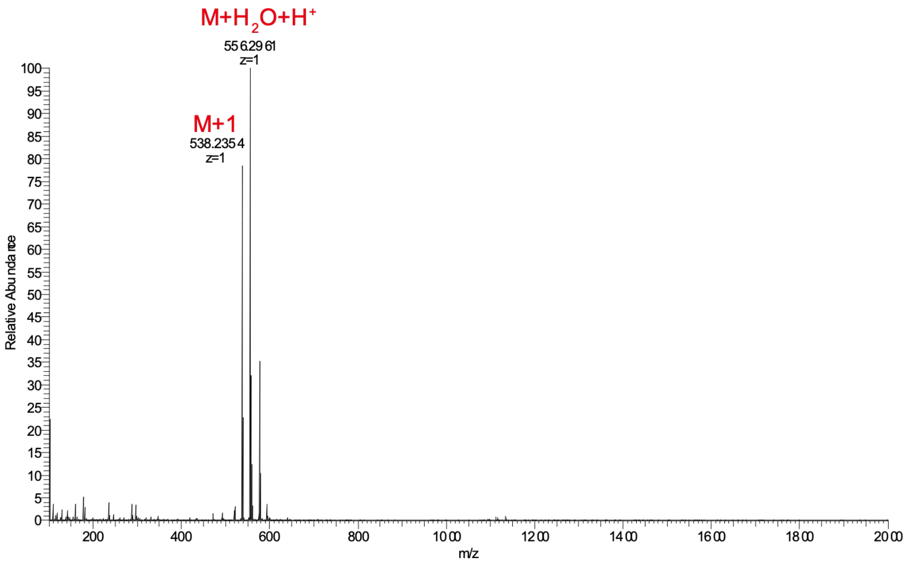

MS-trace of reaction mixture peak at 11.286 min

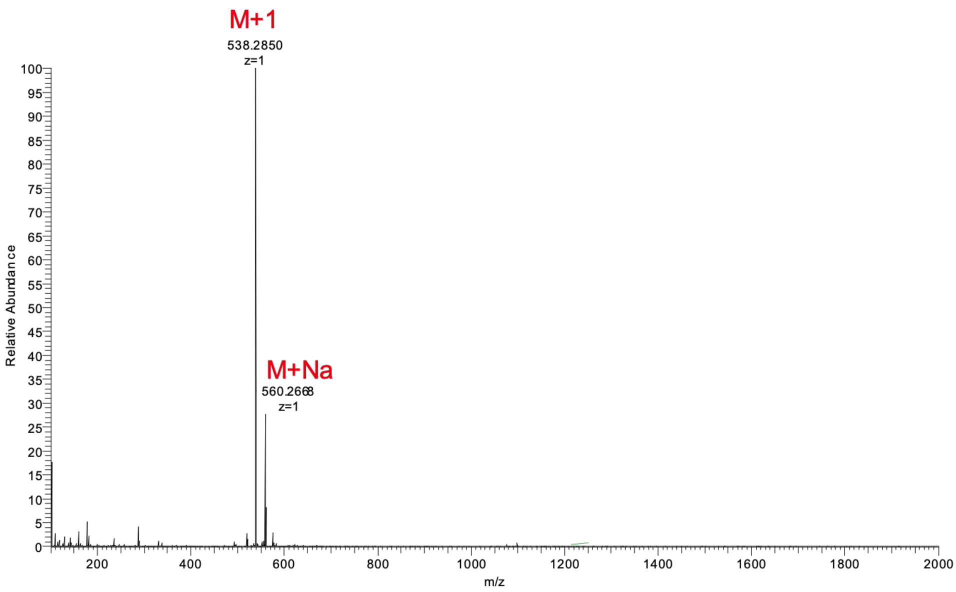

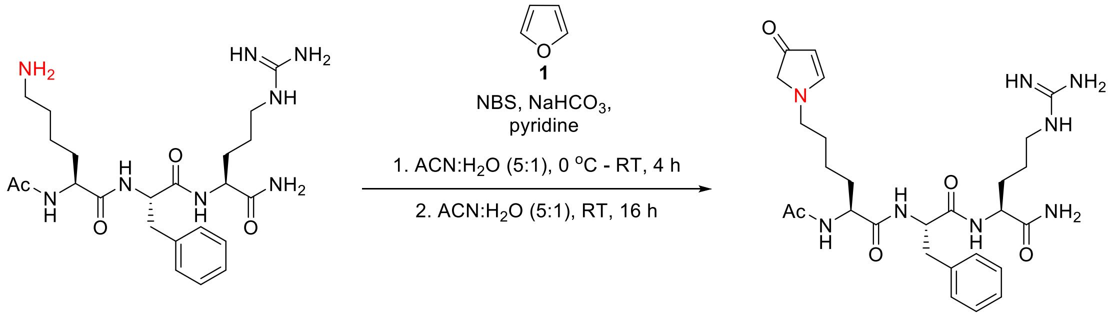

1z **2z**

A solution of furan **1** (100 µL, 1.4 mmol, 1 equiv.) and sodium bicarbonate (118 mg, 1.4 mmol, 1 equiv.) in 12 mL of ACN:H_2O (5:1) was incubated at 0 °C for 15 min. A 12 mL solution of NBS (244 mg, 1.4 mmol, 1 equiv.) in ACN:H_2O (5:1) was added to the mixture dropwise over 5 min at 0 °C and the reaction was stirred for 10 min. Pyridine (222 µL, 2.8 mmol, 2 equiv.) was then added directly and the reaction mixture was allowed to stir at 0 °C for 4 h. This afforded the oxidized intermediate *cis*-2-butene-1,4-dial (BDA), which was not isolated. This reaction served as a stock solution of the reactive intermediate.

From a freshly prepared stock solution of prepared *cis*-2-butene-1,4-dial (BDA), a 37.4 µL aliquot (2.4 µmol, 1.2 equiv.) was added to a 1.0 mL solution of ACN:H_2O (5:1) containing 1 mg of peptide **1z** (2.0 µmol, 1 equiv.). The reaction was allowed to stir at RT for 16 h. Following completion of the reaction, the solvent was removed using a centrifugal vacuum concentrator system. The product was re-dissolved in 350 µL of ACN:H_2O (5:1) and analyzed by HPLC using method B to determine the percent conversion to the products **2z**, representing a total conversion of 39.6 % to the pyrrol-2-one product and its hydrate.

Ac-KFR linear peptide 1z. LCMS *m/z* 491.3104 (calcd. [M+H$^+$] = 491.3089), *m/z* 246.1604 (calcd. [(M+2H$^+$)/2] = 246.1581). Purity: > 95 % (HPLC analysis at 220 nm). Retention time in HPLC: 4.177 min.

Ac-KFR linear peptide 1z. LCMS *m/z* 491.3159 (calcd. [M+H$^+$] = 491.3089), *m/z* 246.1616 (calcd. [(M+2H$^+$)/2] = 246.1581). Retention time in HPLC: 4.617 min.

Ac-KFR pyrrolin-2-one hydrate peptide 2z. LCMS *m/z* 557.3275 (calcd. [M+H$^+$] = 557.3183), *m/z* 575.3378 (calcd. [M+H_2O+H$^+$] = 575.3300). Retention time in HPLC: 9.702 min.

Ac-KFR pyrrolin-2-one peptide 2z. LCMS *m/z* 557.3270 (calcd. [M+H$^+$] = 557.3183). Retention time in HPLC: 11.122 min.

HPLC trace of 1z

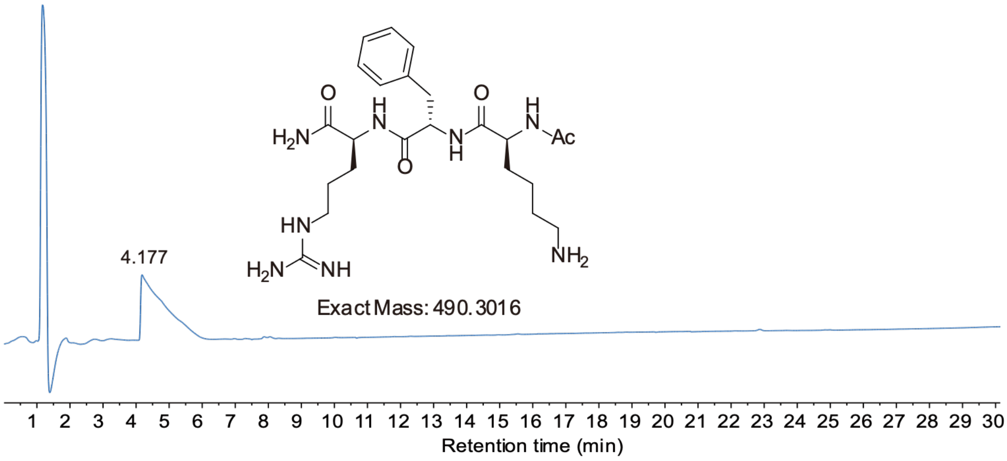

HPLC trace of reaction mixture to generate 2z

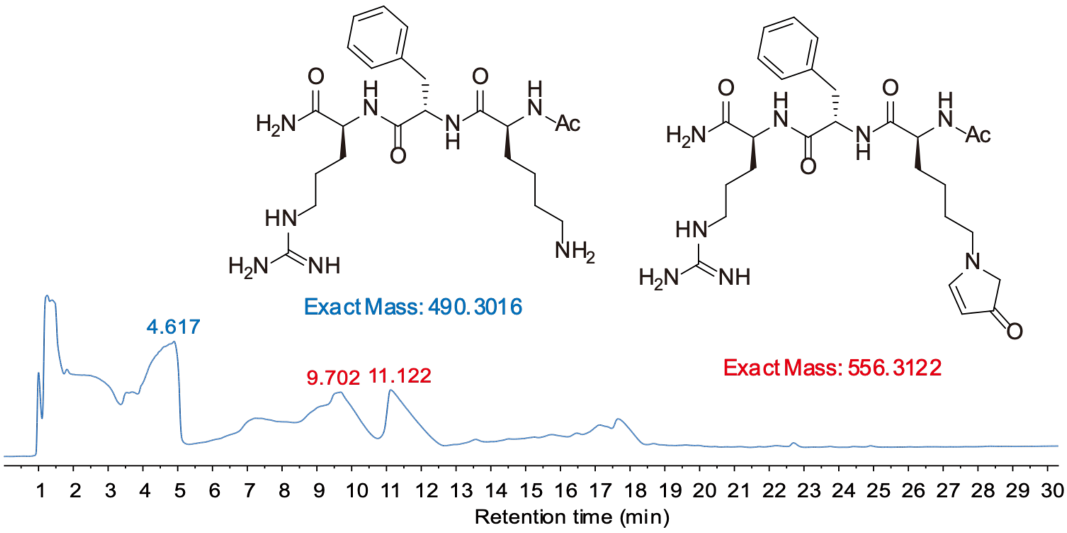

MS-trace 1z

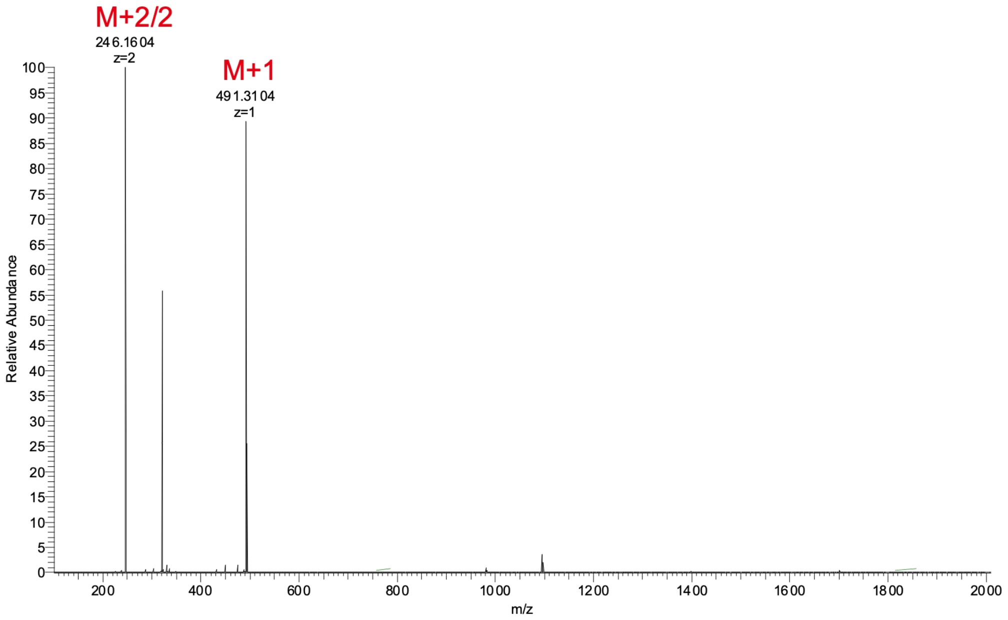

MS-trace of reaction mixture peak at 4.617 min

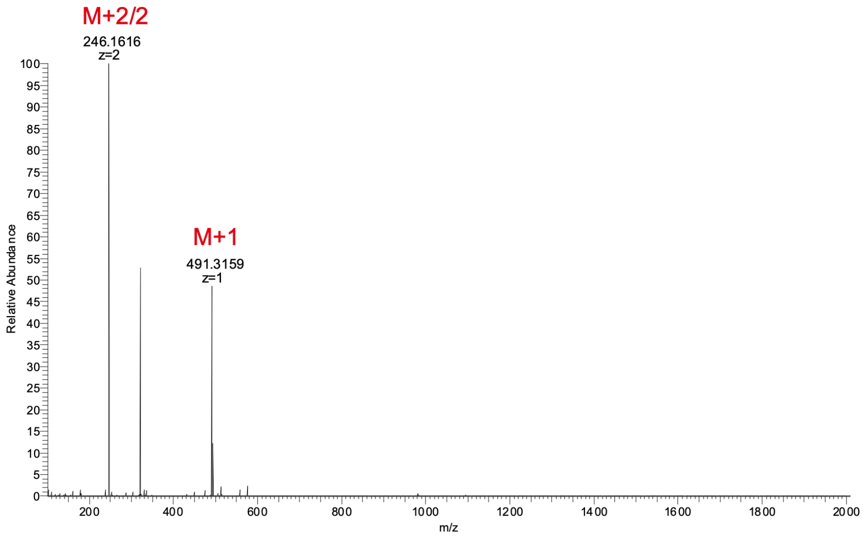

MS-trace of reaction mixture peak at 9.702 min

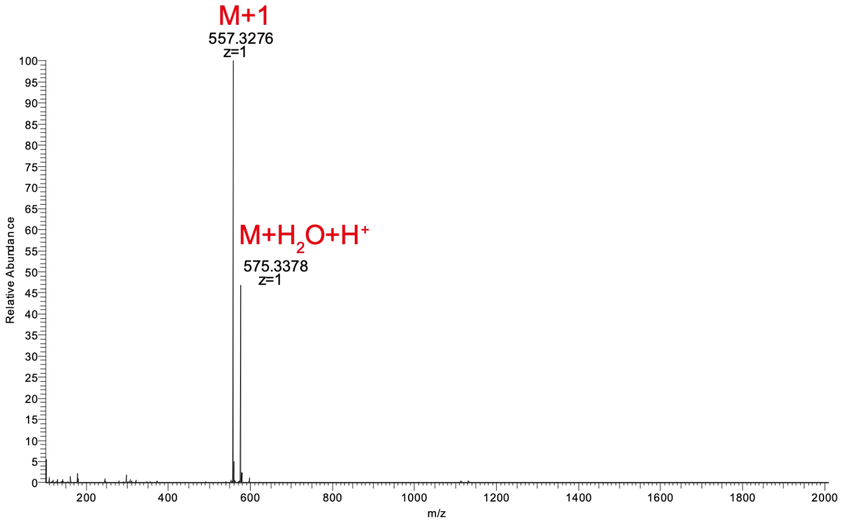

MS-trace of reaction mixture peak at 11.122 min

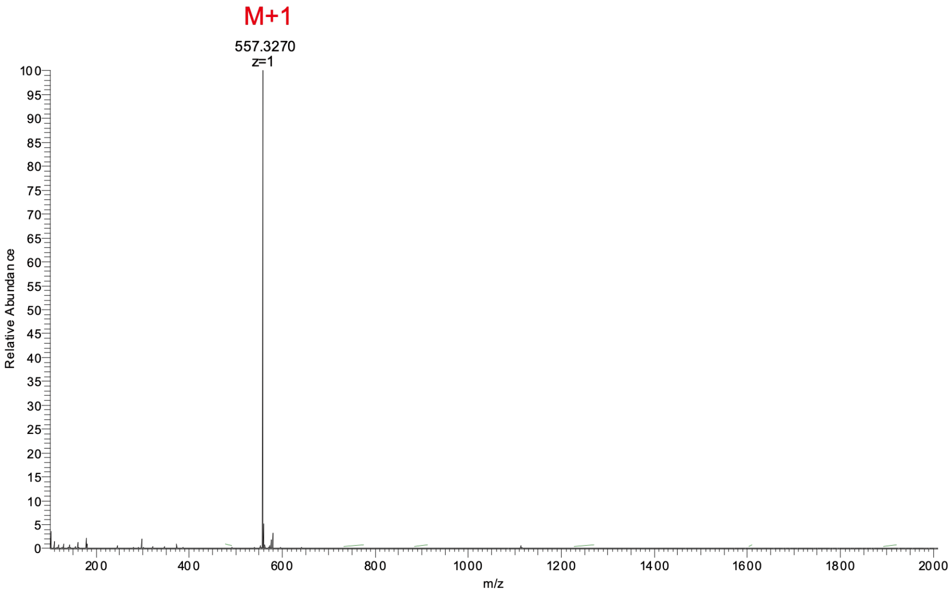

XVI. Supplementary Fig. 11. Procedure for stapling of peptides between two cysteine residues using Furan-Thiol-Amine chemistry.

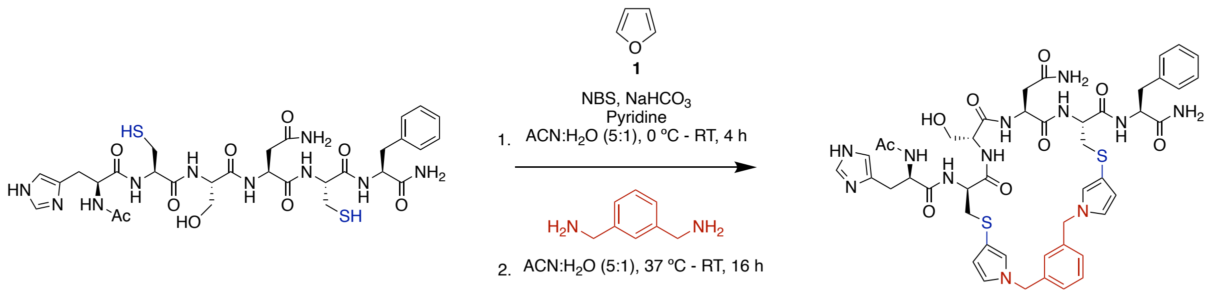

1w **2w**

F Furan **1** (100 µL, 1.38 mmol, 1 equiv.) and sodium bicarbonate (115 mg, 1.38 mmol, 1 equiv.) were added to a solution of 12 mL ACN:H_2O (5:1). The reaction mixture was cooled to 0 °C and left to stir for 15 min. N-Bromosuccinimide (244 mg, 1.38 mmol, 1 equiv.) was dissolved in a solution of 12 mL ACN:H_2O (5:1) and added to the reaction mixture dropwise. Afterward, the reaction mixture was left to stir for 10 min, and pyridine (222 µL, 2.76 mmol, 2 equiv.) was added to the reaction mixture. The reaction mixture was stirred for 4 h and used without further purification. 2.5 equiv. (15 µL) of this reaction mixture was taken directly from the pot and incubated with 0.25 mg of Ac-HCSNCF (**1u**, 1 equiv.) in 600 µL of ACN:H_2O (5:1) at 37 °C for 30 min. m-Xylenediamine (1 equiv.) was dissolved in 600 µL of ACN:H_2O (5:1) and added to the reaction mixture dropwise for a period of 30 min. The concentration of peptide in the reaction mixture was 556 µM. The reaction mixture was left to stir for 16 h at RT. The reaction mixture was analyzed by HPLC using method A to determine the percent conversion to modified product **2w** (>99 %).

Ac-HCSNCF linear peptide 1w. LCMS *m/z* 751.2598 (calcd. $[M+H]^+$ = 751.26), *m/z* 1501.5129 (calcd. $[2M+1]^+$ = 1501.52), Purity: > 95 % (HPLC analysis at 220 nm). Retention time in HPLC: 7.742 min.

Ac-HCSNCF stapled peptide 2w. LCMS *m/z* 983.3590 (calcd. $[M+H]^+$ = 983.36), Purity: > 95 % (HPLC analysis at 220 nm). Retention time in HPLC: 14.385 min.

HPLC trace of 1w

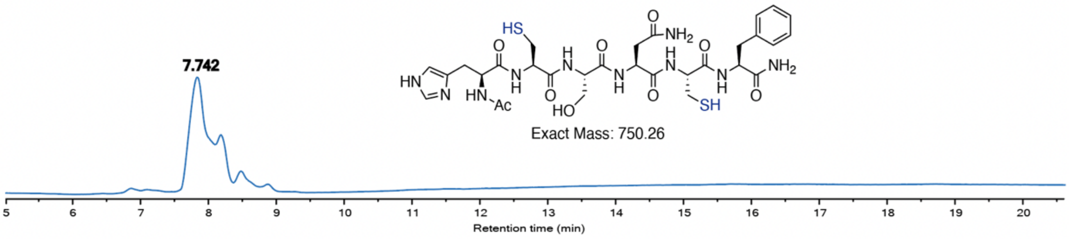

HPLC trace reaction mixture to generate 2w

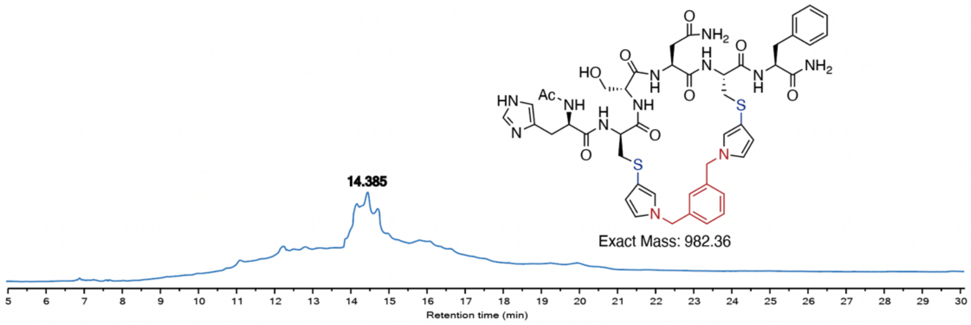

MS-trace of 1w

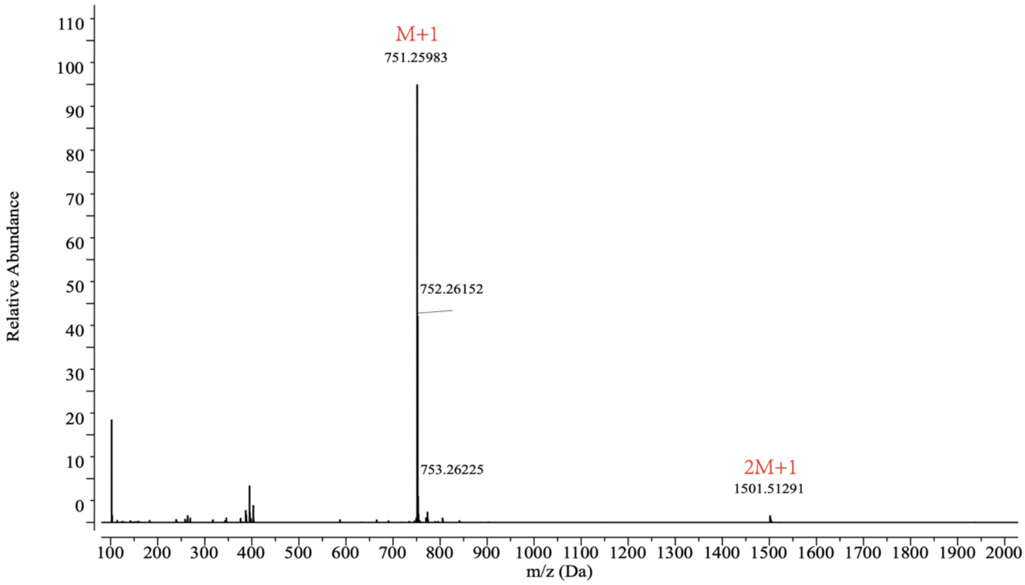

MS-trace of 2w

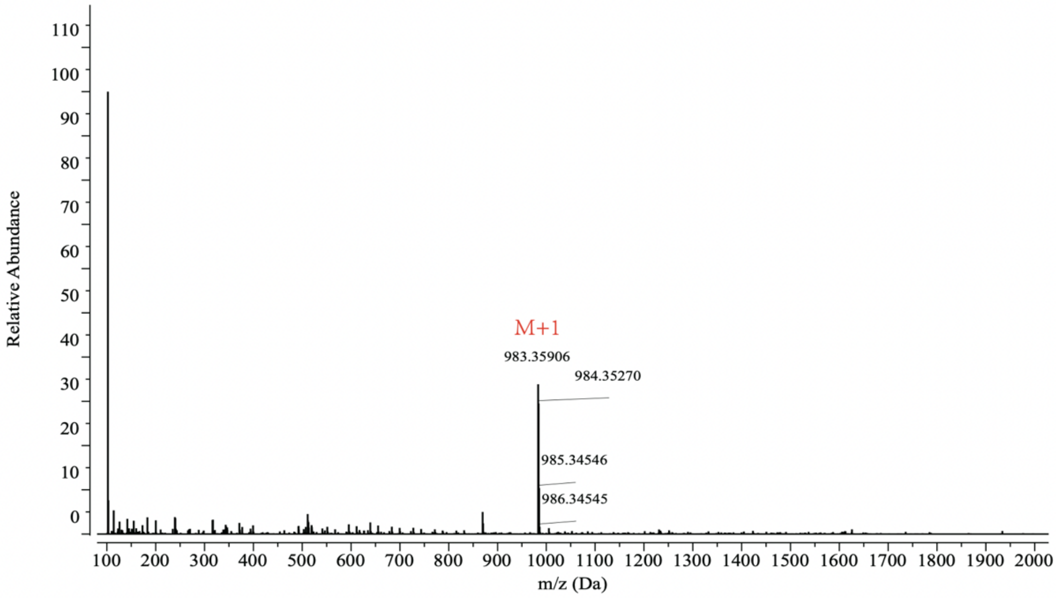

XVII. Supplementary Fig. 12. Procedure for stapling of peptides between two lysine residues using Furan-Thiol-Amine chemistry.

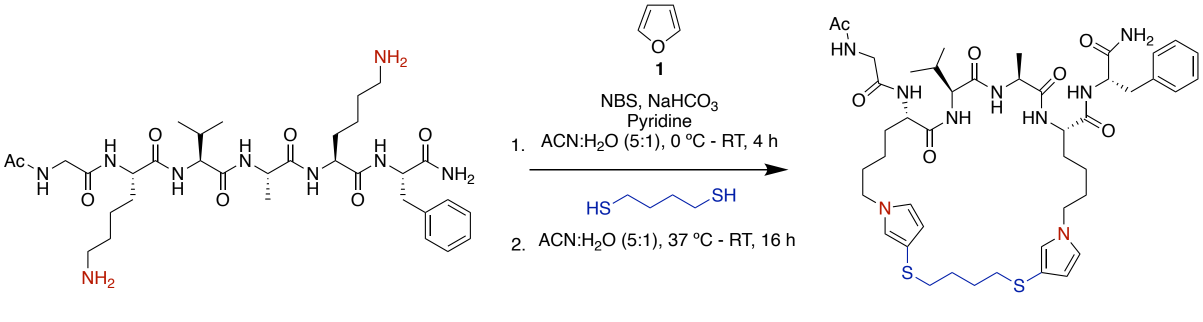

1x **2x**

Furan **1** (100 µL, 1.38 mmol, 1 equiv.) and sodium bicarbonate (115 mg, 1.38 mmol, 1 equiv.) were added to a solution of 12 mL ACN:H_2O (5:1). The reaction mixture was cooled to 0 °C and left to stir for 15 min. N-Bromosuccinimide (244 mg, 1.38 mmol, 1 equiv.) was dissolved in a solution of 12 mL ACN:H_2O (5:1) and added to the reaction mixture dropwise. Afterward, the reaction mixture was left to stir for 10 min, and pyridine (222 µL, 2.76 mmol, 2 equiv.) was added to the reaction mixture. The reaction mixture was stirred for 4 h and used without further purification. 2.5 equiv. (63 µL) of the reaction mixture was taken from the pot and incubated with 1,4-butanedithiol (1 equiv.) at 37 °C for 30 min. 1 mg of GKVAKF (**1v**, 1 equiv.) was dissolved in a 2 mL solution of ACN:H_2O (5:1) and added to the reaction mixture. The concentration of peptide in the reaction mixture was 726 µM. The reaction mixture was left to stir for 16 h at RT. The reaction mixture was analyzed by HPLC using method A to determine the percent conversion to modified product **2x** (>99 %).

Ac-GKVAKF linear peptide 1x. LCMS *m/z* 690.4249 (calcd. $[M\text{+H}]^+$ = 690.42), *m/z* 712.4065 (calcd. $[M\text{+Na}]^+$ = 712.42), *m/z* 1379.8431 (calcd. $[2M\text{+1}]^+$ = 1379.84), *m/z* 345.7161 (calcd. $[M\text{+2/2}]^+$ = 345.62), Purity: > 95 % (HPLC analysis at 220 nm). Retention time in HPLC: 6.109 min.
Ac-GKVAKF stapled peptide 2x. LCMS *m/z* 908.4461 (calcd. $[M\text{+H}]^+$ = 908.44), *m/z* 930.4276 (calcd. $[M\text{+Na}]^+$ = 930.44), Purity: > 95 % (HPLC analysis at 220 nm). Retention time in HPLC: 21.620 min.

HPLC trace of 1x

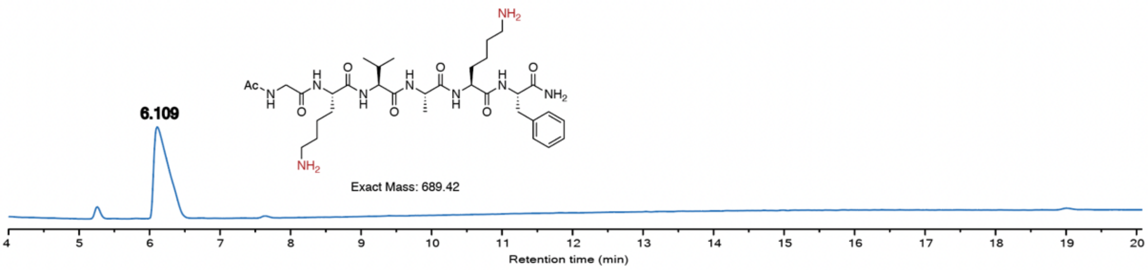

HPLC trace reaction mixture to generate 2x

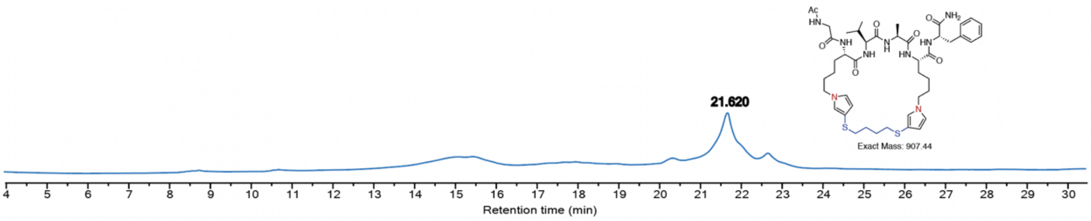

MS spectrum of 1x

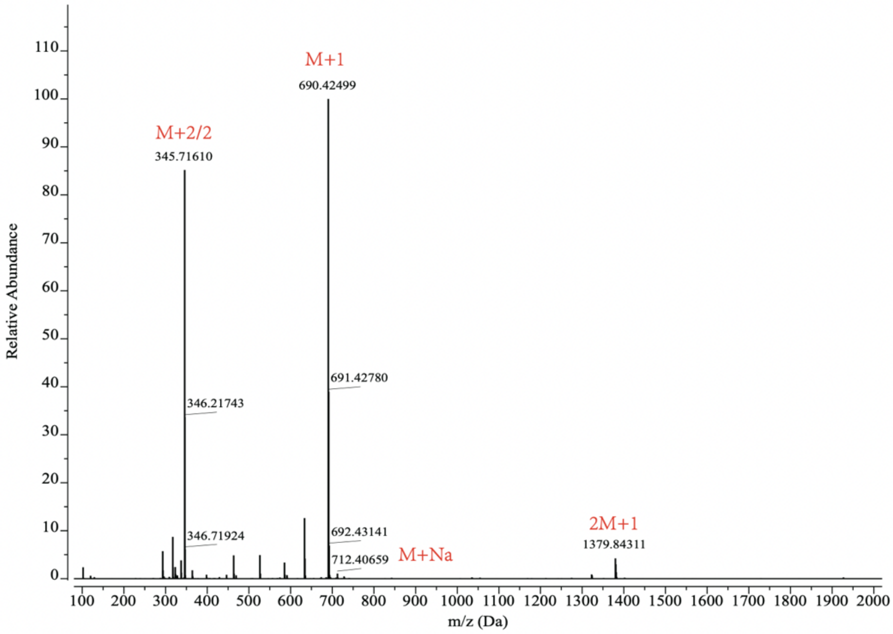

MS spectrum of 2x

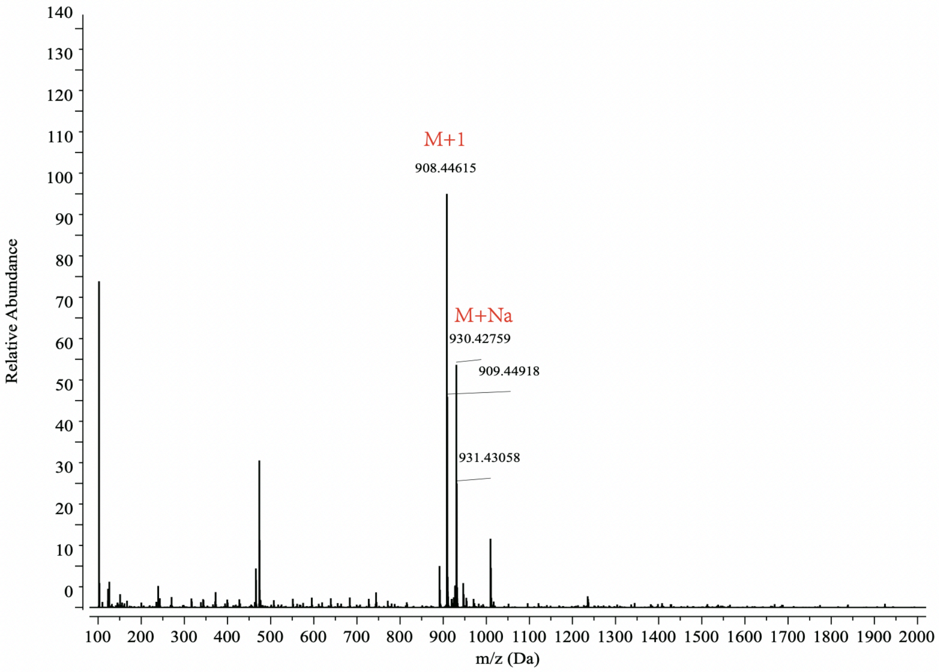

XVIII. Supplementary Fig. 13. Optimization of modification of myoglobin by Furan-Thiol-Amine multicomponent reaction.

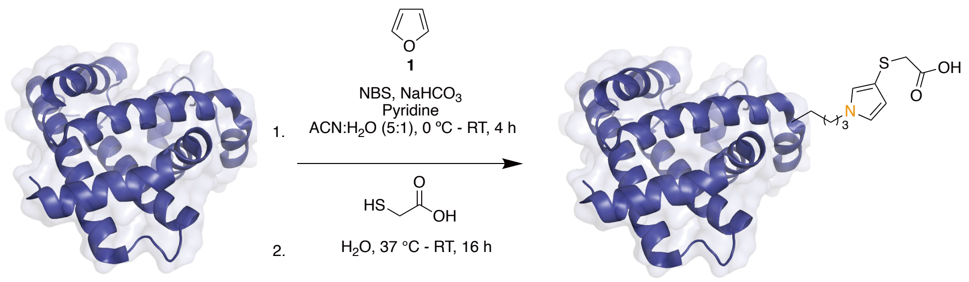

Furan **1** (100 µL, 1.38 mmol, 1 equiv.) and sodium bicarbonate (115 mg, 1.38 mmol) were added to a solution of 12 mL ACN:H_2O (5:1). The reaction mixture was cooled to 0 °C and left to stir for 15 min. N-Bromosuccinimide (244 mg, 1.38 mmol, 1 equiv.) was dissolved in a solution of 12 mL ACN:H_2O (5:1) and added to the reaction mixture dropwise. Afterward, the reaction mixture was left to stir for 10 min, and pyridine (222 µL, 2.76 mmol, 2 equiv.) was added to the reaction mixture. The reaction mixture was stirred for 4 h and used without further purification. From the pot, 1.2 equiv. (2.5 µL) of the mixture was taken and incubated with thioglycolic acid (1.2 equiv.) at 37 °C for 30 min in 1 mL of water. 2 mg of myoglobin (1 equiv.) was dissolved in 2 mL of water and added to the reaction mixture. The reaction mixture was left to stir for 16 h at RT. The reaction mixture was purified by molecular weight cut-off and characterized by LCMS to analyze the protein modification. The modification of myoglobin was repeated with varying amounts of furan and thiol reagents to identify optimized conditions for selective protein labeling. Percent conversions were calculated based on the deconvolution spectra.

Oxidized furan from pot	Thioglycolic acid	Myoglobin	Solvent	Concentration
1 equiv. (2.0 µL)	1 equiv.	2 mg	3 mL	40 µM
5 equiv. (10.2 µL)	5 equiv.	2 mg	3 mL	40 µM
15 equiv. (30.8 µL)	15 equiv.	2 mg	3 mL	40 µM

MS spectrum of unmodified myoglobin

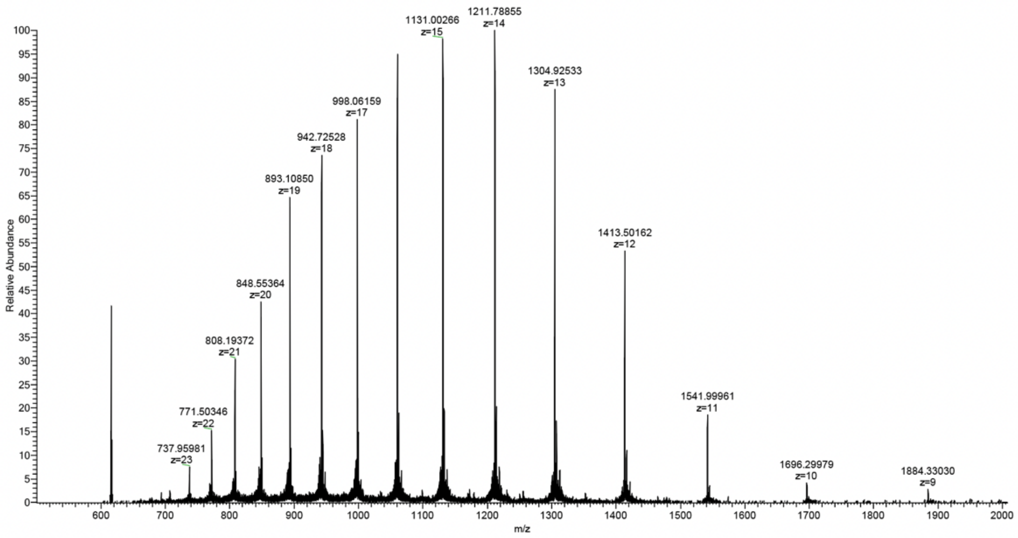

(Deconvoluted spectrum)

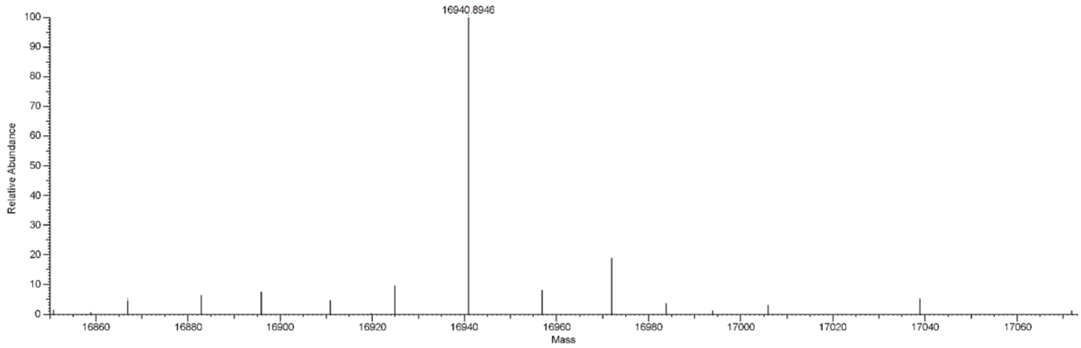

MS spectrum of myoglobin modified with 1 equiv. of oxidized furan from pot and 1 equiv. thioglycolic acid

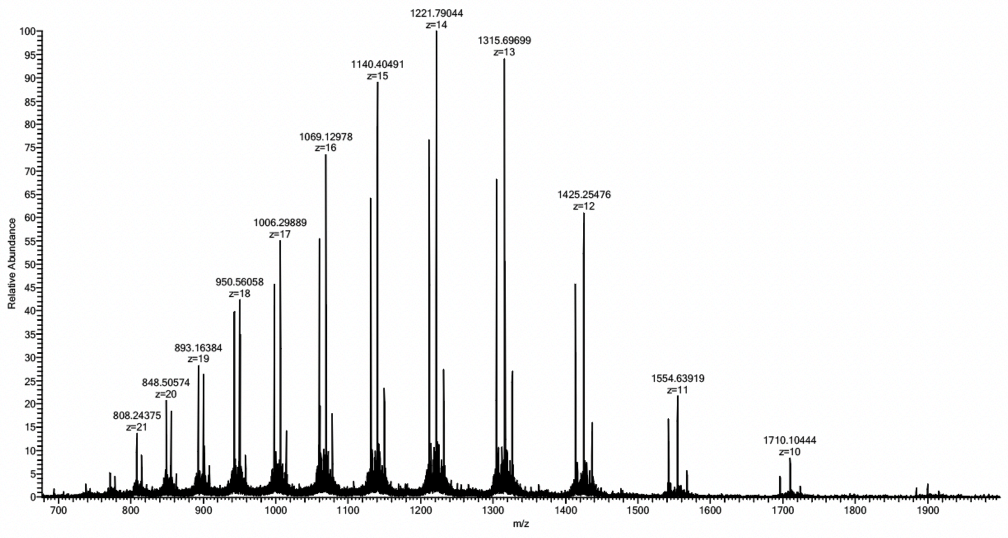

(Deconvoluted spectrum)

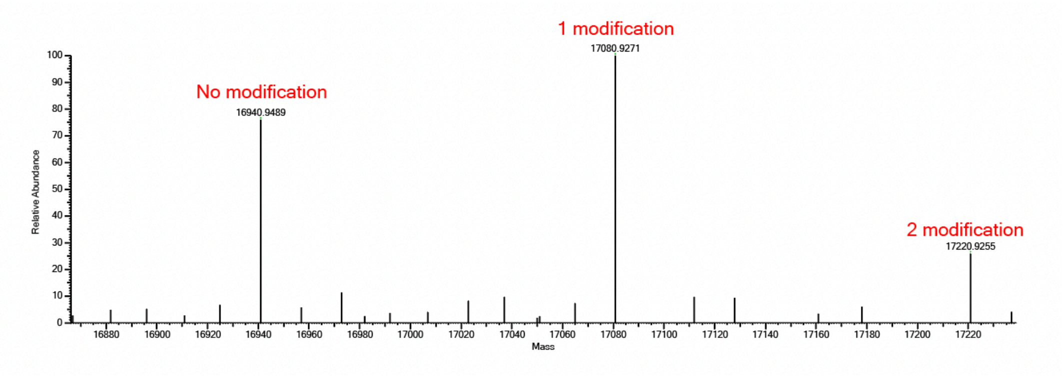

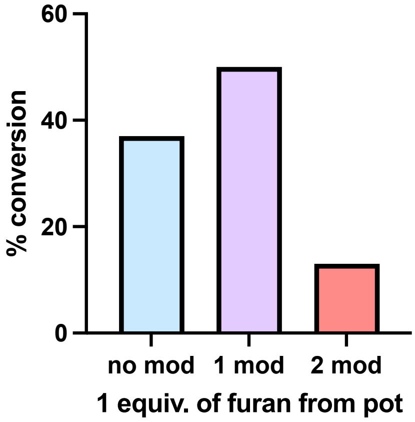

# Modifications	Expected Mass	Observed Mass	% Conversion
No modification	16940.8946	16940.9489	37
1 modification	17080.8846	17080.9271	50
2 modifications	17220.8746	17220.9255	13

Peptide fragments of modified myoglobin under 16 hours of incubation

The site of modified myoglobin, obtained by treatment with 1.2 equiv. of oxidized furan and 1.2 equiv. of thioglycolic acid was determined by Agilent Bioconfirm software after trypsin digestion using the SMART Digest™ Trypsin Kit by Thermo Scientific. The modification site was identified to be K79 and K63 (3:1). The peptide fragment shown in the figures were from AA residues 79-96 with the sequence KGHHEAELKPLAQSHATK and peptide fragment from AA residues 63-77 with the sequence KHGTVVLTALGGILK.

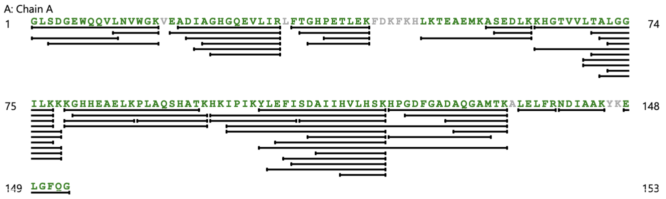

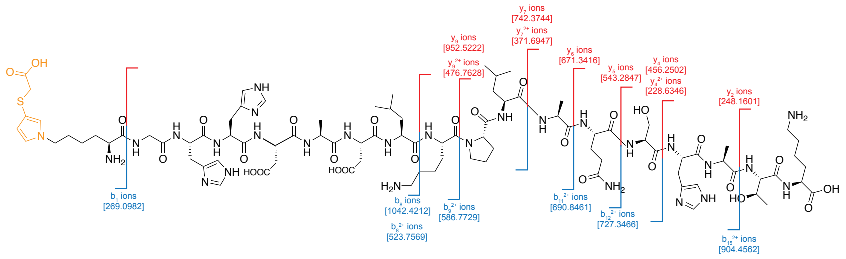

Exact Mass: 2121.04

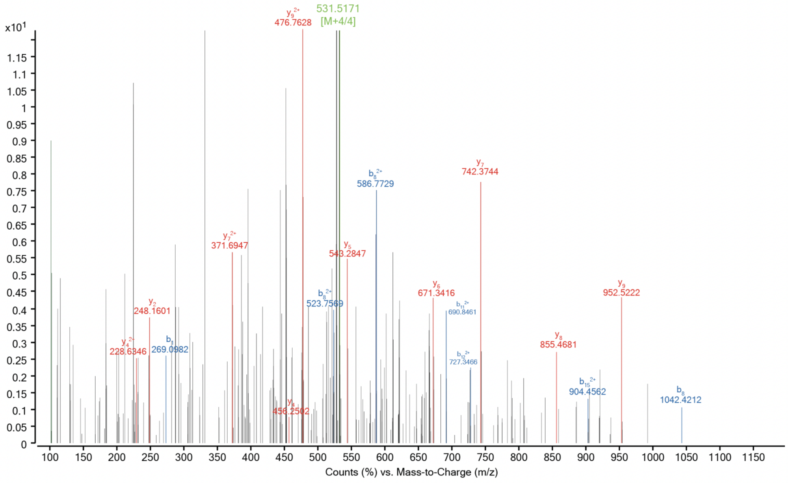

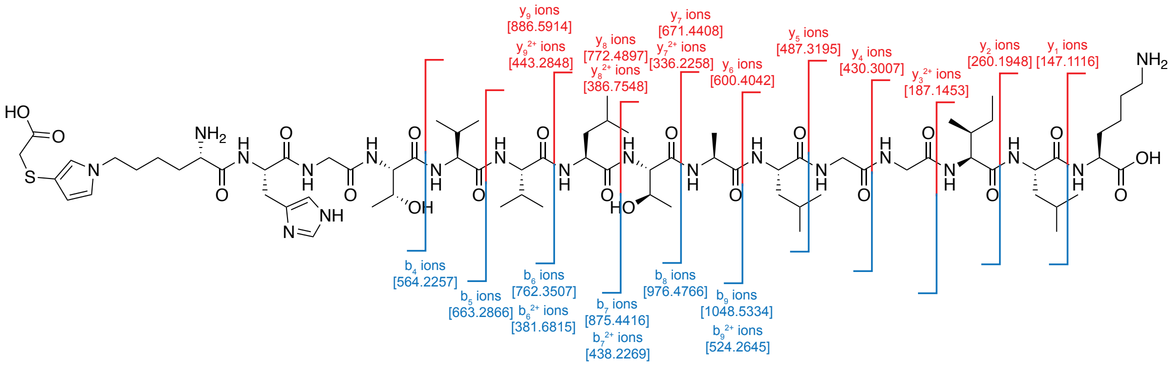

Exact Mass: 1645.92

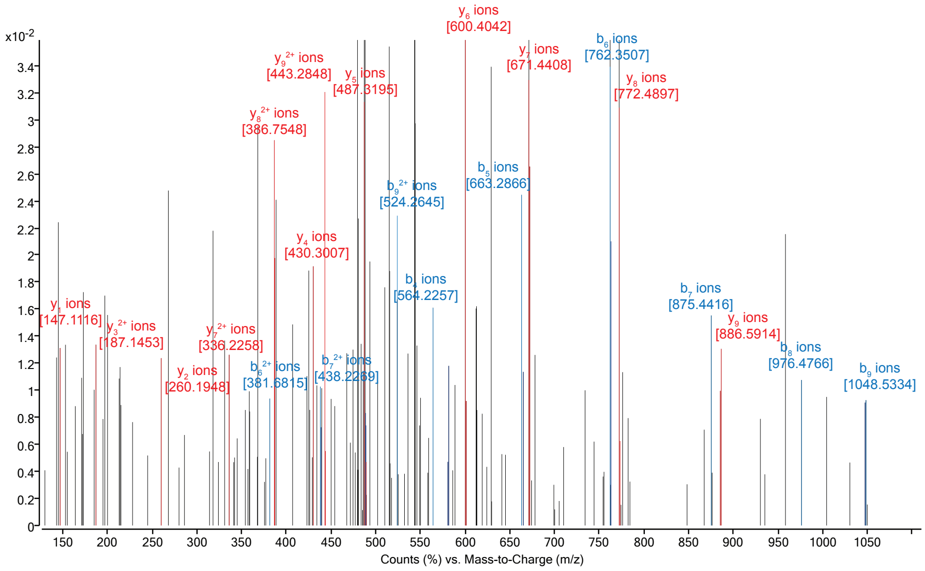

MS spectrum of myoglobin modified with 5 equiv. of oxidized furan from pot and 5 equiv. thioglycolic acid

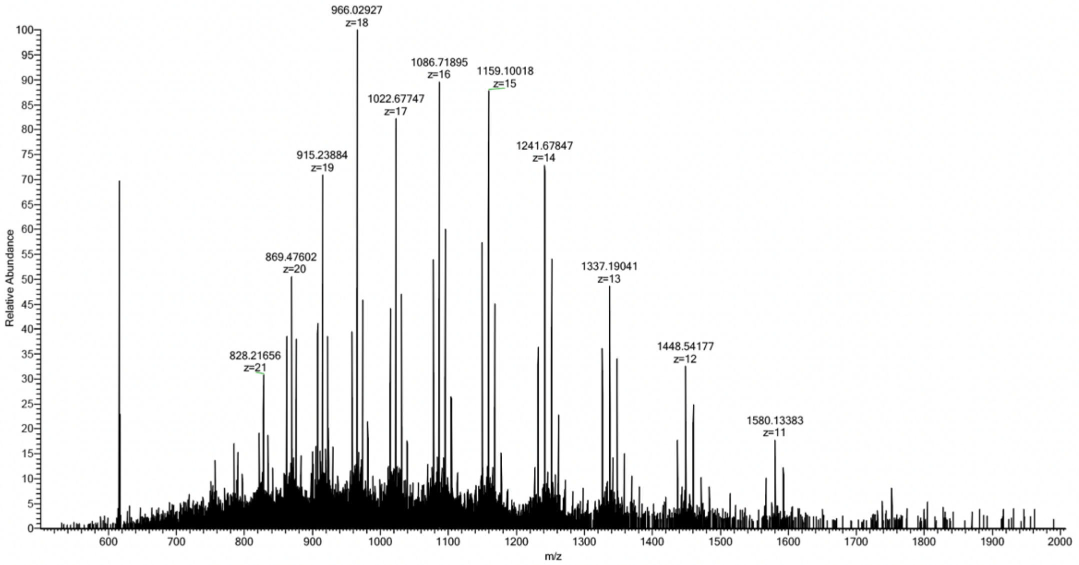

(Deconvoluted spectrum)

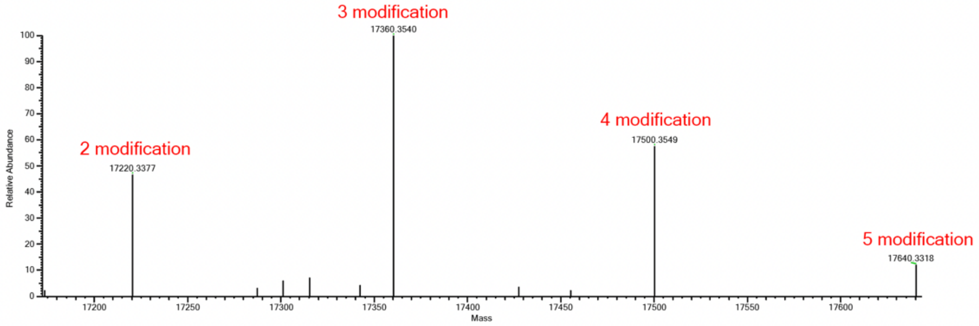

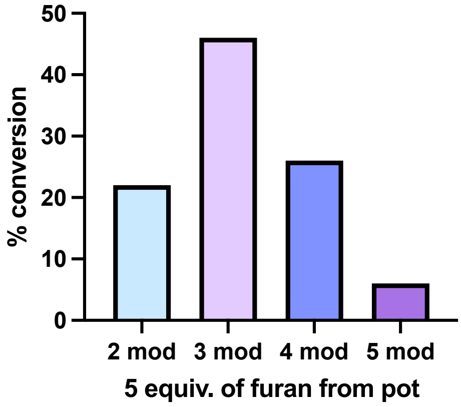

# Modifications	Expected Mass	Observed Mass	% Conversion
2 modifications	17220.8746	17220.3377	22
3 modifications	17360.8646	17360.3540	46
4 modifications	17500.8546	17500.3549	26
5 modifications	17640.8446	17640.3318	6

MS spectrum of myoglobin modified with 15 equiv. of oxidized furan from pot and 15 equiv. thioglycolic acid

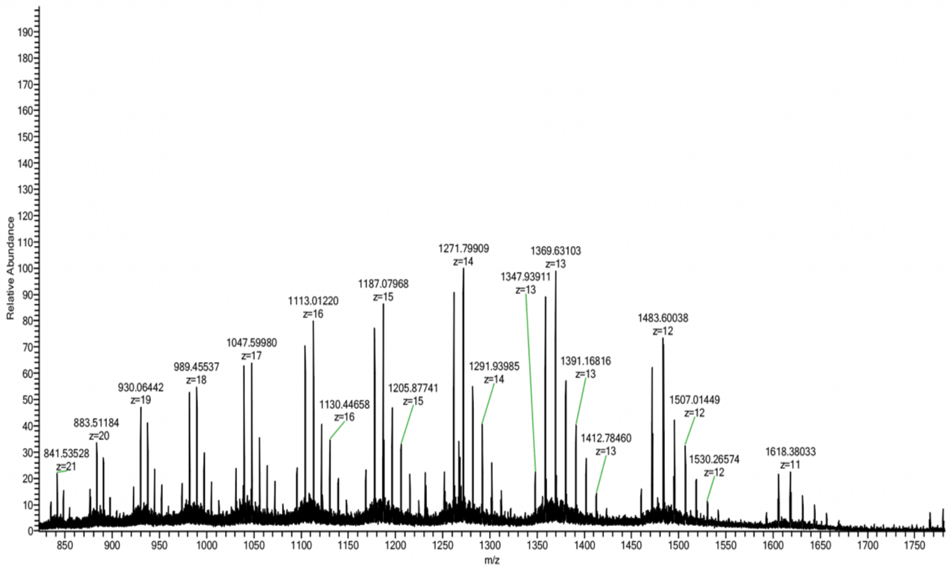

(Deconvoluted spectrum)

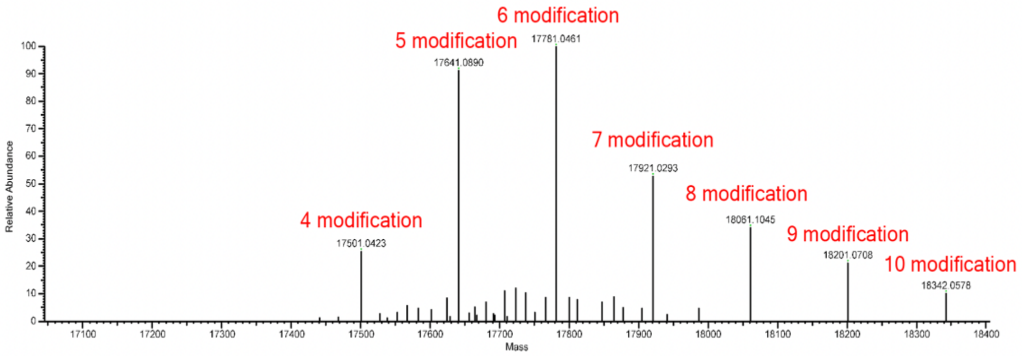

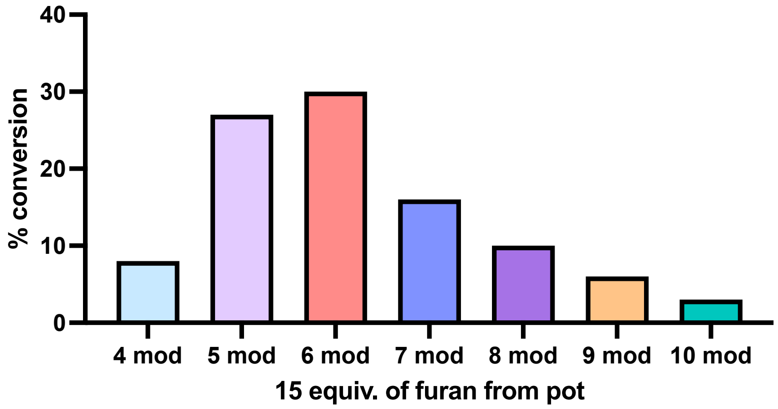

# Modifications	Expected Mass	Observed Mass	% Conversion
4 modifications	17500.8546	17501.0423	8
5 modifications	17640.8446	17641.0890	27
6 modifications	17780.8346	17781.0461	30
7 modifications	17920.8246	17921.0293	16
8 modifications	18060.8146	18061.1045	10
9 modifications	18200.8046	18201.0708	6
10 modifications	18340.7946	18342.0578	3

XIX. Supplementary Fig. 14. Stability of the modified myoglobin in different pH conditions.
Stability of modified myoglobin were assessed in different pH conditions (pH 3, pH 5, pH 9, pH 11). Labeled myoglobin (~2 mg for each reaction) obtained by using 5 equiv. of oxidized furan and thioglycolic acid was dissolved in 2 mL of buffers with different pH and were stirred for ~24 h. The reaction mixtures were purified by molecular weight cut-off and analyzed by HPLC using method A at 280 nm. We observed no shifts in the retention time of peaks.

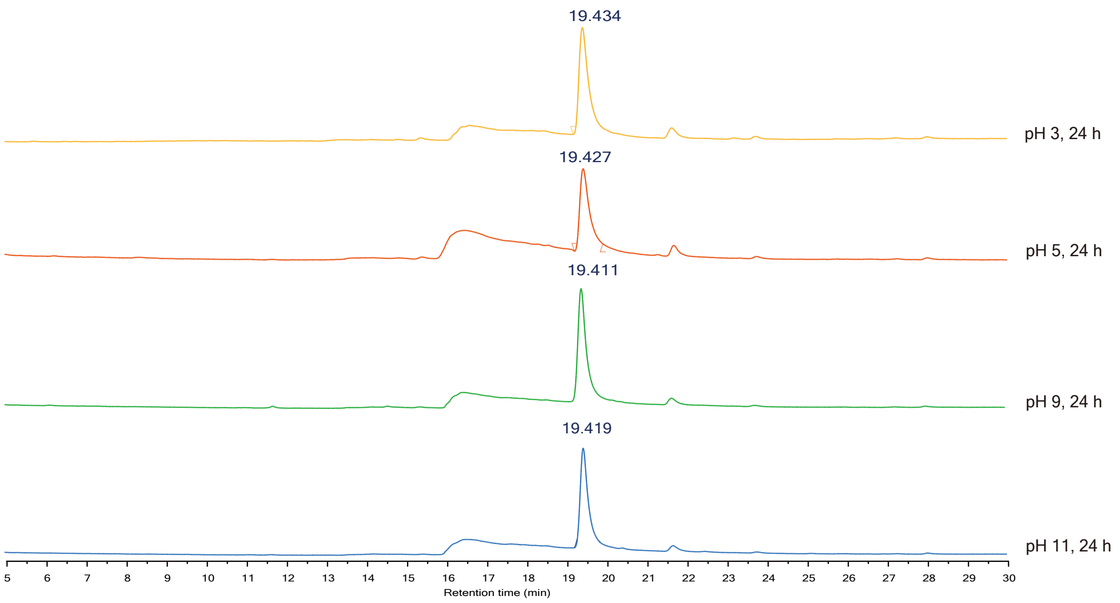

XX. Supplementary Fig. 15. Bioactivity assay of modified myoglobin in oxidation of *o*-phenylenediamine with hydrogen peroxide[2]

Enzymatic assay of myoglobin activity before and after labeling was checked by oxidation of o-phenylenediamine with hydrogen peroxide. Oxidation of *o*-phenylenediamine to 2,3-diaminophenazine was monitored at 426 nm using Agilent Cary UV-Vis Compact. Citric acid-Na_2HPO_4 buffer was prepared mixing 0.1 M citric acid and 0.2 M Na_2HPO_4. Both labeled myoglobin obtained by addition of 5 equiv. of thioglycolic acid and unlabeled native myoglobin (~2 mg) were dissolved in 2 mL of citric acid- Na_2HPO_4 buffer separately in cuvette. To the reaction mixture in cuvette, 20 μL of 0.1 M *o*-phenylenediamine and 2 μL of 1 M hydrogen peroxide were added to the

reaction mixture. The reaction was fully mixed by gentle pipetting up and down in the cuvette. The cuvettes were placed in Agilent Cary UV-Vis Compact and absorbance was measured at 426 nm every 5 min for a period of 1 h. To make a blank, 20 µL of 0.1 M *o*-phenylenediamine and 2 µL of 1 M hydrogen peroxide were added to 2 mL of citric acid- Na_2HPO_4 buffer and absorbance was measured at 426 nm every 5 min for a period of 1 h.

Sample	Amount of myoglobin	Concentration of o-phenylenediamine in experiment	Concentration of hydrogen peroxide in experiment	Total volume
Blank	0 mg	1 mM	1 mM	2 mL
Labeled	~2 mg	1 mM	1 mM	2 mL
Native	~2 mg	1 mM	1 mM	2 mL

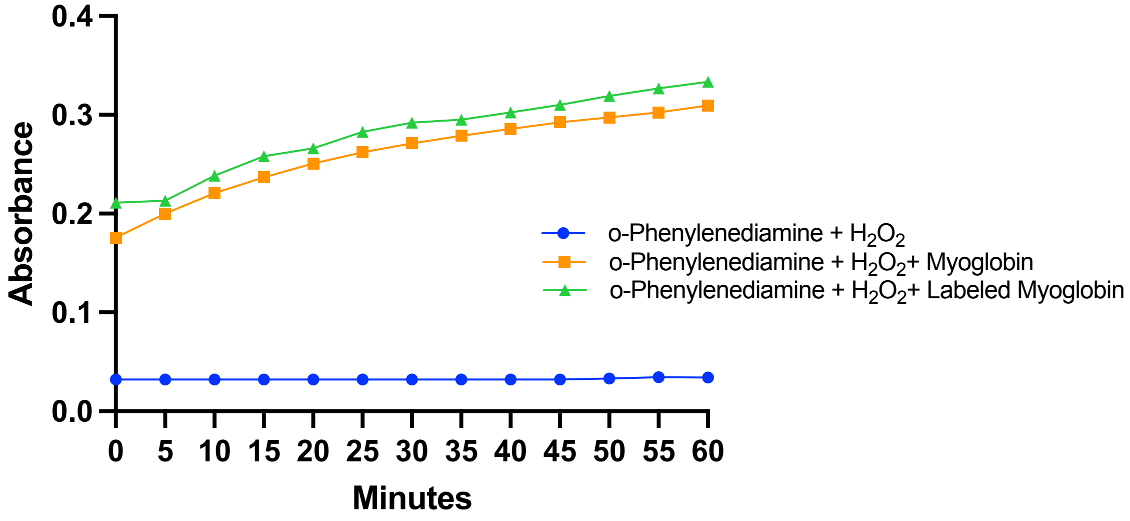

Procedure for preparing samples for Circular Dichorism: 2 mg of wild type myoglobin and modified myoglobin were dissolved in 1 mL of water. 50 µL of each were used to perform Circular Dichroism analyses. Circular Dichroism was recorded on a Jasco-810 Spectropolarimeter. Samples were micro-pipetted onto a 50 µL Hellma Analytics quartz cell with a 0.1 mm path length (Model # 106-0.10-40). Spectra were measured by averaging three scans from 260-190 nm with a 0.2 nm data pitch and 100 nm s^{-1} scanning speed.

XXI. Supplementary Fig. 16. Homogeneous labeling and determinaton the site of modification on homogeneous labeled proteins by LC-MS/MS

Homogeneous labeling of myoglobin

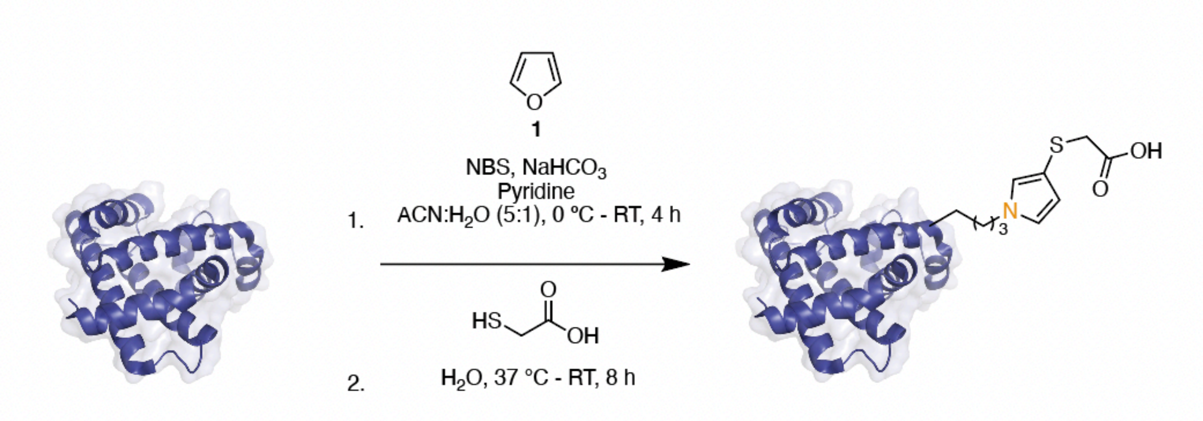

Furan **1** (100 µL, 1.38 mmol) and sodium bicarbonate (115 mg, 1.38 mmol) were added in a solution of 12 mL acetonitrile and water (5:1). The reaction mixture was cooled to 0 °C and left to stir for 15 min. N-Bromosuccinimide (244 mg, 1.38 mmol) was dissolved in a solution of 12 mL acetonitrile and water (5:1) and added to the reaction mixture dropwise. Afterwards, the reaction mixture was left to stir for 10 min, and pyridine (222 µL, 2.76 mmol) was added to the reaction mixture. The reaction mixture was stirred for 4 h and used without further purification. From the pot, 1.2 equiv. (2.5µL) of mixture was taken and incubated with thioglycolic acid (1.2 equiv.) at 37 °C for 30 min in 250 µL of water. 2 mg of myoglobin (1 equiv.) was dissolved in 250 µL of water and added to the reaction mixture (protein concentration in reaction: 236 µM). The reaction mixture was left to stir for 8 h at RT. The reaction mixture was purified by molecular weight cut off and characterized by LCMS to analyze the protein modification. Percent conversions were calculated based on the deconvolution spectra.

MS spectrum of homogenous labeled myoglobin

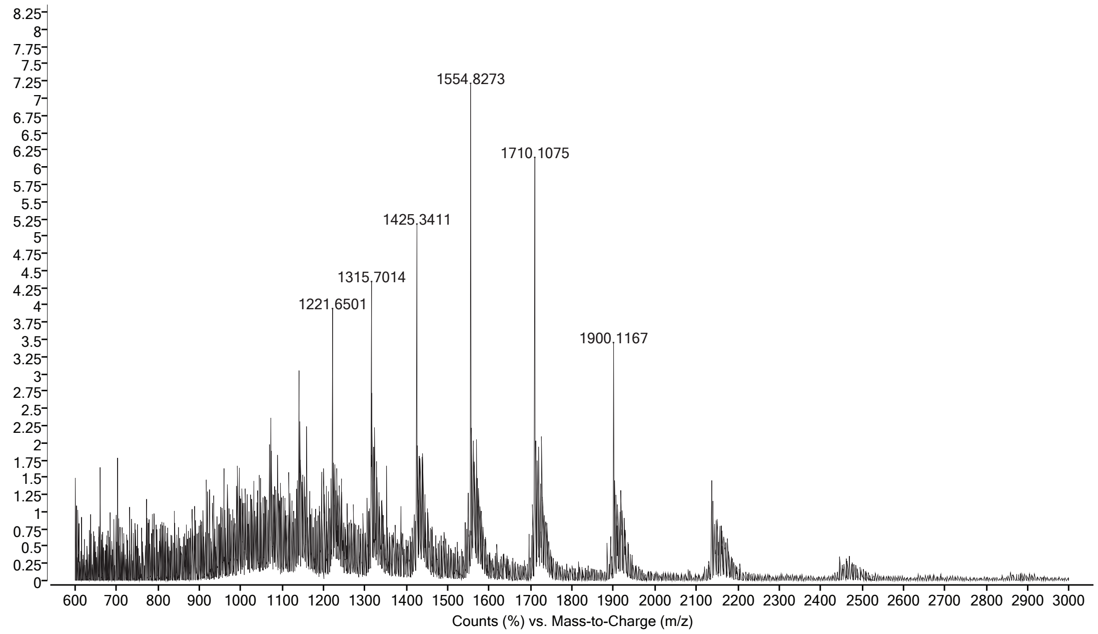

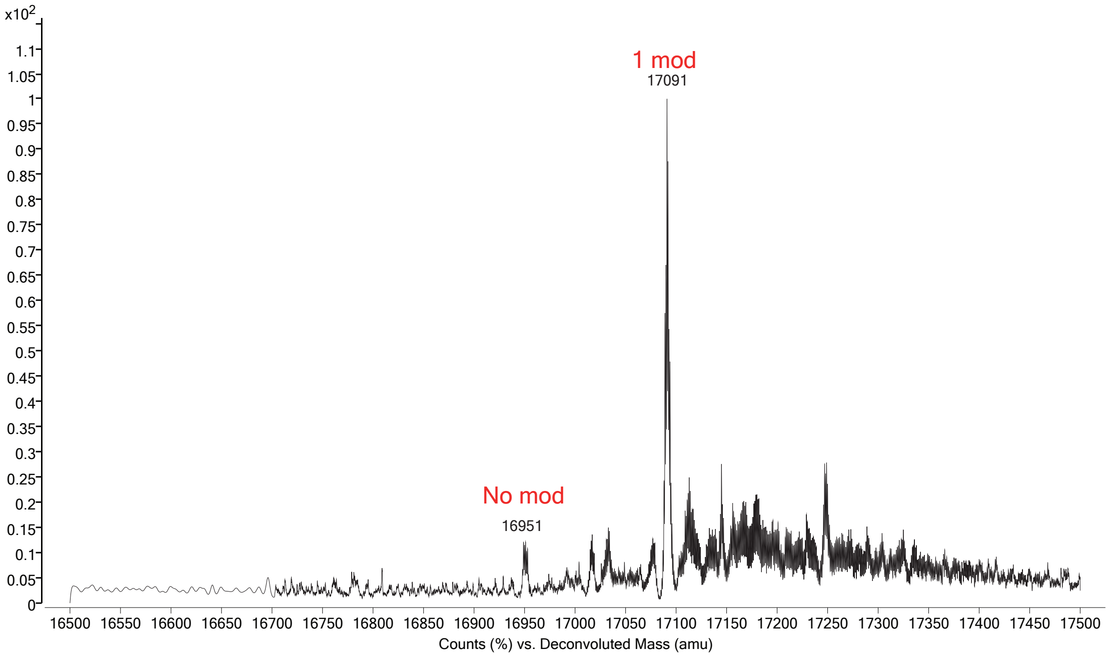

Peptide fragment of homogeneous modified myoglobin

The site of modified myoglobin, obtained by treatment with 1.2 equiv. of oxidized furan and 1.2 equiv. of thioglycolic acid in 8 h was determined by Agilent Bioconfirm software after trypsin digestion using the SMART Digest™ Trypsin Kit by Thermo Scientific. The modification site was identified to be K79. The peptide fragment shown in the figures were from AA residues 79-96 with the sequence KGHHEAELKPLAQSHATK

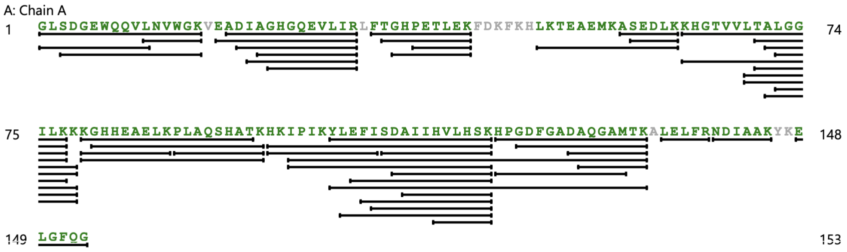

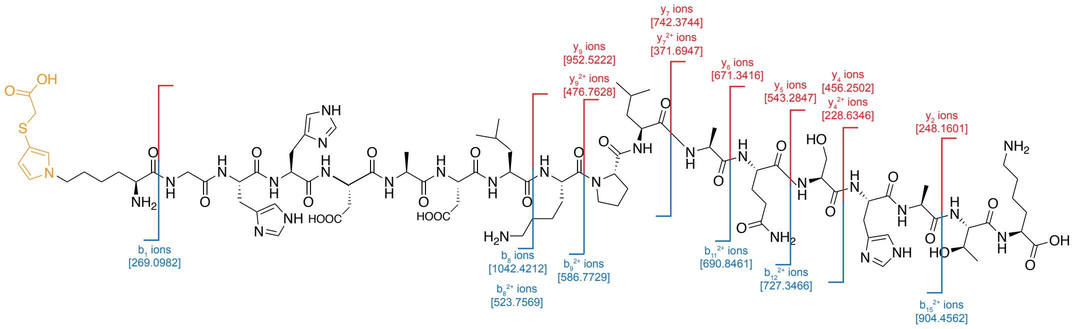

Exact Mass: 2121.04

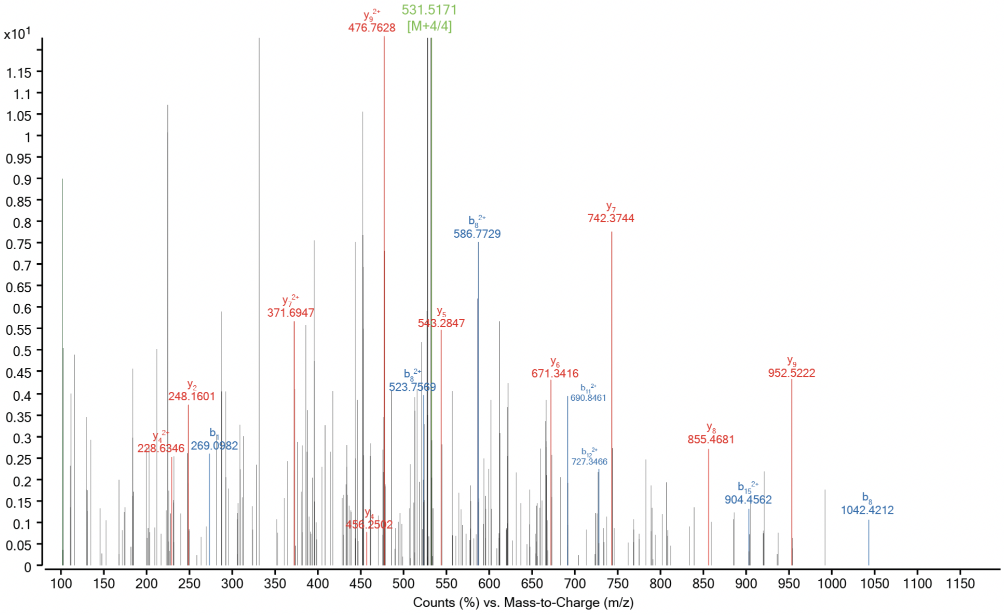

Comparison with NHSester labeling of myoglobin

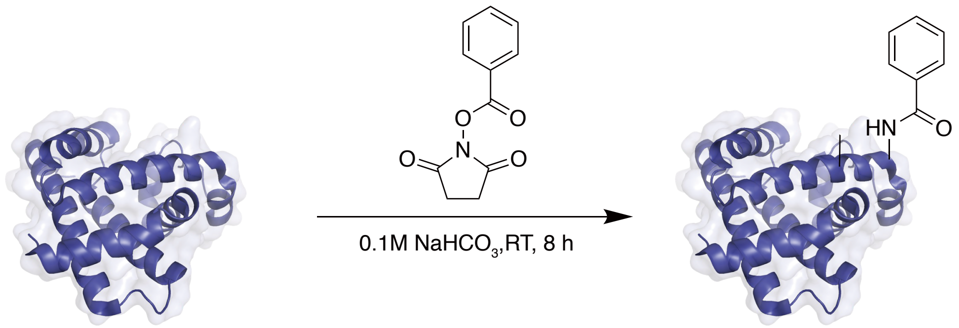

1.2 equiv. of 2,5-dioxopyrrolidin-1-yl benzoate was incubated with 2 mg of myoglobin (1 equiv.) dissolved in 0.1 M $NaHCO_3$ solution. The reaction mixture was left to stir for 8 h at RT. The reaction mixture was purified by molecular weight cut off and characterized by LCMS to analyze the protein modification. Percent conversions were calculated based on the deconvolution spectra.

MS spectrum of NHSester labeled myoglobin

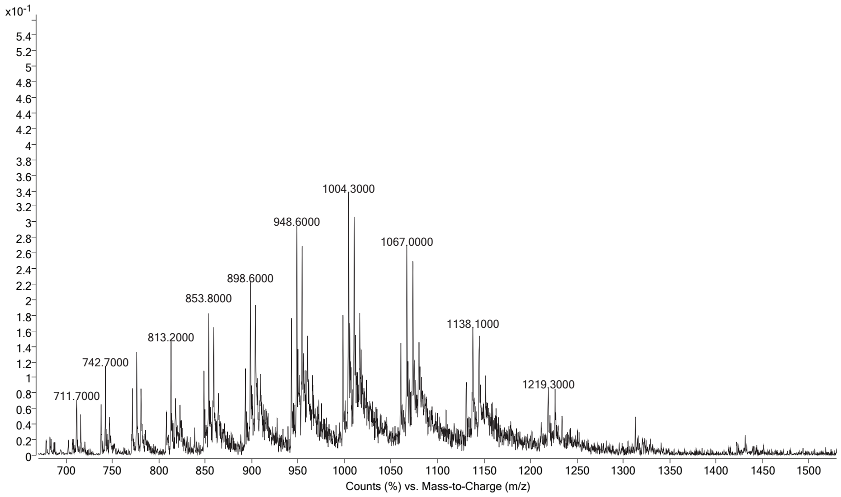

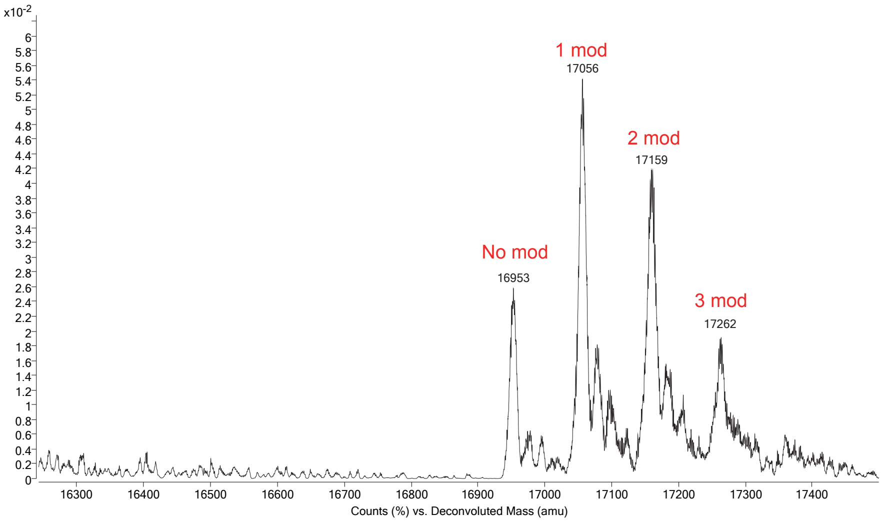

Homogeneous labeling of cytochrome C

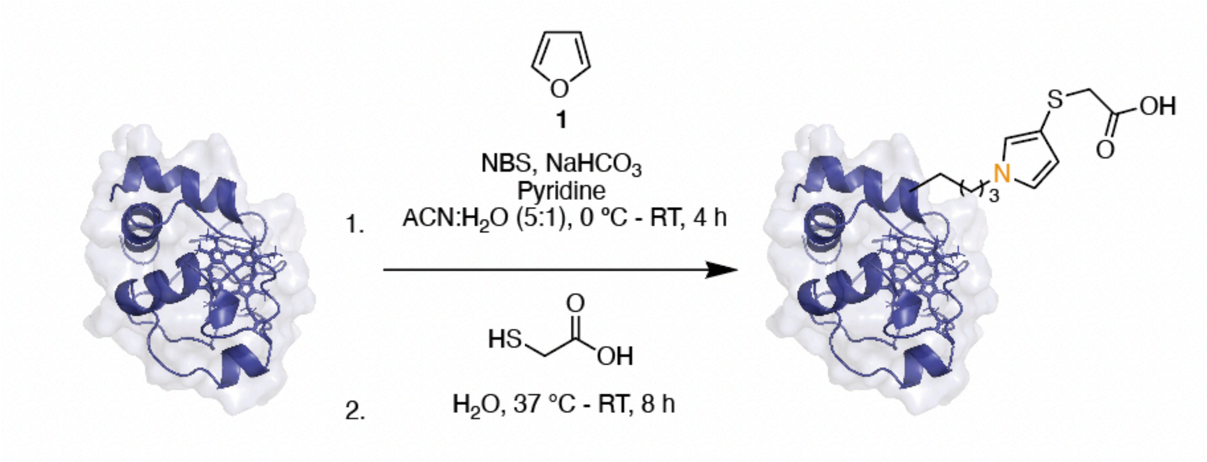

Furan **1** (100 µL, 1.38 mmol) and sodium bicarbonate (115 mg, 1.38 mmol) were added in a solution of 12 mL acetonitrile and water (5:1). The reaction mixture was cooled to 0 °C and left to stir for 15 min. N-Bromosuccinimide (244 mg, 1.38 mmol) was dissolved in a solution of 12 mL acetonitrile and water (5:1) and added to the reaction mixture dropwise. Afterwards, the reaction mixture was left to stir for 10 min, and pyridine (222 µL, 2.76 mmol) was added to the reaction mixture. The reaction mixture was stirred for 4 h and used without further purification. From the pot, 1.2 equiv. (3.3µL) of mixture was taken and incubated with thioglycolic acid (1.2 equiv.) at 37 °C for 30 min in 250 µL of water. 2 mg of cytochrome C (1 equiv.) was dissolved in 250 µL of water and added to the reaction mixture (protein concentration in reaction mixture: 324 µM). The reaction mixture was left to stir for 8 h at RT. The reaction mixture was purified by molecular weight cut off and characterized by LCMS to analyze the protein modification. Percent conversions were calculated based on the deconvolution spectra.

MS spectrum of homogeneous labeled cytochrome C

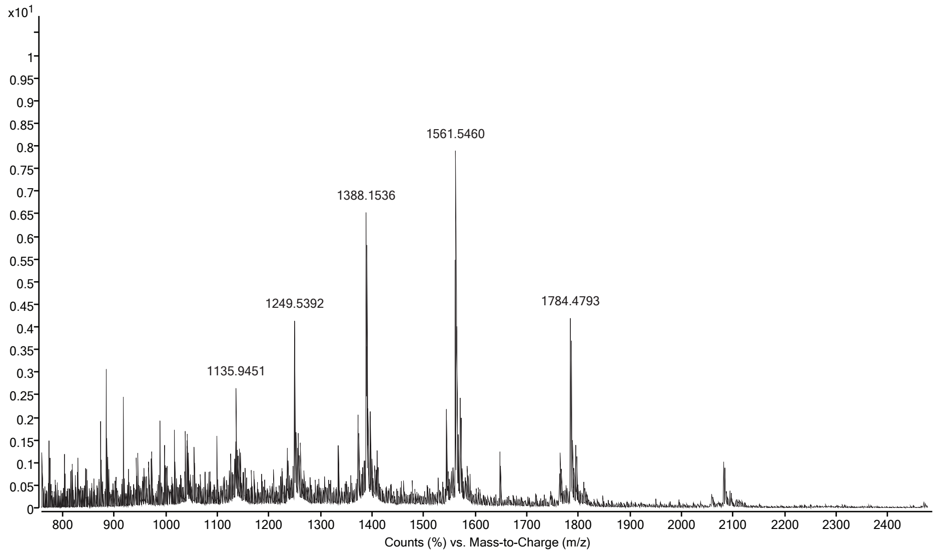

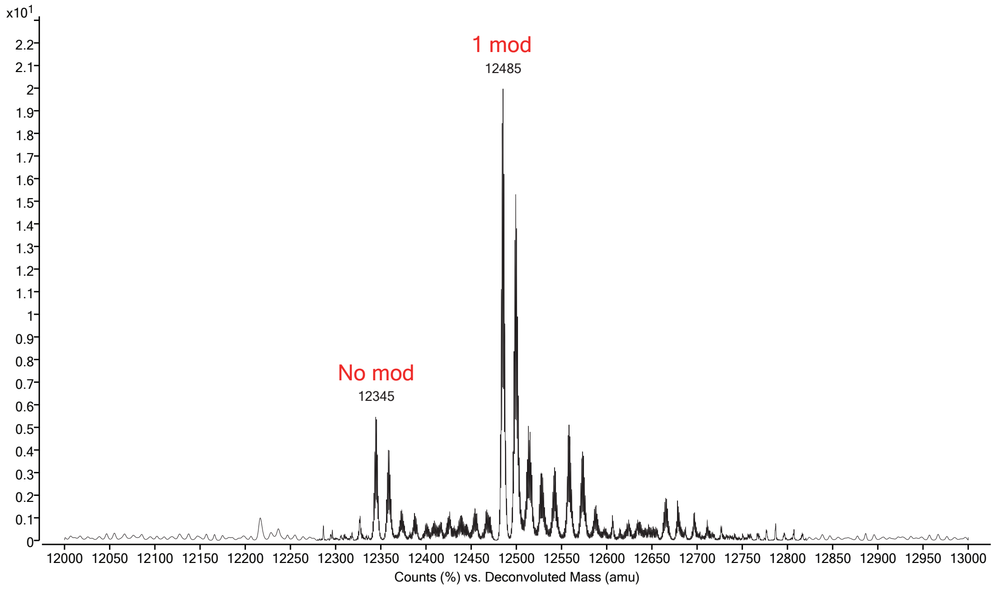

Peptide fragment of homogeneous modified Cytochrome C

The site of modified on Cytochrome C, obtained by treatment with 1.2 equiv. of oxidized furan and 1.2 equiv. of thioglycolic acid in 8 h was determined by Agilent Bioconfirm software after trypsin digestion using the SMART Digest™ Trypsin Kit by Thermo Scientific. The modification site was identified to be K8. The peptide fragment shown in the figures were from AA residues 8-13 with the sequence KIFVQK

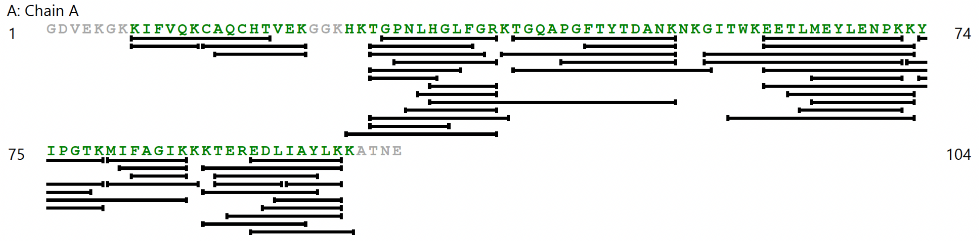

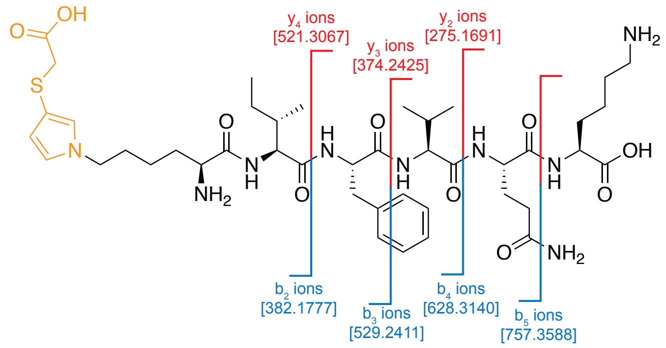

Exact Mass: 901.47

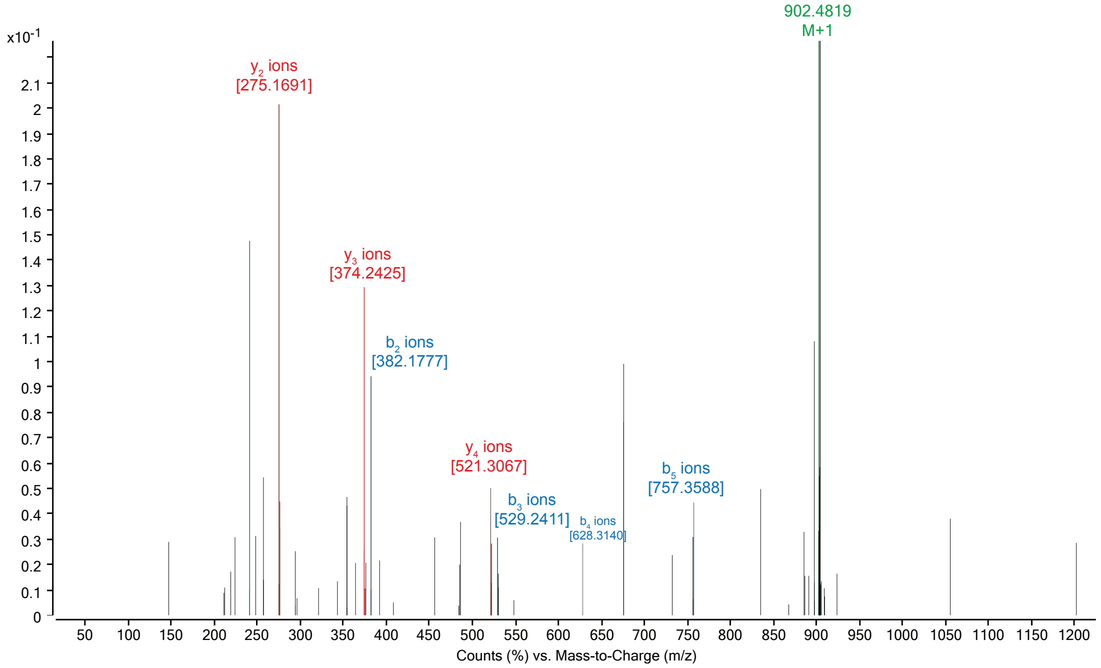

Homogeneous labeling of lysozyme egg white

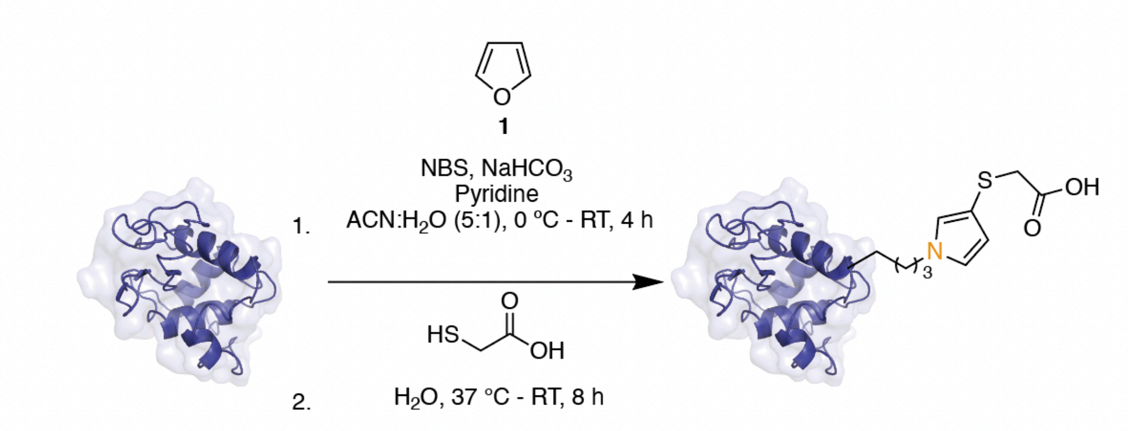

Furan **1** (100 µL, 1.38 mmol) and sodium bicarbonate (115 mg, 1.38 mmol) were added in a solution of 12 mL acetonitrile and water (5:1). The reaction mixture was cooled to 0 °C and left to stir for 15 min. N-Bromosuccinimide (244 mg, 1.38 mmol) was dissolved in a solution of 12 mL acetonitrile and water (5:1) and added to the reaction mixture dropwise. Afterwards, the reaction mixture was left to stir for 10 min, and pyridine (222 µL, 2.76 mmol) was added to the reaction mixture. The reaction mixture was stirred for 4 h and used without further purification. From the pot, 1.2 equiv. (2.9µL) of mixture was taken and incubated with thioglycolic acid (1.2 equiv.) at 37 °C for 30 min in 250 µL of water. 2 mg of lysozyme egg white (1 equiv.) was dissolved in 250 µL of water and added to the reaction mixture (protein concentration in reaction mixture: 280 µM). The reaction mixture was left to stir for 8 h at RT. The reaction mixture was purified by molecular weight cut off and characterized by LCMS to analyze the protein modification. Percent conversions were calculated based on the deconvolution spectra.

MS spectrum of homogeneous labeled lysozyme egg white

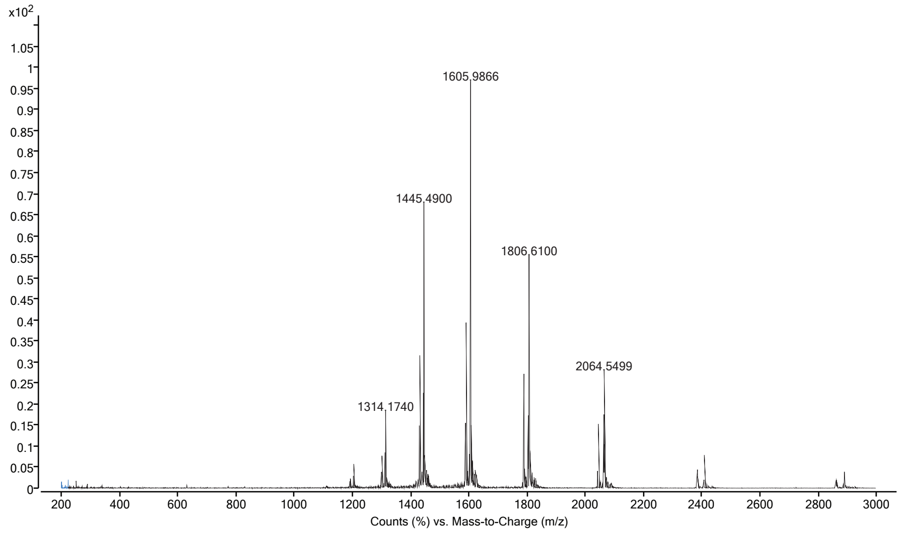

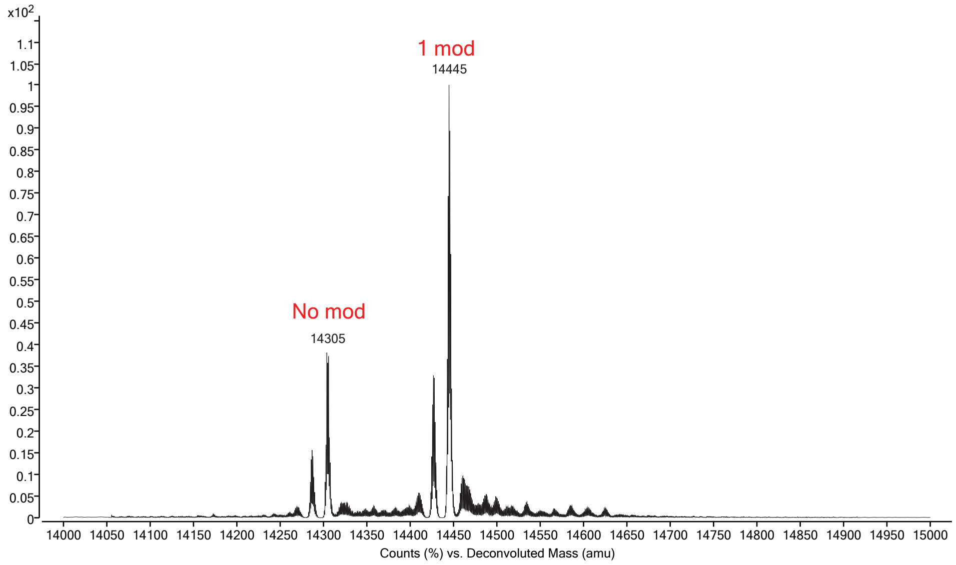

Peptide fragment of homogeneous modified lysozyme egg white

The site of modified on Cytochrome C, obtained by treatment with 1.2 equiv. of oxidized furan and 1.2 equiv. of thioglycolic acid in 8 h was determined by Agilent Bioconfirm software after trypsin digestion using the SMART Digest™ Trypsin Kit by Thermo Scientific. The modification site was identified to be K1 and K97 (3:1). The peptide fragment shown in the figures were from AA residues 1-5 with the sequence KVFGR and AA residues KIVSDGNGMNAW.

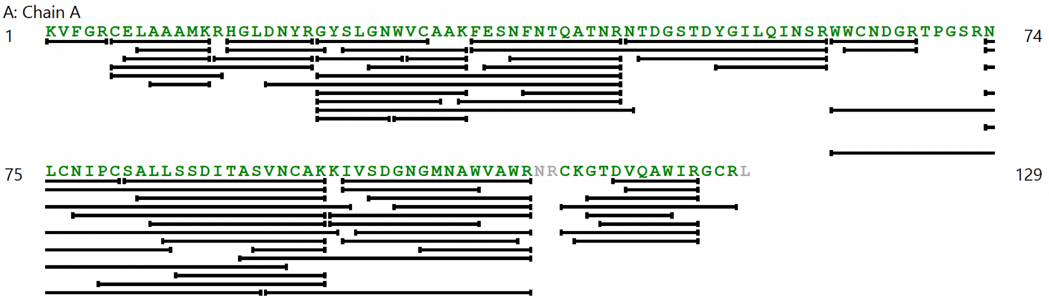

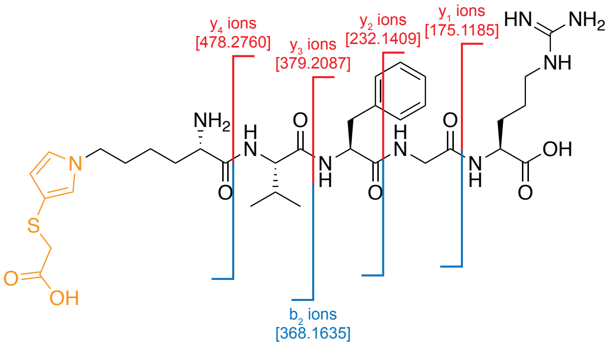

Exact Mass: 745.36

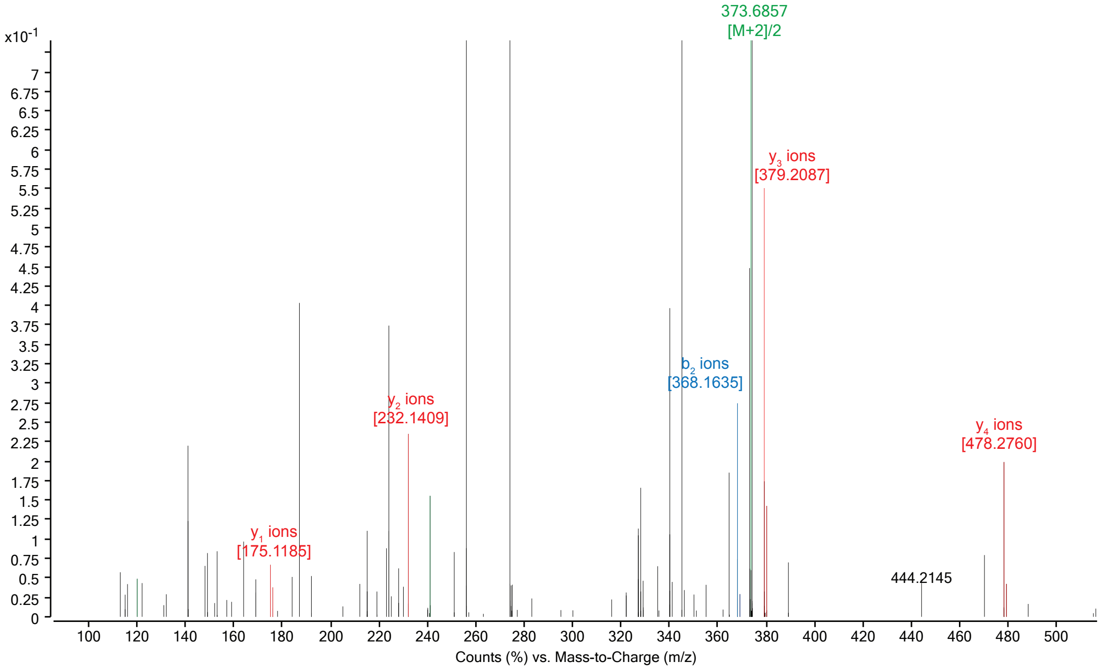

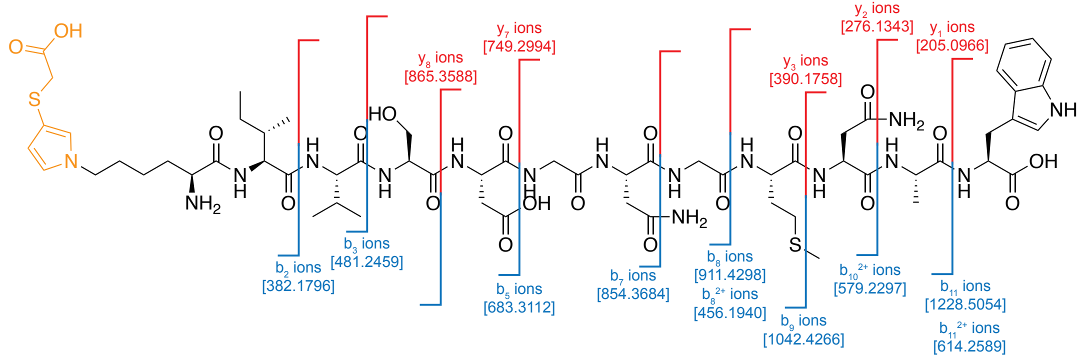

Exact Mass: 1430.60

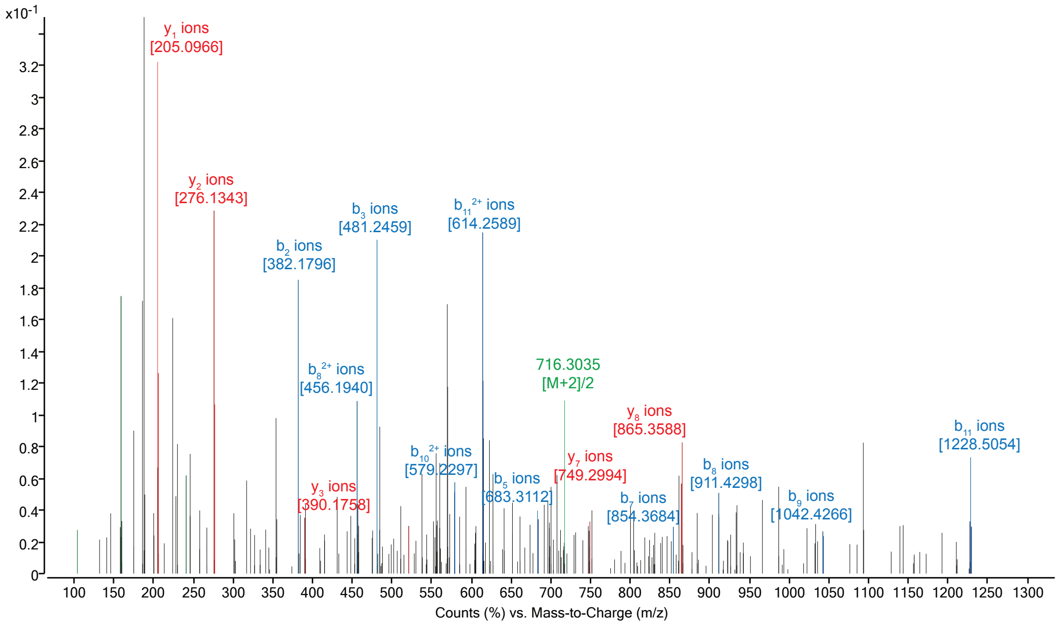

Homogeneous labeling of aprotinin

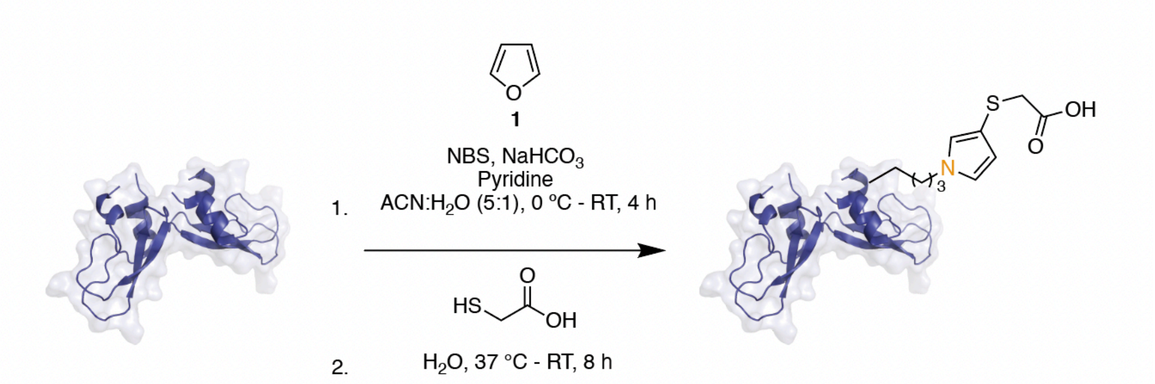

Furan **1** (100 µL, 1.38 mmol) and sodium bicarbonate (115 mg, 1.38 mmol) were added in a solution of 12 mL acetonitrile and water (5:1). The reaction mixture was cooled to 0 °C and left to stir for 15 min. N-Bromosuccinimide (244 mg, 1.38 mmol) was dissolved in a solution of 12 mL acetonitrile and water (5:1) and added to the reaction mixture dropwise. Afterwards, the reaction mixture was left to stir for 10 min, and pyridine (222 µL, 2.76 mmol) was added to the reaction mixture. The reaction mixture was stirred for 4 h and used without further purification. From the pot, 1.2 equiv. (6.4 µL) of mixture was taken and incubated with thioglycolic acid (1.2 equiv.) at 37 °C for 30 min in 250 µL of water. 2 mg of lysozyme egg white (1 equiv.) was dissolved in 250 µL of water and added to the reaction mixture (protein concentration in reaction mixture: 614 µM). The reaction mixture was left to stir for 8 h at RT. The reaction mixture was purified by molecular weight cut off and characterized by LCMS to analyze the protein modification. Percent conversions were calculated based on the deconvolution spectra.

MS spectrum of homogeneous labeled aprotinin

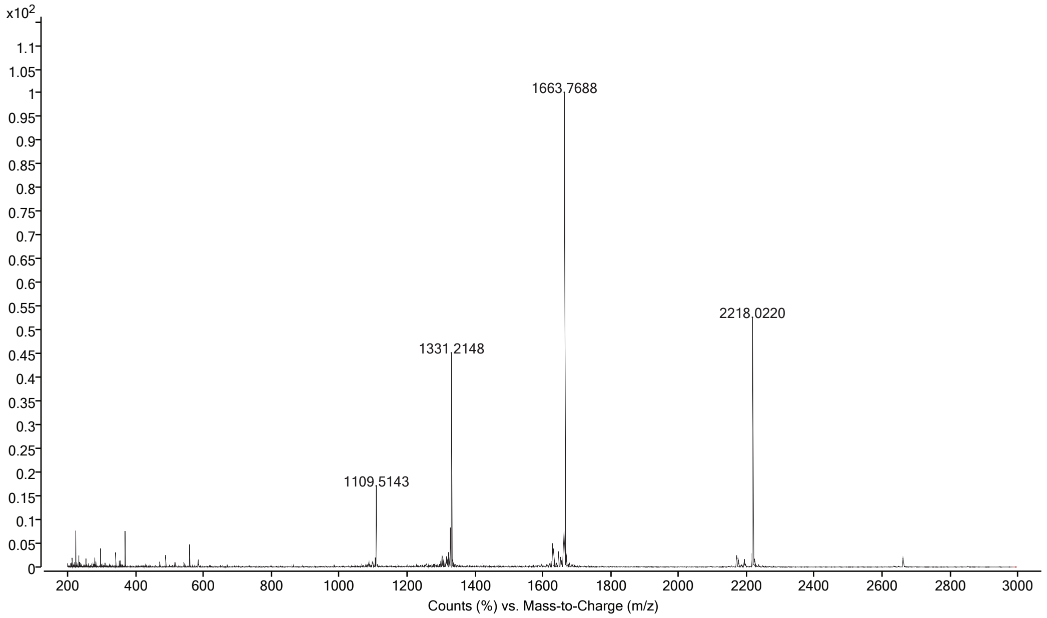

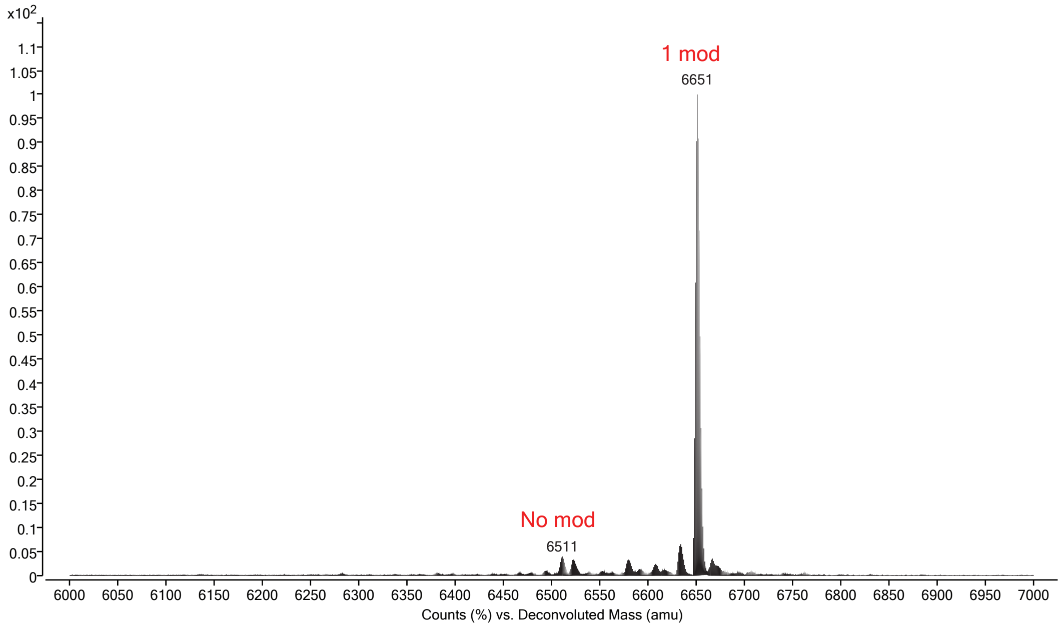

Peptide fragment of homogeneous modified Aprotinin

The site of modified on Aprotinin, obtained by treatment with 1.2 equiv. of oxidized furan and 1.2 equiv. of thioglycolic acid in 8 h was determined by Agilent Bioconfirm software after trypsin digestion using the SMART Digest™ Trypsin Kit by Thermo Scientific. The modification site was identified to be K46. The peptide fragment shown in the figures were from AA residues 46-53 with the sequence KSAEDCMR.

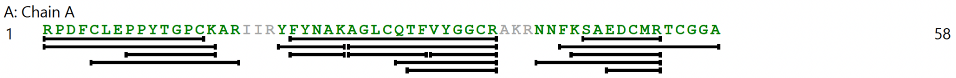

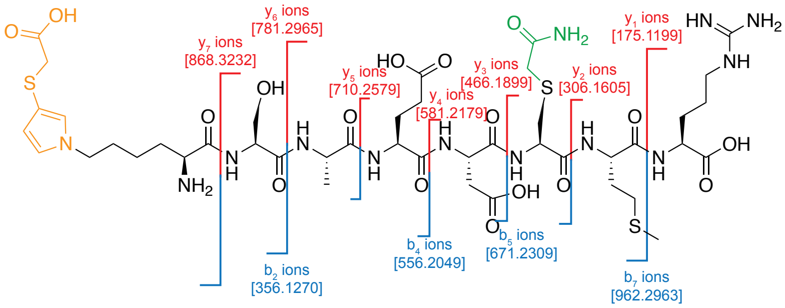

Exact Mass: 1135.41
Alkalyation (iodoacetamide) comes from protein digestion

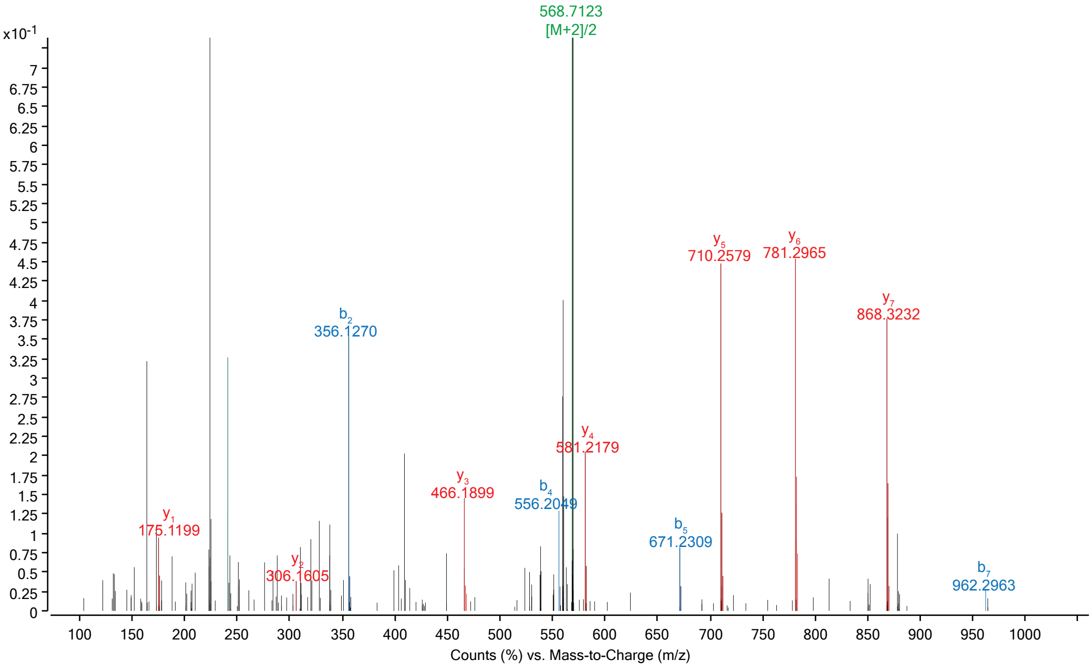

XXII. Supplementary Fig. 17. Dual modification of cysteine and lysine residues on aprotinin

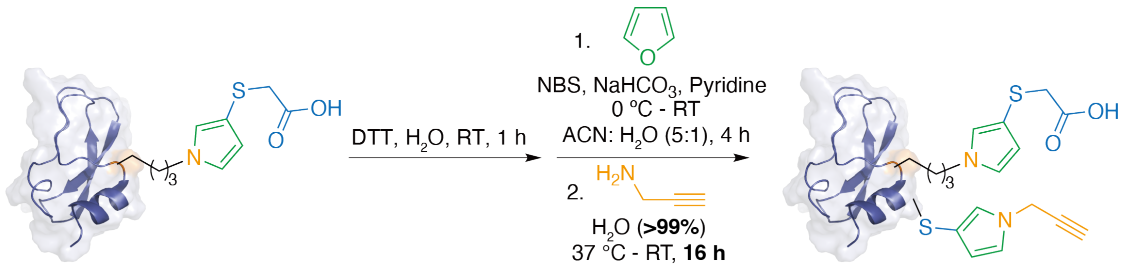

Furan (100 µL, 1.38 mmol) and sodium bicarbonate (115 mg, 1.38 mmol) were added in a solution of 12 mL acetonitrile and water (5:1). The reaction mixture was cooled to 0 °C and left to stir for 15 min. N-Bromosuccinimide (244mg, 1.38 mmol) was dissolved in a solution of 12 mL acetonitrile and water (5:1) and added to the reaction mixture dropwise. Afterwards, the reaction mixture was left to stir for 10 min, and pyridine (222 µL, 2.76 mmol) was added to the reaction mixture. The reaction mixture was stirred for 4 h and used without further purification.

6.5 mM of dithiothreitol in water was prepared and K46 labeled aprotinin were incubated in the DTT solution (1 mL) at room temperature prior to FuTine labeling.

From the furan pot, 5 equiv. of mixture was taken and incubated with the protein samples for 15 minutes. propargylamine (5 equiv.) was added to the reaction mixture and was left to stir for 16 h at RT. The reaction mixture was purified by molecular weight cut off and characterized by MS/MS sequencing using the SMART Digest™ Trypsin Kit by Thermo Scientific. The modification site was identified by Agilent Bioconfirm software to be C30 and C38 (1:1). The peptide fragment shown in the figures were from AA residues 22-39 with the sequence KVFGR and AA residues FYNAKAGLCQTFVYGGCR.

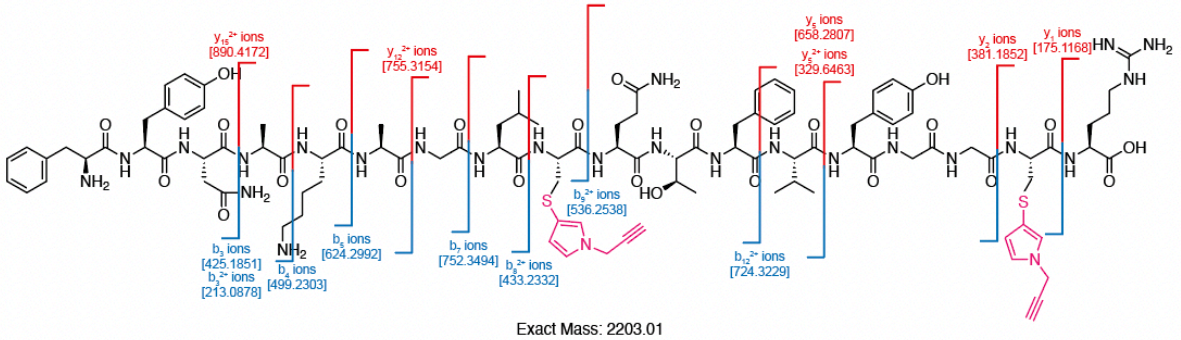

Exact Mass: 2203.01

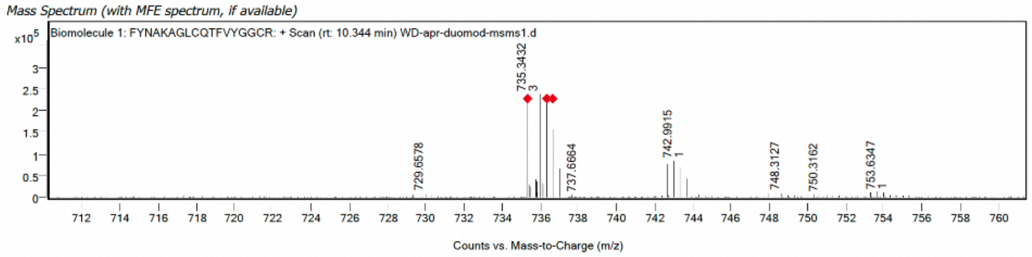

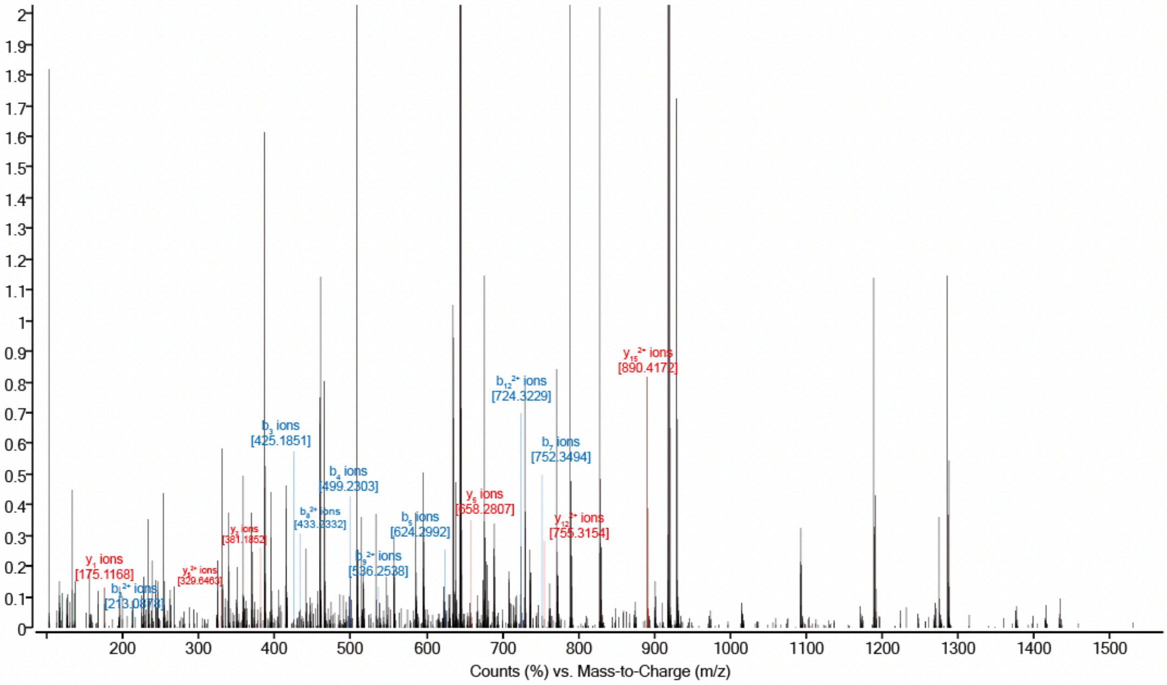

XXIII. Supplementary Fig. 18. Modification of proteins by Furan-Thiol-Amine multicomponent reaction under optimized conditions

Modification of α-Lactalbumin

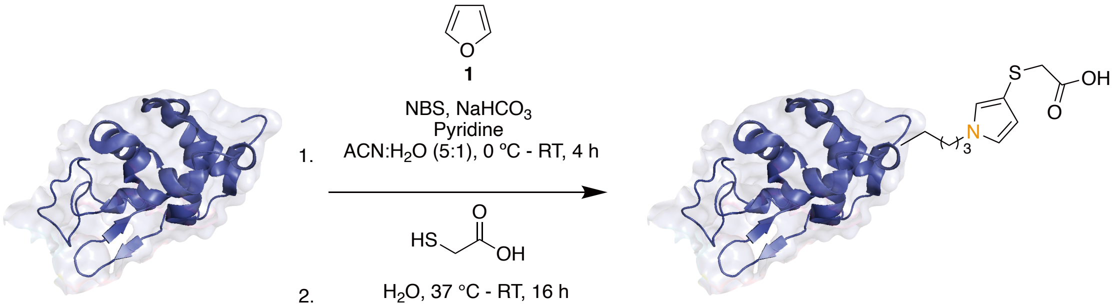

Furan **1** (100 µL, 1.38 mmol) and sodium bicarbonate (115 mg, 1.38 mmol) were added in a solution of 12 mL acetonitrile and water (5:1). The reaction mixture was cooled to 0 °C and left to stir for 15 min. N-Bromosuccinimide (244 mg, 1.38 mmol) was dissolved in a solution of 12 mL acetonitrile and water (5:1) and added to the reaction mixture dropwise. Afterwards, the reaction mixture was left to stir for 10 min, and pyridine (222 µL, 2.76 mmol) was added to the reaction mixture. The reaction mixture was stirred for 4 h and used without further purification. From the furan pot, 5 equiv. (12.3 µL) of mixture was taken and incubated with thioglycolic acid (5 equiv.) at 37 °C for 30 min in 1 mL of water. 2 mg of **α-Lactalbumin** (1 equiv.) was dissolved in 2 mL of water and added to the reaction mixture. The concentration of protein in the reaction mixture was 47 µM. The reaction mixture was left to stir for 16 h at RT. The reaction mixture was purified by molecular weight cut off and analyzed by LCMS to analyze the protein modification. Percent conversions to modified α-Lactalbumin were calculated based on the deconvolution spectrum.

MS spectrum of unmodified α-Lactalbumin

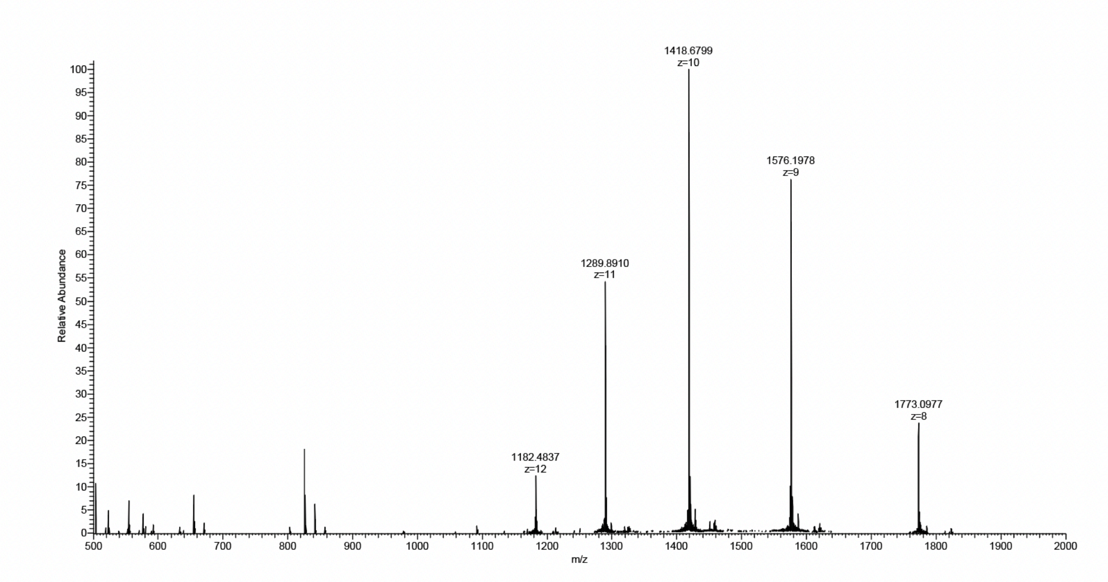

(Deconvoluted spectrum)

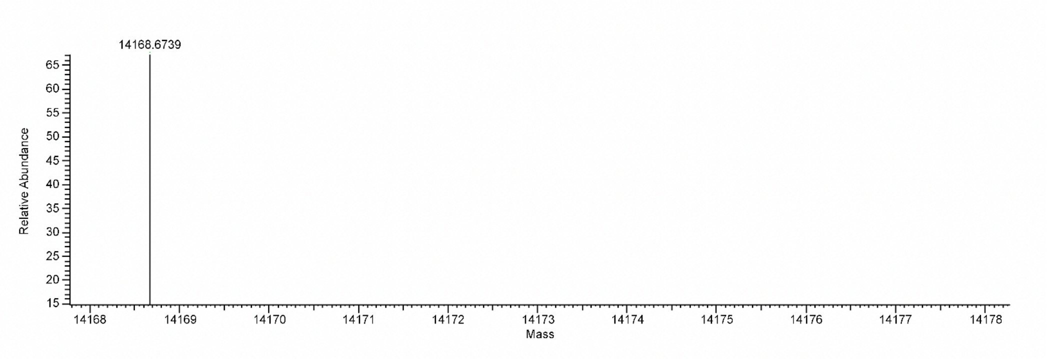

MS spectrum of modified α-Lactalbumin

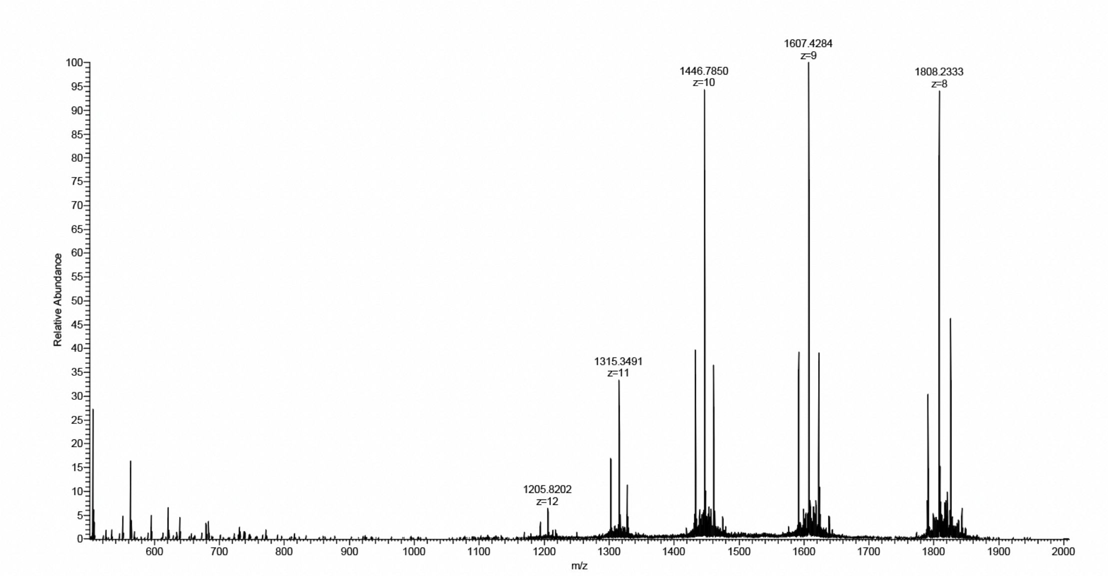

(Deconvoluted spectrum)

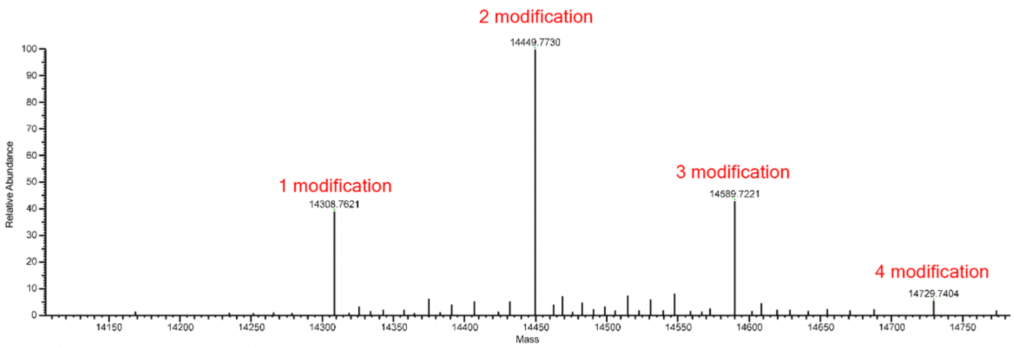

# Modifications	Expected Mass	Observed Mass	% Conversion
1 modification	14308.3339	14308.7621	21
2 modifications	14448.4939	14449.7730	53
3 modifications	14588.6539	14589.7221	23
4 modifications	14728.8139	14729.7404	3

Modification of Carbonic Anhydrase

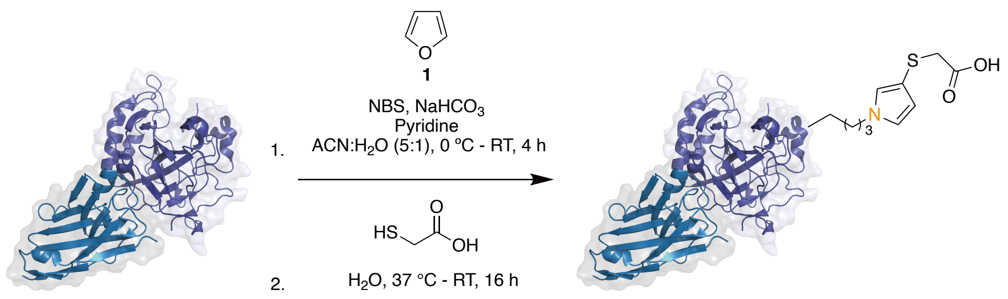

Furan **1** (100 µL, 1.38 mmol) and sodium bicarbonate (115 mg, 1.38 mmol) were added in a solution of 12 mL acetonitrile and water (5:1). The reaction mixture was cooled to 0 °C and left to stir for 15 min. N-Bromosuccinimide (244 mg, 1.38 mmol) was dissolved in a solution of 12 mL acetonitrile and water (5:1) and added to the reaction mixture dropwise. Afterwards, the reaction mixture was left to stir for 10 min, and pyridine (222 µL, 2.76 mmol) was added to the reaction mixture. The reaction mixture was stirred for 4 h and used without further purification. From the furan pot, 5 equiv. (6.0 µL) of mixture was taken and incubated with thioglycolic acid (5 equiv.) at 37 °C for 30 min in 1 mL of water. 2 mg of **carbonic anhydrase** (1 equiv.) was dissolved in 2 mL of water and added to the reaction mixture (Concentration of protein in reaction: 23 µM). The reaction mixture was left to stir for 16 h at RT. The reaction mixture was purified by molecular weight cut off and characterized by LCMS to analyze the protein modification. Percent conversions to modified carbonic anhydrase were calculated based on the deconvolution spectrum.

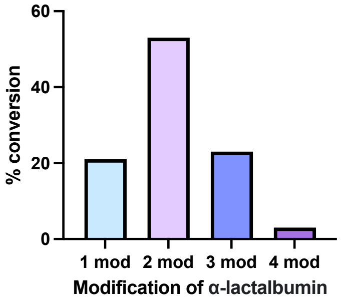

MS spectrum of unmodified carbonic anhydrase

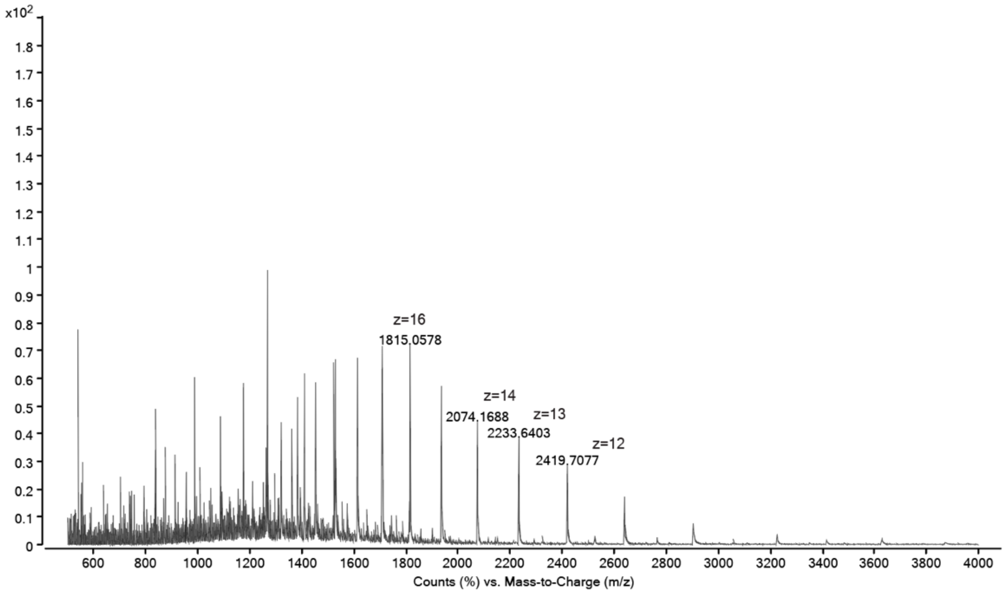

(Deconvoluted spectrum)

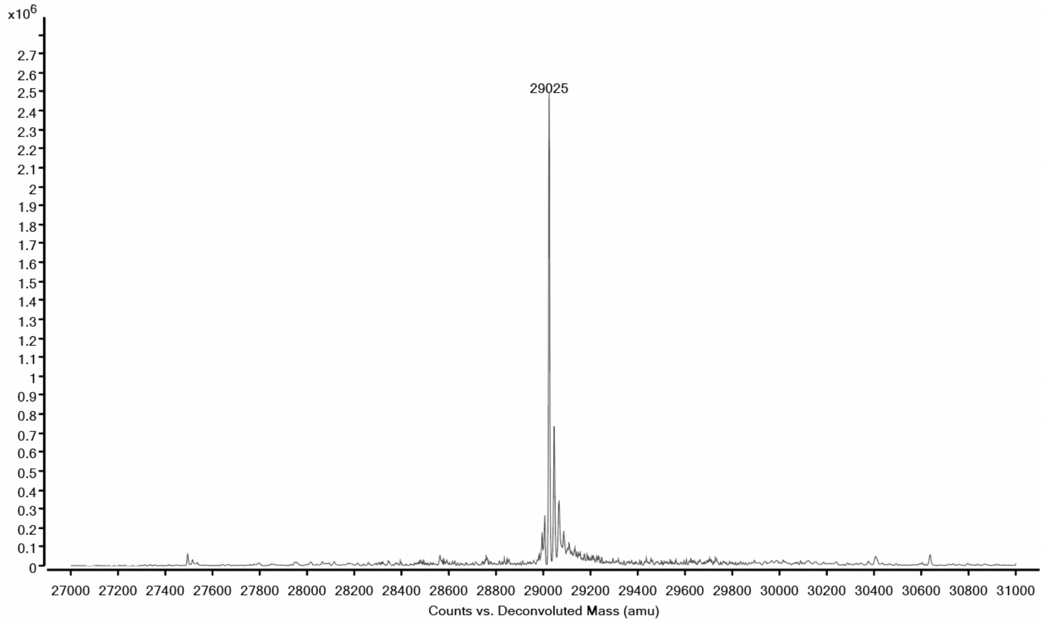

MS spectrum of modified carbonic anhydrase

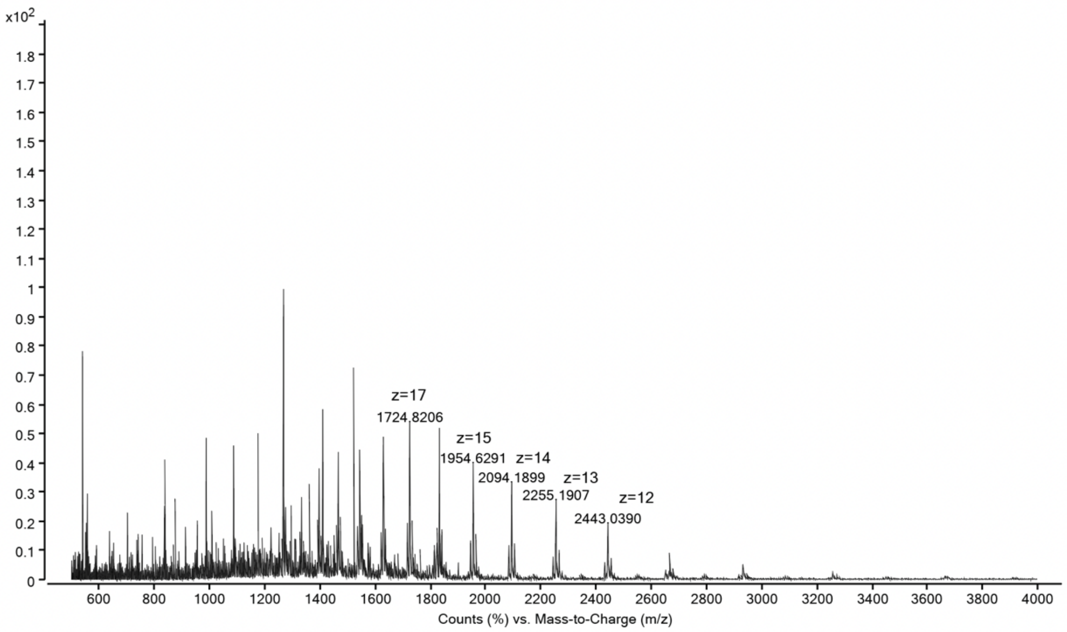

(Deconvoluted spectrum)

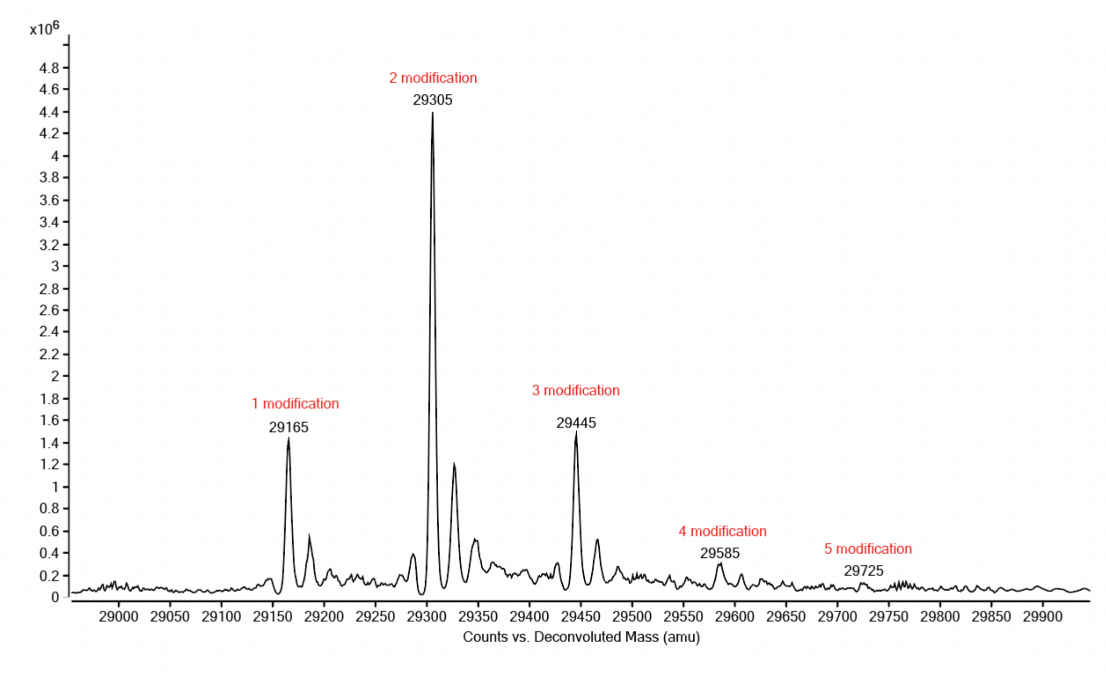

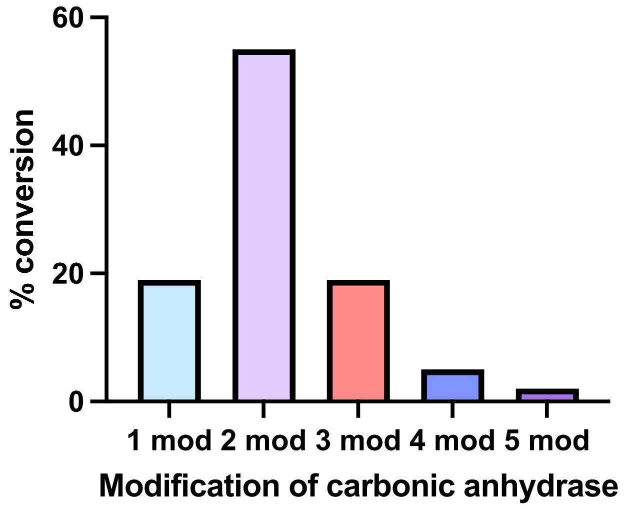

# Modifications	Expected Mass	Observed Mass	% Conversion
1 modification	29165.16	29165	19
2 modifications	29305.32	29305	55
3 modifications	29445.48	29445	19
4 modifications	29585.64	29585	5
5 modifications	29725.80	29725	2

Modification of Cytochrome C

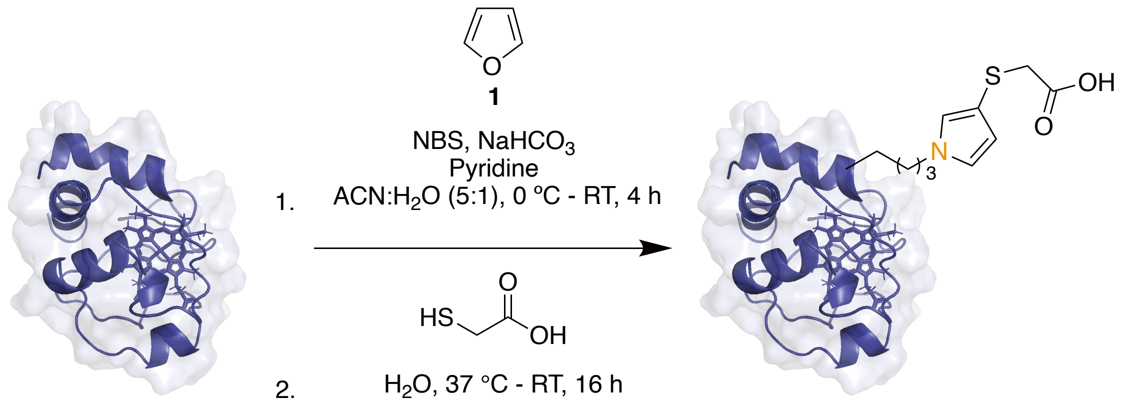

Furan **1** (100 µL, 1.38 mmol) and sodium bicarbonate (115 mg, 1.38 mmol) were added in a solution of 12 mL acetonitrile and water (5:1). The reaction mixture was cooled to 0 °C and left to stir for 15 min. N-Bromosuccinimide (244 mg, 1.38 mmol) was dissolved in a solution of 12 mL acetonitrile and water (5:1) and added to the reaction mixture dropwise. Afterwards, the reaction mixture was left to stir for 10 min, and pyridine (222 µL, 2.76 mmol) was added to the reaction mixture. The reaction mixture was stirred for 4 h and used without further purification. From the furan pot, 5 equiv. (14.1 µL) of mixture was taken and incubated with thioglycolic acid (5 equiv.) at 37 °C for 30 min in 1 mL of water. 2 mg of **cytochrome C** (1 equiv.) was dissolved in 2 mL of water and was added to the reaction mixture (Concentration of protein in reaction: 54 µM). The reaction mixture was left to stir for 16 h at RT. The reaction mixture was purified by molecular weight cut off and analyzed by LCMS to analyze the protein modification. Percent conversions to modified Cytochrome C were calculated based on the deconvolution spectrum.

MS spectrum of modified cytochrome C

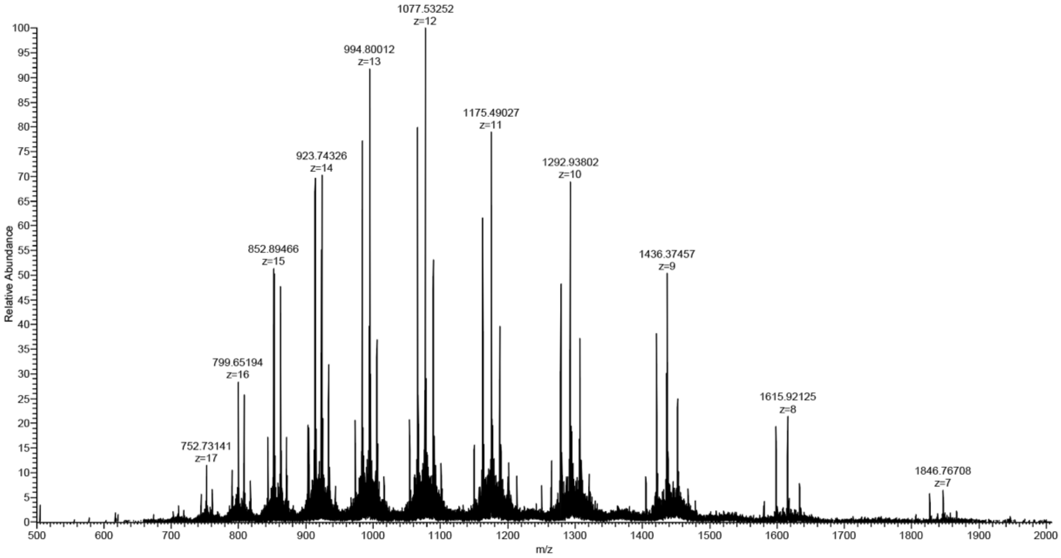

(Deconvoluted spectrum)

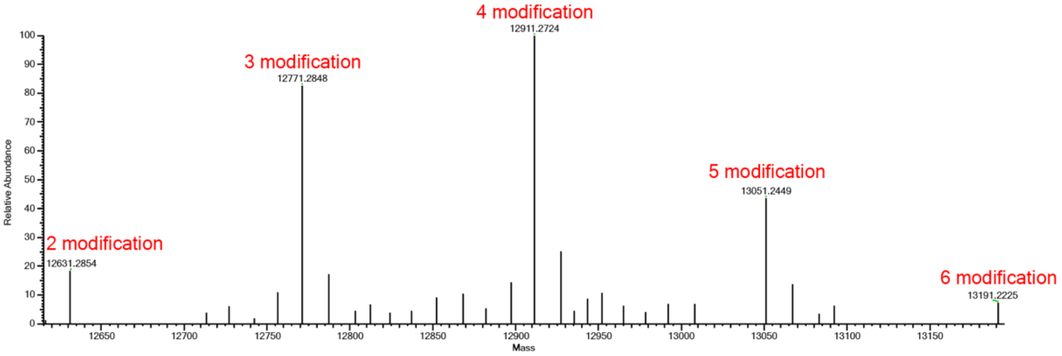

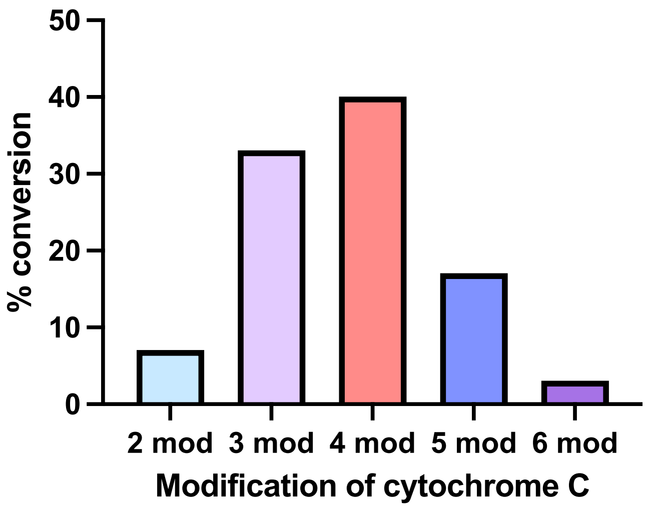

# Modifications	Expected Mass	Observed Mass	% Conversion
2 modifications	12631.32	12631.2854	7
3 modifications	12771.48	12771.2848	33
4 modifications	12911.64	12911.2724	40
5 modifications	13051.80	13051.2449	17
6 modifications	13191.96	13191.2225	3

Modification of Aprotinin

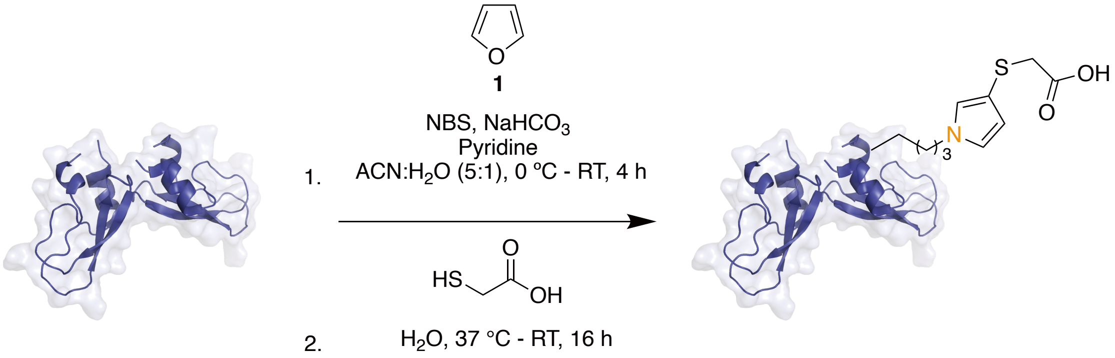

Furan **1** (100 µL, 1.38 mmol) and sodium bicarbonate (115 mg, 1.38 mmol) were added in a solution of 12 mL acetonitrile and water (5:1). The reaction mixture was cooled to 0 °C and left to stir for 15 min. N-Bromosuccinimide (244 mg, 1.38 mmol) was dissolved in a solution of 12 mL acetonitrile and water (5:1) and added to the reaction mixture dropwise. Afterwards, the reaction mixture was left to stir for 10 min, and pyridine (222 µL, 2.76 mmol) was added to the reaction mixture. The reaction mixture was stirred for 4 h and used without further purification. From the furan pot, 5 equiv. (26.7 µL) of mixture was taken and incubated with thioglycolic acid (5 equiv.) at 37 °C for 30 min in 1 mL of water. 2 mg of **aprotinin** (1 equiv.) was dissolved in 2 mL of water and added to the reaction mixture. The concentration of protein in the reaction was 102 µM. The reaction mixture was left to stir for 16 h at RT. The reaction mixture was purified by molecular weight cut off and characterized by LCMS to analyze the protein modification. Percent conversions to modified Aprotinin were calculated based on the deconvolution spectrum.

MS spectrum of unmodified Aprotinin

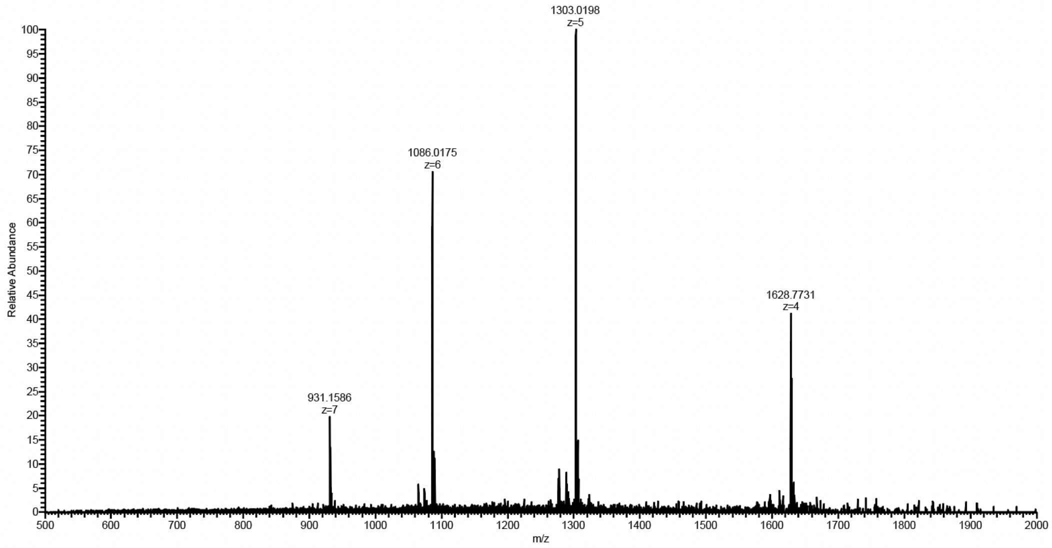

(Deconvoluted spectrum)

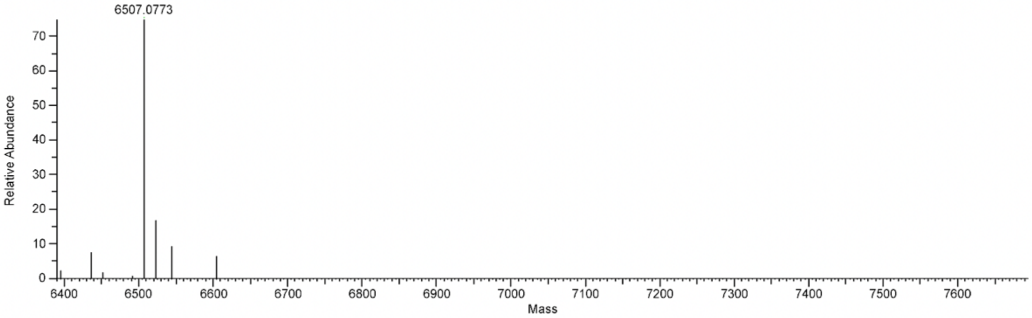

MS spectrum of modified Aprotinin

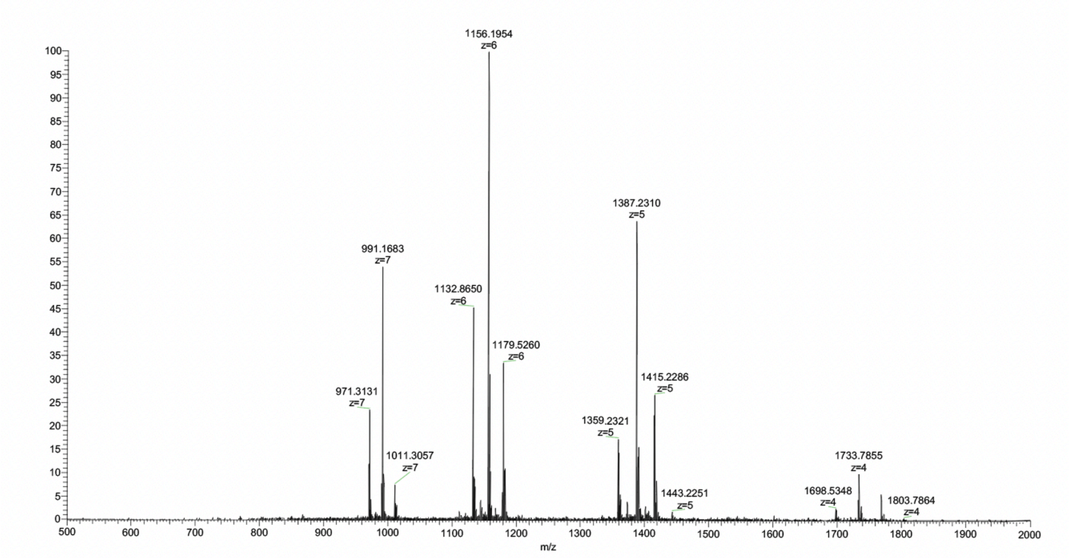

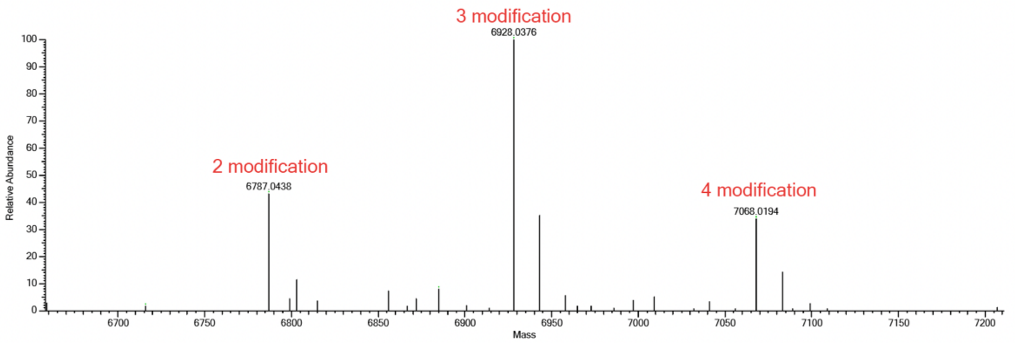

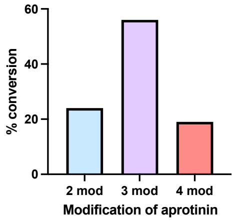

# Modifications	Expected Mass	Observed Mass	% Conversion
2 modifications	6787.3973	6787.0438	24
3 modifications	6927.5573	6928.0376	56
4 modifications	7067.7173	7068.0194	19

Modification of Insulin

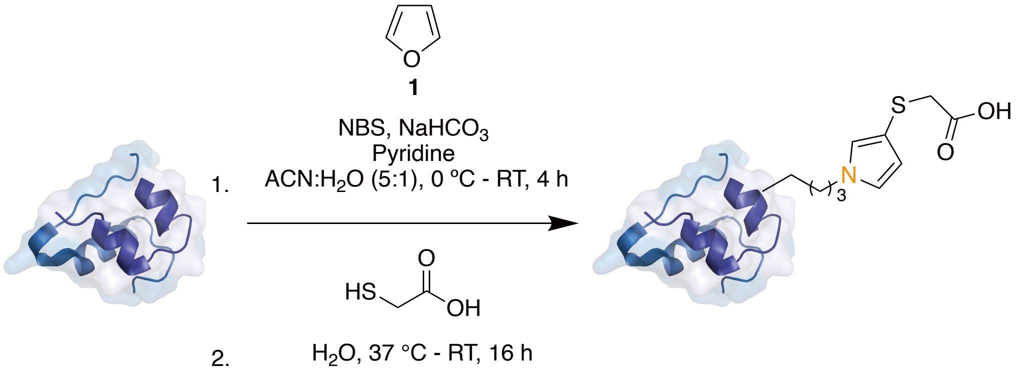

Furan **1** (100 µL, 1.38 mmol) and sodium bicarbonate (115 mg, 1.38 mmol) were added in a solution of 12 mL acetonitrile and water (5:1). The reaction mixture was cooled to 0 °C and left to stir for 15 min. N-Bromosuccinimide (244 mg, 1.38 mmol) was dissolved in a solution of 12 mL acetonitrile and water (5:1) and added to the reaction mixture dropwise. Afterwards, the reaction mixture was left to stir for 10 min, and pyridine (222 µL, 2.76 mmol) was added to the reaction mixture. The reaction mixture was stirred for 4 h and used without further purification. From the furan pot, 5 equiv. (30 µL) of mixture was taken and incubated with thioglycolic acid (5 equiv.) at 37°C for 30 min in 1 mL of water. 2 mg of **insulin** (1 equiv.) was dissolved in 2 mL of water and added to the reaction mixture (Concentration of protein in reaction: 114 µM). The reaction mixture was left to stir for 16 h at RT. The reaction mixture was purified by molecular weight cut off and analyzed by LCMS to analyze the protein modification. Percent conversions to modified Insulin were calculated based on the deconvolution spectrum.

MS spectrum of unmodified Insulin

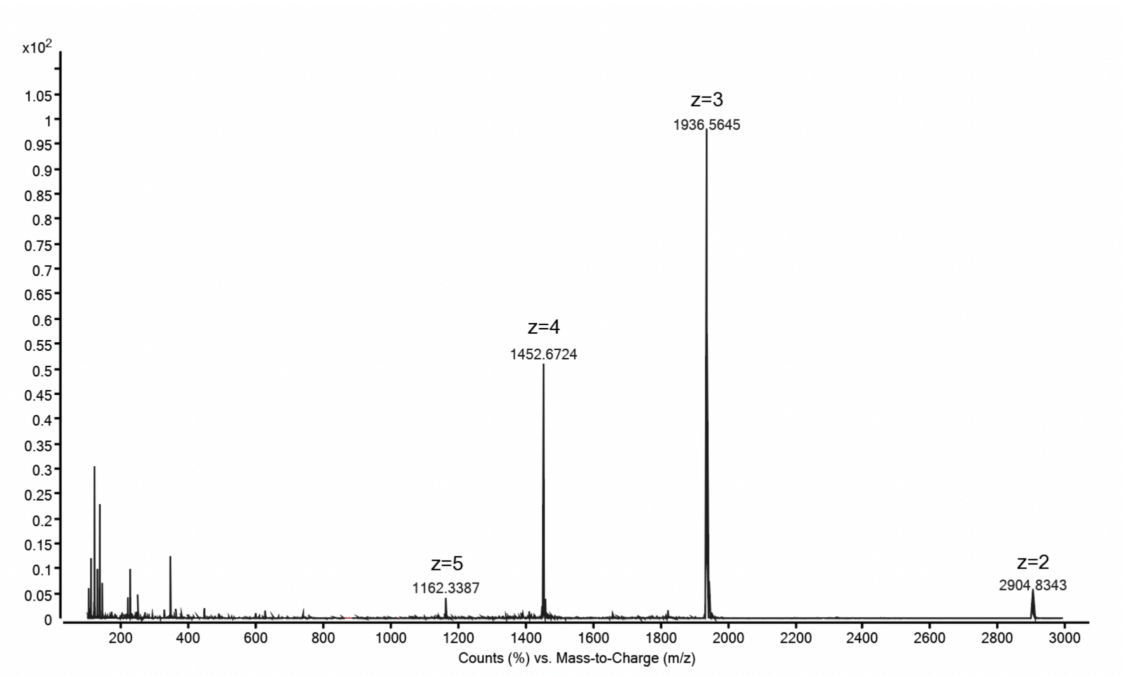

(Deconvoluted spectrum)

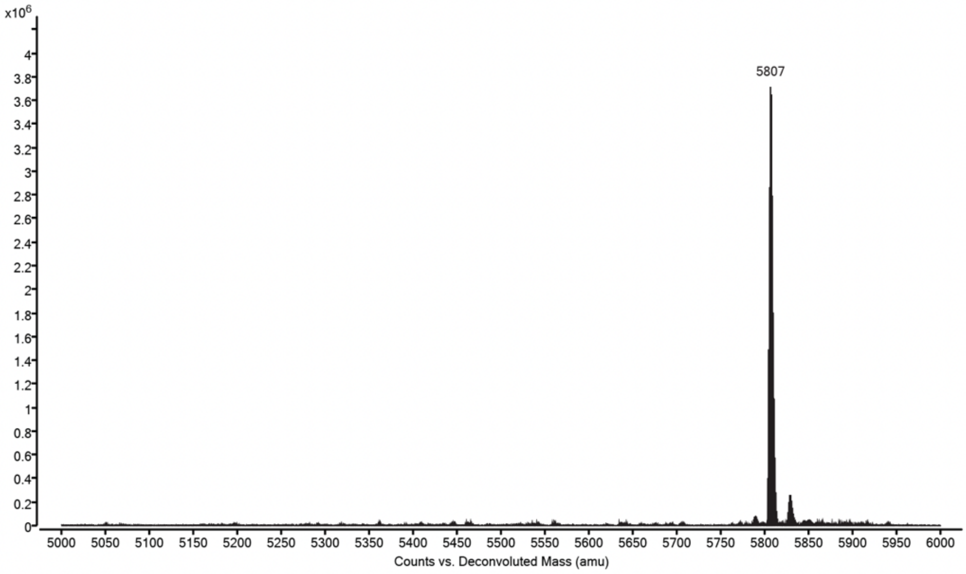

MS spectrum of modified Insulin

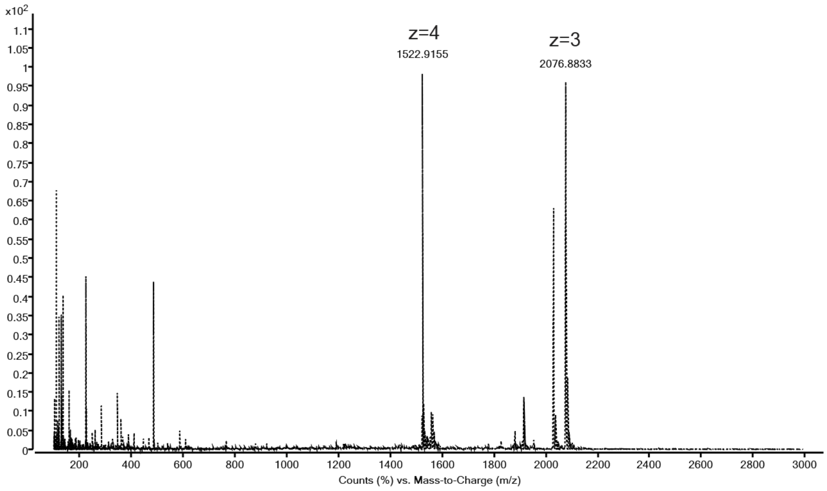

(Deconvoluted spectrum)

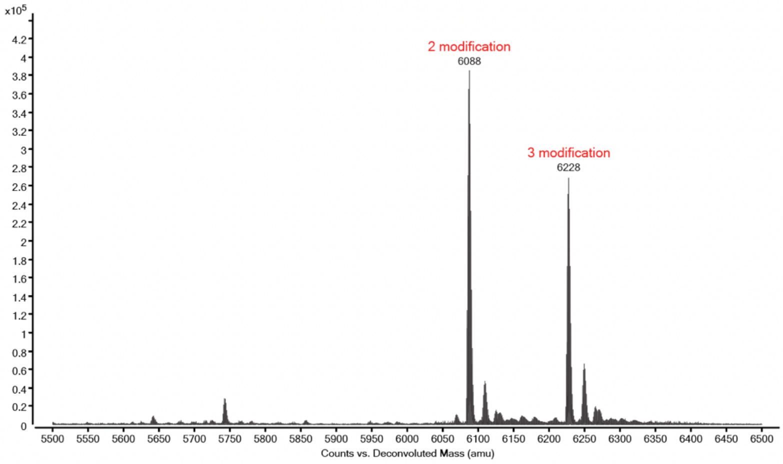

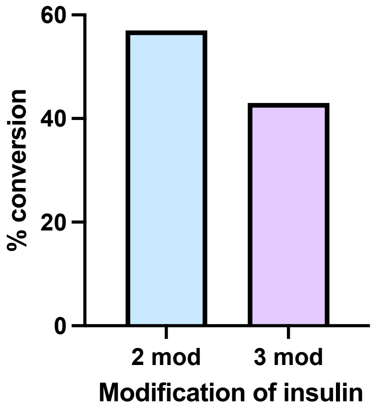

# Modifications	Expected Mass	Observed Mass	% Conversion
2 modifications	6087.32	6088	53 %
3 modifications	6227.48	6228	47 %

Modification of Lysozyme from Chicken Egg White

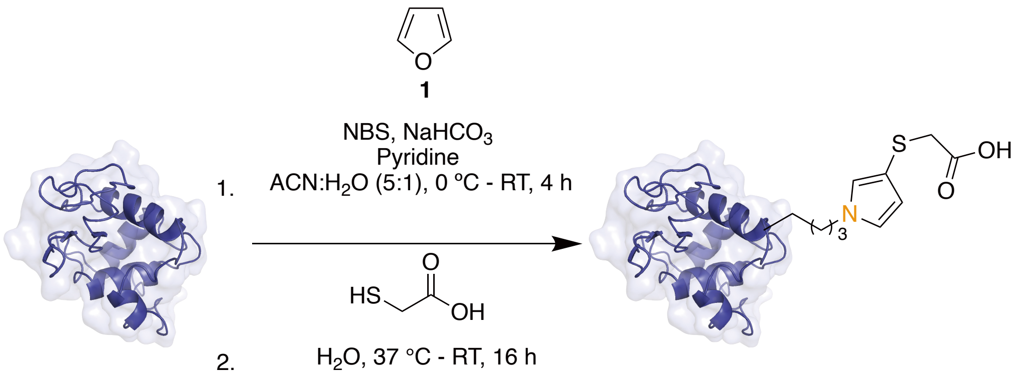

Furan **1** (100 µL, 1.38 mmol) and sodium bicarbonate (115 mg, 1.38 mmol) were added in a solution of 12 mL acetonitrile and water (5:1). The reaction mixture was cooled to 0 °C and left to stir for 15

min. N-Bromosuccinimide (244 mg, 1.38 mmol) was dissolved in a solution of 12 mL acetonitrile and water (5:1) and added to the reaction mixture dropwise. Afterwards, the reaction mixture was left to stir for 10 min, and pyridine (222 µL, 2.76 mmol) was added to the reaction mixture. The reaction mixture was stirred for 4 h and used without further purification. From the furan pot, 5 equiv. (12.2 µL) of mixture was taken and incubated with thioglycolic acid (5 equiv.) at 37°C for 30 min in 1 mL of water. 2 mg of lysozyme from chicken egg white (1 equiv.) was dissolved in 2 mL of water and was added to the reaction mixture. The concentration of protein in the reaction was 47 µM. The reaction mixture was left to stir for 16 h at RT. The reaction mixture was purified by molecular weight cut off and characterized by LCMS to analyze the protein modification. Percent conversions to modified lysozyme from chicken egg white were calculated based on the deconvolution spectrum.

MS spectrum of unmodified lysozyme from chicken egg white

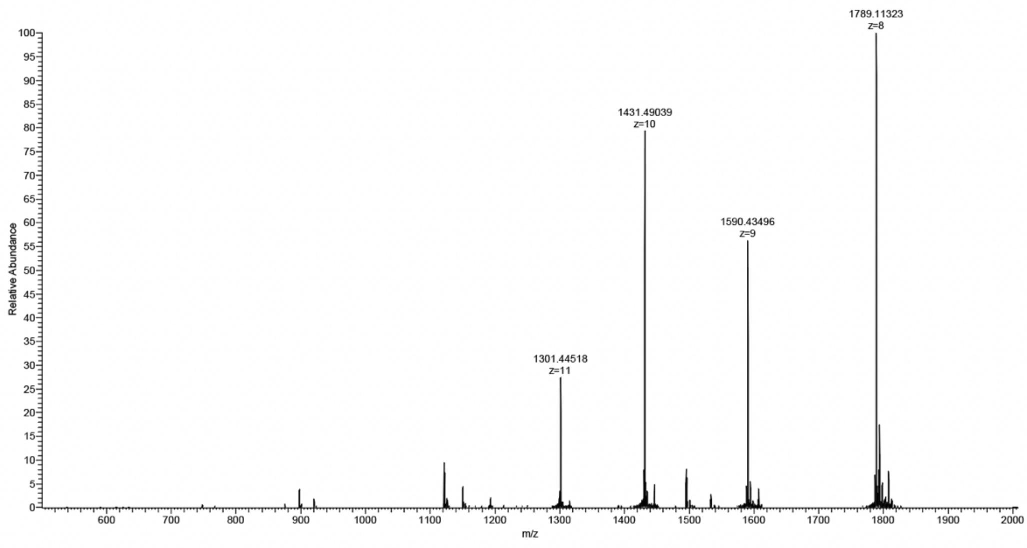

(Deconvoluted spectrum)

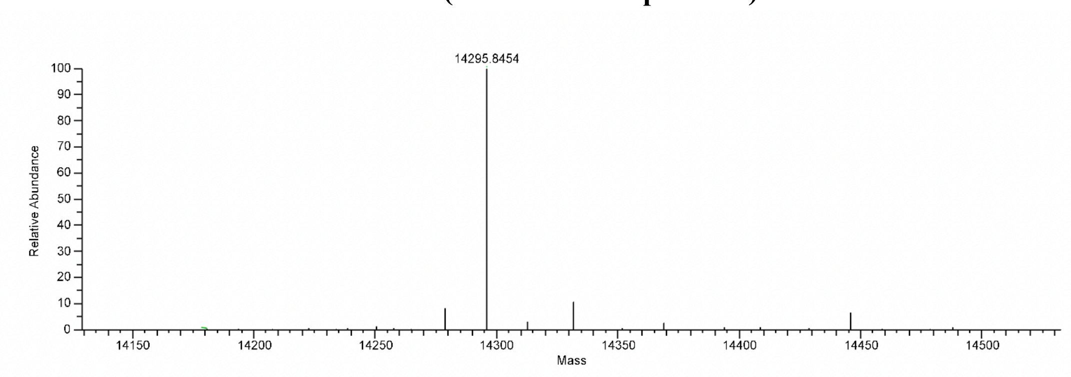

MS spectrum of modified lysozyme from chicken egg white

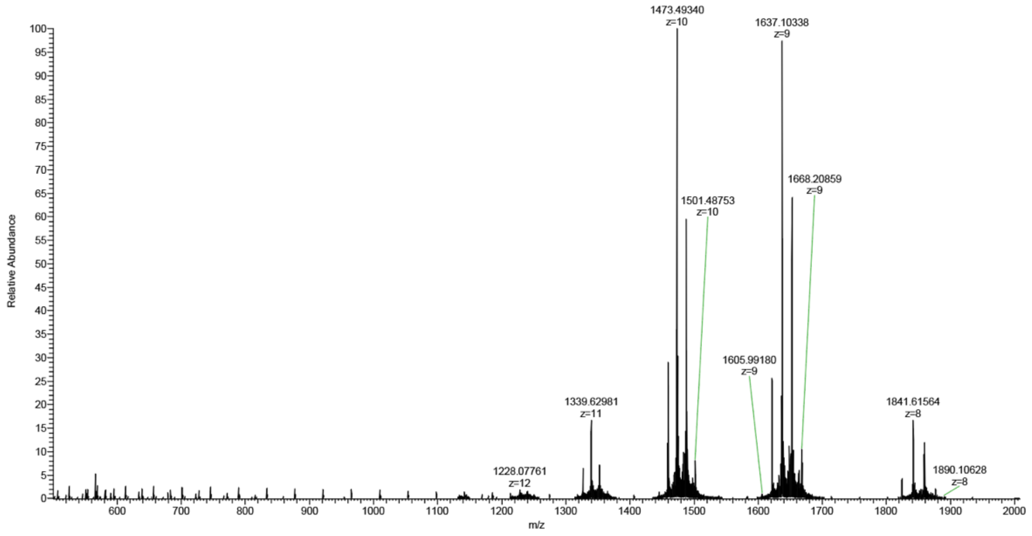

(Deconvoluted spectrum)

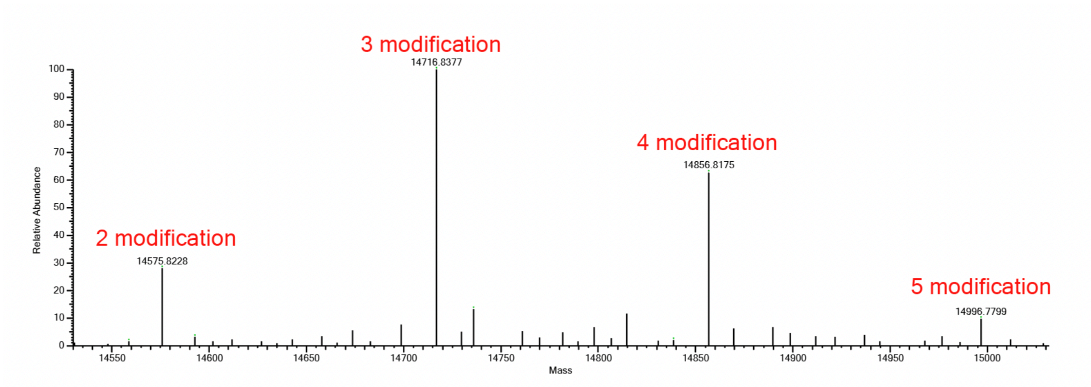

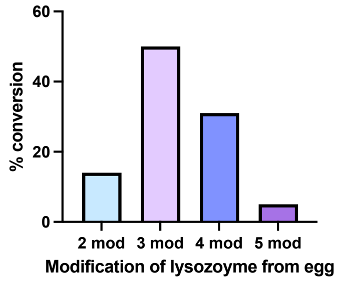

# Modifications	Expected Mass	Observed Mass	% Conversion
2 modifications	14576.1654	14575.8228	14
3 modifications	14716.3254	14716.8377	50
4 modifications	14856.4854	14856.8175	31
5 modifications	14996.6454	14996.7799	5

Modification of Lysozyme human

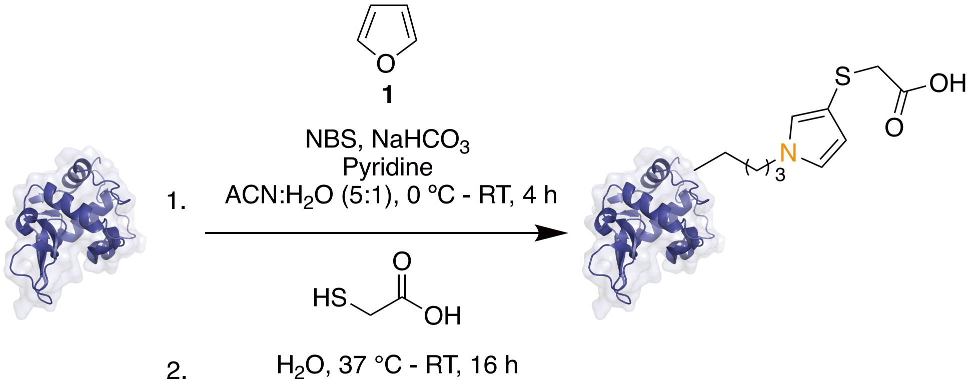

Furan **1** (100 µL, 1.38 mmol) and sodium bicarbonate (115 mg, 1.38 mmol) were added in a solution of 12 mL acetonitrile and water (5:1). The reaction mixture was cooled to 0 °C and left to stir for 15 min. N-Bromosuccinimide (244 mg, 1.38 mmol) was dissolved in a solution of 12 mL acetonitrile and water (5:1) and added to the reaction mixture dropwise. Afterwards, the reaction mixture was left to stir for 10 min, and pyridine (222 µL, 2.76 mmol) was added to the reaction mixture. The reaction mixture was stirred for 4 h and used without further purification. From the furan pot, 5 equiv. (11.8 µL) of mixture was taken and incubated with thioglycolic acid (5 equiv.) at 37 °C for 30 min in 1 mL of water. 2 mg of **lysozyme human** (1 equiv.) was dissolved in 2 mL of water and was added to the reaction mixture. The concentration of protein in the reaction was 45 µM. The reaction mixture was left to stir for 16 h at RT. The reaction mixture was purified by molecular weight cut off and characterized by LCMS to analyze the protein modification. Percent conversions to modified human lysozyme were calculated based on the deconvolution spectrum.

MS spectrum of unmodified lysozyme human

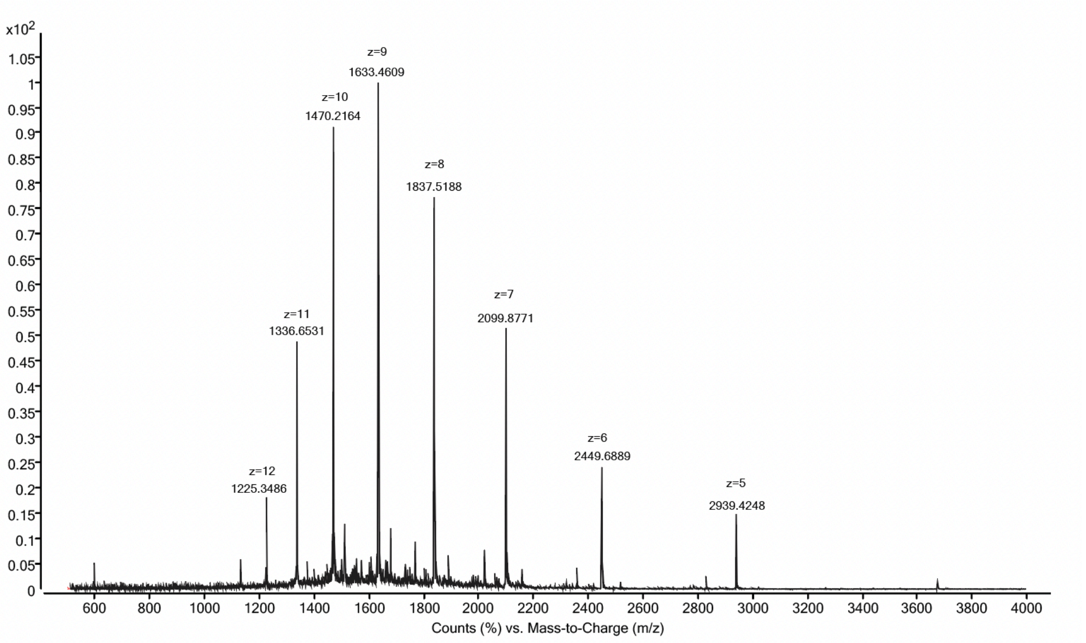

(Deconvoluted spectrum)

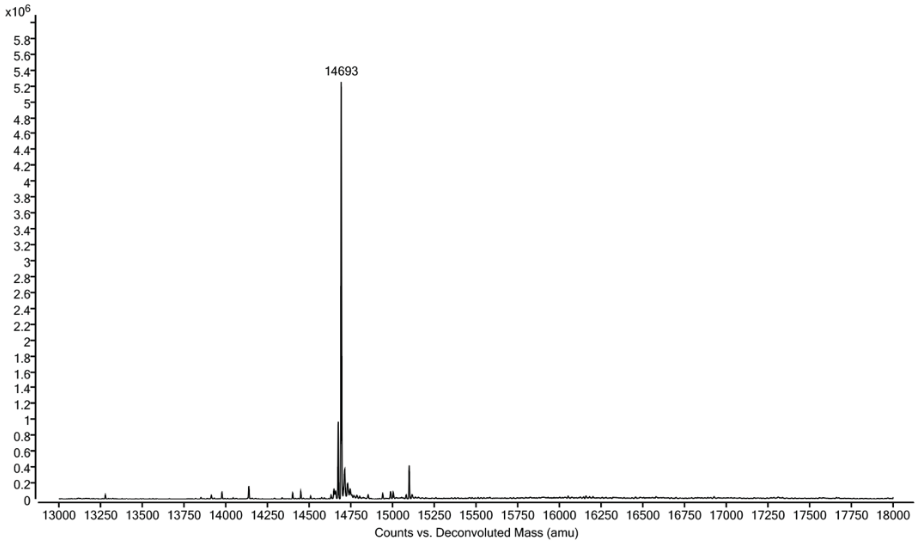

MS spectrum of modified lysozyme human

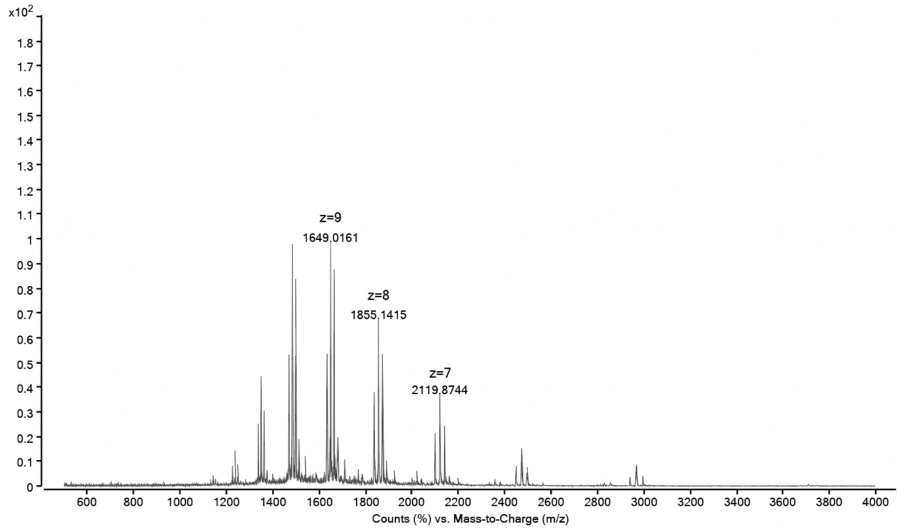

(Deconvoluted spectrum)

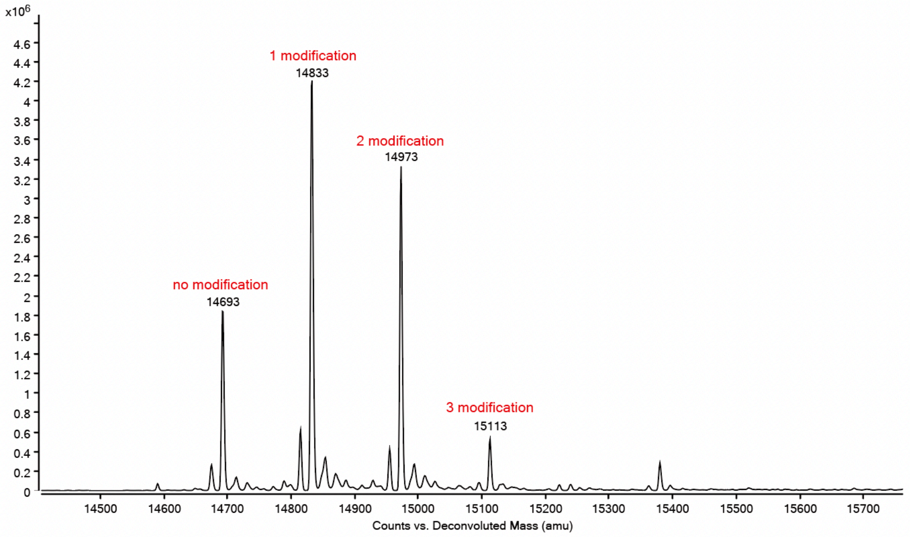

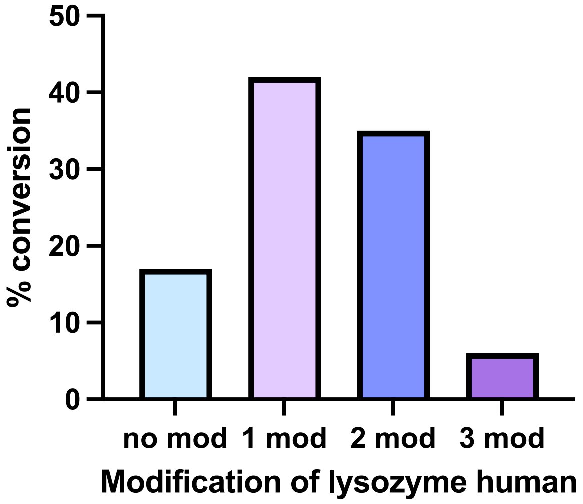

# Modifications	Expected Mass	Observed Mass	% Conversion
0 modifications	14693	14693	17
1 modification	14833.16	14833	42
2 modifications	14973.32	14973	35
3 modifications	15113.48	15113	6

Modification of Ubiquitin

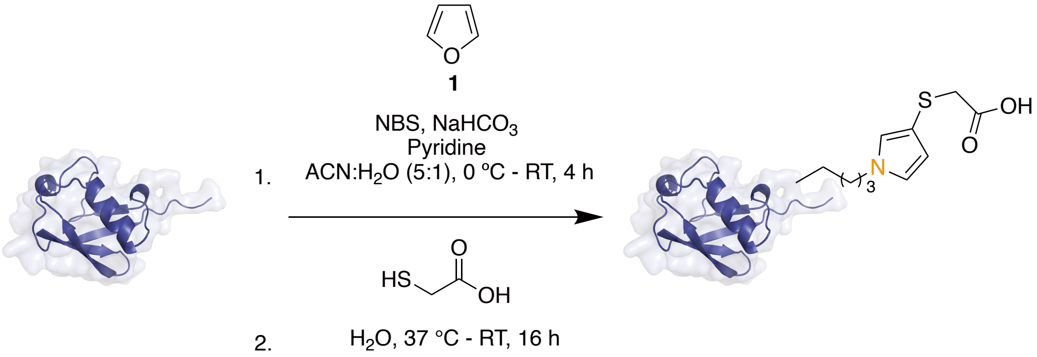

Furan **1** (100 µL, 1.38 mmol) and sodium bicarbonate (115 mg, 1.38 mmol) were added in a solution of 12 mL acetonitrile and water (5:1). The reaction mixture was cooled to 0 °C and left to stir for 15 min. N-Bromosuccinimide (244 mg, 1.38 mmol) was dissolved in a solution of 12 mL acetonitrile and water (5:1) and added to the reaction mixture dropwise. Afterwards, the reaction mixture was left to stir for 10 min, and pyridine (222 µL, 2.76 mmol) was added to the reaction mixture. The reaction mixture was stirred for 4 h and used without further purification. From the furan pot, 5 equiv. (20.3 µL) of mixture was taken and incubated with thioglycolic acid (5 equiv.) at 37 °C for 30 min in 1 mL of water. 2 mg of **Ubiquitin** (1 equiv.) was dissolved in 2 mL of water and added to the reaction mixture (Concentration of protein in reaction: 78 µM). The reaction mixture was left to stir for 16 h at RT. The reaction mixture was purified by molecular weight cut off and characterized by LCMS to analyze the protein modification. Percent conversions to modified ubiquitin were calculated based on the deconvolution spectrum.

MS spectrum of unmodified ubiquitin

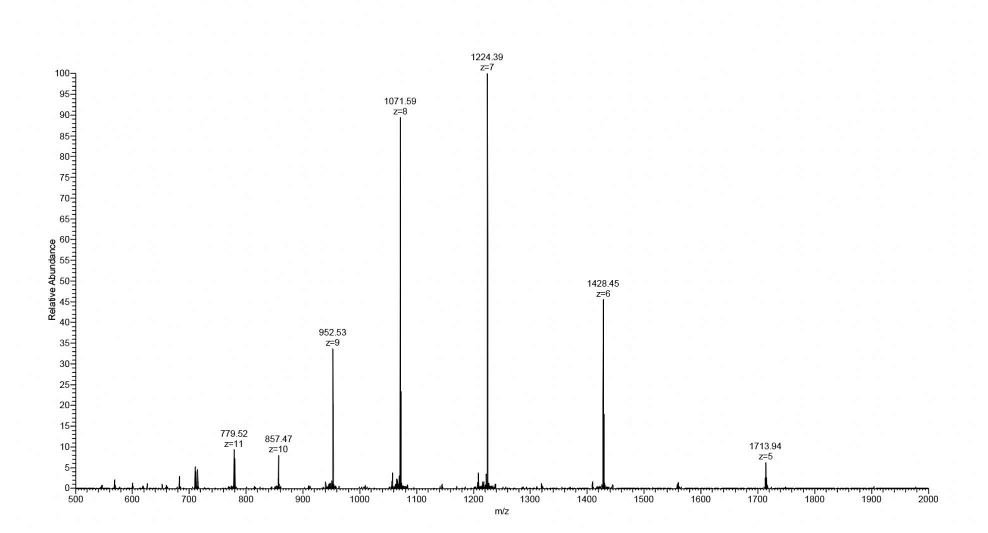

(Deconvoluted spectrum)

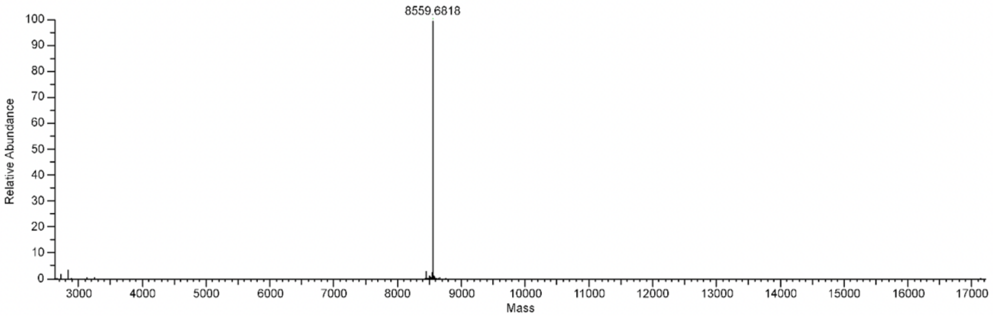

MS spectrum of modified ubiquitin

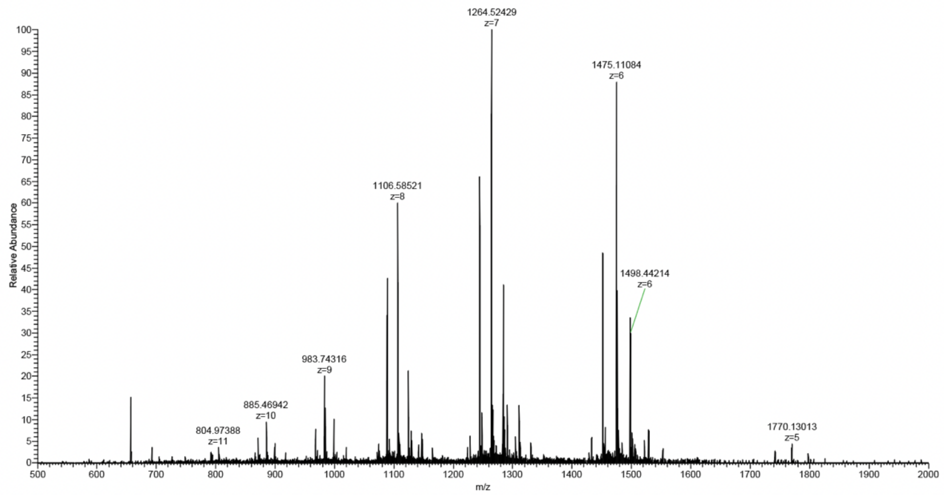

(Deconvoluted spectrum)

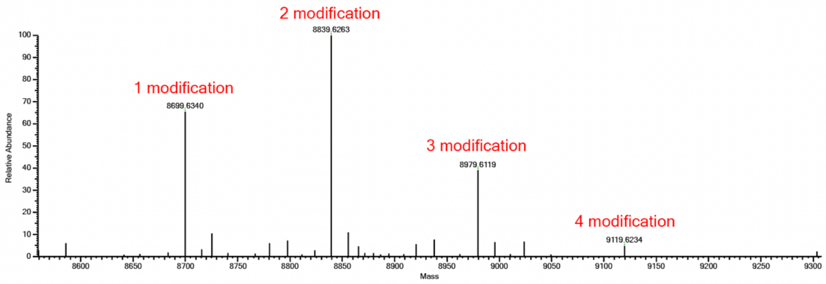

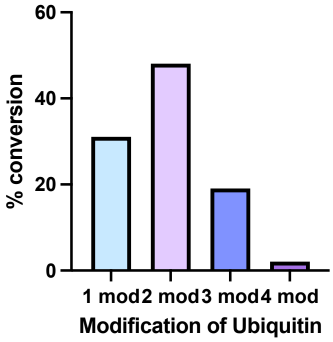

# Modifications	Expected Mass	Observed Mass	% Conversion
1 modification	8699.8418	8699.6340	31
2 modifications	8840.0018	8839.6263	48
3 modifications	8980.1618	8979.6119	19
4 modifications	9120.3218	9119.6234	2

Modification of Transferrin human

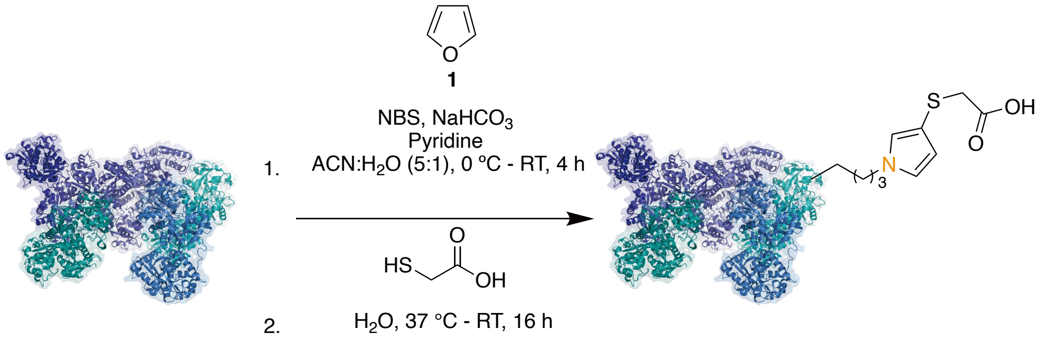

Furan (100 µL, 1.38 mmol) and sodium bicarbonate (115 mg, 1.38 mmol) were added in a solution of 12 mL acetonitrile and water (5:1). The reaction mixture was cooled to 0 °C and left to stir for 15 min. N-Bromosuccinimide (244 mg, 1.38 mmol) was dissolved added in a solution of 12 mL acetonitrile and water (5:1) and added to the reaction mixture dropwise. Afterwards, the reaction mixture was left to stir for 10 min, and pyridine (222 µL, 2.76 mmol) was added to the reaction mixture. The reaction mixture was stirred for 4 h and used without further purification. From the furan pot, 5 equiv. (2.2 µL) of mixture was taken and incubated with thioglycolic acid (5 equiv.) at 37 °C for 30 min in 1 mL of water. 2 mg of Transferrin human (1 equiv.) was dissolved in 2 mL of water and added to the reaction mixture. The concentration of protein in the reaction was 8 µM. The reaction mixture was left to stir for 16 h at RT. The reaction mixture was purified by molecular weight cut off and characterized by LCMS to analyze the protein modification. Percent conversions to modified Transferrin were calculated based on the deconvolution spectrum.

MS spectrum of unmodified transferrin

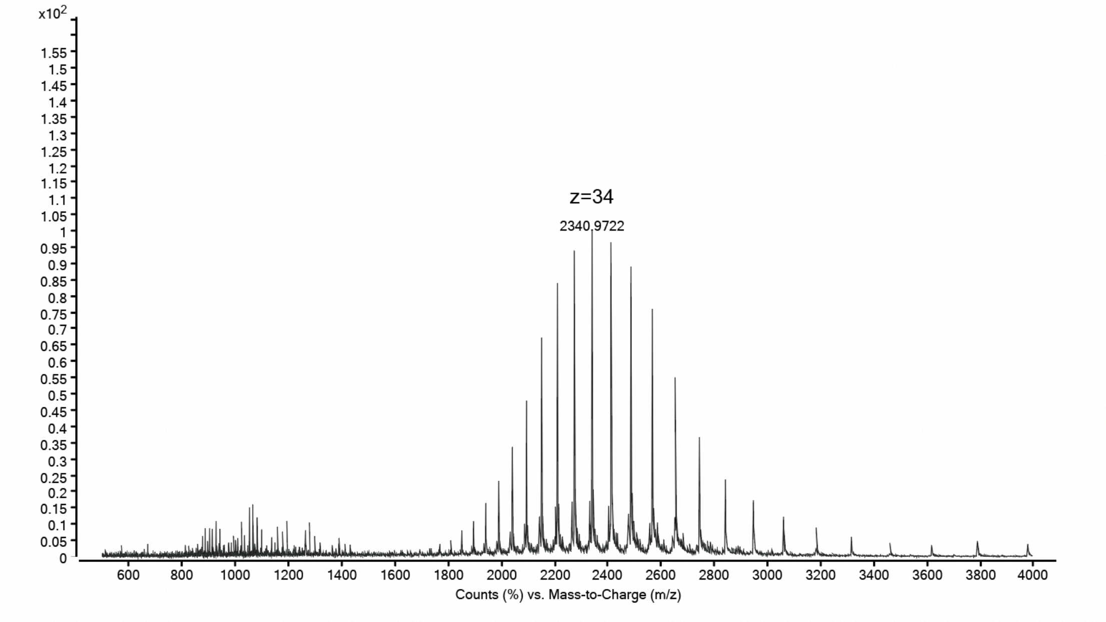

(Deconvoluted spectrum)

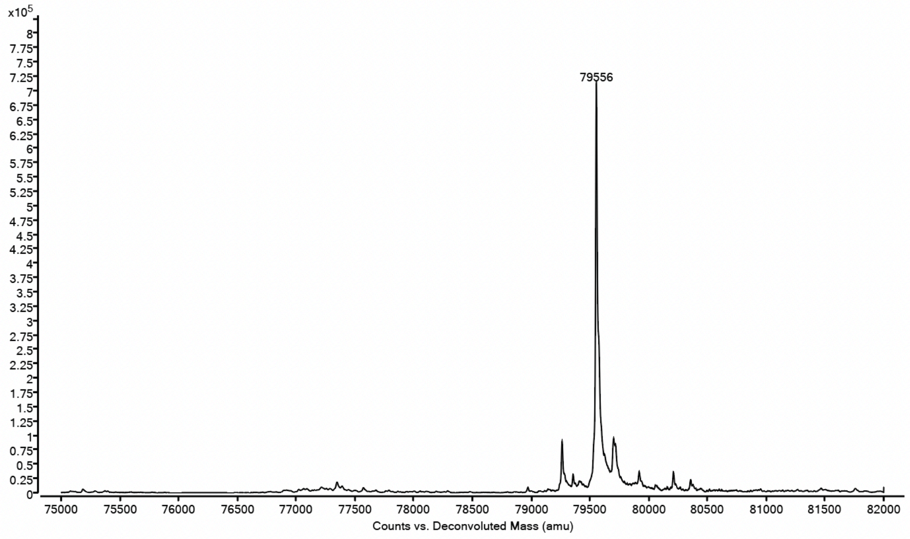

MS spectrum of modified transferrin

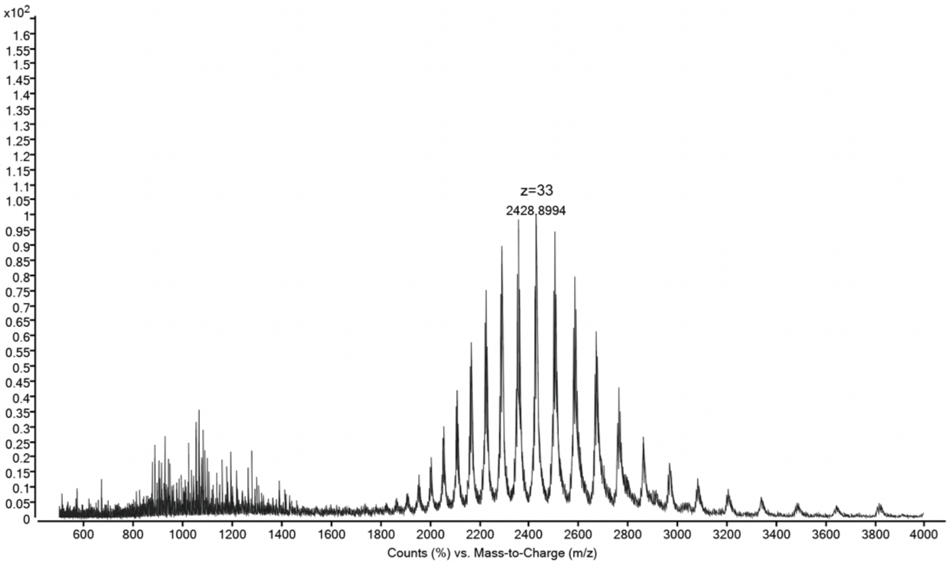

(Deconvoluted spectrum)

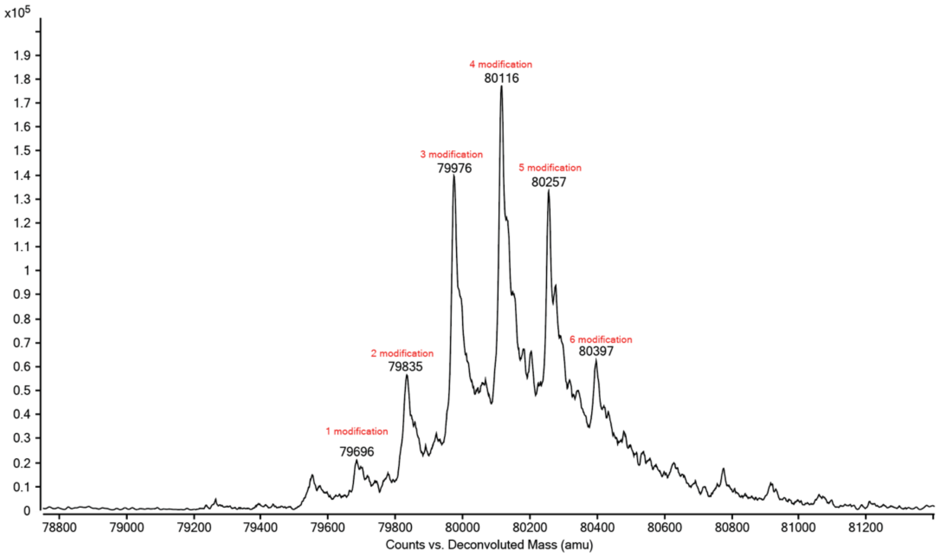

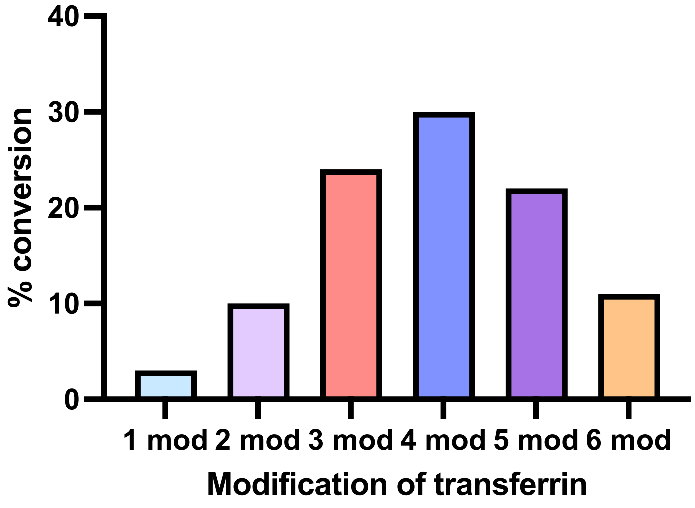

# Modifications	Expected Mass	Observed Mass	% Conversion
1 modification	79696.16	79696	3
2 modifications	79836.32	79835	10
3 modifications	79976.48	79976	24
4 modifications	80116.64	80116	30
5 modifications	80256.80	80257	22
6 modifications	80396.96	80397	11

XXIV. Supplementary Fig. 19. Selective fluorophore labeling of proteins and analysis by SDS-PAGE.

Modification of myoglobin

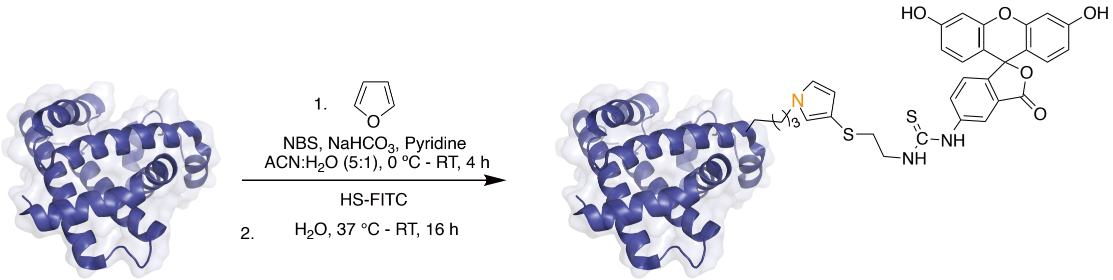

Furan (100 µL, 1.38 mmol) and sodium bicarbonate (115 mg, 1.38 mmol) were added in a solution of 12 mL acetonitrile and water (5:1). The reaction mixture was cooled to 0 °C and left to stir for 15 min. N-Bromosuccinimide (244 mg, 1.38 mmol) was dissolved in a solution of 12 mL acetonitrile and water (5:1) and added to the reaction mixture dropwise. Afterwards, the reaction mixture was left to stir for 10 min, and pyridine (222 µL, 2.76 mmol) was added to the reaction mixture. The reaction mixture was stirred for 4 h and used without further purification. From the furan pot, 1 equiv. (2.1 µL) of mixture was taken and incubated with 1-(3',6'-dihydroxy-3-oxo-3*H*-spiro[isobenzofuran-1,9'-xanthen]-5-yl)-3-(2-mercaptoethyl)thiourea (SH-FITC, 2 equiv.) at 37 °C for 15 min in 1 mL of water. 2 mg of **myoglobin** (1 equiv.) was dissolved in 2 mL of water and was added to the reaction mixture. The reaction mixture was left to stir for 16 h at RT. The reaction mixture was purified by molecular weight cut off and characterized by LCMS to analyze the protein modification. Percent conversions to modified myoglobin were calculated based on the deconvolution spectrum.

MS spectrum of fluorescently labeled myoglobin

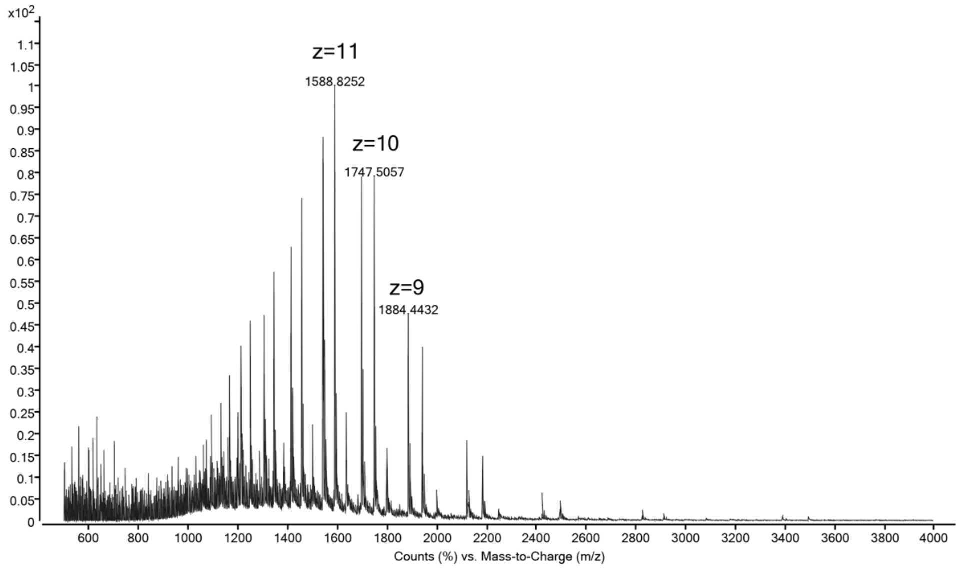

(Deconvoluted spectrum)

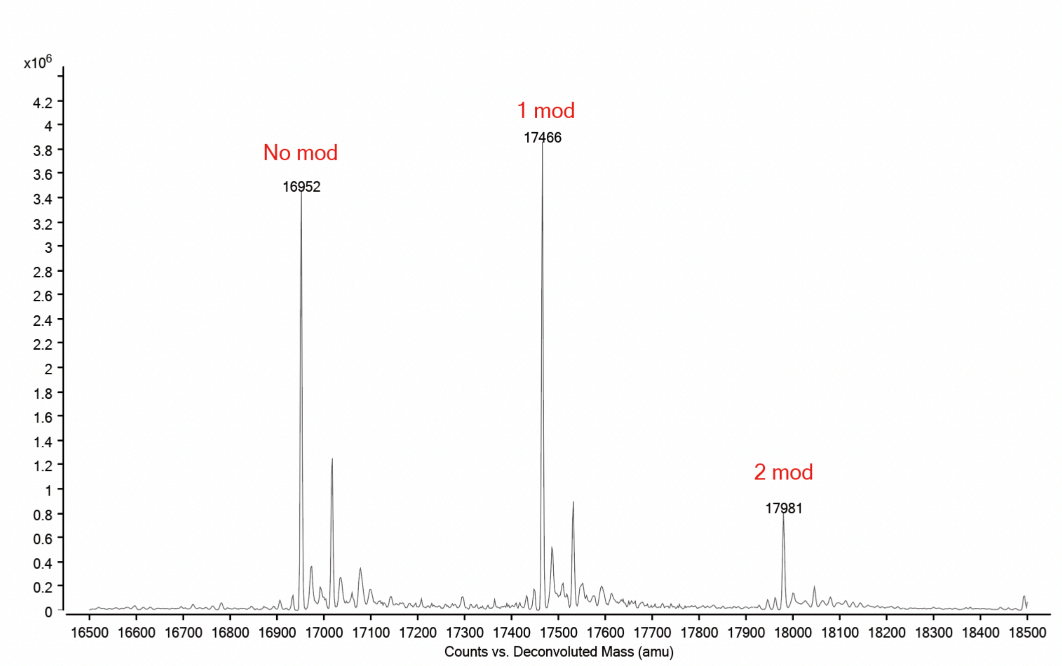

# Modifications	Expected Mass	Observed Mass	% Conversion
0 modification	16952	16952	42
1 modification	17466.57	17466	47
2 modifications	17981.14	17981	11

Fluorescent labeling amine residues on BSA, creatine kinase and Transferrin

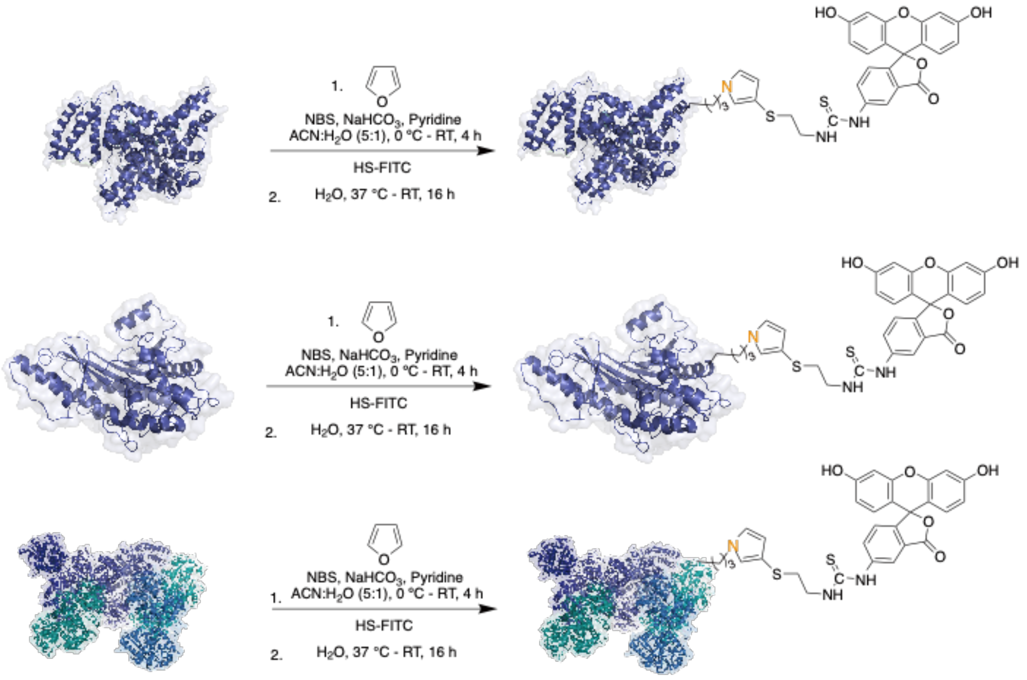

Furan (100 µL, 1.38 mmol) and sodium bicarbonate (115 mg, 1.38 mmol) were added in a solution of 12 mL acetonitrile and water (5:1). The reaction mixture was cooled to 0 °C and left to stir for 15 min. N-Bromosuccinimide (244mg, 1.38 mmol) was dissolved in a solution of 12 mL acetonitrile and water (5:1) and added to the reaction mixture dropwise. Afterwards, the reaction mixture was left to stir for 10 min, and pyridine (222 µL, 2.76 mmol) was added to the reaction mixture. The reaction mixture was stirred for 4 h and used without further purification. From the furan pot, 1 equiv. of mixture was taken and incubated with HS-FITC (2 equiv.) at 37 °C for 15 min in 1 mL of water. 2 mg of BSA, creatine kinase or transferrin (1 equiv.) was dissolved in 2 mL of water and was added to the reaction mixture. The reaction mixture was left to stir for 16 h at RT. The reaction mixture was purified by molecular weight cut off and characterized by in-gel fluorescence to analyze the protein modification. Control experiments were performed by taking a stock solution from furan pot and adding it directly to 2 mg of the protein (BSA, transferrin or Creatine kinase) and incubating it for 16 h followed by purification of protein by molecular weight and analysis by in-gel fluorescence analysis.

Full gel image of Creatine Kinase

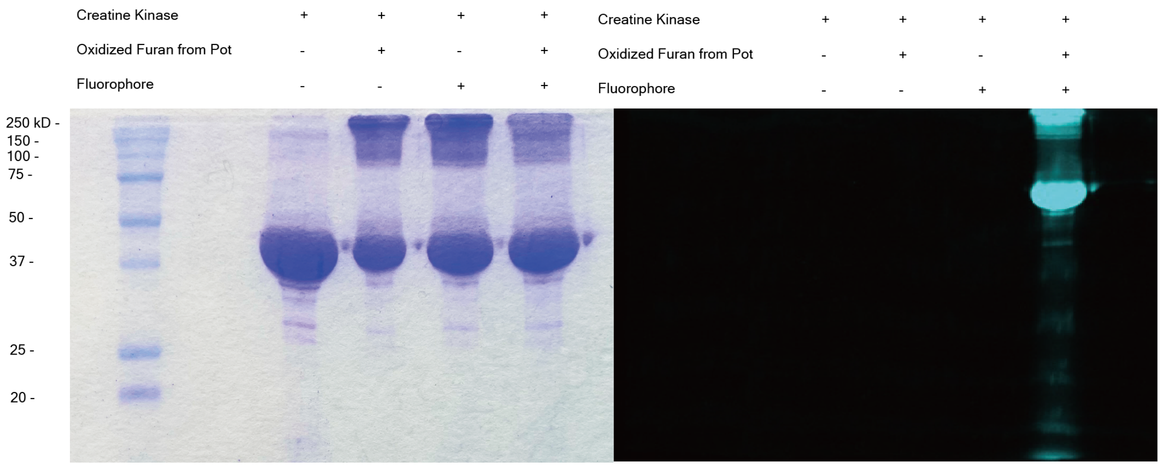

Full gel image of Transferrin Human

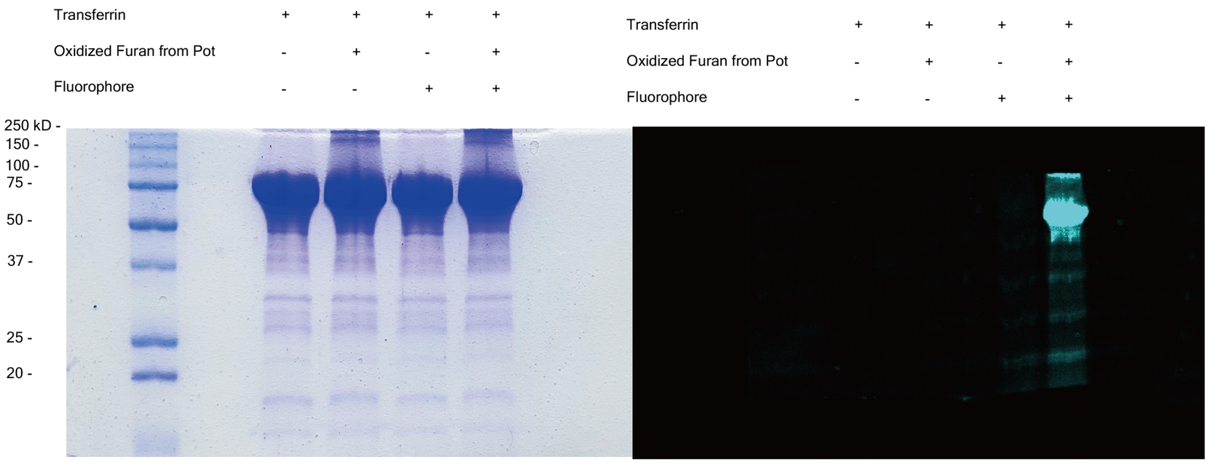

Full gel image of BSA

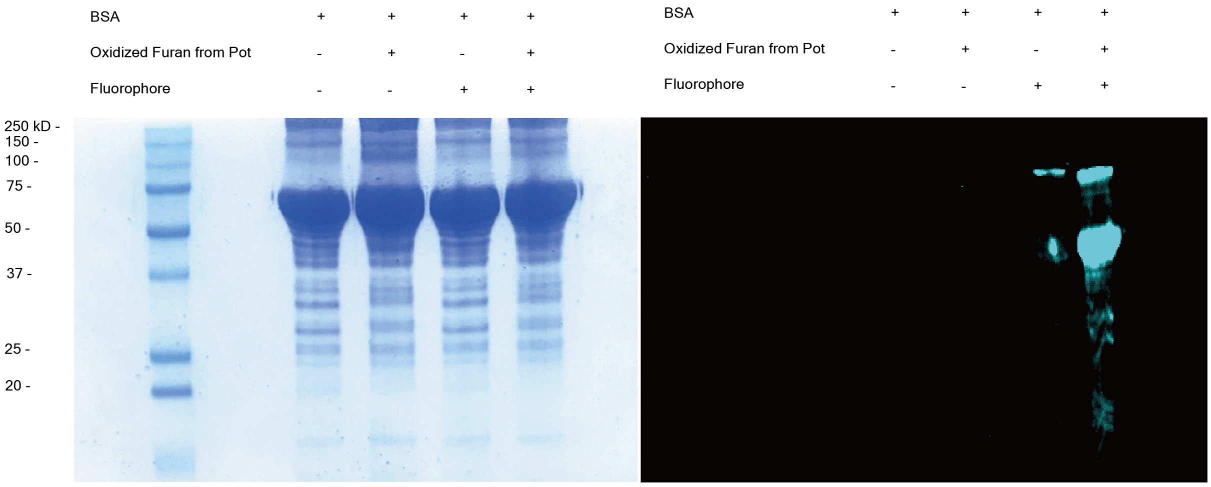

Fluorescent labeling of cysteine residues on BSA, creatine kinase and Transferrin

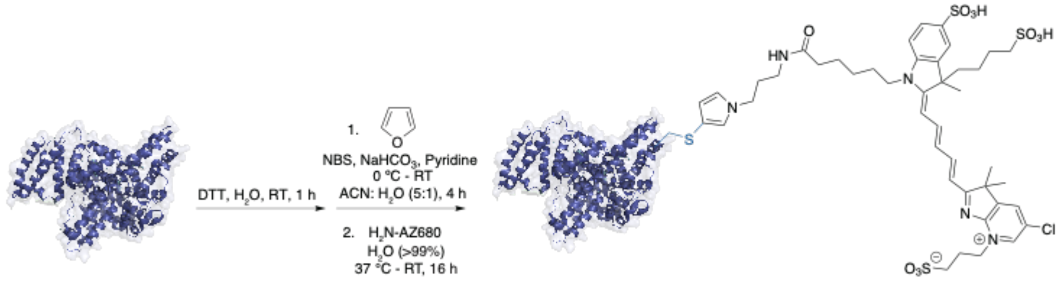

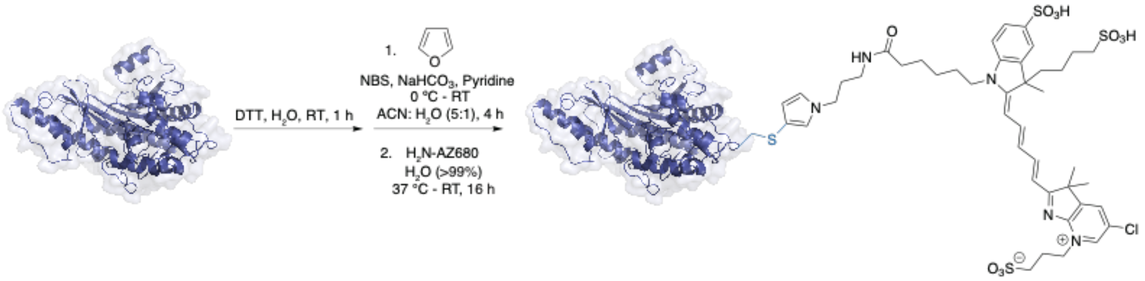

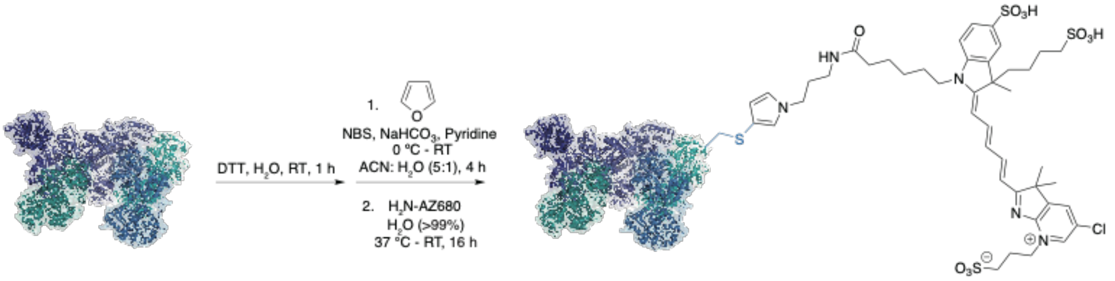

Furan (100 µL, 1.38 mmol) and sodium bicarbonate (115 mg, 1.38 mmol) were added in a solution of 12 mL acetonitrile and water (5:1). The reaction mixture was cooled to 0 °C and left to stir for 15 min. N-Bromosuccinimide (244mg, 1.38 mmol) was dissolved in a solution of 12 mL acetonitrile and water (5:1) and added to the reaction mixture dropwise. Afterwards, the reaction mixture was left to stir for 10 min, and pyridine (222 µL, 2.76 mmol) was added to the reaction mixture. The reaction mixture was stirred for 4 h and used without further purification.

6.5 mM of dithiothreitol in water was prepared and protein samples were incubated in the DTT solution (1 mL) at room temperature prior to FuTine labeling.

From the furan pot, 5 equiv. of mixture was taken and incubated with the protein samples (2 mg of BSA, creatine kinase or transferrin**)** for 15 minutes. AZ680 amine dye (5 equiv.) was added to the reaction mixture and was left to stir for 16 h at RT. The reaction mixture was purified by molecular weight cut off and characterized by in-gel fluorescence to analyze the protein modification. Control experiments were performed by taking a stock solution from furan pot and adding it directly to 2 mg of the protein (BSA, transferrin or Creatine kinase) and incubating it for 16 h followed by purification of protein by molecular weight and analysis by in-gel fluorescence analysis.

Full gel image of Creatine Kinase

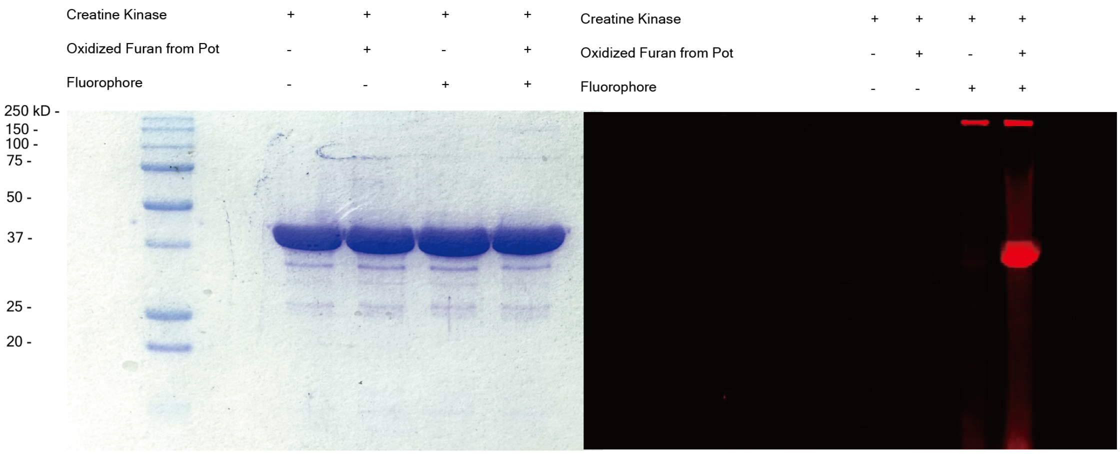

Full gel image of Transferrin Human

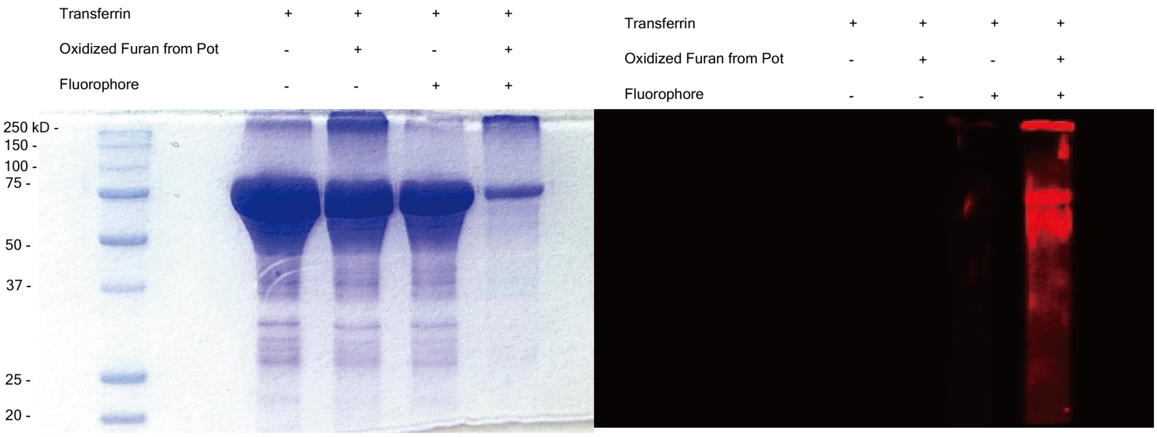

Full gel image of BSA

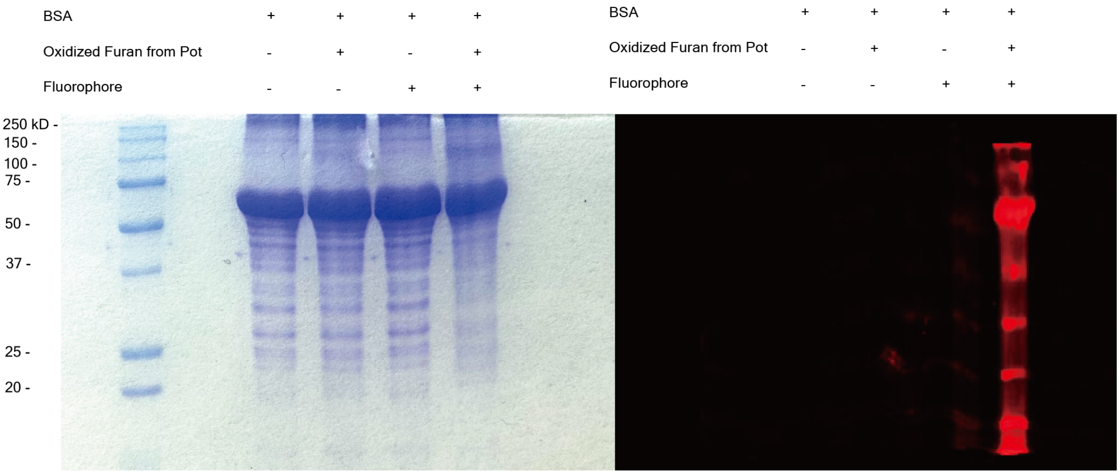

XXV. Supplementary Fig. 20. Cyclization of myoglobin by furan-thiol-amine multicomponent reaction.

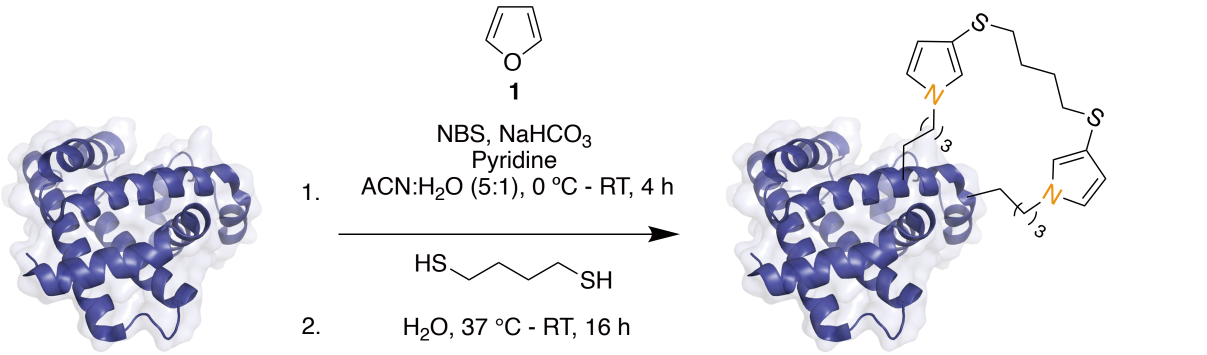

Furan **1** (100 µL, 1.38 mmol) and sodium bicarbonate (115 mg, 1.38 mmol) were added in a solution of 12 mL acetonitrile and water (5:1). The reaction mixture was cooled to 0 °C and left to stir for 15 min. N-Bromosuccinimide (244 mg, 1.38 mmol) was dissolved in a solution of 12 mL acetonitrile and water (5:1) and added to the reaction mixture dropwise. Afterwards, the reaction mixture was left to stir for 10 min, and pyridine (222 µL, 2.76 mmol) was added to the reaction mixture. The reaction mixture was stirred for 4 h and used without further purification. From the furan pot, 2 equiv. (4.1 µL) of mixture was taken and incubated with 1,4-butanedithiol (1 equiv.) at 37 °C for 30 m in 1 mL of water. 2 mg of **myoglobin** (1 equiv.) was dissolved in 2 mL of water and added to the reaction mixture. The reaction mixture was left to stir for 16 h at RT. The reaction mixture was purified by molecular weight cut off and characterized by LCMS to analyze the protein modification. Percent conversions to cyclized myoglobin were calculated based on the deconvolution spectrum (38 %).

MS spectrum of cyclized myoglobin

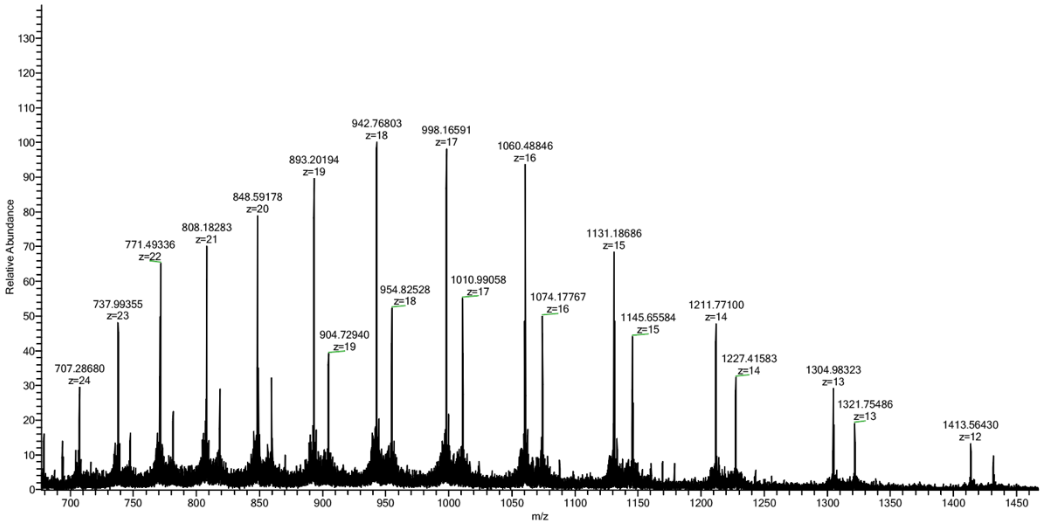

(Deconvoluted spectrum)

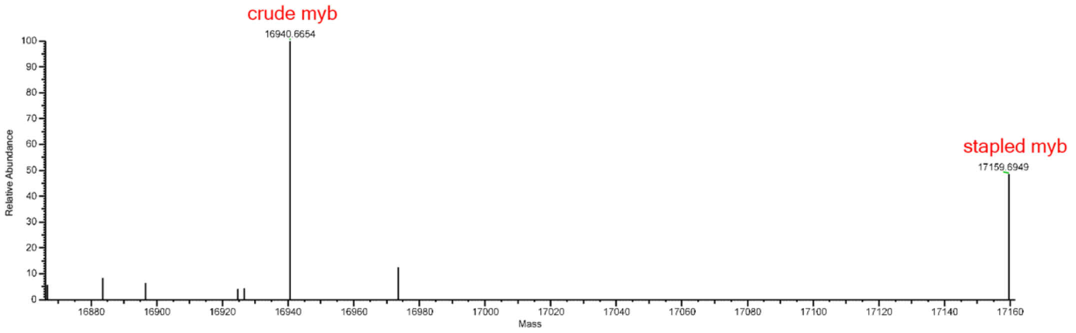

# Modifications	Expected Mass	Observed Mass	% Conversion
no modifications	16940.8946	16940.6654	62 %
1 modification	17158.9146	17159.6946	38 %

XXVI. Supplementary Fig. 21. Dual modification of proteins.

Modification of tyrosine on transferrin

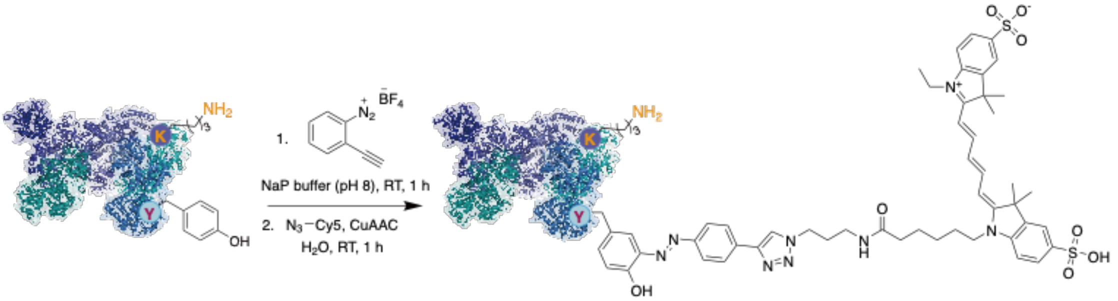

2 mg of **transferrin human** (1 equiv.) was dissolved in 500 µL of sodium phosphate buffer (pH 8). The stock solution of 2-ethynylbenzenediazonium tetrafluoroborate (2 mg in 100 µL ACN) was prepared and 5.5 µL of the stock solution (10 equiv.) was added to the reaction. The reaction mixture was left to stir for 1 h at RT. The reaction mixture was purified by molecular weight cut off and lyophilized.

Diazonium modified **transferrin human** (~1.7 mg) was dissolved in 300 µL of water. To the reaction mixture, 20 µL of 100 mM Tris(benzyltriazolylmethyl)amine (THPTA), 20 µL of 20 mM $CuSO_4$ solution, 20 µL of 300 mM sodium ascorbate solution and 5 µL of 10 mM Cy5-azide dye were added to the reaction mixture. The reaction was left to stir at RT for 1 h. The reaction mixture was purified by molecular weight cut off and SDS-PAGE gel electrophoresis was performed to analyze the results.

Modification of lysine on transferrin

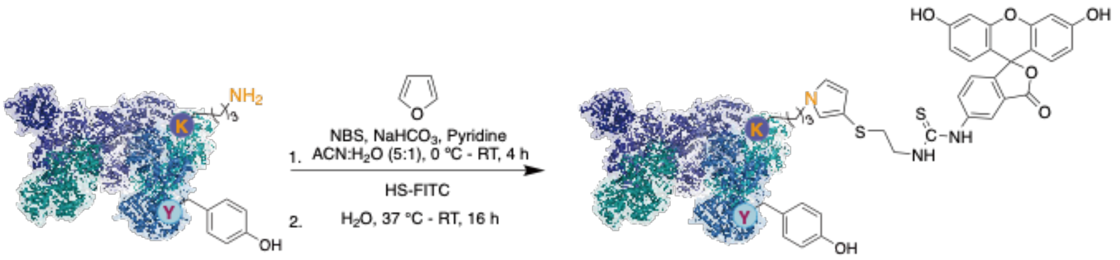

Furan **1** (100 µL, 1.38 mmol) and sodium bicarbonate (115 mg, 1.38 mmol) were added in a solution of 12 mL acetonitrile and water (5:1). The reaction mixture was cooled to 0 °C and left to stir for 15 min. N-Bromosuccinimide (244 mg, 1.38 mmol) was dissolved in a solution of 12 mL acetonitrile and water (5:1) and added to the reaction mixture dropwise. Afterwards, the reaction mixture was left to stir for 10 min, and pyridine (222 µL, 2.76 mmol) was added to the reaction mixture. The reaction mixture was stirred for 4 h and used without further purification. From the furan pot, 1 equiv. (0.5 µL) of mixture was taken and incubated with 1-(3',6'-dihydroxy-3-oxo-3*H*-spiro[isobenzofuran-1,9'-xanthen]-5-yl)-3-(2-mercaptoethyl) thiourea (2 equiv.) at 37°C for 30 min in 1 mL of water. 2 mg of transferrin human (1 equiv.) was dissolved in 2 mL of water and was added to the reaction mixture. The concentration of protein in the reaction was 8 µM. The reaction mixture was left to stir for 16 h at RT. The reaction mixture was purified by molecular weight cut off and SDS-PAGE gel electrophoresis was performed to analyze the results.

Dual Modification

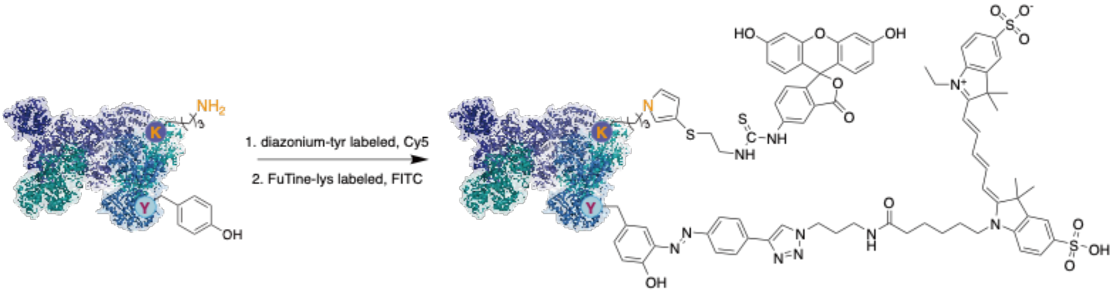

2 mg of **transferrin human** (1 equiv.) was dissolved in 500 µL of sodium phosphate buffer (pH 8). The stock solution of 2-ethynylbenzenediazonium tetrafluoroborate (2 mg in 100 µL ACN) was prepared and 5.5 µL of the stock solution (10 equiv.) was added to the reaction. The reaction mixture was left to stir for 1 h at RT. The reaction mixture was purified by molecular weight cut off and lyophilized. Diazonium modified transferrin human (~1.7 mg) was dissolved in 300 µL of water. To the reaction mixture, 20 µL of 100 mM THPTA, 20 µL of 20 mM $CuSO_4$ solution, 20 µL of 300 mM sodium ascorbate solution and 5 µL of 10 mM Cy5-azide dye were added to the reaction mixture. The reaction was left to stir at RT for 1 h. The reaction mixture was purified by molecular weight cut off and lyophilized.

Furan **1** (100 µL, 1.38 mmol) and sodium bicarbonate (115 mg, 1.38 mmol) were added in a solution of 12 mL acetonitrile and water (5:1). The reaction mixture was cooled to 0 °C and left to stir for 15 min. N-Bromosuccinimide (244 mg, 1.38 mmol) was dissolved in a solution of 12 mL acetonitrile and water (5:1) and added to the reaction mixture dropwise. Afterwards, the reaction mixture was left to stir for 10 min, and pyridine (222 µL, 2.76 mmol) was added to the reaction mixture. The reaction

mixture was stirred for 4 h and used without further purification. From the pot, 1 equiv. of oxidized furan was taken and incubated with 1-(3',6'-dihydroxy-3-oxo-3*H*-spiro[isobenzofuran-1,9'-xanthen]-5-yl)-3-(2-mercaptoethyl) thiourea HS-FITC (2 equiv.) at 37 °C for 30 min in 1 mL of water. 1 mg of diazonium Cy5 was added to modified transferrin human (1 equiv.) was dissolved in 2 mL of water and was added to the reaction mixture. The reaction mixture was left to stir for 16 h at RT. The reaction mixture was purified by molecular weight cut off and SDS-PAGE gel electrophoresis was performed to analyze the results.

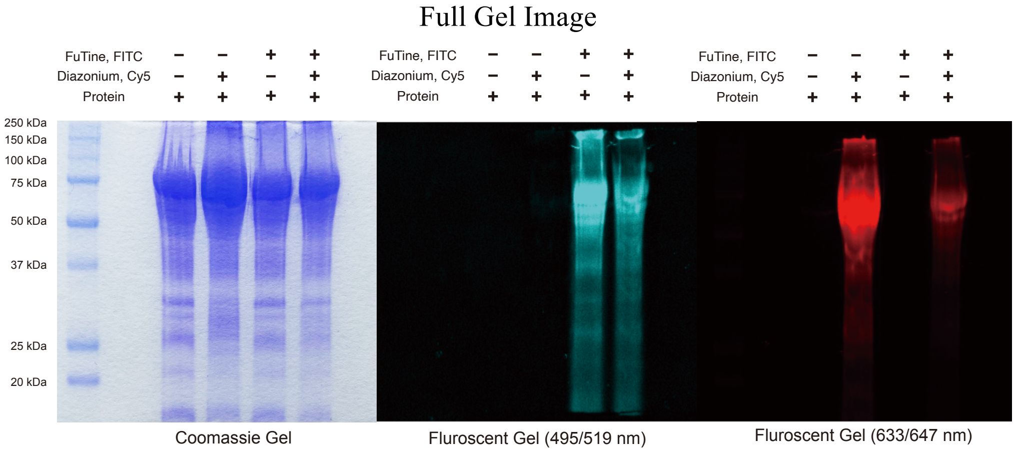

XXVII. Supplementary Fig. 22. Selective fluorophore labeling of proteins in complex cell lysates and analysis by SDS-PAGE.

Cell Lysate preparation procedures

Lncap cells were removed from the incubator and the media was aspirated immediately using vacuum. Cells were washed with ice cold PBS and aspirated again. Cells were scraped out of the dish on ice and transferred to 1.5 mL centrifuge tubes. Tubes were centrifuged for 5 minutes at 300 x g at 4 °C to pellet the cells and the excess PBS was removed and centrifuged tubes were kept on ice. Cell pellets were resuspended in RIPA buffer and incubated on ice for 10 minutes. The tubes were centrifuged at 10000 x g for 10 minutes at 4 °C. The supernatants was removed to the final storage tubes and stored at -20 °C.

Labeling amine residues in cell lysates

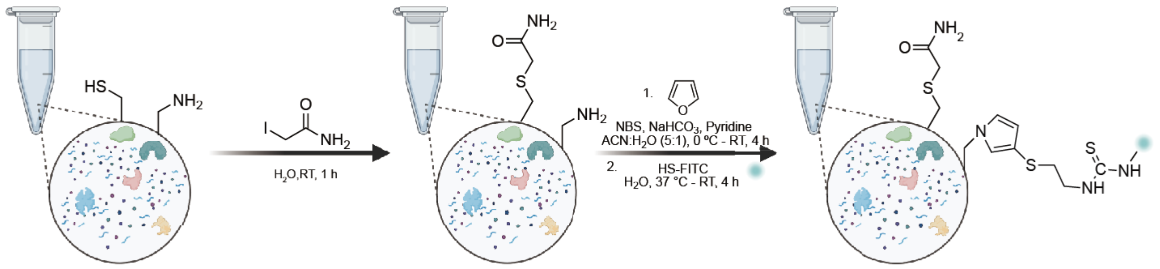

To block free thiol from forming disulfide linkage with the fluorophore, the free thiol on cell lysates were blocked using 100 µL solution of iodoacetamide (15 mM) in water for 1 hour. The reaction. The cell lysates were precipitated out using acetone and centrifuge at 1500 x g for 10 min at 4 ºC. The

supernatant was removed and the samples were ready for lysine labeling. To prepare for lysine labeling, furan (100 µL, 1.38 mmol) and sodium bicarbonate (115 mg, 1.38 mmol) were added in a solution of 12 mL acetonitrile and water (5:1). The reaction mixture was cooled to 0 °C and left to stir for 15 min. N-Bromosuccinimide (244 mg, 1.38 mmol) was dissolved in a solution of 12 mL acetonitrile and water (5:1) and added to the reaction mixture dropwise. Afterwards, the reaction mixture was left to stir for 10 min, and pyridine (222 µL, 2.76 mmol) was added to the reaction mixture. The reaction mixture was stirred for 4 h and used without further purification. From the pot, 1 mM of oxidized furan were incubated with 2 µL of 33 mM 1-(3',6'-dihydroxy-3-oxo-3*H*-spiro[isobenzofuran-1,9'-xanthen]-5-yl)-3-(2-mercaptoethyl) thiourea solution in 50 µL of water at 37 ºC for 30 min. 200 µg of cysteine blocked cell lysate was dissolved in 200 µL of water and added to the reaction mixture. The reaction mixture was left to stir for 4 h at RT. Cell lysate was precipitated using cold acetone and analyzed using SDS-PAGE in-gel fluorescence analysis. Control experiments were performed by taking a stock solution from furan pot and adding it directly in a cell lysate sample and incubating it for 4 h followed by purification of protein by molecular weight and analysis by in-gel fluorescence analysis.

Competition inhibition assay for lysine labeling

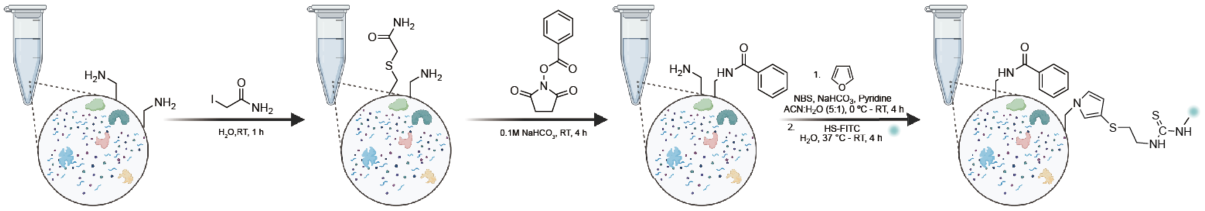

To block free thiol from forming disulfide linkage with the fluorophore, the free thiol on cell lysates were blocked using 100 µL solution of iodoacetamide (15 mM) in water for 1 hour. The reaction. The cell lysates were precipitated out using acetone and centrifuge at 1500 x g for 10 min at 4 ºC. The supernatant was removed, and the sample was incubated with a various concentration of 2,5-dioxopyrrolidin-1-yl benzoate in 0.1 M $NaHCO_3$ for 4 h at room temperature. The cell lysate was precipitated out using acetone and centrifuge at 1500 x g for 10 minutes at 4 ºC. To prepare for lysine labeling, furan (100 µL, 1.38 mmol) and sodium bicarbonate (115 mg, 1.38 mmol) were added in a solution of 12 mL acetonitrile and water (5:1). The reaction mixture was cooled to 0 °C and left to stir for 15 min. N-Bromosuccinimide (244 mg, 1.38 mmol) was dissolved in a solution of 12 mL acetonitrile and water (5:1) and added to the reaction mixture dropwise. Afterwards, the reaction mixture was left to stir for 10 min, and pyridine (222 µL, 2.76 mmol) was added to the reaction mixture. The reaction mixture was stirred for 4 h and used without further purification. From the pot, 1 mM of oxidized furan were incubated with 2 µL of 33 mM 1-(3',6'-dihydroxy-3-oxo-3*H*-spiro[isobenzofuran-1,9'-xanthen]-5-yl)-3-(2-mercaptoethyl) thiourea solution in 50 µL of water at 37 ºC for 30 min. ~100 µg of amine and cysteine blocked cell lysate was dissolved in 200 µL of water and added to the reaction mixture. The reaction mixture was left to stir for 4 h at RT. Cell lysate was precipitated using cold acetone and analyzed using SDS-PAGE in-gel fluorescence analysis. Loss of fluorescent intensity was observed with the addition of NHSester analog. Lane 1: No NHSester analog was added, Lane 2: 1 mM NHSester was used to block amine residues, Lane 3: 5 mM NHSester was used to block amine residues.

Full gel imaging of lysine competition assay

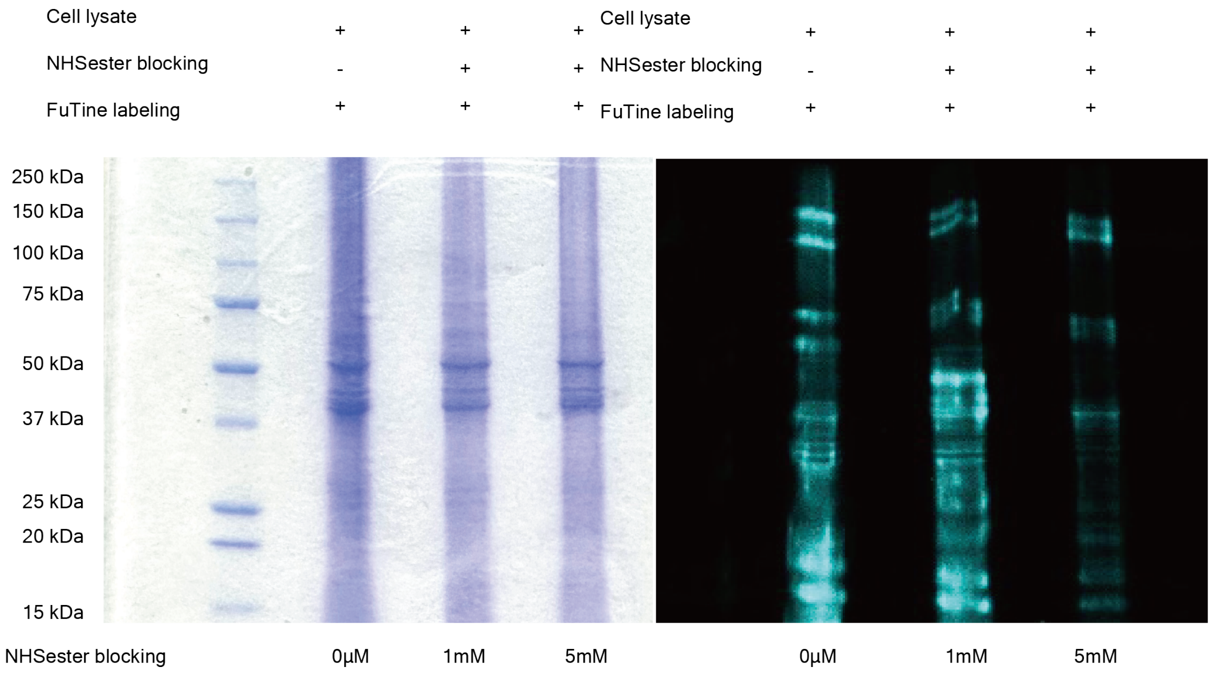

Labeling cysteine residues in cell lysates

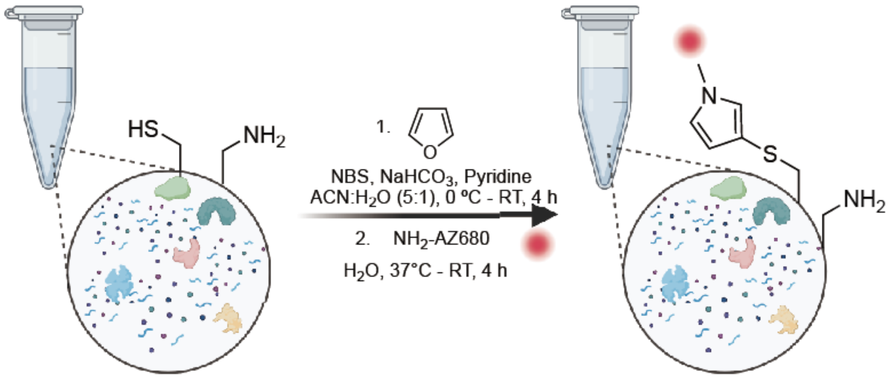

Furan (100 µL, 1.38 mmol) and sodium bicarbonate (115 mg, 1.38 mmol) were added in a solution of 12 mL acetonitrile and water (5:1). The reaction mixture was cooled to 0 °C and left to stir for 15 min. N-Bromosuccinimide (244 mg, 1.38 mmol) was dissolved in a solution of 12 mL acetonitrile and water (5:1) and added to the reaction mixture dropwise. Afterwards, the reaction mixture was left to stir for 10 min, and pyridine (222 µL, 2.76 mmol) was added to the reaction mixture. The reaction mixture was stirred for 4 h and used without further purification. From the furan pot, 1 mM of oxidized furan were incubated with 100 µg of cell lysate in 100 µL of water and incubated at 37 ºC for 10 min. 3 µL of 10 mM AZ680 amine dye in DMSO were added to the reaction mixture. The reaction mixture was left to stir for 4 h at RT. Cell lysate was precipitated using cold acetone and analyzed using SDS-PAGE in-gel fluorescence analysis. Control experiments were performed by taking a stock solution from furan pot and adding it directly in a cell lysate sample and incubating it

for 4 h followed by purification of protein by molecular weight and analysis by in-gel fluorescence analysis.

Competition inhibition assay for cysteine labeling

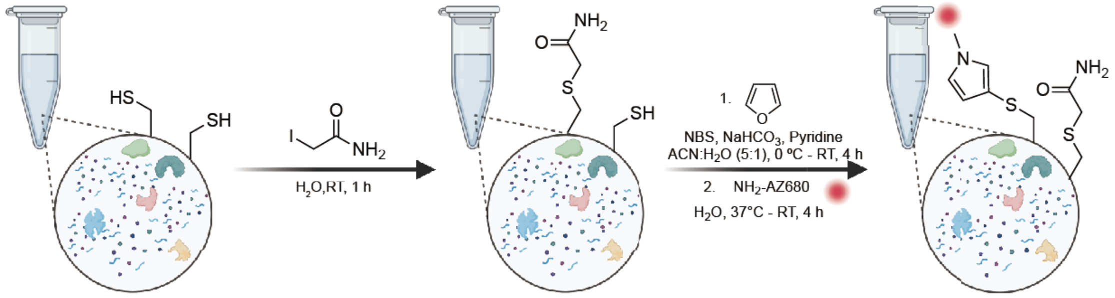

To block free thiol from forming disulfide linkage with the fluorophore, the free thiol on cell lysates were blocked using 100 µL solution of iodoacetamide in various concentration for 1 hour. The reaction. The cell lysate was precipitated out using acetone and centrifuge at 1500 x g for 10 min at 4 ºC. To prepare for cysteine labeling, furan (100 µL, 1.38 mmol) and sodium bicarbonate (115 mg, 1.38 mmol) were added in a solution of 12 mL acetonitrile and water (5:1). The reaction mixture was cooled to 0 °C and left to stir for 15 min. N-Bromosuccinimide (244 mg, 1.38 mmol) was dissolved in a solution of 12 mL acetonitrile and water (5:1) and added to the reaction mixture dropwise. Afterwards, the reaction mixture was left to stir for 10 min, and pyridine (222 µL, 2.76 mmol) was added to the reaction mixture. The reaction mixture was stirred for 4 h and used without further purification. From the furan pot, 1 mM of oxidized furan were incubated with 100 µg of cell lysate in 100 µL of water and incubated at 37 ºC for 10 min. 2 µL of 10 mM AZ680 amine dye in DMSO were added to the reaction mixture. The reaction mixture was left to stir for 4 h at room temperature. Cell lysate was precipitated using cold acetone and analyzed using SDS-PAGE in-gel fluorescence analysis. Control experiments were performed by taking a stock solution from furan pot and adding it directly in a cell lysate sample and incubating it for 4 h followed by purification of protein by molecular weight and analysis by in-gel fluorescence analysis. Loss of fluorescent intensity was observed with the addition of NHSester analog. Lane 1: No NHSester analog was added, Lane 2: 500 µM iodoacetamide was used to block free cysteine residues, Lane 3: 1 mM NHSester was used to block free cysteine residues.

Full gel imaging of cysteine competition assay

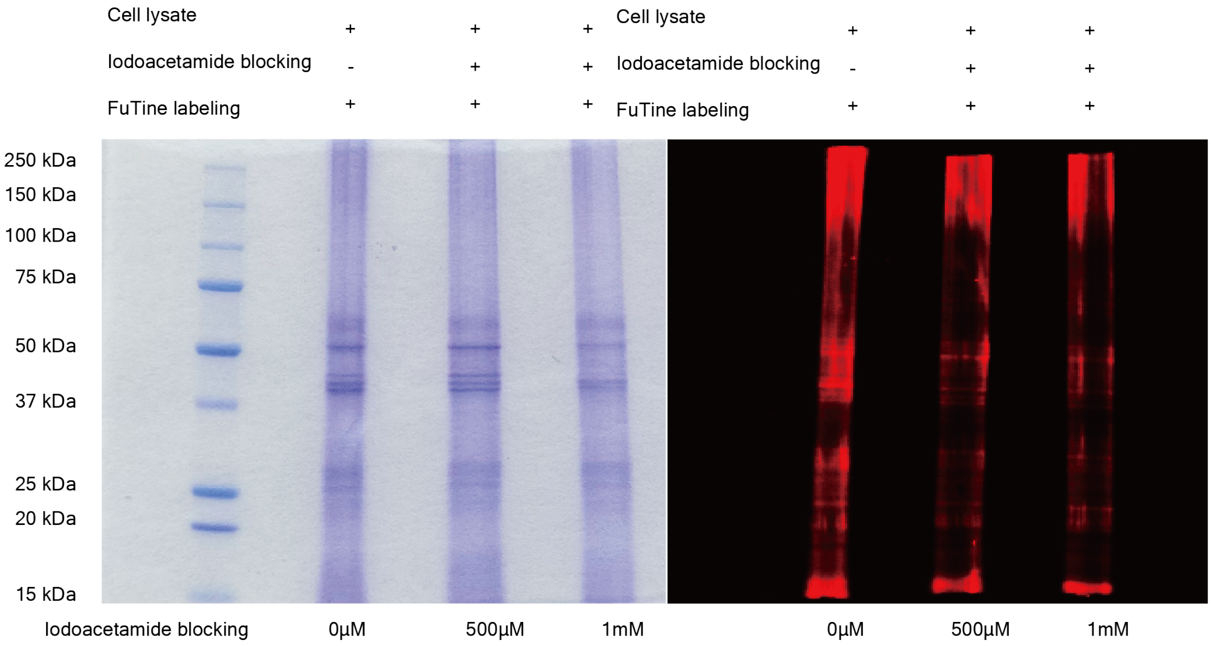

XXVIII. Supplementary References

1. Chan, W. C. & White, P. D. Fmoc solid phase peptide synthesis: A practical approach (Oxford Univ. Press, New York, 2000).

2. Mahesh, S., Adebomi, V., Muneeswaran, Z. P. & Raj. M. Bioinspired nitroalkylation for selective protein modification and peptide stapling. *Angew. Chem. Int. Ed.* **59**, 2793-2801 (2020).
